# ﻿An annotated checklist of the vascular plants of Taita Hills, Eastern Arc Mountain

**DOI:** 10.3897/phytokeys.191.73714

**Published:** 2022-03-01

**Authors:** Benjamin Muema Watuma, Solomon Kipkoech, David Kimutai Melly, Veronicah Mutele Ngumbau, Peninah Cheptoo Rono, Fredrick Munyao Mutie, Elijah Mbadi Mkala, John Mulinge Nzei, Geoffrey Mwachala, Robert Wahiti Gituru, Guang-Wan Hu, Qing-Feng Wang

**Affiliations:** 1 Core Botanical Gardens/Wuhan Botanical Garden, Chinese Academy of Sciences, Wuhan 430074, Hubei, China Wuhan Botanical Garden Wuhan China; 2 Sino-Africa Joint Research Center (SAJOREC), Chinese Academy of Sciences, Wuhan 430074, Hubei, China Jomo Kenyatta University of Agriculture and Technology Nairobi Kenya; 3 University of Chinese Academy of Sciences, Beijing 100049, China Sino-Africa Joint Research Center Wuhan China; 4 East African Herbarium, National Museums of Kenya, P. O. Box 45166 - 00100 Nairobi, Kenya University of Chinese Academy of Sciences Beijing China; 5 Botany Department, Jomo Kenyatta University of Agriculture and Technology, Nairobi, Kenya Kenya Forestry Research Institute Nairobi Kenya; 6 Bomet University College, P.O Box 701-20400. Bomet, Kenya Bomet University College Bomet Kenya; 7 Kenya Forestry Research Institute (KEFRI), P.O Box 20412–00200 Nairobi, Kenya National Museums of Kenya Nairobi Kenya

**Keywords:** Biodiversity, checklist, conservation, Eastern arc mountains

## Abstract

Taita Hills forests are an ecological island within the Tsavo plains and are the northern-most part of the Eastern Arc Mountains in southeast Kenya. They are highly fragmented forests embedded in a mosaic of human settlements and farms on the slopes and hilltops. Despite their intensive degradation, they exhibit a high degree of plant diversity and endemism, and therefore are regarded as a biodiversity hotspot. In spite of their distinct importance to the biodiversity of the region as well as supporting the livelihoods of the surrounding communities, floristic studies in these hills have been finite. Through repetitive floral expeditions, herbarium records from the East African Herbarium (EA), Global Biodiversity Information (GBIF), and the Integrated Digitized Biocollections (iDigbio) databases, as well as plant lists from literature and monographs, we provide a comprehensive checklist of 1594 taxa representing 159 families, 709 genera, 1530 species, 39 subspecies, 27 varieties, and 2 hybrids. Out of these, 75 are endemic or near-endemic, 59 are exotic, and 83 are listed as either endangered or near endangered as evaluated in the IUCN Redlist. *Zehneriatuberifera* G.W.Hu & Q.F.Wang, a new species to science, which has previously been described, was also discovered from the Ngangao forest fragment. Information on the habit(s), habitat(s), and altitudinal range of each taxon is provided in this study. This checklist is an updated inventory of the vascular plants of the Taita Hills. It confirms the high plant diversity of the hills and provides a clear baseline for strategic conservation and sustainable management of plant resources and diversity under the Convention on Biological Diversity (CBD).

## ﻿Introduction

Global biodiversity loss has been widely attributed to forest loss and fragmentation, which are considered as its two principal factors (Newmark 1998; Brooks et al. 2002). Fragmentation oftentimes is superseded by over-exploitation, degradation, and introduction of exotic species, thus decreasing indigenous plant diversity (Newmark 1998; Cordeiro and Howe 2001; Teucher et al. 2020). This ensuing biodiversity crisis has increased interest in conservation planning and prioritization strategies (Turner et al. 2007; Brooks 2010; Krupnick 2013; Habel et al. 2016; Ndang’ang’a et al. 2016; Conservation International 2021). Since the distribution and decline in species diversity are variable across the biomes, Myers (1988) proposed biodiversity hotspots for conservation prioritization (Mittermeier et al. 1998; Myers et al. 2000; Carrasco et al. 2020). The majority of hotspots are located in high densely populated areas and therefore are under high pressure due to demand for land and forest resources (Cincotta et al. 2000; Asongu and Jingwa 2012; Habel et al. 2016). The average population density within the hotspots has increased by 36% between the years 1995–2015 (Cunningham and Beazley 2018), and approximately two billion people live within the hotspots (CEPF 2021). Although a high human population does not directly guarantee habitat loss, the resulting human impact leads to it (Mittermeier et al. 2004; CEPF 2021). Twenty-two of the hotspots within the tropics are more affected, retaining a lesser proportion of their indigenous vegetation compared to those in temperate and subtropical zones (Hrdina and Romportl 2017).

Tropical montane cloud forests naturally have a small area of occupancy on moist ridge tops with a frequency of clouds (Lovett 1998a). These forests have been highlighted arguably as the world’s most important tropical forests and diversity hotspots (Myers 1993; Lovett 1998a). Due to the isolation of these ecological islands, they have a high endemic species richness (Hamilton et al. 1995; Lovett 1998a, 1998b). The lower elevations of these ecosystems have been highly transformed mainly due to the conversion of forest to farmlands and, therefore, the forests serve as refugia for endangered species (Hamilton et al. 1995; Lovett 1998a). Cloud forests have low resilience to disturbance and their regeneration is fairly slow (Lovett 1998a). These forests provide a wide range of ecosystem services for millions of people living in the tropics. They are major watershed areas, providing timber, food, and medicinal plants for the local communities, acting as carbon sinks, and preventing denudation of hilltops through soil erosion (Myers 1993; Hamilton et al. 1995; Medley and Kalibo 2007).

The Eastern Arc Mountains (EAM) are part of the larger Eastern Afromontane Biodiversity Hotspot (EABH) which is ranked 9^th^ in terms of hotspot importance (Hrdina and Romportl 2017). They are ancient crystalline mountains uplifted 30 million years ago stretching from the Taita Hills in Kenya to the Udzungwa Mountains in Tanzania (Burgess et al. 2007; Thijis et al. 2013). The EAM have been the center of numerous studies over the years delineating them as a key center of endemism (Beentje 1996; Lovett 1998b; Mittermeier et al. 1998; Myers et al. 2000; Gereau et al. 2005; Isango 2007; Linder 2014). Their unique flora has evolved gradually under environmental stability and thus their vegetation is fragile and susceptible to large-scale disturbances (Lovett 1998a). Previously under the Eastern Arc Mountains and Coastal Forests of Tanzania and Kenya Hotspot, they were considered highly likely to suffer most extinction events from a given loss of habitat (Brooks et al. 2002). It’s estimated that 31% of the species in the EAM are extinct or in danger of extinction (Newmark 1998).

Taita Hills forests are one of the most endemic-rich montane forests of the Eastern Arc (Lovett 1998a), and the smallest in size, with only 6 km^2^ of natural forests remaining, having lost 98% of their original forest cover (Newmark 1998; Burgess et al. 2007). 50% of the indigenous vegetation has been cleared in the last 60 years (Pellikka et al. 2013). Despite their small size and a high degree of fragmentation and degradation, these forests host a high diversity of both vascular and non-vascular plants, amphibians, reptiles and are also an Important Bird Area (IBA) (Beentje 1988; Bytebier 2001; Enroth et al. 2013, 2019; BirdLife International 2021).

Plant collection records in the Taita Hills span from as early as 1877 by a German botanist, Johan Maria Hildebrandt (Beentje 1998). Subsequently, other botanical explorations have been conducted, yielding substantial herbarium collections but lacking sufficient literature on the flora and vegetation (Beentje 1988). In 1985, a team of multidisciplinary experts carried an expedition in the Taita Hills courtesy of the National Museums of Kenya, focusing on the indigenous forest patches to ascertain their remaining size, status, and threats. They recorded 475 plant taxa representing 110 families and 283 genera, providing the first floral checklist of the indigenous fragmented forests (Beentje 1988). Ecological and ethnobotanical surveys carried out in Mt. Kasigau from 2002–2006 recorded 338 wild woody plants of economical and medicinal value to the local communities (Medley and Kalibo 2007). Finally, Thijis et al. (2013) published a field guide to the woody plant species of the Taita Hills indigenous relics recording 184 species and their uses.

Studies on the vegetational structure and health monitoring of these forests indicated that, between the years 1955–2004 forest cover within Taita Hills has reduced by 50% and the notable increase of 2% has been due to a growth in exotic plantations. In the heavily disturbed sites, selective logging has led to the dominance of secondary successive species and there is also an indication of a loss of endemic species within forest patches due to fragmentation (Wilder et al. 1998; Chege and Bytebier 2005; Maria 2007; Rodgers et al. 2008; Spanhove and Lehouck 2008; Pellikka et al. 2009, 2013; Omoro et al. 2010; Aerts et al. 2011; Medley and Maingi 2014). The woodland vegetation along the slopes has unique floristic affinities and is an important source of renewable natural resources for the local community. By meeting the community’s needs, it helps in maintaining the evergreen forests for watershed protection (Rogo and Oguge 2000; Medley and Kalibo 2005; Himberg et al. 2009; Mwamidi et al. 2012; Medley et al. 2017). These hills exhibit a high biodiversity of both vascular and non-vascular plants (Enroth et al. 2013; 2019).

Floristic data for an area provides foundational tools for research, management, and policy making for the preservation, restoration, and use of biodiversity (Palmer et al. 1995). An unprecedented rate of extinction may exceed that of species recovery (Wheeler et al. 2012) and therefore prompting a crucial need for floristic inventories (Palmer et al. 1995). In Kenya, an updated flora checklist of several key areas of biodiversity importance such as Mount Elgon (Tweedie 1976), Shimba Hills (Luke 2005), Kakamega (Fischer et al. 2010), Mount Kenya (Zhou 2017), Cherengani Hills (Mbuni et al. 2019), Coastal forests of Kenya (Ngumbau et al. 2020), Aberdares Ranges Forest (Kipkoech et al. 2020), and South and North Nandi forests (Girma et al. 2015; Melly et al. 2020) have been documented.

This study aims to provide a broader description of the updated vascular plants of Taita Hills through a comprehensive species inventory. Our objectives were to document the vascular plants occurring in the entire Taita Hills area, to determine the range restricted taxa as well as the species of conservation concern and record the different life forms and habitats within the Taita Hills. The checklist will assist policy-makers to make more informed decisions and appropriate strategies in the conservation and management of biodiversity in the area, especially for the range-restricted taxa. It also provides a basis for further botanical studies in Taita Hills.

## ﻿Materials and methods

### ﻿Study area

**Figure 1.**
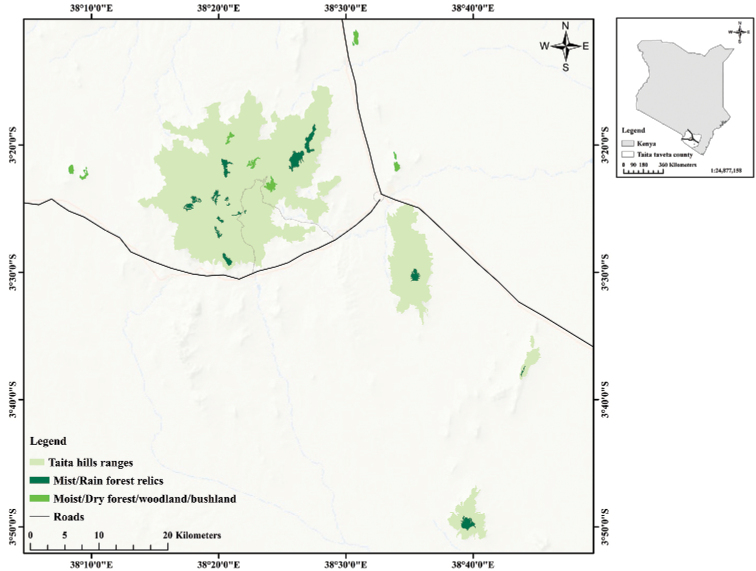
Location of the main Taita Hills blocks and adjacent hills showing the distribution of the forest patches.

The Taita Hills are located in the southeast part of Kenya within Taita Taveta county, forming the northernmost tip of the Eastern Arc Mountains. These ranges are divided into the Dabida, Mbololo, Sagala, and Kasigau massifs. Dabida and Mbololo are nearly intact, separated by a valley at 900 m a.s.l., while Sagala and Kasigau are isolated, 20 km and 50 km respectively, to the south (Pellikka et al. 2013; Medley and Maingi 2014). Adjacent to these main blocks are smaller hills, namely Maungu, Ndi, Maktau, and Mzinga, which have been included in this study (Fig. 1). This region lies between 38°08'E; 03°15'S and 38°45'E; 03°51'S, west of Voi town located along the Nairobi-Mombasa highway (A 109).

The elevation of the surrounding plains ranges from 480–700 m a.s.l. (Medley and Kalibo 2005; Medley et al. 2010), from which there are steep rises within short distances forming a series of ridges peaking at 2228 m a.s.l. on Vuria summit (Beentje 1988; Thijis et al. 2013). The steepness of the rocks is due to the hardness of a quartzite cap overlaying soft metamorphic rocks (Beentje 1988; Conte 2010).

### ﻿Forest fragments

The once intact montane forest on these hills has been reduced to 52 forest patches comprised of indigenous, mixed, and plantation forest vegetation (CEPF 2005). Administration of these forests is divided between two institutions, the Kenya Forest Service (KFS) overseeing 25 forest patches covering an area of 1310.41 ha, and the Taita Taveta County Council which oversees 24 forest patches (excluding Kitobo 160 ha, Salaita 40.5 ha, and Lotima 40.5 ha, which lie outside the scope of this study area) covering 7350.6 ha (CEPF 2005; Mwang’ombe 2005). Out of these, only 14 are indigenous relics varying greatly in size with the largest being Mbololo (220 ha) and the smallest Kituchenyi (<1 ha) (CEPF 2005; Thijis et al. 2013; Medley and Maingi 2014). Only three indigenous forests (Mbololo juu, Mwambirwa, and Ngangao) have been surveyed, leaving 11 forests lacking clear boundaries (Beentje 1988; CEPF 2005).

### ﻿Climate

Due to their isolation, these hills form the first large barrier from the coastal strip trapping moisture-laden clouds. This moisture results in precipitation in the hilltop forests creating near permanent humid conditions all year round in areas with altitudes above 1400 m on the south-eastern slopes and 1700 m on the much drier north-western slopes (Pellikka et al. 2009). The climate of the area is under the influence of the Inter-Tropical Convergence Zone creating two distinct rainy seasons. Long rains occur from March to June while short rains are experienced from October to December. The annual average rainfall varies from 500 mm on the foothills to 1910 mm in the hills (Beentje 1988; Salminen 2004; CEPF 2005). The mean annual temperature ranges from 16–18 °C in the hills and as high as 24.6 °C in the lower zones (Omoro et al. 2013).

### ﻿Vegetation

Vegetation structure within the hills varies along the elevation gradient with the steep slopes providing very short transitional bands from one vegetational type to the other. Though there is a lack of continuous indigenous vegetative cover due to a matrix of forests, settlements, and farms, there are distinct changes in vegetation structure and phenology due to temperature and moisture variations (Medley and Kalibo 2007). In areas below ca. 650 m a.s.l., *Acacia*-*Commiphora* bushland vegetation forms a wildlife corridor in the plains between the Tsavo East and Tsavo West national parks around and on the foothills (Medley et al. 2017). Along the slopes, forest fragments and deciduous/evergreen montane woodland vegetation are nested within an agricultural rural landscape (Newmark 1998).

In the much drier north-western slopes, vegetation mostly comprises *Euphorbiaingens* E.Mey. ex Boiss. and Euphorbiabusseivar.kibweziensis (N.E.Br.) S.Carter (Pellikka et al. 2013). On the hilltops, lies the isolated evergreen forest described as upland mist forest (Beentje 1988), Afromontane rainforest (Bytebier 2001; Aerts et al. 2011), or cloud forests (Pellikka et al. 2009; Medley and Maingi 2014). The potential natural vegetation community here is *Ocotea*-*Podocarpus*. However, these two species have undergone intensive selective harvesting and only a small number of mature individuals remain (Bytebier 2001).

Disturbance indicator species such as *Tabernaemontanastapfiana* Britten, *Albiziagummifera* (J.F.Gmel.) C.A.Sm., *Maesalanceolata* Forssk., and *Phoenixreclinata* Jacq., are more common in these forest patches (Chege and Bytebier 2005; Omoro and Luukkanen 2011). Canopy species include *Aningeriaadolfi-friedericii* (Engl.) Robyns & Gilbert, *Albiziagummifera*, *Polysciasfulva* (Hiern) Harms, *Ocotea* sp., *Macarangaconglomerata* Brenan, and *Newtoniabuchanannii* (Baker) G.C.C.Gilbert & Boutique while understory species include *Psychotria* sp., *Dracaenasteudneri* Engl., *Rytigyniauhligii* (K.Schum. & K.Krause) Verdc., *Strombosiascheffleri* Engl., *Pauridianthapaucinervis* (Hiern) Bremek. (Chege and Bytebier 2005; Thijis et al. 2013). Plantation forests of *Pinuspatula* Schiede ex Schltdl. & Cham., *Hesperocyparislusitanica* (Mill.) Bartel, *Eucalyptussaligna* Sm, and *Juniperus* sp. exist within some of the indigenous fragments and in areas where denuded hilltops were reforested with exotic tree species (Beentje 1988; Omoro et al. 2013). Common invasive species found within Taita Hills include *Maesopsiseminii* Engl., *Cinnamomumcamphora* (L.) J.Presl, *Lantanacamara* L., *Biancaedecapetala* (Roth) O.Deg., and *Acaciamearnsii* De Wild. (Spanhove and Lehouck 2008).

### ﻿Land use

Increased demand for fertile land in the area due to a rise in human population over the past 250 years has led to the conversion of indigenous forests to agricultural land (Jaetzold et al. 2010; CEPF 2005). The bulk of farmlands are located in areas that have high agricultural potential or are forested between 800–1700 m (Bytebier 2001), with some farms extending to as low as 480 m in Kasigau (Medley and Kalibo 2005). The main cultivated crops are coffee, mangoes, cassava, banana, maize, and beans (Beentje 1988; Jaetzold et al. 2010). As a means to safeguard against crop failure due to changes in weather patterns, families own farmlands at different elevations and thus leading to more forest loss and fragmentation (Bytebier 2001; Madoffe et al. 2005).

### ﻿Floristic survey, plant collection and nomenclature

Several field expeditions were carried out between the years 2015 and 2019 by the Sino-Africa Joint Research Center (SAJOREC) and Flora of Kenya Project (FOKP). The various forest fragments were surveyed in both the wet and dry seasons with an effort put to expand geographical coverage to areas where previous collections had been scarce. General walk-over surveys were carried out targeting fertile plant species which were thereafter photographed, tagged, and collected. The species habit, habitat, elevation, location, and collector’s details were recorded. The specimens were then pressed, dried, and deposited at the East African herbarium (EA) with duplicates deposited at the Hubei Institute of Botany herbarium (HIB). Identification of collected specimens was done using existing floral monographs (FTEA 1952–2012; Beentje 1994; Agnew 2013), as well as comparing collections with identified specimens in the EA.

Previous collections recorded from the Taita Hills were collated to supplement records from the field surveys. These were obtained from specimens in the EA, monographs, and published literature (FTEA 1952–2012; Beentje 1988, 1994; Medley and Kalibo 2007; Thijis et al. 2013), Global Biodiversity Information Facility (GBIF 2021) (http://www.gbif.org) and the Integrated Digitized Biocollections (iDigBio 2021) (http://www.idigbio.org).

The conservation status of all vascular plants recorded was determined using the International Union for Conservation of Nature criterion (IUCN 2021) (https://www.iucnredlist.org). Existing range-restricted taxa were determined by searching all recorded species in the GBIF occurrence database (www.gbif.org), IUCN Red List (IUCN 2021) (https://www.iucnredlist.org) and the Flora of Tropical East Africa (FTEA 1952–2012). The current taxonomic circumscription, as well as the authorities for each taxon were determined through the Plants of the World Online (POWO 2019) (http://www.plantsoftheworldonline.org), African Plant Database (http://africanplantdatabase.ch) (African Plant Database 2021), Tropicos (http://www.tropicos.org) (Tropicos 2021) and the Catalogue of Life (Roskov et al. 2021) (http://www.catalogueoflife.org).

## ﻿Results

### ﻿Species diversity

**Figure 2.**
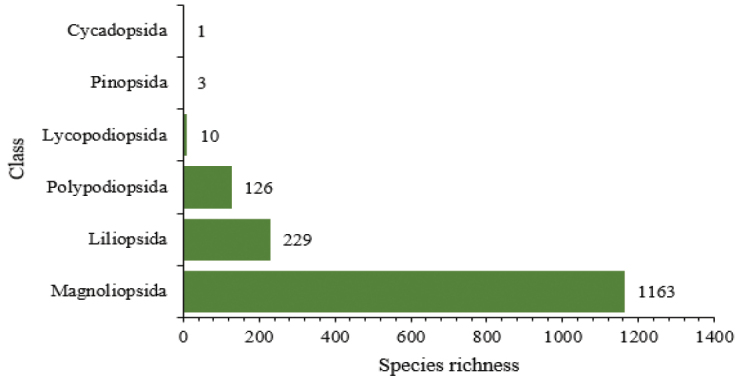
The classes of vascular plant species in Taita Hills.

We recorded 1594 taxa (1530 species, 39 subspecies, 27 varieties, and two hybrids) representing 709 genera and 159 families of vascular plants within the Taita Hills. These constitute 60.89%, 42.78%, and 23.41% of all indigenous families, genera and species, and 35.48%, 16.89%, and 10.03% of all exotic families, genera and species represented in the flora of Kenya respectively (FTEA 1952–2012). Eighty-three, 17, 58 and 59 taxa represent threatened, endemic, near-endemic and exotic species in Taita Hills respectively. Diversity within the classes in sequence from the most to the least diverse was Magnoliopsida (75.91%), Liliopsida (14.95%), Polypodiopsida (8.22%), Lycopodiopsida (0.65%), Pinopsida (0.2%), and Cycadopsida (0.07%) (Fig. 2).

The most speciose families are Fabaceae, 133 taxa (125 species), Rubiaceae, 88 (75), Asteraceae, 78 (77), and, Orchidaceae, 70 (69). Genera with 15 or more species include *Euphorbia* (Euphorbiaceae), *Asplenium* (Aspleniaceae), *Cyperus* (Cyperaceae), *Coleus* (Lamiaceae) and, *Ceropegia* (Apocynaceae) (Table 1).

**Table 1.** The largest families and genera of vascular plants in Taita Hills.

Family	Genera	Species	Genus	Species
Fabaceae	55	125	*Euphorbia* L. (Euphorbiaceae)	34
Asteraceae	46	77	*Asplenium* L. (Aspleniaceae)	31
Rubiaceae	41	75	*Cyperus* L. (Cyperaceae)	30
Orchidaceae	28	69	*Coleus* Lour. (Lamiaceae)	21
Apocynaceae	34	64	*Ceropegia* L. (Apocynaceae)	15
Malvaceae	21	58	*Commiphora* Jacq. (Burseraceae)	15
Euphorbiaceae	13	55	*Crotalaria* L. (Fabaceae)	15
Lamiaceae	20	50	*Solanum* L. (Solanaceae)	15
Poaceae	25	47	*Ipomoea* L. (Convolvulaceae)	14
Aspleniaceae	44	44	*Ficus* Tourn. ex L. (Moraceae)	14
Cyperaceae	9	42	*Polystachya* Hook. (Orchidaceae)	13

### ﻿Plant growth forms

**Figure 3.**
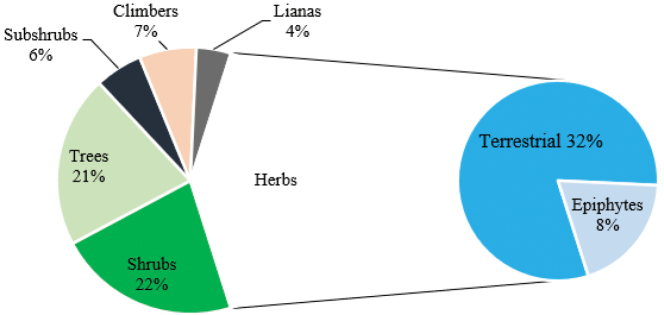
Growth form of vascular plants in Taita Hills.

The growth habit(s) of each species was recorded using a simple classification into six categories: trees, shrubs, lianas, subshrubs, climbers and herbs. Species that express variations in their growth habit are recorded in two or more categories. Herbs represented the highest percentage of life forms with 711 taxa (573 terrestrial and 138 epiphytic), followed by shrubs with 391 taxa, trees with 369 taxa, climbers with 123 taxa, subshrubs with 101 taxa, and lianas with 74 taxa. (Fig. 3).

### ﻿Plant species global conservation status

According to the IUCN Red List of Threatened Species (IUCN 2021), a total of 484 of the plants found in Taita Hills were evaluated. Of these taxa, 63 are threatened while 20 are near threatened. Two taxa are recorded as Critically Endangered (CR), 27 as Endangered (EN), 34 as Vulnerable (VU), 20 as Near Threatened (NT), 399 as of Least Concern (LC) and two taxa as Data Deficient (DD) (Table 2, 3). The families with the most numbers of species in need of conservation concern are Rubiaceae (17 taxa), Orchidaceae (eight taxa) and Euphorbiaceae (six taxa). In terms of life forms, trees were the highest in number forming 35% of the total. They were followed by herbs 32%, shrubs 29%, subshrubs 2%, climbers and lianas 1% each (Table 7).

**Table 2.** Number of plant taxa and their proportion in each IUCN category.

IUCN Category	No. of taxa	Percentage
Critically Endangered (CR)	2	0.13%
Endangered (EN)	27	1.70%
Vulnerable (VU)	34	2.13%
Near Threatened (NT)	20	1.25%
Least Concern (LC)	399	25.03%
Data Deficient (DD)	2	0.13%
Not Evaluated (N/A)	1110	69.63%
**Total**	**1594**	**100**%

**Table 3.** List of threatened and near-threatened plant taxa found in Taita Hills, their IUCN category and current status.

Family	Species	Life form	Category	Status	Version
Acanthaceae	*Crossandrafriesiorum* Mildbr.	Herb	VU	Decreasing	3.1
Acanthaceae	Dyschoristekeniensissubsp.glandulifera Malombe, Mwachala & Vollesen	Subshrub	VU	Decreasing	3.1
Acanthaceae	*Isoglossacandelabrum* Lindau	Shrub	EN	Unknown	3.1
Acanthaceae	*Justiciagaleata* Hedrén	Shrub	VU	Decreasing	3.1
Acanthaceae	Neuracanthustephrophyllussubsp.tsavoensis Bidgood & Brummitt	Subshrub	EN	Decreasing	3.1
Araliaceae	*Astropanaxmyrianthus* (Baker) Lowry, G.M.Plunkett & al.	Liana/Tree	NT	Decreasing	3.1
Araliaceae	*Polysciasstuhlmannii* Harms	Tree	EN	Unknown	3.1
Asphodelaceae	*Aloeballyi* Reynolds	Tree	EN	Decreasing	3.1
Asphodelaceae	*Aloeclassenii* Reynolds	Herb	CR	Decreasing	3.1
Asphodelaceae	*Aloedeserti* A.Berger	Shrub	NT	Unknown	3.1
Asphodelaceae	*Aloependuliflora* Baker	Shrub	EN	Unknown	3.1
Asphodelaceae	AloevolkensiiEngl.subsp.volkensii	Shrub	NT	Decreasing	3.1
Asteraceae	*Bothrioclineglomerata* (O.Hoffm. & Muschl.) C.Jeffrey	Shrub	EN	Unknown	3.1
Asteraceae	*Brachylaenahuillensis* O.Hoffm.	Shrub/Tree	NT	Unspecified	2.3
Asteraceae	*Solaneciomirabilis* (Muschl.) C.Jeffrey	Herb	EN	Decreasing	3.1
Aspleniaceae	*Thelypterisusambarensis* (Holttum) Christenh.	Herb	EN	Unknown	3.1
Burmanniaceae	*Gymnosiphonusambaricus* Engl.	Herb	EN	Unknown	3.1
Burseraceae	*Commiphoracampestris* Engl.	Tree	NT	Unknown	2.3
Burseraceae	*Commiphoraoblongifolia* J.B.Gillett	Shrub/Tree	NT	Unknown	3.1
Cucurbitaceae	*Gerrardanthusgrandiflorus* Gilg ex Cogn.	Climber	NT	Decreasing	3.1
Cyperaceae	Bulbostylishispidulasubsp.halophila (Lye) R.W.Haines	Herb	EN	Unknown	3.1
Cyperaceae	*Cyperuspurpureoviridis* Lye	Herb	NT	Stable	3.1
Dennstaedtiaceae	*Blotiellahieronymi* (Kümmerle) Pic. Serm.	Herb	EN	Unknown	3.1
Euphorbiaceae	EuphorbiabusseiPaxvar.bussei	Shrub	EN	Unknown	3.1
Euphorbiaceae	*Euphorbiafurcata* N.E.Br.	Herb	VU	Unknown	3.1
Euphorbiaceae	*Euphorbiapetricola* P.R.O.Bally & S.Carter	Herb	EN	Unknown	3.1
Euphorbiaceae	EuphorbiatenuispinosaGillivar.tenuispinosa	Herb	VU	Unknown	3.1
Euphorbiaceae	*Macarangaconglomerata* Brenan	Tree	VU	Decreasing	3.1
Euphorbiaceae	Milbraediacarpinifolia(Pax)Hutch.var.carpinifolia	Shrub	VU	Unspecified	2.3
Fabaceae	*Craibiabrevicaudata* (Vatke) Dunn	Tree	NT	Unspecified	2.3
Fabaceae	*Crotalariaukambensis* Vatke	Herb	EN	Unknown	3.1
Fabaceae	*Dalbergiamelanoxylon* Guill. & Perr.	Shrub/Tree	NT	Unspecified	2.3
Fabaceae	Millettiaoblatasubsp.teitensis J.B.Gillett	Tree	VU	Unspecified	2.3
Gesneriaceae	*Streptocarpusteitensis* (B.L.Burtt) Christenh.	Herb	CR	Decreasing	3.1
Gesneriaceae	*Streptocarpusmontanus* Oliv.	Herb	NT	Unknown	3.1
Iridaceae	*Gladiolususambarensis* Marais ex Goldblatt	Herb	NT	Unknown	3.1
Lamiaceae	*Coleustriangularis* (A.J.Paton) A.J.Paton	Herb	NT	Decreasing	3.1
Lamiaceae	Vitexfischerivar.keniensis (Turrill) Meerts	Tree	EN	Decreasing	3.1
Lauraceae	*Ocoteakenyensis* (Chiov.) Robyns & R.Wilczek	Tree	VU	Unspecified	2.3
Loranthaceae	*Englerinadrummondii* Balle ex Polhill & Wiens	Herb	VU	Decreasing	3.1
Lycopodiaceae	*Huperziaholstii* (Hieron.) Pic.Serm.	Herb	NT	Unknown	3.1
Malvaceae	*Hibiscusgreenwayi* Baker f.	Shrub	VU	Unknown	3.1
Melastomataceae	*Memecylonteitense* Wickens	Tree	VU	Unknown	3.1
Moraceae	*Dorsteniagoetzei* Engl.	Herb	NT	Decreasing	3.1
Moraceae	*Dorsteniawarneckei* Engl.	Herb	NT	Decreasing	3.1
Moraceae	*Miliciaexcelsa* (Welw.) C.C.Berg	Tree	NT	Unspecified	2.3
Myrtaceae	*Eucalyptuscamaldulensis* Dehnh.	Tree	NT	Stable	3.1
Myrtaceae	*Syzygiummicklethwaitii* Verdc.	Tree	VU	Decreasing	3.1
Myrtaceae	*Syzygiumsubcordatum* (Verdc.) Byng &N.Snow	Tree	VU	Decreasing	3.1
Nymphaeaceae	Nymphaeanouchalivar.caerulea (Savigny) Verdc.	Herb	EN	Decreasing	3.1
Ochnaceae	*Campylospermumscheffleri* (Engl. & Gilg) Farron	Tree	VU	Unspecified	2.3
Orchidaceae	*Anselliaafricana* Lindl.	Herb	VU	Decreasing	3.1
Orchidaceae	Cynorkisbuchwaldianasubsp.braunii (Kraenzl.) Summerh.	Herb	NT	Decreasing	3.1
Orchidaceae	*Cynorkisuncata* (Rolfe) Kraenzl.	Herb	VU	Unknown	3.1
Orchidaceae	Polystachyacaespitificasubsp.latilabris (Summerh.) P.J Cribb & Podz.	Herb	VU	Unknown	3.1
Orchidaceae	*Polystachyadisiformis* P.J. Cribb	Herb	EN	Unknown	3.1
Orchidaceae	*Polystachyateitensis* P.J.Cribb	Herb	EN	Unknown	3.1
Orchidaceae	*Tridactylecruciformis* Summerh.	Herb	EN	Unknown	3.1
Orchidaceae	*Ypsilopustanneri* (P.J.Cribb) D’haijère & Stévart	Herb	EN	Unknown	3.1
Phyllanthaceae	*Meineckiaovata* (E.A.Bruce) J.F.Brunel ex Radcl.-Sm.	Shrub	VU	Unspecified	2.3
Podocarpaceae	*Afrocarpususambarensis* (Pilg.) C.N.Page	Tree	EN	Decreasing	3.1
Rosaceae	*Prunusafricana* (Hook.f.) Kalkman	Tree	VU	Unspecified	2.3
Rubiaceae	Canthiumoligocarpumsubsp.intermedium Bridson	Shrub/Tree	VU	Unspecified	2.3
Rubiaceae	*Coffeafadenii* Bridson	Tree	EN	Unknown	3.1
Rubiaceae	*Coffeazanguebariae* Lour.	Shrub	VU	Unknown	3.1
Rubiaceae	Empogonaovalifoliavar.glabrata (Oliv.) Tosh & Robbr.	Shrub	VU	Unspecified	2.3
Rubiaceae	Empogonaovalifoliavar.taylorii (S.Moore) Tosh & Robbr.	Shrub	VU	Unspecified	2.3
Rubiaceae	Oxyanthuspyriformissubsp.brevitubus Bridson	Shrub/Tree	VU	Unspecified	2.3
Rubiaceae	Oxyanthuspyriformissubsp.longitubus Bridson	Shrub/Tree	EN	Unspecified	2.3
Rubiaceae	PavettasepiumK.Schum.var.sepium	Shrub	VU	Unspecified	2.3
Rubiaceae	*Pavettateitana* K.Schum.	Shrub	VU	Decreasing	3.1
Rubiaceae	*Psychotriaalsophila* K.Schum.	Shrub	VU	Unspecified	2.3
Rubiaceae	*Psychotriacrassipetala* E.M.A. Petit	Tree	EN	Decreasing	3.1
Rubiaceae	*Psychotriacyathicalyx* E.M.A.Petit	Tree	VU	Unspecified	2.3
Rubiaceae	*Psychotriapseudoplatyphylla* E.M.A.Petit	Shrub	VU	Unspecified	2.3
Rubiaceae	*Psychotriapetitii* Verdc.	Tree	EN	Decreasing	3.1
Rubiaceae	*Psychotriapseudoplatyphylla* E.M.A.Petit	Shrub	VU	Unspecified	2.3
Rubiaceae	*Psychotriataitensis* Verdc.	Tree	EN	Unknown	3.1
Rubiaceae	*Psydraxpolhillii* Bridson	Shrub	VU	Decreasing	3.1
Rubiaceae	*Rytigyniaeickii* (K.Schum. & K.Krause) Bullock	Shrub	VU	Unspecified	2.3
Rutaceae	*Veprisfadenii* (Kokwaro) Mziray	Tree	VU	Decreasing	3.1
Salicaceae	*Biviniajalbertii* Tul.	Tree	NT	Unspecified	2.3
Zamiaceae	*Encephalartoskisambo* Faden & Beentje	Tree	EN	Decreasing	3.1

### ﻿Endemic and near-endemic species

Species with a geographic restricted range are treated in this checklist as either strict endemics (i.e. occurring only within Taita Hills forests) or as near endemics which are further subdivided into:

i. Eastern Arc endemics – Those taxa whose geographical scope of occurrence is limited to the Eastern Arc Mountain ranges.

ii. Taita-Taveta District endemics – Those taxa restricted to Taita Taveta district within the study area but not within the forest fragments.

iii. Regional Endemics – Those taxa that are extant in 10 or fewer locations, or are threatened and in decline according to the IUCN, or cover a small area of occupancy (<10, 000 km^2^) such that the population size in Taita Hills is significant for the survival of the species (Darbyshire et al. 2019).

**Figure 4.**
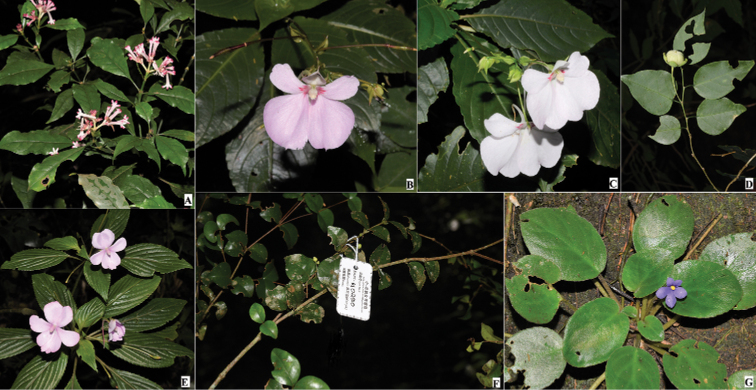
Some Taita Hills endemic and near endemic plant species **A**Chassaliadiscolorsubsp.taitensis**B-C**ImpatiensteitensisGrey-Wilsonsubsp.teitensis**D***Meineckiaovata***E**Impatiensenglerisubsp.pubescens**F***Memecylonteitense***G***Streptocarpusteitensis*.

This checklist contains 17 strict endemics and 58 near endemics (Fig. 4) (Tables 4, 5). These are distributed in 32 families and 55 genera. They represent 4.9% of the total species recorded in this area. The taxa-rich families are Rubiaceae (12 taxa), Orchidaceae (seven taxa), Euphorbiaceae, Apocynaceae (six taxa each) and Fabaceae (five taxa). In terms of life forms, herbs formed the bulk being 37% of the total, followed by shrubs 26%, trees 24%, climbers 8%, lianas 3% and subshrubs 2% (Table 7).

**Table 4.** Number of endemic and near-endemic species found in the Taita Hills.

Region	Number of species
Taita relicts	17
Eastern Arc Mountains (Excluding Taita Hills)	30
Taita district	2
Regional Endemics	26
**Total**	**75**

**Table 5.** Endemic and near-endemic plants of Taita Hills.

Family	Species	Life form	Distribution
Acanthaceae	Dyschoristekeniensissubsp.glandulifera Malombe, Mwachala & Vollesen	Subshrub	K7, T2, 3
Acanthaceae	*Isoglossacandelabrum* Lindau	Shrub	EAM
Apocynaceae	Ceropegiadistinctavar.rostrata Masinde	Climber	Taita District
Apocynaceae	*Ceropegiakonasita* Masinde	Climber	K7
Apocynaceae	*Ceropegiaverticillata* Masinde	Climber	Mbololo
Apocynaceae	*Huerniaandreaeana* (Rauh) L.C.Leach	Herb	K7
Apocynaceae	*Orbeataitica* Bruyns	Herb	K4, 7
Apocynaceae	*Tylophora* sp. B of FTEA	Climber	Ngangao
Asphodelaceae	*Aloeclassenii* Reynolds	Herb	K7
Asphodelaceae	*Aloependuliflora* Baker	Shrub	K7
Asteraceae	*Bothrioclineglomerata* (O.Hoffm. & Muschl.) C.Jeffrey	Shrub	K4, 7, T3
Asteraceae	*Solaneciobuchwaldii* (O.Hoffm.) C.Jeffrey	Shrub	EAM
Balsaminaceae	Impatiensenglerisubsp.pubescens Grey-Wilson	Herb	Ngangao
Balsaminaceae	*Impatiensteitensis* Grey-Wilson	Herb	Taita Hills
Burmanniaceae	*Gymnosiphonusambaricus* Engl.	Herb	EAM
Cucurbitaceae	*Gerrardanthusgrandiflorus* Cogn.	Climber	K7, T3
Cucurbitaceae	*Zehneriatuberifera* G.W.Hu & Q.F.Wang	Climber	Ngangao
Cyperaceae	*Cyperusscott-elliotii* Govaerts	Herb	Taita District
Dennstaedtiaceae	*Blotiellahieronymi* (Kümmerle) Pic.Serm.	Herb	EAM
Dichapetalaceae	*Dichapetalumeickii* Ruhland	Shrub/Liana	EAM
Euphorbiaceae	*Euphorbiaclassenii* P.R.O.Bally & S.Carter	Shrub	Kasigau
Euphorbiaceae	*Euphorbiafurcata* N.E.Br.	Herb	K7, T2, 3
Euphorbiaceae	*Euphorbianeoglaucescens* Bruyns	Shrub/Tree	K7, T3, 5, 6
Euphorbiaceae	*Euphorbiapetricola* P.R.O.Bally & S.Carter	Herb	K7
Euphorbiaceae	*Jatrophavelutina* Pax & K.Hoffm.	Subshrub	K7
Euphorbiaceae	*Macarangaconglomerata* Brenan	Tree	EAM
Fabaceae	*Crotalarialukwangulensis* Harms	Shrub/Liana	EAM
Fabaceae	*Cynometra* sp. A of FTEA	Tree	EAM
Fabaceae	MillettiaoblataDunnsubsp.burttii J.B.Gillett	Shrub/Tree	K7, T5
Fabaceae	Millettiaoblatasubsp.teitensis J.B.Gillett	Tree	EAM
Gesneriaceae	*Streptocarpuskirkii* Hook.f.	Herb	EAM
Gesneriaceae	*Streptocarpussaxorum* Engl.	Herb	EAM
Gesneriaceae	*Streptocarpusteitensis* (B.L.Burtt) Christenh.	Herb	Mbololo
Hymenophyllaceae	*Hymenophyllum* sp. A of FTEA	Herb	EAM
Iridaceae	*Gladiolusrupicola* Vaupel	Herb	EAM
Iridaceae	*Gladiolususambarensis* Marais ex Goldblatt	Herb	K7, T2, 3, 6
Lamiaceae	*Coleustriangularis* (A.J.Paton) A.J.Paton	Herb	EAM
Lamiaceae	Leucasoligocephalavar.usambarica Sebald	Herb	EAM
Linderniaceae	*Linderniellabrevidens* (Skan) Eb.Fisch., Schäferh. & Kai Müll.	Herb	EAM
Malphigiaceae	*Acridocarpustaitensis* Mwadime, Ngumbau & Q.Luke	Liana	Ngangao
Malvaceae	Abutilonmauritianumvar.grandiflorum Verdc.	Shrub	EAM
Melastomataceae	*Memecylongreenwayi* Brenan	Tree	EAM
Melastomataceae	*Memecylonteitense* Wickens	Shrub/Tree	Ngangao/Mbololo
Moraceae	*Dorsteniachristenhuszii* M.W.Chase & M.F.Fay	Herb	Mbololo
Moraceae	*Dorsteniawarneckei* Engl.	Herb	K7, T3, 6
Myrtaceae	*Syzygiummicklethwaitii* Verdc.	Tree	K7, T2, 3, 6
Myrtaceae	*Syzygiumsubcordatum* (Verdc.) Byng & N.Snow	Tree	EAM
Ochnaceae	*Campylospermumscheffleri* (Engl. & Gilg) Farron	Tree	EAM
Orchidaceae	Cynorkisbuchwaldianasubsp.braunii (Kraenzl.) Summerh.	Herb	EAM
Orchidaceae	*Cynorkisuncata* (Rolfe) Kraenzl.	Herb	EAM
Orchidaceae	*Microcoeliasmithii* (Rolfe) Summerh.	Herb	K7, T3, 6
Orchidaceae	*Polystachyateitensis* P.J.Cribb	Herb	Maungu/Kasigau
Orchidaceae	*Rhipidoglossumleedalii* (P.J.Cribb) Farminhão & Stévart	Herb	EAM
Orchidaceae	*Tridactylecruciformis* Summerh.	Herb	K6, 7, T3
Orchidaceae	*Ypsilopustanneri* (P.J.Cribb) D’haijère & Stévart	Herb	EAM
Orobanchaceae	*Harveyakenyensis* Hepper	Herb	K7, 4
Phyllanthaceae	*Meineckiaovata* (E.A.Bruce) J.F. Brunel ex Radcl.-Sm.	Shrub	Ngangao
Phyllanthaceae	*Phyllanthusmittenianus* Hutch.	Shrub	K7
Rubiaceae	Chassaliadiscolorsubsp.taitensis Verdc.	Shrub	Taita Hills
Rubiaceae	*Coffeafadenii* Bridson	Tree	EAM
Rubiaceae	*Galiumbrenanii* Ehrend. & Verdc.	Herb	K7, T2, 3, 6
Rubiaceae	Oxyanthuspyriformissubsp.longitubus Bridson	Shrub/Tree	K7
Rubiaceae	*Parapentasbattiscombei* Verdc.	Herb	Ngangao, K4
Rubiaceae	*Psychotriaalsophila* K.Schum.	Shrub/Tree	Ngangao, T2, 3
Rubiaceae	*Psychotriacrassipetala* E.M.A.Petit	Shrub/Tree	K7
Rubiaceae	*Psychotriapetitii* Verdc.	Shrub/Tree	Ngangao/Mbololo
Rubiaceae	*Psychotriapseudoplatyphylla* E.M.A.Petit	Shrub/Tree	Taita, T2, 3
Rubiaceae	*Psychotria* sp. B of FTEA	Shrub	Ngangao
Rubiaceae	*Psychotriataitensis* Verdc.	Tree	Kasigau
Rubiaceae	*Rytigyniaeickii* (K.Schum. & K.Krause) Bullock	Shrub/Tree	EAM
Rutaceae	*Veprisfadenii* (Kokwaro) Mziray	Shrub/Tree	Taita Hills
Thelypteridaceae	*Thelypterisusambarensis* (Holttum) Christenh.	Herb	EAM
Thymelaeaceae	*Dicranolepisusambarica* Gilg	Shrub/Tree	EAM
Vitaceae	*Cyphostemmabraunii* (Gilg & M.Brandt) Desc.	Climber	EAM
Zamiaceae	*Encephalartoskisambo* Faden & Beentje	Tree	EAM

**Table 6.** List of exotic plant species recorded in Taita Hills.

Family	Species	Habit	Status
Amaranthaceae	*Alternantherapungens* Kunth	Herb	Introduced
Amaranthaceae	*Alternantherasessilis* (L.) R.Br. ex DC.	Herb	Imtroduced
Amaranthaceae	*Amaranthusgraecizans subsp. thellungianus* (Nevski) Gusev	Herb	Introduced
Amaranthaceae	*Amaranthushybridus* L. subsp. *Hybridus*	Herb	Introduced
Amaranthaceae	*Gomphrenacelosioides* Mart.	Herb	Naturalized-Introduced
Amaranthaceae	*Guillemineadensa* (Willd. ex Schult.) Moq.	Herb	Introduced
Annonaceae	*Annonacherimola* Mill.	Shrub	Cultivated
Apiaceae	*Cyclospermumleptophyllum* (Pers.) Sprague ex Britton & P.Wilson	Herb	Naturalized-Introduced
Apocynaceae	*Calotropisgigantea* (L.) W.T.Aiton	Shrub	Naturalized-Introduced
Apocynaceae	*Catharanthusroseus* (L.) G.Don	Tree	Cultivated-Naturalized
Apocynaceae	*Gomphocarpusphysocarpus* E.Mey.	Herb	Introduced
Asteraceae	*Acmellauliginosa (Sw.) Cass.*	Herb	Introduced
Asteraceae	*Erigeronkarvinskianus* DC.	Herb	Naturalized-Introduced
Asteraceae	*Flaveriatrinervia* (Spreng.) C.Mohr	Herb	Naturalized-Introduced
Asteraceae	*Galinsogaparviflora* Cav.	Herb	Naturalized-Introduced
Asteraceae	*Seneciovulgaris* L.	Herb	Introduced
Asteraceae	*Tagetesminuta* L.	Herb	Naturalized-Introduced
Asteraceae	*Tithoniadiversifolia* (Hemsl.) A.Gray	Subshrub	Naturalized-Introduced
Asteraceae	*Tridaxprocumbens* L.	Herb	Naturalized-Introduced
Brassicaceae	*Brassicarapa* L.	Herb	Naturalized-Introduced
Brassicaceae	*Lepidiumbonariense* L.	Herb	Naturalized-Introduced
Cactaceae	*Opuntiahumifusa* (Raf.) Raf.	Shrub	Introduced
Crassulaceae	*Kalanchoeminiata* Hilsenb. & Bojer ex Tul.	Herb	Cultivated
Cupressaceae	*Callitrispreissii* Miq.	Tree	Introduced-Cultivated
Euphorbiaceae	*Euphorbiaserpens* Kunth	Herb	Naturalized-Introduced
Euphorbiaceae	*Jatrophacurcas* L.	Shrub/Tree	Naturalized-Introduced
Euphorbiaceae	*Ricinuscommunis* L.	Herb	Naturalized-Cultivated
Fabaceae	*Acaciamearnsii* De Wild.	Tree	Naturalized-Introduced
Fabaceae	*Acaciamelanoxylon* R.Br.	Tree	Naturalized-Introduced
Fabaceae	*Biancaeaedecapetala* (Roth) O.Deg.	Shrub	Cultivated
Fabaceae	*Calliandrahoustoniana* (Mill.) Standl.	Shrub	Naturalized-Introduced
Fabaceae	*Indigoferatinctoria* L. *var. tinctoria*	Subshrub	Introduced
Fabaceae	*Leucaenaleucocephala* (Lam.) de Wit	Tree	Cultivated
Fabaceae	*Sennabicapsularis (L.) Roxb.*	Shrub	Introduced
Fabaceae	*Sennaoccidentalis* (L.) Link	Subshrub	Naturalized-Introduced
Lamiaceae	*Mesosphaerumpectinatum* (L.) Kuntze	Herb	Naturalized-Introduced
Lauraceae	*Cinnamomumcamphora* (L.) J.Presl	Tree	Naturalized-Introduced
Malvaceae	*Callianthemegapotamica* (A.Spreng.) Dorr	Shrub	Introduced-Cultivated
Malvaceae	*Hibiscuslunariifolius* Willd.	Herb	Introduced
Malvaceae	*Malvaverticillata* L.	Herb	Introduced
Meliaceae	*Meliaazedarach* L.	Tree	Introduced
Myrtaceae	*Eucalyptuscamaldulensis* Dehnh.	Tree	Cultivated-Naturalized
Myrtaceae	*Psidiumcattleianum* Sabine	Tree	Cultivated
Myrtaceae	*Psidiumguajava* L.	Tree	Naturalized-Introduced
Myrtaceae	*Syzygiumjambos* (L.) Alston	Tree	Cultivated
Passifloraceae	*Passifloraedulis* Sims	Climber	Cultivated-Naturalized
Poaceae	*Coixlacryma-jobi* L.	Herb	Introduced
Poaceae	*Setariasulcata* Raddi	Herb	Naturalized-Introduced
Proteaceae	*Grevillearobusta* A. Cunn. ex R.Br.	Tree	Naturalized-Introduced
Rosaceae	*Rubusniveus* Thunb.	Shrub	Naturalized-Introduced
Rosaceae	*Rubusrosifolius* Sm.	Shrub	Naturalized-Introduced
Solanaceae	*Nicandraphysalodes* (L.) Gaertn.	Herb	Cultivated
Solanaceae	*Nicotianatabacum* L.	Herb	Cultivated-Naturalized
Solanaceae	*Physalisperuviana* L.	Herb	Naturalized-Introduced
Solanaceae	*Solanumaculeatissimum* Jacq.	Shrub	Introduced
Solanaceae	*Solanummauritianum* Scop.	Shrub/Tree	Naturalized-Introduced
Solanaceae	*Solanumseaforthianum* Andrews	Climber	Naturalized-Introduced
Verbanaceae	*Lantanacamara* L.	Shrub	Naturalized
Verbanaceae	*Stachytarphetaurticifolia* Sims	Subshrub	Naturalized-Introduced

**Table 7.** Life form representatives of different categories of vascular plant species in Taita Hills.

**Life forms**	**Number of vascular plant species**		
	**Endemic and near-endemic**	**Threatened and near-threatened**	**Exotic**
Trees	22	31	13
Shrubs	26	25	12
Lianas	3	1	–
Subshrubs	2	2	4
Herbs	31	29	28
Climbers	7	1	2

**Figure 5.**
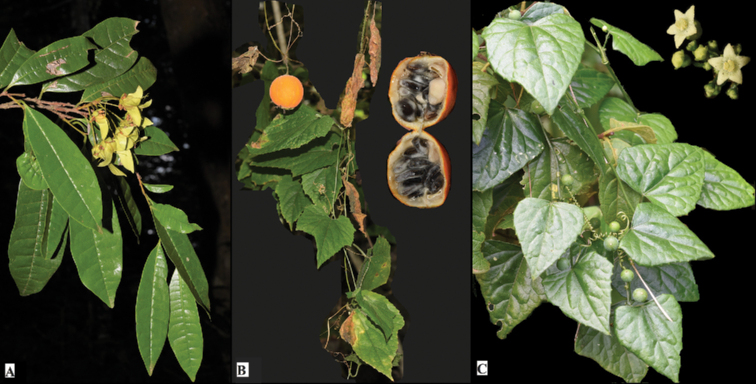
Photographs of new plant species recorded from Taita Hills **A***Acridocarpustaitensis***B***Momordicacalantha***C***Zehneriatuberifera*.

## ﻿Discussion and conclusion

The findings of this study validate Taita Hills as a floristically important area with a high diversity of plants as well as rare and endemic plant species. Its unique geographical position near the coastal strip and within an arid and semi-arid landscape provides for multiple habitats which subsequently support a wide range of taxa. The 1594 total taxa recorded represent 25.33% of all vascular plants found in Kenya (Zhou et al. 2017). This area also has a significant portion of lycophytes and pteridophytes representing 8.72% of all total records in this study.

The most species-rich families are Fabaceae, Rubiaceae, Asteraceae, Orchidaceae, Apocynaceae, Malvaceae and Euphorbiaceae. These results concur with similar studies conducted in other tropical rainforests such as the Coastal forests of Kenya (Ngumbau et al. 2020) and the Nandi forests (Melly et al. 2020), as well as other forests in the Afromontane archipelago of East Africa such as the Aberdares forests (Kipkoech et al. 2020), Agama forest (Addi et al. 2016), Afromontane forests of Ethiopia (Senbeta et al. 2014), and the Jibat Afromontane forest (Burju et al. 2013). Euphorbiaceae, Orchidaceae, and Rubiaceae are the most important families in Taita Hills in terms of species richness, endemism, and conservation status as all of them have substantial numbers in all three categories. Herbs were the most prevalent growth forms recorded in Taita Hills, especially in the forest habitats in both the understory and margins. However, woody plant life forms also formed a considerable amount of the total percentage of recorded plant life forms supporting the observation by Senbeta et al. (2014) that moist evergreen Afromontane forests sustain a high density of woody plants. Exotic species recorded in this study represent 3.85% of the total number of species. Solanaceae, Asteraceae, and Fabaceae have the highest number of exotic species. This concurs with the study done by Ngumbau et al. (2020) on the coastal forests of Kenya. A large number of these exotic species have colonized natural habitats and have also been listed as invasive (Spanhove and Lehouck 2008; Thijis et al. 2013). Mitigating the introduction of exotic tree species and levels of disturbance within indigenous forests might increase the recruitment of indigenous tree seedlings and subsequently the restoration of the original native forest status (Omoro and Luukkanen 2011).

The hilltop mist forests have been of great botanical appeal owing to their unique flora with Afromontane and rainforest elements (Beentje 1988, Thijis et al. 2013). The discovery of a new species and a new record within the rainforest fragments show that these forests are a biodiversity reservoir with more potential for scientific exploration. However, the high human population within the Taita-Taveta district resides within the hills and the demand for more agricultural and settlement land has put additional anthropogenic pressures on the already severely fragmented landscape (Bytebier 2001; CEPF 2005). Threatened and near-threatened taxa in Taita Hills according to IUCN (2021) represent 4.11% and 1.31% of the total taxa recorded. Although these figures might be small compared to the total, the probable loss of the already small habitats puts these species at a higher extinction risk on a local scale. A high percentage of species from Taita Hills have not been assessed (69.63%). Out of those that are assessed, most have been evaluated to be of least concern and thus are not facing imminent risk. Nonetheless, some of the species, though evaluated to be of LC, are also indicated as decreasing in the wild, thus presenting a potential risk to such, in the absence of strict conservation measures. In some cases, the global assessment might not reflect the real picture in specific local sites. The populations of species such *Kuloausambarensis* (Engl.) Trofimov & Rohwer and *Podocarpuslatifolius* (Thunb.) R.Br. ex Mirb., both evaluated as LC, and *Mitragynarubrostipulata* (K.Schum.) Havil., whose status has not been evaluated according to IUCN, have decreased to critical levels due to selective harvesting (Beentje 1988; Thijis et al. 2013). Ngangao forest fragment was recorded to have the highest number of range restricted taxa while many of the lesser fragments were recorded to have single endemics. This poses a great risk as there have been indications of fragmentation leading to the loss of endemic species. There is a need for a localized Red List for the forests to determine the level of species threats.

The Taita Hills have been part of the local community’s culture and the people have utilized these forests for many years. More community-based conservation programs should be introduced to promote the sustainable utilization of renewable forest resources while promoting regeneration (Himberg 2004). The regeneration of indigenous forests will not only improve the quality of habitat for endemic plants but also increase habitable ecosystems for range-restricted birds within the different fragments (Mwang’ombe 2005). Previously restored indigenous forests have exhibited a high abundance in avifaunal species compared to exotic forests. Restorations especially between larger fragments can act as migration corridors for endemic bird species and subsequently increase indigenous vegetation cover while mitigating the extinction risk of range restricted species (Borghesio et al. 2017). In addition to an increase in natural forest cover, native tree regeneration will protect the plant diversity within the fragments as studies in Taita Hills have shown that indigenous forests tend to have a higher plant diversity compared to exotic forests (Omoro et al. 2010). The comprehensive listing of endemic and threatened species in this study will serve as a guideline to enable policy-makers to make more informed and appropriate decisions beneficial for the conservation and management of biodiversity in the area. It also sets a foundation for other floristic studies in these forests and updates the plant biodiversity records of the Taita hills ranges.

## ﻿Checklist

This checklist records comprehensively the vascular plants of the Taita Hills in their respective families which have been listed alphabetically. The families are grouped into six classes namely Lycopodiopsida, Polypodiopsida, Cycadopsida, Pinopsida, Liliopsida and Magnoliopsida. Taxa of Lycopodiopsida and Polypodiopsida are classified based on the Pteridophyte Phylogeny Group I system (PPG 1 2016), Pinopsida and Cycadopsida according to Christenhusz et al. (2011), while Liliopsida and Magnoliopsida are classified, based on the APG IV system (Chase et al. 2016). For each species recorded, information on its life form, habitat, altitudinal range of occurrence (in meters) and voucher number(s) is listed. Species names that are succeeded with a letter, (e.g. A), indicate that they have not been described yet. Those with a genus name succeeded by “sp” indicate that they were listed as such and cannot be identified further as the specimen material available is not sufficient to make a satisfactory identification to the specific level. In the case where no elevational information was available for a species; an elevation estimate of the named place locality was made using Google Earth. Exotic species are marked by an asterisk (*) before the species name, while species names preceded by (^E^) and (^NE^) signify endemic and near-endemic species respectively. Species of conservation concern are indicated as such using the IUCN Red List categories and criteria. Herbaria are listed according to their respective codes: EA (East African Herbarium, Nairobi, Kenya) and HIB (Hubei Institute of Botany, Wuhan, China). SAJIT means Sino-Africa Joint Investigation Team, NMK refers to the National Museum of Kenya and KEFRI stands for the Kenya Forestry Research Institute.

### ﻿Part 1. Lycopodiopsida

**F1. LYCOPODIACEAE**

3 Genera, 6 species

***Huperziaverticillata* (L.f.) Trevis.** – Habit: Herb. Habitat: Intermediate and montane evergreen forest, 1440–1550 m. Vouchers: Faden RB et al. 71/53 & 91 (EA).

***Lycopodiumclavatum* L.** – Habit: Herb. Habitat: Moist montane forest, 1800–2200 m. Vouchers: Kamau P & Christenhusz MJM 648, Faden RB & Evans A 70/504, Drummond RB & Hemsley JH 4306, Gillett JB 17252 (EA).

***Palhinhaeacernua* (L.) Franco & Vasc.** – Habit: Herb. Habitat: Disturbed areas in evergreen forest, loose sand, 1200–2200 m. Vouchers: Faden RB et al. NMK Exped. 416 & 1135, Beentje HJ et al. NMK Exped. 1116 (EA), Faden RB & Evans A 70/508 (EA).

***Phlegmariurusdurus* (Pic. Serm.) A.R.Field & Bostock** – Habit: Herb. Habitat: Rainforest, dry evergreen mist forest, intermediate wetter evergreen forest, 900–1500 m. Vouchers: Luke WRQ et al. 5367, Faden RB, Evans A & Githui M 70/180, 478 & 740, Faden RB et al. 71/157, Dale IR 3788, Faden RB et al. NMK Exped. 963 (EA).

***Phlegmariurusholstii* (Hieron.) A.R.Field & Bostock** – Habit: Herb. Habitat: Mist forest, 1350–1875 m. Vouchers: Luke WRQ et al. 5351B, Faden RB et al. NMK Exped. 294, 415 & 772, Faden RB et al. 69/831, 71/55 & 158 (EA). Near Threatened.

***Phlegmariurusophioglossoides* (Lam.) A.R.Field & Bostock** – Habit: Herb. Habitat: *Podocarpus* upland evergreen forest, *Newtonia*, *Macaranga* mist forest, 1800–2200 m. Vouchers: Kamau P & Christenhusz MJM et al. 619 & 650, Faden RB et al. NMK Exped. 108, Drummond RB & Hemsley JH 4321, Faden RB et al. 70/490 (EA).

**F2. SELAGINELLACEAE**

1 Genus, 4 species

***Selaginellacaffrorum* (Milde) Hieron.** – Habit: Herb. Habitat: Dry evergreen forest on seepage areas on sandstone, 1050–1585 m. Vouchers: Beentje HJ et al. NMK Exped. 896, Faden RB & Faden AJ 72/241, Schippers RR K312 (EA).

***Selaginellagoudotiana* Spring** – Habit: Herb. Habitat: Evergreen forest floor, rocks near stream sides, 1150–1250 m. Voucher: Faden RB, Evans A & Msafiri F 70/985 (EA).

***Selaginellakraussiana* (Kunze) A.Braun** – Habit: Herb. Habitat: Moist ground in forest, near streams, 1350–2200 m. Vouchers: SAJIT–004539 (EA, HIB), Kamau P & Christenhusz MJM et al. 6617, Drummond RB & Hemsley JH 4320, Faden RB et al. NMK Exped. 75 & 1133, Beentje HJ et al. NMK Exped. 1089, Faden RB & Faden AJ 77/324, Faden RB et al. 70/492 (EA).

***Selaginellamittenii* Baker** – Habit: Herb. Habitat: Wet rocks of streams and waterfalls in semi-deciduous woodland and evergreen forest, 790–1000 m. Vouchers: Faden RB et al. 69/446, Faden RB, Evans A & Msafiri F 70/968 (EA).

### ﻿Part 2. Polypodiopsida

**F3. ADIANTACEAE**

1 Genus, 2 species

***Hemionitisdoniana* (J.Sm. & Hook.) Christenh.** – Habit: Herb. Habitat: Lowland evergreen forest, woodland on damp banks, ca. 914 m. Hildebrandt JM 2477a (EA).

***Hemionitisschweinfurthii* (Hieron.) Christenh.** – Habit: Herb. Habitat: Rock outcrops, pavements, shady gullies in woodland and bushland, 600–1875 m. Vouchers: Mwachala G et al. EW3187, Faden RB, Evans A & Msafiri M 70/965, Faden RB et al. NMK Exped. 309, Schippers RR K311 (EA).

**F4. ANEMIACEAE**

1 Genus, 2 species

***Anemiacaffrorum* (L.) Christenh.** – Habit: Herb. Habitat: Forest edges, ca. 2103 m. Vouchers: Schippers RR K264, Gardner HM 2982 (EA).

***Anemiavestita* (Baker) Christenh.** – Habit: Herb. Habitat: Mist forest, roadside banks, 1500–2170 m. Vouchers: Kamau P & Christenhusz MJM et al. 624, Faden RB & Faden AJ 72/246, Faden RB, Evans A & Wolf T 69/833 (EA).

**F5. ASPLENIACEAE**

4 Genera, 44 species

***Aspleniumadiantum-nigrum* L.** – Habit: Herb. Habitat: Shaded areas in mist forest, rock crevices, road banks, 1900–2134 m. Vouchers: Faden RB et al. 70/471 & 72/250, Schippers RR K270 (EA).

***Aspleniumaethiopicum* (Burm.f.) Bech.** – Habit: Herb. Habitat: Forest undergrowth, 1400–2000 m. Vouchers: Kamau P 390, Mwachala G et al. EW3070A, Kamau P & Christenhusz MJM et al. 608, Wakanene KM & Mwangangi OM 260 & 270, Schippers RR K243, Faden RB et al. NMK Exped. 56, Beentje HJ et al. NMK Exped. 924, Faden RB & Evans A 69/325 & 838, Faden RB et al 71/11 (EA).

***Aspleniumalbersii* Hieron.** – Habit: Herb. Habitat: Mist forest, 1400–1850 m. Vouchers: Faden RB et al. NMK Exped. 424 & 959, Faden RB, Evans A & Smeenk C 70/530 & 556 (EA).

***Aspleniumanisophyllum* Kunze** – Habit: Herb. Habitat: Moist forest, 1500–1700 m. Vouchers: Luke WRQ et al. 4184, Faden RB et al. 69/826 (EA).

***Aspleniumbarteri* Hook.** – Habit: Herb. Habitat: Moist forest, near river, 1400–1850 m. Vouchers: Kamau P 445, Kamau P & Christenhusz MJM et al. 634, NMK Taita Hills Exped. 778A, 782 & 1126, Faden RB et al. 71/3 (EA).

***Aspleniumboltonii* Hook. ex Schelpe** – Habit: Herb. Habitat: Moist forest, 1500–2175 m. Vouchers: Kamau P 402, Faden RB et al. NMK Exped. 272a & 312, Faden RB & Faden AJ 77/327, Faden RB 69/874 & 70/465 (EA).

***Aspleniumbuettneri* Hieron. ex Brause** – Habit: Herb. Habitat: Shallow soil on rocks in forest, 1275–2170 m. Vouchers: Faden RB et al. NMK Exped. 316, Faden RB et al. 70/526 (EA).

***Aspleniumchristii* Hieron.** – Habit: Herb. Habitat: Moist forest, stream banks, 1400–1875 m. Vouchers: Kamau P 393, Schippers RR K240, Faden RB et al. NMK Exped. 261, Beentje HJ et al. NMK Exped. 1091, Faden RB & Faden AJ 77/321, Faden RB et al. 70/558 & 71/4 (EA).

***Aspleniumdregeanum* Kunze** – Habit: Herb. Habitat: Moist forest, waterfalls, along streams, 1250–1433 m. Vouchers: Luke WRQ & Luke PA 5362, Hemp A 5373, Schippers RR K323, Faden RB, Evans A, Rathbun G 69/468, Faden RB, Evans A, Msafiri F 70/993, Reichstein T 2894, Napper DM, Gillett JB, Bally PRO 2169 (EA).

***Aspleniumelliottii* C.H.Wright** – Habit: Herb. Habitat: Undergrowth in moist forest, 1425–2195 m. Vouchers: Kamau P 458, Kamau P & Christenhusz MJM et al 592, Faden RB et al. NMK Exped. 272 & 389, Faden RB & Githui M 70/728, Faden RB & Faden AJ 77/331, Schippers RR K280 (EA).

***Aspleniumerectum* Bory ex Willd.** – Habit: Herb. Habitat: Mist forest floor, along streams on banks, 1250–1875 m. Vouchers: Kamau P 410, Faden RB et al. NMK Exped. 267 & 397, Faden RB & Faden AJ 77/333, Schippers RR K245, K252, K296 & K322, Faden RB, Evans A, Msafiri F 70/534, 994 & 71/16, Joanna B 9087 (EA).

***Aspleniumfriesiorum* C.Chr.** – Habit: Herb. Habitat: Moist forest, 1425–2134 m. Vouchers: Kamau P 398, Schippers RR K266, Faden RB et al. NMK Exped. 314 & 968, Faden RB et al. 69/821 (EA).

***Aspleniumholstii* Hieron.** – Habit: Herb. Habitat: Wet evergreen forest on moist rocks, logs, under waterfalls, 1350–1875 m. Vouchers: Kamau P 394 & 417, Luke WRQ & Luke PA 4145, Faden RB et al. NMK Exped. 320, 418 & 966, Schippers RR K256 & 325, Gillett JB, Burtt BL & Osborn RM 17133, Faden RB et al. 69/834, 70/466 & 555 (EA).

***Aspleniumhypomelas* Kuhn** – Habit: Herb. Habitat: Moist primary forest on tree ferns or terrestrial, 1350–2050 m. Vouchers: Kamau P 443, Kamau P & Christenhusz MJM et al. 618, Beentje HJ et al. NMK Exped. 1083, Faden RB et al. 69/820 (EA).

***Aspleniuminaequilaterale* Willd.** – Habit: Herb. Habitat: Moist forest on rocks in deep shade, streams banks, 1425–1925 m. Vouchers: Kamau P 459, Faden RB et al. NMK Exped. 409, Schippers RR K239 & 324, Faden RB et al. 69/863 (EA).

***Aspleniumlinckii* Kuhn** – Habit: Herb. Habitat: Deeply shaded wet evergreen forest, 1425–1925 m. Vouchers: Kamau P 395, Kamau P & Christenhusz MJM et al. 584, Faden RB et al. NMK Exped. 270 & 410, Schippers RR K238 & 255A, Faden RB & Faden AJ 69/865 & 77/329, Drummond RB & Hemsley JH 4340, Lubai lK HB 26 (EA).

***Aspleniumloxoscaphoides* Baker.** – Habit: Herb. Habitat: Moist montane forest, on rocks, along river, 1400–1800 m. Voucher: Kamau P, Christenhusz MJM, Chase MW, Mbale M & Kyaa J 641 (EA).

***Aspleniumgemmiferum* Schrad.** – Habit: Herb. Habitat: Moist forest, 1700–1950 m. Voucher: Kamau P, Christenhusz MJM, Chase MW, Mbale M & Kyaa J 590 (EA).

***Aspleniummacrophlebium* Baker** – Habit: Herb. Habitat: Intermediate wet evergreen forest, 1425–1875 m. Vouchers: Kamau P 424, Kamau P & Christenhusz MJM 583 & 589, Faden RB et al. NMK Exped. 321 & 960, Faden RB & Faden AJ 77/323, Faden RB & Githui M 70/754 (EA).

***Aspleniummannii* Hook.** – Habit: Herb. Habitat: Epiphytic in moist forest, 1530–1630 m. Voucher: Faden RB et al. 70/488 (EA).

***Aspleniummegalura* Hieron.** – Habit: Herb. Habitat: Moist forest, often near streams, 1250–1950 m. Vouchers: Kamau P 436, Kamau P & Christenhusz MJM 598, Faden RB et al. 71/49 & 137, Faden RB et al. NMK Exped. 965 (EA).

***Aspleniumnormale* D.Don** – Habit: Herb. Habitat: Moist primary forest, 1200–1850 m. Vouchers: Kamau P 386, Luke WRQ & Luke PA 4121b, Faden RB et al. NMK Exped. 375, Faden RB et al. 69/829 (EA).

***Aspleniumvariabile* Hook.** – Habit: Herb. Habitat: Moist forest, along streams and waterfalls, 1425–1850 m. Vouchers: Kamau P 403 & 425, Kamau P & Christenhusz MJM et al. 640, Faden RB et al. NMK Exped. 366, Bytebier B 1179, Faden RB et al. 70/561 (EA).

***Aspleniumpeteri* Bech.** – Habit: Herb. Habitat: Mist or swamp forest, ca. 1480 m. Voucher: Luke WRQ et al. 5368 (EA).

***Aspleniumrutifolium* (Bergius) Kunze** – Habit: Herb. Habitat: Moist and dry evergreen forest, dry rocks in shade, river banks, 762–1780 m. Vouchers: Kamau P 434, Schippers RR K317A, Faden RB et al. 69/327 & 70/451, Hildebrandt JM 2470 (EA).

***Aspleniumsandersonii* Hook.** – Habit: Herb. Habitat: Epiphytic on tree trunks in forest, 950–1400 m. Vouchers: Mungai GM et al. EW3166, Faden RB et al. 69/470, Joanna B 8844, Drummond RB & Hemsley JH 4374 (EA).

***Aspleniumsimii* A.F.Braithw. & Schelpe** – Habit: Herb. Habitat: Epiphytic in mist forest, 1500–1630 m. Vouchers: Beentje HJ et al. NMK Exped. 833, Faden RB et al. 70/484 (EA).

***Aspleniumsmedsii* Pic. Serm.** – Habit: Herb. Habitat: Moist upland forest, ca. 1830 m. Voucher: Faden RB & Faden AJ 77/327 (EA).

***Asplenium* sp. A** – Habit: Herb. Habitat: Epiphytic in forest, 1400–1800 m. Voucher: Kamau P, Christenhusz MJM, Chase MW, Mbale M & Kyaa J 637 (EA).

***Aspleniumstuhlmannii* Hieron.** – Habit: Herb. Habitat: Moist forest on rock outcrops and crevices, 1275–1325 m. Voucher: Faden RB et al. 70/520 (EA).

***Aspleniumtheciferum* (Kunth) Mett.** – Habit: Herb. Habitat: Low to high epiphyte in forest, woodland or thicket, on rocks, 1067–1950 m. Vouchers: Kamau P 397, Kamau P & Christenhusz MJM 591, Wakanene KM & Mwangangi OM 672, Schippers RR K251 & 292, Drummond RB & Hemsley JH 4396, Faden RB et al. NMK Exped. 197, Beentje HJ et al. NMK Exped. 826, Faden RB et al. 70/480 (EA).

***Hymenaspleniumunilaterale* (Lam.) Hayata** – Habit: Herb. Habitat: Moist forest, near streams and waterfalls, 1440–1950 m. Vouchers: Kamau P 431, Kamau P & Christenhusz MJM 600, Faden RB et al. 70/470, 540 & 71/245, van Someren C 850, Faden RB et al. s.n. (EA).

***Phegopteriscruciata* (Willd.) Mett.** – Habit: Herb. Habitat: Streambank in wet evergreen forest, moist sites, 1440–2200 m. Vouchers: Faden RB et al. 70/500 & 577 (EA).

***Thelypterisbergiana* (Schltdl.) Ching** – Habit: Herb. Habitat: Evergreen forest along rivulet and in wet places, 1219–2170 m. Vouchers: Reichstein & Bally PRO 2917, Faden RB et al. NMK Exped. 1150, Schippers RR K275 & 301, Faden RB et al. 70/499 (EA).

***Thelypterischaseana* Schelpe** – Habit: Herb. Habitat: Mist forest along stream sides, riverine forest, river valleys with *Terminalia*, *Combretum*, *Acacia*, 800–1525 m. Vouchers: Kabuye CHS et al. NMK Exped. 631, Faden RB, Evans A, Githui M, Osborn R, Smeenk C 71/243 (EA).

***Thelypterisconfluens* (Thunb.) C.V.Morton** – Habit: Herb. Habitat: Swampy areas, 1800–1925 m. Voucher: Faden RB & Evans A 69/885 (EA).

***Thelypterisdentata* (Forssk.) E.P.St.John** – Habit: Herb. Habitat: Evergreen forest, usually riverine, 870–1550 m. Vouchers: Schippers RR K235, 248 & 304, Faden RB et al. 70/463 & 71/222, Beentje HJ et al. NMK Exped. 830 (EA).

***Thelypterisgueintziana* (Mett.) Schelpe** – Habit: Herb. Habitat: By forest streams, rock faces above rivers, wet ditches along road, 870–1600 m. Vouchers: Schippers RR K234, 236 & 299, Faden RB et al. 69/828, 70/448 & 450 (EA).

***Thelypterisinterrupta* (Willd.) K.Iwats.** – Habit: Herb. Habitat: Swampy area, riverine bushland, 1275–1282 m. Vouchers: Kamau P 440, Faden RB et al. 70/519 (EA).

***Thelypterismadagascariensis* (Fée) Schelpe** – Habit: Herb. Habitat: Evergreen rainforest, forest edges, roadside, 1300–1875 m. Vouchers: Faden RB et al. NMK Exped. 471, Schippers RR K247, Gillett JB et al. 18768, Faden RB et al. 70/468 (EA).

***Thelypterisoppositiformis* (C.Chr.) Ching** – Habit: Herb. Habitat: Evergreen forest in moist places, 2000–2208 m. Voucher: Faden RB et al. 72/249 (EA).

***Thelypterispozoi* (Lag.) C.V.Morton** – Habit: Herb. Habitat: Upland forest, often by streams, 1900–2205 m. Voucher: Faden RB et al. 72/242 (EA).

***Thelypterispulchra* (Bory ex Willd.) Schelpe** – Habit: Herb. Habitat: Riverine forest, stream banks, 1425–2000 m. Vouchers: Faden RB et al. 71/236, Beentje HJ et al. NMK Exped. 925 (EA).

^**NE**^***Thelypterisusambarensis* (Holttum) Christenh.** – Habit: Herb. Habitat: Evergreen forest, ca. 1829 m. Voucher: Joanna B 9035 (EA). Endangered.

**F6. ATHYRIACEAE**

2 Genera, 3 species

***Depariaboryana* (Willd.) M.Kato** – Habit: Herb. Habitat: Evergreen forest by streams, 1740–2000 m. Voucher: Beentje HJ et al. NMK Exped. 937 (EA).

***Depariazanzibarica* (Baker) Christenh.** – Habit: Herb. Habitat: Wet evergreen forest, 2000–2200 m. Voucher: Faden RB et al. 70/495 (EA).

***Diplaziumnemorale* (Baker) Schelpe** – Habit: Herb. Habitat: Moist gullies in intermediate wet evergreen forest, 1525–1850 m. Vouchers: Kamau P 428, Faden RB et al. 70/756 (EA).

**F7. BLECHNACEAE**

2 Genera, 2 species

***Blechnumbakeri* C.Chr.** – Habit: Herb. Habitat: Intermediate wet evergreen forest, 1400–1850 m. Vouchers: Faden RB et al. 71/178, Faden RB & Githui M 70/752 (EA).

***Lomaridiumattenuatum* (Sw.) Gasper & V.A.O.Dittrich** – Habit: Herb. Habitat: Moist montane and evergreen forest, rocky stream banks and waterfalls, 1755–1850 m. Vouchers: Kamau P 426, Kamau P & Christenhusz MJM et al. 623, Luke WRQ & Luke PA 4124, Faden RB & Githui M 70/732 (EA).

**F8. CYATHEACEAE**

1 Genus, 3 species

***Alsophiladregei* (Kunze) R.M.Tryon** – Habit: Tree. Habitat: Forest along streams, 1100–1850 m. Vouchers: Kamau P 438, Mwachala G et al. EW3544, Faden RB et al. 70/518, Beentje HJ et al. NMK Exped. 1166 (EA).

***Alsophilahumilis* (Hieron.) Pic.Serm.** – Habit: Tree. Habitat: Moist forest gullies, intermediate wet evergreen forest, 1950–2208 m. Vouchers: Kamau P & Christenhusz MJM et al. 617, Faden RB & Githui M 70/757 (EA).

***Alsophilamanniana* (Hook.) R.M.Tryon** – Habit: Tree. Habitat: Hilltops, shaded and open slopes of evergreen forest, 1525–2205 m. Vouchers: Kamau P & Christenhusz MJM et al. 628, Faden RB et al. NMK Exped. 757, Schippers RR K267, Faden RB & Githui M 70/716, Faden RB & Faden AJ 72/256, Gillett JB 17246 (EA).

**F9. DAVALLIACEAE**

1 Genus, 1 species

***Davalliachaerophylloides* (Poir.) Steud.** – Habit: Herb. Habitat: Mist forest, 820–900 m. Voucher: Faden RB et al. 70/162B (EA).

**F10. DENNSTAEDTIACEAE**

4 Genera, 8 species

***Blotiellaglabra* (Bory) R.M.Tryon** – Habit: Herb. Habitat: Mist forest, rainforest, ca. 1371 m. Voucher: Dale IR 3794 (EA).

**^NE^*Blotiellahieronymi* (Kümmerle) Pic. Serm.** – Habit: Herb. Habitat: Forest floor, margins in intermediate rainforest, 1450–2000 m. Vouchers: Kamau P & Christenhusz MJM et al. 582 & 620, Wakanene KM & Mwangangi OM 219, Hemp A 5368, Faden RB, Evans A & Smeenk C 70/563b & 563c, Faden RB, Evans A & Wolf 69/844, Gillett JB, Burtt BL & Osborn RM 17147 (EA). Endangered.

***Blotiellastipitata* (Alston) Faden** – Habit: Herb. Habitat: Mist forest, wet evergreen forest, 1000–1900 m. Vouchers: Kamau P 391, WRQ Luke & PA Luke 4142, Faden RB et al. NMK Exped. 271 & 815, Hemp A 5370, Faden RB et al. 69/458 & 71/175, Drummond RB & Hemsley JH 4355 (EA).

***Blotiellastipitata* (Alston) Faden subsp. nov** – Habit: Herb. Habitat: Mist forest, 1400–1680 m. Vouchers: Faden RB, Evans A & Msafiri F 71/39, Faden RB, Evans A & Smeenk C 70/563A (EA).

***Blotiellastipitata* (Alston) Faden × *Blotiellaglabra* (Bory) R.M.Tryon** – Habit: Herb. Habitat: Montane forest, 1400–1600 m. Voucher: Faden RB et al. 71/174 (EA).

***Hypolepissparsisora* (Schrad.) Kuhn** – Habit: Herb. Habitat: Wet upland and evergreen montane forest, 1530–1785 m. Vouchers: Kamau P & Christenhusz MJM et al. 599, van Someren HD 855, Faden RB et al. 70/469, Faden RB et al. NMK Exped. 469 (EA).

**Hypolepisaff.rugosulasubsp.africana (C.Chr.) Schwartsb. & J.Prado** – Habit: Herb. Habitat: Forest clearings, 1425–1850 m. Voucher: NMK Taita Hills Exped. 401 (EA).

***Microlepiaspeluncae* (L.) T.Moore** – Habit: Herb. Habitat: Forest and plantation edges, ditch sides, stream sides, ca. 1766 m. Voucher: Kamau P 415 (EA).

***Pteridiumaquilinum* (L.) Kuhn** – Habit: Herb. Habitat: Rainforest clearings and margins, riparian forest, rocky places, 1275–1645 m. Vouchers: Kamau P & Christenhusz MJM et al. 606, Wakanene KM & Mwangangi OM 248, Schippers RR K237, Faden RB et al. 69/814, Faden RB, Faden AJ, Evans A & Smeenk C 70/522, Faden RB, Faden AJ, Evans A, Kariuki & Smeenk C 71/189 (EA).

**F11. EQUISETACEAE**

1 Genus, 1 species

***Equisetumramosissimum* Desf.** – Habit: Herb. Habitat: Riparian forest, 800–1000 m. Vouchers: Kabuye CHS et al. NMK Exped. 836 (EA), Mwachala G et al. EW1684 (EA), Faden RB et al. 70/446 (EA).

**F12. GLEICHENIACEAE**

1 Genus, 1 species

***Dicranopterislinearis* (Burm.f.) Underw.** – Habit: Herb. Habitat: Rainforest, rocky banks by streams, 1067–1850 m. Vouchers: Faden RB et al. NMK Exped. 419, Beentje HJ et al. NMK Exped. 1041, Schippers RR K283, Bytebier B, Luke WRQ & Pakia M 5360, Goyder DJ, Masinde PS, Whitehouse C 4020, Faden RB et al. 69/817 & 71/190, Bally PRO et al. 2164, Gardner HM 2943, Gillett JB, Burtt BL & Osborn RM 17163 (EA).

**F13. HYMENOPHYLLACEAE**

2 Genera, 12 species

***Hymenophyllumcapillare* Desv.** – Habit: Herb. Habitat: Moist forest on tree trunks and moist cliff surfaces, 1450–1660 m. Vouchers: Kamau P & Christenhusz MJM et al. 644, Luke WRQ & Luke PA 4214, Faden RB & Faden AJ 71/1022 (EA).

***Hymenophyllumkuhnii* C.Chr.** – Habit: Herb. Habitat: Rock faces in moist forest, 1200–1850 m. Vouchers: Luke WRQ et al. 4186, Beentje HJ et al. NMK Exped. 973, Faden RB et al. NMK Exped. 443, Faden RB & Githui M 70/705, Napper DM, Gillett JB & Bally PRO 2173, Faden RB, Evans A & Wolf T 69/845 & 849 (EA).

***Hymenophyllumsibthorpioides* (Bory ex Willd.) Mett. ex Kuhn** – Habit: Herb. Habitat: Shady rocks in mist forest, 1200–1850 m. Vouchers: Kamau P 419, Luke WRQ & Luke PA 4095D, Beentje HJ et al. NMK Exped. 974, Faden RB, Evans A & Wolf T 69/848, Napper DM, Gillett JB & Bally PRO 2172, Faden RB et al. 70/546 (EA).

**^NE^*Hymenophyllum* sp. A of FTEA** – Habit: Herb. Habitat: Moist shady rocks in moist forest, 1788–1850 m. Vouchers: Kamau P 456, Kamau P & Christenhusz MJM et al. 644 (EA).

***Hymenophyllumsplendidum* Bosch** – Habit: Herb. Habitat: Moist forest near water, ca. 1200 m. Voucher: Napper DM, Gillett JB & Bally PRO 2175 (EA).

***Trichomaneschevalieri* Christ** – Habit: Herb. Habitat: Rocks in moist forest, often on spray zone, 1400–1600 m. Voucher: Faden RB et al. NMK Exped. 59 (EA).

***Trichomanescupressoides* (Desv.)** – Habit: Herb. Habitat: Moist forest in wet places, ca. 1067 m. Voucher: Schippers RR K293 (EA).

***Trichomanesdiaphanum* Kunth** – Habit: Herb. Habitat: In moist forest by streams, moss covered rocks, 1400–1850 m. Vouchers: Luke WRQ et al. 4187 (EA), Faden RB et al. 70/702 (EA).

***Trichomaneserosum* Willd.** – Habit: Herb. Habitat: Moist forest on rocks and tree ferns, 1423–1850 m. Vouchers: Kamau P & Christenhusz MJM et al. 635, Faden RB & Githui M 70/718, Schippers RR K327 (EA).

***Trichomanesfrappieri* Cordem.** – Habit: Herb. Habitat: Moist forest near water or rock surfaces by falls, 1275–1325 m. Voucher: Faden RB et al. 70/524 (EA).

***Trichomanesmelanotrichum* Schltdl.** – Habit: Herb. Habitat: Mist forest, waterfall sprays and streams, 820–2170 m. Vouchers: Schippers RR K326, Faden RB & Githui M 70/172, 523, 547 & 753, Faden RB et al. NMK Exped. 139, Faden RB et al. 69/847, Beentje HJ et al. NMK Exped. 972 (EA).

***Trichomanesrigidum* Sw.** – Habit: Herb. Habitat: Moist forest, deeply shaded stream banks, under large rocks, 1150–1270 m. Vouchers: Faden RB et al. 69/467, Faden RB et al. 70/571 & 984 (EA).

**F14. MARATTIACEAE**

1 Genus, 1 species

***Ptisanafraxinea* (Sm.) Murdock** – Habit: Herb. Habitat: Montane forest, along rivers, 1219–1850 m. Vouchers: Kamau P & Christenhusz MJM et al. 638, Schippers RR K306 & 320, Gillett JB, Burtt BL & Osborn RM 17168, Faden RB et al. NMK Exped. 788, Gardner HM 2990, Faden RB & Githui M 70/473 & 715 (EA).

**F15. OPHIOGLOSSACEAE**

1 Genus, 1 species

**OphioglossumvulgatumL.subsp.africanum Pocock ex J.E.Burrows** – Habit: Herb. Habitat: Ecotone between grassland and evergreen montane forest, 1900–2000 m. Voucher: Faden RB & Faden AJ et al. 72/245 (EA).

**OphioglossumvulgatumL.subsp.kilimandsharicum (Hieron.) J.E.Burrows** – Habit: Herb. Habitat: Montane grassland or scrub, path sides on forest edges, 1450–1525 m. Voucher: Faden RB et al. 71/240 (EA).

**F16. OSMUNDACEAE**

1 Genus, 1 species

***Osmundaregalis* L.** – Habit: Herb. Habitat: Wet evergreen forest, 2000–2170 m. Voucher: Faden RB et al. NMK Exped. 1152 (EA).

**F17. POLYPODIACEAE**

16 Genera, 23 species

**Arachniodeswebbiana(A.Braun)Schelpesubsp.foliosa (C.Chr.) Gibby, Rasbach, Reichst., Widén & Viane** – Habit: Herb. Habitat: Montane and intermediate forest, 1372–2164 m. Vouchers: Kamau P 412, Faden RB et al. NMK Exped. 148, 782 & 1153, Faden RB et al. 69/472 & 70/998, Schippers RR K278 & 321 (EA).

**Arachniodeswebbiana(A.Braun)Schelpesubsp.webbiana** – Habit: Herb. Habitat: Montane forest, ca. 2050 m. Voucher: Kamau P & Christenhusz MJM et al. 613 (EA).

***Arthropterisorientalis* (Gmel.) Posth.** – Habit: Herb. Habitat: Mist forest, 910–1875 m. Vouchers: Kamau P 596, Mungai GM et al. EW3278, Drummond RB & Hemsley JH 4393, Faden RB et al. 70/554, Faden RB et al. NMK Exped. 315, Joanna B 8881 (EA).

***Ctenitiscirrhosa* (Schumach.) Ching** – Habit: Herb. Habitat: Moist forest, often near streams, 1260–1850 m. Vouchers: Kamau P 422, Kabuye CHS et al. NMK Exped. 807, Faden RB et al. 70/549, Faden RB et al. NMK Exped. 792 (EA).

***Didymochlaenatruncatula* (Sw.) J.Sm.** – Habit: Herb. Habitat: Mist forest on stream sides and moist gullies, 1250–1850 m. Vouchers: Kamau P & Christenhusz MJM et al. 603, Faden RB et al. NMK Exped. 787, Gillett JB 18764, Faden RB et al. 69/464, 70/472 & 758, Joanna B 8983 & 9049 (EA).

***Drynarialaurentii* (Christ) Hieron.** – Habit: Herb. Habitat: Fringing forest and associated woodland, 600–1200 m. Vouchers: Archer PG 14368 & 15010 (EA).

***Dryopterisinaequalis* (Schltdl.) Kuntze** – Habit: Herb. Habitat: Montane forest, riverine forest, 1400–2170 m. Vouchers: Faden RB et al. NMK Exped. 53, 422 & 1149, Faden RB & Evans A 69/844, Joanna B 9397 (EA).

***Dryopteriskilemensis* (Kuhn) Kuntze** – Habit: Herb. Habitat: Wet montane forest, 2000–2200 m. Vouchers: Faden RB et al. NMK Exped. 164, Faden RB et al. 70/498, Schippers RR K265 & 273 (EA).

***Dryopterismanniana* (Hook.) C.Chr.** – Habit: Herb. Habitat: Moist and riparian forests, 1425–1875 m. Vouchers: Kamau P & Christenhusz MJM et al. 588, Faden RB et al. NMK Exped. 273 & 391, Faden RB et al. 69/4281, 71/219 & 77/340, Hemp A 5245 (EA).

***Dryopterispentheri* (Krasser) C.Chr.** – Habit: Herb. Habitat: Montane forest, riverine, woodland, 1425–2100 m. Vouchers: Kamau P 411 (EA), Bally PRO et al. 2972 (EA), Faden RB et al. 70/477 (EA), Faden RB et al. NMK Exped 422 (EA).

***Elaphoglossumacrostichoides* (Hook. & Grev.) Schelpe** – Habit: Herb. Habitat: Rocks and soil in moist montane forest, ca. 1700–1800 m. Voucher: Faden RB et al. 71/1018 (EA).

***Elaphoglossumdeckenii* (Kuhn) C.Chr.** – Habit: Herb. Habitat: Montane forest, ca. 1480 m. Voucher: Luke WRQ et al. 5355B (EA).

***Elaphoglossumlastii* (Baker) C.Chr.** – Habit: Herb. Habitat: Evergreen forest, 1250–1550 m. Vouchers: Faden RB, Evans A & Rathbun G 69/462, Faden RB, Evans A, Kariuki B & Smeenk C 71/148, Gillett JB 18767 (EA).

***Grammitispygmaea* (Mett.) Copel.** – Habit: Herb. Habitat: Moist montane forest, 1425–1850 m. Vouchers: Luke WRQ & Luke PA 4149, Faden RB et al. 69/846, 70/717 & 71/185, Gillett JB, Burtt BL & Osborn RM 17166, Faden RB et al. NMK Exped. 964 (EA).

***Grammitisstrangeana* (Pic.Serm.) Christenh.** – Habit: Herb. Habitat: Tree trunks in montane forest, 1500–1640 m. Vouchers: Luke WRQ et al. 4215, Faden RB, Evans A, Kariuki B & Smeenk C 71/186, Faden RB et al. 69/850 (EA).

***Lepisorusexcavatus* (Bory ex Willd.) Ching** – Habit: Herb. Habitat: Upland and montane evergreen forest, 1250–2170 m. Vouchers: Kamau P 399, Kamau P & Christenhusz MJM et al. 607, Faden RB et al. NMK Exped. 33, 297, 958, 1151, Beentje HJ et al. NMK Exped. 1094, Faden RB et al. 69/825, Faden RB et al. 70/507, 551 & 749, Schippers RR K242, 262, 281, 290 & 298 (EA).

***Lepisorusschraderi* (Mett.) Ching** – Habit: Herb. Habitat: Ravines and waterfalls, 1650–1800 m. Voucher: Faden RB et al. 72/212 (EA).

***Lomariopsiswarneckei* (Hieron.) Alston** – Habit: Herb. Habitat: Moist montane forests, 1150–1875 m. Vouchers: Kamau P & Christenhusz MJM 643, Faden RB et al. NMK Exped. 319 & 390, Faden RB, Evans A, Wolf T 69/823 (EA), Faden RB & Githui M 70/532, 730, 755 & 999, Faden RB et al. 71/179, Gardner HM 2942 (EA).

***Loxogrammeabyssinica* (Baker) M.G.Price** – Habit: Herb. Habitat: Evergreen forest, riverbanks and rocky places, 1400–2134 m. Vouchers: Kamau P & Christenhusz MJM 593, Faden RB et al. NMK Exped. 9, Beentje HJ et al. NMK Exped. 824, Faden RB et al. 69/873, Schippers RR K254 & 272, Gillett JB, Burtt BL, Osborn RM 17132 (EA).

***Megalastrumlanuginosum* (Kaulf.) Holttum** – Habit: Herb. Habitat: Moist forest, near water, 1440–1875 m. Vouchers: Kamau P 427 & 444, Kamau P & Christenhusz MJM et al. 595, Schippers RR K249, Faden RB et al. NMK Exped. 468, Faden RB et al. 70/548, Bally PRO 9036 (EA).

***Oleandradistenta* Kunze** – Habit: Herb. Habitat: Evergreen forest, rocky caves, near water, rock faces, ca. 1250 m. Voucher: Faden RB, Evans A & Rathbun G 69/465 (EA).

***Pleopeltismacrocarpa* (Willd.) Kaulf.** – Habit: Herb. Habitat: Moist evergreen forest, 1350–2195 m. Vouchers: Kamau P 392, Kamau P & Christenhusz MJM et al. 612 & 649, Schippers RR K253, 274, 277, 279 & 287, Beentje HJ et al. NMK Exped. 822 & 1093, Goyder DJ, Masinde PS & Whitehouse C 4022, Drummond RB & Hemsley JH 4395, Faden RB et al. NMK Exped. 7 (EA).

***Polystichumsinense* (Christ) Christ** – Habit: Herb. Habitat: Moist forest, near streams, 1500–2205 m. Vouchers: Kamau P & Christenhusz MJM et al. 616, Faden RB et al. NMK Exped. 162 & 1148, Faden RB & Faden AJ 72/255, Faden RB, Evans A & Wolf T 69/836 (EA).

***Tectariagemmifera* (Fée) Alston** – Habit: Herb. Habitat: 850–1900 m. Vouchers: Kamau P & Christenhusz MJM et al. 601, Schippers RR K244 & 318, Faden RB et al. NMK Exped. 277 & 1168, Beentje HJ et al. NMK Exped. 825, Gillett JB, Burtt BL & Osborn RM 17144, Faden RB, Evans A & Rathbun G 69/426 & 872, Joanna B 8908 & 9040 (EA).

**F18. PSILOTACEAE**

1 Genus, 1 species

***Psilotumnudum* (L.) P.Beauv.** – Habit: Herb. Habitat: Dry riverbeds, under cliff overhangs, rock crevices, 870–1097 m. Vouchers: Bally PRO 8573 (EA), Faden RB, Glover PE & Evans A 70/445 (EA).

**F19. PTERIDACEAE**

5 Genera, 20 species

***Actiniopterisdimorpha* Pic.Serm.** – Habit: Herb. Habitat: Forests on granite outcrops and under boulders, 1350–1400 m. Voucher: SAJIT–005368 (EA, HIB).

***Actiniopterisradiata* (Sw.) Link** – Habit: Herb. Habitat: Open deciduous woodland and bushland, rock crevices, 600–1150 m. Vouchers: Mwachala G et al. EW1190, Beentje HJ et al. NMK Exped. 654, Faden RB et al. 71/40, Faden RB, Evans A & Msafiri F 70/973 (EA).

***Adiantumcapillus*-*veneris* L.** – Habit: Herb. Habitat: Waterfalls, on wet rock, 1205–1981 m. Vouchers: Schippers RR K259, 288 & 303, Faden RB, Evans A & Wolf T 69/808, Hildebrandt JM 2537 & 8088 (EA).

***Adiantumhispidulum* Sw.** – Habit: Herb. Habitat: Near waterfall and rivers, 825–1205 m. Vouchers: Mwachala G et al. EW3328, Faden RB & Evans A & Wolf T 69/809 (EA).

***Adiantumincisum* Forssk.** – Habit: Herb. Habitat: Damp rocks, crevices, disturbed forest, 610–1524 m. Vouchers: Mwachala G et al. EW247 & 211, Kabuye CHS et al. NMK Exped. 643, Beentje HJ et al. NMK Exped. 1096, Schippers RR K316 & 285, Gillett JB & Burtt BL 17058, Bally PRO 12727, Hildebrandt JM 2473, Sacleux C 852, Abdullah et al. s.n. (EA).

***Haplopterisguineensis* (Desv.) E.H.Crane** – Habit: Herb. Habitat: Wet evergreen mist forest, on rock faces, 1150–1400 m. Vouchers: Luke WRQ et al. 4183, Faden RB, Evans A & Msafiri F 70/987, Joanna B 8842 (EA).

***Haplopterisvolkensii* (Hieron.) E.H.Crane** – Habit: Herb. Habitat: Epiphytic and on boulders in upland and mist forests, 1700–1800 m. Voucher: Faden RB et al. 71/1024 (EA).

***Hemionitisbergiana* (Schltdl.) Christenh.** – Habit: Herb. Habitat: Intermediate to montane evergreen forest, mist forest, 1700–1925 m. Vouchers: Faden RB & Evans A 69/867, Faden RB et al. NMK Exped. 550 (EA).

***Hemionitisconcolor* (Langsd. & Fisch.) Christenh.** – Habit: Herb. Habitat: Wet and dry forest floors and edges, moist bushland, 870–1875 m. Vouchers: Schippers RR K286, Faden RB et al. 70/460, Faden RB et al. NMK Exped. 538 (EA).

***Hemionitisfarinosa* (Forssk.) Christenh.** – Habit: Herb. Habitat: Forest remnants and edges, rock crevices, 1800–2170 m. Vouchers: Kamau P & Christenhusz MJM et al. 627, Faden RB et al. NMK Exped. 1146 (EA).

***Hemionitismultifida* (Sw.) Christenh.** – Habit: Herb. Habitat: Forest wet and dry habitats, 1400–1981 m. Vouchers: Kamau P & Christenhusz MJM 625, Faden RB et al. NMK Exped. 52 & 513, Schippers RR K250, 260 & 309, Faden RB et al. 70/476 (EA).

***Hemionitisquadripinnata* (Forssk.) Christenh.** – Habit: Herb. Habitat: Riparian forests, roadside banks, 1275–2200 m. Vouchers: Kamau P & Christenhusz MJM et al. 605 & 629, Faden RB et al. NMK Exped. 1128, Faden RB & Evans A 70/503 & 514, Schippers RR K261 (EA).

***Hemionitisviridis* (Forssk.) Christenh.** – Habit: Herb. Habitat: Forest edges, riverbanks, rocky crevices and outcrops, *Acacia*-*Commiphora* woodland, roadsides, 914–1768 m. Vouchers: Mwachala G et al. EW2570, Goyder DJ, Masinde PS & Whitehouse C 4021, Schippers RR K264 & 294, Hildebrandt JM 2477, Sacleux C 1365, Faden RB et al. 70/525 & 71/30, Reichstein T & Bally PRO 2916 (EA).

***Pteriscatoptera* Kunze** – Habit: Herb. Habitat: Wet evergreen forest, stream sides, forest clearings, 1158–1850 m. Vouchers: Kamau P & Christenhusz MJM et al. 585, Wakanene KM & Mwangangi OM 279, Schippers RR K233, 289 & 295, Faden RB et al. NMK Exped. 411 & 892, Drummond RB & Hemsley JH 4379, Gillett JB & Burtt BL 17128, Faden RB et al. 69/330, 449, 827, 70/464, 988 & 71/2 (EA).

***Pterisfriesii* Hieron.** – Habit: Herb. Habitat: Forest, ca. 1829 m. Voucher: Joanna B 9042 (EA).

***Pterisdentata* Forssk.** – Habit: Herb. Habitat: 1400–1500 m. Vouchers: Kamau P & Christenhusz MJM et al. 610, Beentje HJ et al. NMK Exped. 1086, Faden RB et al. NMK Exped. 58, Joanna B 8954 & 9047, Faden RB et al. 69/436, 871 & 71/34 (EA).

***Pterispteridioides* (Hook.) F.Ballard** – Habit: Herb. Habitat: Upland evergreen forest floor, stream sides, 1425–1850 m. Vouchers: Kamau P & Christenhusz MJM et al. 639, Beentje HJ et al. NMK Exped. 781, Joanna B 8991 & 9041, Faden RB et al. 71/1006 (EA).

***Pterisusambarensis* Hieron.** – Habit: Herb. Habitat: Montane forest edges and roadside banks, 1000–1925 m. Vouchers: Wakanene KM, Mwangangi OM & Dunn B 363, Luke WRQ & Luke PA 4141, 5336 & 5495, Faden RB et al. NMK Exped. 274 & 412, Gillett JB 18765, Hemp A 5248 & 5371, Gardner HM 2944, Joanna B 9033, Faden RB et al. 69/473, 853, 868, 70/729 & 77/330 (EA).

***Pterisvittata* L.** – Habit: Herb. Habitat: Forested hillslopes, cliffs, rocky areas, 800–1500 m. Vouchers: Kamau P & Christenhusz MJM et al. 646, Kabuye CHS et al. NMK Exped. 609, Schippers RR K232, Joanna B 8934 (EA).

***Vittariaisoetifolia* Bory** – Habit: Herb. Habitat: Evergreen and mist forest, 1425–1850 m. Vouchers: Faden RB et al NMK Exped 962, Faden RB, Faden AJ, Evans A & Wolf T 69/851, Faden RB et al. 71/1023 (EA).

### ﻿Part 3. Cycadopsida

**F20. ZAMIACEAE**

1 Genus, 1 species

**^NE^*Encephalartoskisambo* Faden & Beentje** – Habit: Tree. Habitat: Low canopy dry evergreen mist forest, 900–1025 m. Vouchers: Faden RB, Faden AJ, Smeenk C, Smeenk N & Kichoi D 71/965, Archer PG 606 & 614, Willing TD & Robertson IJ 85/1 (EA). Endangered.

### ﻿Part 4. Pinopsida

**F21. CUPRESSACEAE**

1 Genus, 1 species

*Callitrispreissii Miq. – Habit: Tree. Habitat: Shrubland, ca. 1371 m. Vouchers: Dyson WG 334 & 535 (EA).

**F22. PODOCARPACEAE**

2 Genera, 2 species

***Afrocarpususambarensis* (Pilg.) C.N.Page** – Habit: Tree. Habitat: Montane forest, 1700–1875 m. Voucher: Faden RB et al. NMK Exped. 540 (EA). Endangered.

***Podocarpuslatifolius* (Thunb.) R.Br. ex Mirb.** – Habit: Tree. Habitat: Upland rainforest, 1550–2170 m. Vouchers: Watuma BM W0309 (EA, HIB), Wakanene KM & Mwangangi OM 101, Bytebier B 2329, Faden RB et al. NMK Exped. 84 & 313, Drummond RB & Hemsley HJ 4353 (EA).

### ﻿Part 5. Liliopsida

**F23. AMARYLLIDACEAE**

3 Genera, 3 species

***Cryptostephanushaemanthoides* Pax** – Habit: Herb. Habitat: Evergreen woodland, deciduous bushland on granitic rocks, 720–1250 m. Vouchers: SAJIT–005364 (EA, HIB), Luke WRQ et al. 5523 & 6424, Faden RB & Faden AJ 74/252, Bjørnstad A 270, Ossent J 165, Hildebrandt JM 2578 (EA).

**Cyrtanthussanguineus(Lindl.)Walp.subsp.wakefieldii (Sealy) Nordal** – Habit: Herb. Habitat: Sandy soils in deciduous woodland, rocky slopes, ca. 1000 m. Vouchers: Faden RB et al. 69/444, Archer PG 564 (EA).

**Scadoxusmultiflorus(Martyn)Raf.subsp.multiflorus** – Habit: Herb. Habitat: Montane forest undergrowth, 1400–1763 m. Vouchers: Watuma BM W0025 & W0051 (EA, HIB), Faden RB et al. NMK Exped. 48, Faden RB et al. 71/216 (EA).

**F24. ARACEAE**

4 Genera, 4 species

***Culcasiafalcifolia* Engl.** – Habit: Climber. Habitat: Moist evergreen forest, 1425–1875 m. Vouchers: Watuma BM W0124 (EA, HIB), Joanna B 8774, NMK Taita Hills Exped. 260, Gardner HM 3009 (EA).

***Stylochaetonsalaamicum* N.E.Br.** – Habit: Herb. Habitat: Lowland evergreen forest, wooded grassland, 700–1000 m. Vouchers: Mwachala G et al. EW1357, Faden RB, Evans A & Githui M 70/155 (EA).

***Wolffiaarrhiza* (L.) Horkel ex Wimm.** – Habit: Herb. Habitat: Ditches and pools, 1700–1875 m. Voucher: Faden RB et al. NMK Exped. 1052 (EA).

***Zamioculcaszamiifolia* (G.Lodd.) Engl.** – Habit: Herb. Habitat: Humid to dry evergreen forest, often on rocks, 900–1100 m. Voucher: Faden RB & Faden AJ 71/956 (EA).

**F25. ARECACEAE**

1 Genus, 1 species

***Phoenixreclinata* Jacq.** – Habit: Tree. Habitat: Disturbed forest sites, open rocky hillsides, 1000–1850 m. Vouchers: Watuma BM W0137 (EA, HIB), Drummond RB & Hemsley JH 4369 (EA), Raler I 0019 (EA), Faden RB et al. 71/242 (EA).

**F26. ASPARAGACEAE**

5 Genera, 26 species

***Albucaabyssinica* Jacq.** – Habit: Herb. Habitat: Forest, soils on rocks and inselbergs, ca. 1794 m. Voucher: SAJIT–004520 (EA, HIB).

***Albucadonaldsonii* Rendle** – Habit: Herb. Habitat: Deciduous bushland, grassland, ca. 862 m. Vouchers: SAJIT–004623 (EA, HIB), Polhill RM & Kibuwa SP 954 (EA).

**Albucavirens(Lindl.)J.C.Manning & Goldblattsubsp.virens** – Habit: Herb. Habitat: Bushland, woodland and woodland edges, 800–1000 m. Voucher: SAJIT–005366 (EA, HIB).

***Asparagusasparagoides* (L.) Druce** – Habit: Climber. Habitat: Mist forest remnants, forest on basement complex, 1440–2170 m. Vouchers: SAJIT–004517 & 006371 (EA, HIB), Wakanene KM & Mwangangi OM 686, Beentje HJ et al. NMK Exped. 103 & 368, Joanna B 9024, Drummond RB & Hemsley JH 4322, Faden RB et al. 70/533, Gillett JB, Burtt BL & Osborn RM 1712 (EA).

***Asparagusbuchananii* Baker** – Habit: Climber. Habitat: Dry bushland, woodland, grassland, ca. 960 m. Vouchers: SAJIT–005380 (EA, HIB), Bally JB 13528 (EA).

***Asparagusfalcatus* L.** – Habit: Climber. Habitat: Dry forest margins, deciduous or evergreen bushland, 500–1610 m. Vouchers: Watuma BM W0272 (EA, HIB), Mwachala G et al. EW184, Drummond RB & Hemsley JH 4272, Bally JB 13408 (EA).

***Asparagusflagellaris* (Kunth) Baker** – Habit: Shrub. Habitat: Wooded grassland, dry bushland, regularly burned areas. Vouchers: Hucks M 450, Bally PRO 232 (EA).

***Asparagusracemosus* Willd.** – Habit: Climber/Shrub. Habitat: Forest margins, wooded grassland, secondary bushland, ca. 856 m. Vouchers: SAJIT–006417, Watuma BM W0082 (EA, HIB), Medley KE 1002 (EA).

***Asparagussetaceus* (Kunth) Jessop** – Habit: Climber/Shrub. Habitat: Forest and forest margins, 900–1875 m. Vouchers: Wakanene KM & Mwangangi OM 346, Mwachala G et al. EW820, Mungai GM et al. EW3198, Faden RB et al. NMK Exped. 211 & 436, Medley KE 991, Christenhusz MJM et al. 6623 (EA).

***Chlorophytumandongense* Baker** – Habit: Herb. Habitat: Open woodland, forest margins, riverine thicket, termite mounds, ca. 930 m. Vouchers: Polhill RM & Kibuwa SP 975, Bally 12699A (EA).

***Chlorophytumcomosum* (Thunb.) Jacques** – Habit: Herb. Habitat: Undergrowth in rainforest, riverine, woodlands, 859–1850 m. Vouchers: SAJIT–005333 & 006415, Watuma BM W0020 (EA, HIB), Beentje HJ et al. NMK Exped. 357, Drummond RB & Hemsley JH 4335, Faden RB et al. 70/750 (EA).

***Chlorophytumsparsiflorum* Baker** – Habit: Herb. Habitat: Moist clearings in forest, forest margins, 1425–1850 m. Vouchers: Beentje HJ et al. NMK Exped. 949, Drummond RB & Hemsley JH 4335 (EA).

***Chlorophytumsuffruticosum* Baker** – Habit: Herb. Habitat: Rocky wooded bushland, 800–1100 m. Voucher: SAJIT–005363 (EA, HIB).

***Chlorophytumtuberosum* (Roxb.) Baker** – Habit: Herb. Habitat: Woodland, bushland, seasonally flooded areas, 487–1000 m. Vouchers: Mwachala G et al. EW026, Greenway PJ & Kanuri 12718, Rauh W 12471 (EA).

***Dracaenaarborescens* (Cornu ex Gérôme & Labroy) Byng & Christenh.** – Habit: Herb. Habitat: Evergreen bushland, ca. 548 m. Voucher: Bally PRO 8651 (EA).

***Dracaenaafromontana* Mildbr.** – Habit: Tree. Habitat: Understorey in rainforest, 1740–2203 m. Vouchers: SAJIT–006362 (EA, HIB), Christenhusz MJM et al. 6646 (EA), Beentje HJ et al. NMK Exped. 915, Faden RB et al. NMK Exped. 1130, Kimeu JM et al. KEFRI 502 (EA).

***Dracaenafragrans* (L.) Ker Gawl.** – Habit: Tree. Habitat: Moist forest near streams, 1300–1875 m. Vouchers: SAJIT–004604 (EA, HIB), Luke WRQ & Luke PA 4166, Faden RB et al. NMK Exped. 199 & 1049, Luke WRQ et al. 5348, Gardner HM 3014, Dale IR 3766 (EA).

***Dracaenalaxissima* Engl.** – Habit: Shrub. Habitat: Moist and riverine forests, dry forest near water, 1200–1800 m. Vouchers: Beentje HJ et al. NMK Exped. 1076, Beentje HJ 2149, Faden RB & Faden AJ 72/268, Raler I 0017, Breteler FJ 7507, Bally PRO 8702, Battiscombe E 860, Gardner HM 11415 (EA).

***Dracaenaparva* N.E.Br. Byng & Christenh.** – Habit: Herb. Habitat: Dry forest, rocky bushland, termite hills, 600–1371 m. Vouchers: Joanna B 8898, Bally PRO 13416 (EA).

***Dracaenapowellii* N.E.Br. Byng & Christenh.** – Habit: Herb. Habitat: Dry bushland, scattered tree grassland, 590–700 m. Voucher: Faden RB, Evans A & Rathbun G 69/403A (EA).

***Dracaenaraffillii* N.E.Br. Byng & Christenh.** – Habit: Herb. Habitat: Dry forest, woodland, bushland, ca. 1010 m. Voucher: Mwachala G et al. EW1858 (EA).

**DracaenaserpentaByng & Christenh.var.humbertiana (Guillaumin) Byng & Christenh.** – Habit: Herb. Habitat: Dry bushland in rocky sites, thicket, ca. 600 m. Voucher: Bally PRO 13416 (EA).

***Dracaenasingularis* (N.E.Br.) Byng & Christenh.**– Habit: Herb. Habitat: Dry bushland. Vouchers: Powell 10, Pfenning 1019 (EA).

***Dracaenasteudneri* Engl.** – Habit: Tree. Habitat: Moist or dry forest margins, 1000–1640 m. Vouchers: Watuma BM W0128 (EA, HIB), Faden RB et al. 69/459, Medley KEM p.r. (EA).

***Dracaenavolkensii*** Gürke Byng & Christenh. – Habit: Herb. Habitat: *Acacia*-*Commiphora*-*Euphorbia* bushland, 600–800 m. Vouchers: Drummond RB & Hemsley JH 4420, Bos JJ 10419 (EA).

**DrimiopsisbotryoidesBakersubsp.botryoides** – Habit: Herb. Habitat: Moist shaded thickets, ca. 840 m. Voucher: Faden RB & Faden AJ 74/505 (EA).

**F27. ASPHODELACEAE**

2 Genera, 10 species

***Aloeballyi* Reynolds** – Habit: Shrub. Habitat: Deciduous bushland & *Acacia* with succulents, 609–865 m. Vouchers: Bally PRO 8115, Mrs Robertson SA 3589, Reynolds GW 6378 (EA). Endangered.

**^NE^*Aloeclassenii* Reynolds** – Habit: Herb. Habitat: Rocky outcrops in deciduous bushland, ca. 792 m. Vouchers: Archer PG 565 & 566 (EA). Critically Endangered.

***Aloedeserti* A.Berger** – Habit: Shrub. Habitat: Deciduous bushland edges on sandy soils, 548–950 m. Vouchers: Kabuye CHS et al. NMK Exped. 589, Bally PRO 8652 & 13571 (EA). Near Threatened.

**AloelateritiaEngl.var.lateritia** – Habit: Herb. Habitat: Rocky places, grassy hillsides, 1500–1250 m. Vouchers: Faden RB et al. NMK Exped. 21 & 293, Drummond RB & Hemsley JH 4289, Bally PRO 8790 (EA).

**^NE^*Aloependuliflora* Baker** – Habit: Shrub. Habitat: Rocky slabs, deciduous dry forest, thick bush edges, 700–1640 m. Vouchers: Luke WRQ et al. 5400, Luke WRQ & Luke PA 9439, Faden RB et al. NMK Exped. 21, Newton LE 3543 & 3241, Bally PRO 13303, Gillett JB 18739, Chase s.n. (EA). Endangered.

***Aloerabaiensis* Rendle** – Habit: Shrub. Habitat: Woodland, open bushland and sandy soil, ca. 1600 m. Voucher: Beentje HJ et al. NMK Exped. 1136 (EA).

***Aloeruspoliana* Baker** – Habit: Herb. Deciduous bushland on sandy and rocky soils, ca. 600 m. Voucher: Bally PRO 8791 (EA).

***Aloesecundiflora* Engl.** – Habit: Herb. Habitat: Open deciduous woodland. Vouchers: Rauh W Ke871, Reynolds 6374 (EA).

***Aloevolkensii* Engl.** – Habit: Shrub. Habitat: Dry forest on steep slopes, dense riverine woodland, ca. 1200 m. Vouchers: Luke WRQ et al. 5513, Bally PRO 12713 (EA). Near Threatened.

***Kniphofiathomsonii* Baker** – Habit: Herb. Habitat: Montane bushland and grassland on watercourse edges, 2000–2195 m. Vouchers: SAJIT–006378 (EA, HIB), Christenhusz MJM, Kamau P, Chase MW, Mbale M & Kyaa J 6651, Faden RB et al. NMK Exped. 93, Verdcourt B & Polhill RM 2719 (EA), Gardner HM (EA).

**F28. BURMANNIACEAE**

1 Genus, 1 species

**^NE^*Gymnosiphonusambaricus* Engl.** – Habit: Herb. Habit: Evergreen forest floor among leaf mould, 1300–1900 m. Vouchers: SAJIT–003290 (EA, HIB), Luke WRQ & Luke PA 4136, Bytebier B 1200 & 1217, Faden RB et al. NMK Exped. 484, Faden RB et al. 71/9, 50 & 155, Gillett JB et al 17141 & 18759, Bally PRO 8747 (EA). Endangered.

**F29. COLCHICACEAE**

1 Genus, 1 species

***Gloriosasuperba* L.** – Habit: Climber. Habitat: Rainforest, forest edges and glades, open *Acacia*-*Commiphora* woodland, 1000–1875 m. Vouchers: Mungai G et al. EW2821, Faden RB et al. NMK Exped. 590, Verdcourt B & Polhill RM 2745 (EA).

**F30. COMMELINACEAE**

4 Genera, 13 species

***Aneilemaaequinoctiale* (P.Beauv.) G.Don** – Habit: Herb. Habitat: Moist forest undergrowth and edges, 1500–1875 m. Vouchers: Faden RB et al. 69/839, Faden RB et al. NMK Exped. 245 (EA).

***Aneilemahockii* De Wild.** – Habit: Herb. Habitat: Deciduous bushland, grassland, 520–1180 m. Vouchers: Mwachala G et al. EW242, Faden RB et al. 74/502 (EA).

***Aneilemajohnstonii* K.Schum.** – Habit: Herb. Habitat: *Acacia*-*Commiphora* woodland, scrub, 985–1100 m. Vouchers: Mwachala G et al. EW229, Faden RB, Evans A & Siggins 69/323, Faden RB et al. 74/490 (EA).

***Aneilemaleiocaule* K.Schum.** – Habit: Herb. Habitat: Upland forest, shaded areas near streams, 1530–2170 m. Vouchers: Faden RB et al. NMK Exped. 78, Beentje HJ et al. NMK Exped. 904, Faden RB et al. 70/485, Gillett JB, Burtt BL & Osborn 17077 (EA).

**CommelinaafricanaL.var.villosior (C.B.Clarke) Brenan** – Habit: Herb. Habitat: Forest undergrowth and edges, woodland, grassland, roadside banks, cultivated lands, 2000–2200 m. Voucher: Faden RB et al. 70/497 (EA).

***Commelinabenghalensis* L.** – Habit: Herb. Habitat: Forest, forest edges and clearings, disturbed areas, weed of cultivation, 1400–1640 m. Voucher: Faden RB et al. NMK Exped. 23 (EA).

***Commelinabracteosa* Hassk.** – Habit: Herb. Habitat: Forest edge, moist thickets, woodland, grassland, bushland, cultivated grounds, 590–700 m. Voucher: Faden RB, Evans A & Rathbun G 69/409 (EA).

***Commelinalatifolia* Hochst. ex A.Rich.** – Habit: Herb. Habitat: Forest and thicket edges, disturbed places, roadsides, weed in cultivated lands, 1240–1830 m. Vouchers: Mungai GM et al. EW2813, Faden RB & Faden AJ 77/317 (EA).

***Commelinalukei* Faden** – Habit: Herb. Habitat: Forest edges and clearings, moist thickets, bushland, grassland, roadsides, 680–750 m. Vouchers: Faden RB, Evans A & Rathbun G 69/412, Faden RB et al. 70/149 (EA).

***Cyanotisarachnoidea* C.B.Clarke** – Habit: Herb. Habitat: Rock crevices, exposed rock faces over shallow soil on hilltops, 1700–2100 m. Vouchers: Watuma BM W0111 (EA, HIB), Kabuye CHS 82/62, Faden RB & Faden AJ 77/316, Faden RB et al. NMK Exped. 256, Drummond RB & Hemsley JH 4301, Bally PRO 13635 (EA).

***Cyanotislanata* Benth.** – Habit: Herb. Habitat: Rock outcrops in open grasslands, bushlands and woodlands, ca. 850 m. Vouchers: Mwachala G et al. EW175 & 754 (EA).

***Murdanniasemiteres* (Dalzell) Santapau** – Habit: Herb. Habitat: Mist forest near on rocky outcrops, ca. 1603 m. Voucher: Watuma BM W0223 (EA, HIB).

***Murdanniasimplex* (Vahl) Brenan** – Habit: Herb. Habitat: Rocky outcrops, forest clearings, woodland, grassland, 1676–1838 m. Vouchers: Watuma BM W0108 & W0262 (EA, HIB), Kabuye CHS et al. NMK Exped. 813, Faden RB & Faden AJ 77/315 (EA).

**F31. CYPERACEAE**

9 Genera, 42 species

***Abildgaardiaovata* (Burm.f.) Kral** – Habit: Herb. Habitat: Hilltop grasslands, wooded grassland, grazed fallow areas, ca. 1100 m. Voucher: Sacleux C 2560 (EA).

**Bulbostyliscoleotricha(Hochst. ex A.Rich.)C.B.Clarkevar.coleotricha** – Habit: Herb. Habitat: Shallow soil overlaying rock outcrops and crevices, 800–1050 m. Vouchers: Mwachala G et al. EW586 & 3297 (EA).

***Bulbostylishensii* (C.B.Clarke) R.W.Haines** – Habit: Herb. Habitat: Dry grassland in hilly areas, roadside banks, 1400–1600 m. Voucher: Kabuye CHS 82/108 (EA).

**Bulbostylishispidula(Vahl)R.W.Hainessubsp.filiformis (C.B.Clarke) R.W.Haines** – Habit: Herb. Habitat: Grassland, *Acacia* scrub, roadsides, seasonal pools and wet rock crevices, ca. 500 m. Vouchers: Muasya AM 2091, 2092 & 2093 (EA).

**Bulbostylishispidula(Vahl)R.W.Hainessubsp.halophila (Lye) R.W.Haines** – Habit: Herb. Habitat: Short grasslands, abandoned cultivations, ca. 850 m. Voucher: Gillett JB & Napper DM 2170 (EA). Endangered.

**Bulbostylishispidula(Vahl)R.W.Hainessubsp.hispidula** – Habit: Herb. Habitat: Grassland with scattered trees, shrubs, roadside, seasonally water-logged soils, 1030–1875 m. Vouchers: Mwachala G et al. EW809, Beentje HJ et al. NMK Exped. 229 & 433 (EA).

**Bulbostylishispidula(Vahl)R.W.Hainessubsp.pyriformis (Lye) R.W.Haines** – Habit: Herb. Habitat: Open *Commiphora*-*Acacia*-*Sterculia*-*Delonix*-*Euphorbia* scrub, ca. 564 m. Voucher: Polhill RM & Kibuwa SP 953 (EA).

***Carexcastanostachya* K.Schum. ex Kük.** – Habit: Herb. Habitat: Intermediate wet evergreen forest, 1500–1850 m. Vouchers: Faden RB & Githui M 70/736, Luke WRQ et al. 4190 (EA).

***Coleochloaabyssinica* (Hochst. ex A.Rich.) Gilly** – Habit: Herb. Habitat: Wet rock surfaces, hilly grasslands, ca. 1295 m. Voucher: Mungai GM et al. 3091A (EA).

***Coleochloasetifera* (Ridl.) Gilly** – Habit: Herb. Habitat: Shallow soil overlaying rocky outcrops and crevices, 610–700 m. Vouchers: Luke WRQ et al. 4172, Polhill RM & Kibuwa SP 947 (EA).

**CyperusalternifoliusL.subsp.flabelliformis** Kük. – Habit: Herb. Habitat: Streambanks, swampy areas, 950–1371 m. Vouchers: Kabuye CHS 82/31, Mwachala G et al. EW237 & 2808 (EA).

***Cyperusamabilis* Vahl** – Habit: Herb. Habitat: Seasonally wet habitats, ca. 500 m. Voucher: Muasya AM 2089 (EA).

***Cyperusamauropus* Steud.** – Habit: Herb. Habitat: Grassland and wooded grassland on rocky slopes, 500–579 m. Vouchers: Verdcourt B & Polhill RM 2713, Greenway PJ & Kanuri K 12802, Muasya AM 2095 & 2102 (EA).

***Cyperusbulbosus* Vahl** – Habit: Herb. Habitat: Open grassy places in mist forest, ca. 1873 m. Vouchers: SAJIT–003297 (EA, HIB), Fleuret A 25 (EA).

***Cyperuscyperoides* (L.) Kuntze** – Habit: Herb. Habitat: Forest clearings, path sides, grassland, woodland, stream sides, 1040–1100 m. Vouchers: Mwachala G et al. EW239 & 600 (EA).

***Cyperusdifformis* L.** – Habit: Herb. Habitat: Temporary pools, seasonally wet grasslands, along water edges, roadside ditches, 800–950 m. Voucher: Kabuye CHS et al. NMK Exped. 704 (EA).

***Cyperusdistans* L.f** – Habit: Herb. Habitat: Forest margins, moist shaded bushland, woodland, forest zones, 800–950 m. Voucher: Kabuye CHS et al. NMK Exped. 711 (EA).

**CyperusdubiusRottb.var.macrocephalus (C.B.Clarke) Kük.** – Habit: Herb. Habitat: Forest margins and clearings, riverine, dry bushland, grassland, thin soils over rock, 850–1875 m. Vouchers: Mwachala G et al. EW154, Mbale M et al. NMK 965, Muasya AM 2100, Faden RB et al. NMK Exped. 496, Verdcourt B 2741 (EA).

***Cyperuselegantulus* Steud.** – Habit: Herb. Habitat: Wet forest margins, grasslands and riverine margins. Voucher: Kirika P et al. 01/2006/17 (EA).

***Cyperusferrugineoviridis* (C.B.Clarke) Kük.** – Habit: Herb. Habitat: Grassland and forest clearings, weed of cultivated lands, 900–1050 m. Voucher: Mwachala G et al. EW602 (EA).

***Cyperusfischerianus* G.W.Schimp. ex A.Rich.** – Habit: Herb. Habitat: Forest, secondary areas in forest zone, stream sides, 998–1400 m. Voucher: Mwachala G et al. EW153 & 993, Mungai GM et al. EW3104/A (EA).

***Cyperusglaucophyllus* Boeckeler** – Habit: Herb. Habitat: Forest, secondary areas in forest zone, stream sides, 1425–1850 m. Voucher: Beentje HJ et al. NMK Exped. 429 (EA).

***Cyperusgrandibulbosus* C.B.Clarke** – Habit: Herb. Habitat: Seasonally wet habitats, wooded grasslands and grasslands, 487–600 m. Vouchers: Hucks M 575, Greenway PJ & Kanuri K 12716, Scott-Elliot 6284 (EA).

***Cuperushemisphaericus* Boeckeler** – Habit: Herb. Habitat: Open and wooded grassland, ca. 1626 m. Voucher: Kirika P et al. 01/2006/32 (EA).

***Cyperushortensis* (Salzm. ex Steud.) Dorr** – Habit. Herb. Habitat: Riverine, 800–1750 m. Vouchers: Kabuye CHS et al. NMK Exped. 709, Wakanene KM & Mwangangi OM 607 (EA).

***Cyperuslatifolius* Poir.** – Habit: Herb. Habitat: Swampy areas, roadside ditches and stream sides, 600–1350 m. Vouchers: Kabuye CHS 82/1, McDonald J 840, Bally B 13597 (EA).

**CyperuslongusL.subsp.longus** – Habit: Herb. Habitat: Temporary pools in grassland and bushland, ca. 500 m. Voucher: Faden RB, Faden AJ & Holland P 72/151 (EA).

***Cyperusmaranguensis* K.Schum.** – Habit: Herb. Habitat: Grassland, swampy grassland, weed of cultivation, roadsides, 1404–1875 m. Vouchers: Mbale M et al. NMK 963, Beentje HJ et al. NMK Exped. 431 & 585, Gilbert M & Gilbert C 6108 (EA).

***Cyperusmicrostylus* (C.B.Clarke) Mattf. & Kük.** – Habit: Herb. Habitat: Shallow soil over rock, bushland, scattered tree grassland, 840–1020 m. Vouchers: Mbale M, Muthoka P, Chesire C & Hay F NMK 972, Faden RB & Faden AJ 74/510 (EA).

***Cyperusmollipes* (C.B.Clarke) K.Schum.** – Habit: Herb. Habitat: Grassland, open woodland, bushed grassland, ca. 450–600 m. Vouchers: Muasya AM 2086, 2103 & 2107 (EA).

***Cyperuspectinatus* Vahl** – Habit: Herb. Habitat: Floating on shallow waters, ca. 1573 m. Voucher: Mbale M et al. NMK 969 (EA).

***Cyperusplateilema* (Steud.) Kük.** – Habit: Herb. Habitat: Roadsides in rainforest, montane grassland, swampy sides, stream banks, 1400–1640 m. Vouchers: Kabuye CHS 82/52, Faden RB et al. NMK Exped. 18 (EA).

***Cyperuspurpureoviridis* Lye** – Habit: Herb. Habitat: Montane forests, steep rocky slopes, swampy habitats edges, ca. 1573 m. Voucher: Mbale M et al. NMK 971 (EA). Near Threatened.

***Cyperusrichardii* Steud.** – Habit: Herb. Habitat: Grassland in damp sites, roadsides, ca. 1515 m. Voucher: Mbale M, Muthoka P, Chesire C & Hay F NMK 966 (EA).

***Cyperusrohlfsii* Boeckeler** – Habit: Herb. Habitat: Rocky outcrops, scattered tree grassland, 610–1326 m. Vouchers: Mbale M et al. NMK 959, Polhill RM & Kibuwa SP 946 (EA).

***Cyperusrotundus* L.** – Habit: Herb. Habitat: Forest glades, damp sites, riverbanks, ca. 998 m. Voucher: Mwachala G et al. EW116 (EA).

***Cyperusrubicundus* Vahl** – Habit: Herb. Habitat: Seasonally wet habitats, temporary pools or swamps, 453–950 m. Vouchers: Kabuye CHS et al. NMK Exped. 596, Muasya AM 2106 (EA).

***Cyperusschimperianus* Steud.** – Habit: Herb. Habitat: Sandy or stony riverbanks, ca. 450 m. Voucher: Faden RB, Faden AJ & Holland P 72/145 (EA).

**^NE^*Cyperusscott-elliotii* Govaerts** – Habit: Herb. Habitat: Shallow soil over rock, swampy grassland, ca. 500 m. Voucher: Muasya AM et al. 2090 (EA).

**Cyperussesquiflorus(Torr.)Mattf. & Kük.subsp.sesquiflorus** – Habit: Herb. Habitat: Open forest, forest margins, open grassland, 1371–2103 m. Vouchers: Kabuye CHS 82/26, Osborn RM 8, Joanna B 8817 (EA).

***Eleocharisatropurpurea* (Retz.) J.Presl & C.Presl** – Habit: Herb. Habitat: Seasonal pools and seepage areas, ca. 500 m. Voucher: Faden RB, Faden AJ & Holland P 72/149 (EA).

***Fimbristylisdichotoma* (L.) Vahl** – Habit: Herb. Habitat: Riverbanks, grasslands, cultivated areas, 950–1371 m. Vouchers: Watuma BM W0249 (EA, HIB), Kabuye CHS 82/22, Mwachala G et al. EW770 (EA).

**Fuirenapubescens(Poir.)Kunthvar.pubescens** – Habit: Herb. Habitat: Seasonally wet grasslands, swamp and stream edges, ca. 1150 m. Voucher: Mwachala G et al. EW3551 (EA).

**FuirenastrictaSteud.subsp.chlorocarpa (Ridl.) Lye** – Habit: Herb. Habitat: Seasonally wet grasslands, swamp and stream edges, 1371 m. Voucher: Kabuye CHS 82/23 (EA).

***Sclerialithosperma* (L.) Sw.** – Habit: Herb. Habitat: Shady and open places in evergreen forest, 1030–1150 m. Vouchers: Mwachala G et al. EW607 & 792 (EA).

**F32. DIOSCOREACEAE**

1 Genus, 1 species

***Dioscoreaasteriscus* Burkill** – Habit: Climber. Habitat: Rain-forest, dry evergreen forest, forest edges, 1425–1850 m. Voucher: Beentje HJ et al. NMK Exped. 1098 (EA).

**F33. HYPOXIDACEAE**

1 Genus, 3 species

***Hypoxisschimperi* Baker** – Habit: Herb. Habitat: Grassland, swampy grassland, ca. 2050 m. Voucher: Gillett JB 17261 (EA).

***Hypoxisrigidula* Baker** – Habit: Herb. Habitat: Open grassland or woodland. Voucher: Joanna B 9023 (EA).

***Hypoxisurceolata* Nel** – Habit: Herb. Habitat: Open bushland, disturbed woodland, grassland, 600–800 m. Voucher: Hildebrandt JM 2542 (EA).

**F34. IRIDACEAE**

1 Genus, 4 species

***Gladioluscandidus* (Rendle) Goldblatt** – Habit: Herb. Habitat: Moist highlands in rock outcrops, woodland and bushland, 610–1000 m. Vouchers: Sacleux CJ 2279, Johnston HH s.n. (EA).

**GladiolusdaleniiVan Geelsubsp.dalenii** – Habit: Herb. Habitat: Open grassland on rocky outcrops, 1800–1850 m. Voucher: Watuma BM W0066 (EA, HIB).

^**NE**^***Gladiolusrupicola* Vaupel** – Habit: Herb. Habitat: Mountains in grasslands or in rocky outcrops, 1280–2230 m. Vouchers: Lavranos JJ & Newton 17640, Dale IR 3802, Gardner HM 2957 (EA).

**^NE^*Gladiolususambarensis* Marais ex Goldblatt** – Habit: Herb. Habitat: Montane habitats, rocky sites and cliffs, ca. 1219 m. Vouchers: Archer PG 547, Bally PRO 13579, Joanna B 8840 (EA). Near Threatened.

**F35. MUSACEAE**

1 Genus, 1 species

***Enseteventricosum* (Welw.) Cheesman** – Habit: Herb. Habitat: Upland evergreen forests, ravines, riverbanks, drier lowland forests, 1000–2100 m. Vouchers: Faden RB et al. NMK Exped. 456, Medley p.r. (EA).

**F36. ORCHIDACEAE**

27 Genera, 69 species

***Aerangisconfusa* J.Stewart** – Habit: Herb. Habitat: Forest, dry forest and bush, woodland, ca. 1180 m. Voucher: Mungai GM et al. EW240 (EA).

***Aerangiskotschyana* (Rchb.f.) Schltr.** – Habit: Herb. Habitat: Forest, woodland, humid wooded grassland, ca. 1050 m. Vouchers: Mungai GM et al. EW1660 (EA).

***Aerangissomalensis* (Schltr.) Schltr.** – Habit: Herb. Habitat: Woodland along streams, 700–969 m. Vouchers: van der Laan FM 419 (EA), van der Laan FM & Wubben JM 1286 (WAG), Piers F s.n. (K).

***Aerangisthomsonii* (Rolfe) Schltr.** – Habit: Herb. Habitat: Montane forest, 1818–2200 m. Vouchers: SAJIT–004538 (EA, HIB), Osborn RM 15 (EA).

**AngraecumeburneumBorysubsp.giryamae (Rendle) Senghas & P.J.Cribb** – Habit: Herb. Habitat: On trees and rocks in forest, ca. 929 m. Vouchers: Archer PG 605, Mr. & Mrs. Stewart DRM 1292, Wakefield TG & Grant s.n. (EA).

***Angraecumsacciferum* Lindl.** – Habit: Herb. Habitat: Dense mist forest, 1480–1875 m. Vouchers: SAJIT–004163 (EA, HIB), Bytebier B 1148 & 1189, Faden RB et al. NMK Exped. 457, Luke WRQ & Luke PA 4155 (EA).

**Angraecumcf.sacciferum Lindl.** – Habit: Herb. Habitat: High epiphyte in montane evergreen forest, 1650–1800 m. Voucher: Faden RB et al. 72/205 (EA).

***Anselliaafricana* Lindl.** – Habit: Herb. Habitat: Dense mist forest, ca. 1524 m. Voucher: van Someren CGR 827 (EA). Vulnerable.

**BrachycorythispleistophyllaRchb.f.var.pleistophylla** – Habit: Herb. Habitat: Deciduous woodland edges, glades in forest, ca. 1325 m. Vouchers: Faden RB et al. 71/987, Joanna B 8919, van Someren CGR 115 (EA).

**Bolusiellairidifolia(Rolfe)Schltr.subsp.iridifolia** – Habit: Herb. Habitat: Forest and savannah trees, ca. 1600 m. Voucher: Bytebier B 1188 (BR, EA).

***Bulbophyllumintertextum* Lindl.** – Habit: Herb. Habitat: Intermediate to montane evergreen forest, mist forest, 1640–1730 m. Vouchers: Faden RB & Faden AJ 72/263, Faden RB et al. 70/778, Bytebier B 1211 & 1324 (EA).

**Bulbophyllumjosephi(Kuntze)Summerh.var.josephi** – Habit: Herb. Habitat: Mist forest, 1560–1640 m. Vouchers: Luke WRQ & Luke PA 4163, Bytebier B 1210, Faden RB, Evans A & Smeenk C 70/565 (EA).

***Calanthesylvatica* (Thouars) Lindl.** – Habit: Herb. Habitat: Riverine forest, 1425–1850 m. Vouchers: Beentje HJ et al. NMK Exped. 995 (EA), Faden RB et al. NMK Exped. 764 (EA).

***Cheirostylislepida* (Rchb.f.) Rolfe** – Habit: Herb. Habitat: Forest floor in leaf mounds, 1440–1680 m. Vouchers: SAJIT–004267 (EA, HIB), Faden RB, Evans A, Smeenk C 70/545, Bytebier B 1678 (EA).

**^NE^CynorkisbuchwaldianaKraenzl.subsp.braunii (Kraenzl.) Summerh.** – Habit: Herb. Habitat: Montane forest in open grassland, heath zone, 1643–2195 m. Vouchers: Wakanene KM & Mwangangi OM 636, Beentje HJ et al. NMK Exped. 859, Bytebier B 1661 & 1675, Dale IR 3778, Archer PG 571 & 725, Napper DM et al. 2166, Gardiner N s.n. (EA). Near Threatened.

**^NE^*Cynorkisuncata* (Rolfe) Kraenzl.** – Habit: Herb. Habitat: Secondary forest in humus layer, 1400–1850 m. Vouchers: SAJIT–005324 (EA, HIB), Mwachala G et al. EW3573, Wakanene KM & Mwangangi OM 360, Beentje HJ et al. NMK Exped. 997 & 1067, Faden RB & Faden AJ 72/260, Bytebier B 1153, 1161 & 1753, Archer PG 611, Campbell H s.n. (EA). Vulnerable.

***Cyrtorchisarcuata* (Lindl.) Schltr.** – Habit: Herb. Habitat: Forest, woodland, bushland on trees and rocks, ca. 1050 m. Voucher: Mungai GM et al. EW1661 (EA).

***Diaphananthefragrantissima* (Rchb.f.) Schltr.** – Habit: Herb. Habitat: Forest, gallery forests, 868–900 m. Voucher: Archer PG 603 (EA).

***Diaphananthelorifolia* Summerh.** – Habit: Herb. Habitat: In montane and riverine forest. No voucher seen. Recorded in *Orchids of Kenya* (Stewart and Campbell 1996).

***Diaphanantheodoratissima* (Rchb.f.) P.J.Cribb & Carlsward** – Habit: Herb. Habitat: Primary and secondary forest on rocks or trees, ca. 1219 m. Voucher: Archer PG 582 (EA).

***Disperiskilimanjarica* Rendle** – Habit: Herb. Habitat: Shaded evergreen forest, ca. 1700–2200 m. Voucher: SAJIT–004288 (EA, HIB).

***Disperisnemorosa* Rendle** – Habit: Herb. Habitat: Mossy places and leaf litter on floor of evergreen and mist forest, 1730–1900 m. Vouchers: Watuma BM W0105 (EA, HIB), Bytebier B 1662, Beentje HJ 2184, Faden RB & Evans A 69/883, Dale IR 3790 (EA).

***Disperisreichenbachiana* Welw. ex Rchb.f.** – Habit: Herb. Habitat: Forest epiphyte, ca. 1350–2100 m. Voucher: SAJIT–004195 (EA, HIB).

***Eulophiamechowii* (Rchb.f.) T.Durand & Schinz** – Habit: Herb. Habitat: Grassland and wooded grassland, ca. 1524 m. Voucher: Gardner HM 3018 (EA).

***Eulophiaschweinfurthii* Kraenzl.** – Habit: Herb. Habitat: Grassland, thicket, bushland, woodland, 1220–1525 m. Voucher: Joanna B 8773 (EA).

***Eulophiaspeciosa* (R.Br.) Bolus** – Habit: Herb. Habitat: In grassland, deciduous bushland and woodland, ca. 609 m. Voucher: Napier ER 1038 (EA).

**EulophiastreptopetalaLindl.var.streptopetala** – Habit: Herb. Habitat: Forest margins and grasslands, 1580–1730 m. Vouchers: Mwachala G et al. 3068A, Beentje HJ 2185 (EA).

**EulophiastreptopetalaLindl.var.stenophylla (Summerh.) P.J.Cribb** – Habit: Herb. Habitat: Shade of bushes, open grasslands, rocky hillsides, 1430–2000 m. Vouchers: SAJIT–004582 (EA, HIB), Friis I & Hansen OJ 2685, Bally PRO 8703, 8772 & 8779 (EA).

***Eulophiataitensis* Pfennig & P.J.Cribb** – Habit: Herb. Habitat: *Acacia* thicket on sandy soils, woodland, 1000–1348 m. Vouchers: Watuma BM W0235 (EA, HIB), Pfennig H 1113, Meyer H 1432 (EA).

***Habenariahuillensis* Rchb.f**. – Habit: Herb. Habitat: Grassland without trees, rock crevices on basement complex, 1643–2110 m. Vouchers: SAJIT–004551, 006407 & 006412 (EA, HIB), Gillett JB, Burtt BL & Osborn RM 17079, Archer PG 581 (EA).

***Habenariahumilior* Rchb.f.** – Habit: Herb. Habitat: Short grassland over rocks, ca. 1219 m. Voucher: Napier ER 1144 (EA).

***Habenariandiana* Rendle** – Habit: Herb. Habitat: In *Themeda*, *Hyparrhenia* grassland, with or without *Acacia* or *Combretum* scrub, ca. 1000 m. Voucher: Gregory s.n. (BM).

***Habenariaperistyloides* A.Rich.** – Habit: Herb. Habitat: Short upland grassland, open scrub, ca. 1850 m. Voucher: SAJIT–006369 (EA, HIB).

***Habenariathomsonii* Rchb.f.** – Habit: Herb. Habitat: Forest edge, among rocks, swampy grassland. Voucher: Bally PRO 13638 (EA).

***Microcoeliacorallina* Summerh.** – Habit: Herb. Habitat: Mixed woodland near river. Voucher: Luke WRQ & Luke PA 5572 (K).

***Microcoeliaexilis* Lindl.** – Habit: Herb. Habitat: Riverine forest, woodland, secondary forest, plantations, 670–945 m. Vouchers: Faden RB & Faden AJ 74/470, Faden RB, Evans A & Rathbun G 69/410, Archer PG 624 (EA).

***Microcoeliaglobulosa* (Hochst.) L.Jonss.** – Habit: Herb. Habitat: Margins of evergreen forest, relict rainforest forest, riverine forest, ca. 1180 m. Voucher: Mwachala G et al. EW245 (EA).

***Microcoeliaobovata* Summerh.** – Habit: Herb. Habitat: Woodland, wooded grassland, riverine forest, ca. 1097 m. Voucher: Archer PG 660 (EA).

**^NE^*Microcoeliasmithii* (Rolfe) Summerh.** – Habit: Herb. Habitat: Dry evergreen forest, ca. 540 m. Voucher: Bytebier B 424 (EA).

***Microcoeliastolzii* (Schltr.) Summerh.** – Habit: Herb. Habitat: Upland rainforest, dry evergreen and riverine forest, 627–1100 m. Vouchers: Miyawa D et al. NMK 961, Faden RB, Evans A, Msafiri F 70/979 (EA).

***Oberoniadisticha* (Lam.) Schltr.** – Habit: Herb. Habitat: Rainforest, dry evergreen mist forest, 700–1050 m. Voucher: Faden RB, Faden AJ & Smeenk C 71/960 (EA).

***Oeceocladesdecaryana* (H.Perrier) Garay & P.Taylor** – Habit: Herb. Habitat: Rocky outcrop, in leaf mold under bushes, 975–1006 m. Voucher: Archer PG 530 (EA).

***Platylepisglandulosa* (Lindl.) Rchb.f.** – Habit: Herb. Habitat: Rocks in forest, shady, marshy places on river banks, 1200–1300 m. Vouchers: Faden RB et al. 70/522a & 72/258 (EA).

**PolystachyaalbescensRidl.subsp.kraenzlinii (Rolfe) Summerh.** – Habit: Herb. Habitat: Mist forest, 1219–2100 m. Voucher: Archer PG 569 (EA).

**PolystachyacaespitificaKraenzl.subsp.latilabris (Summerh.) P.J.Cribb & Podz.** – Habit: Herb. Habitat: Montane forest, 1490–2100 m. Vouchers: Faden RB, Evans A, Msafiri F & Smeenk C 71/57A, Faden RB et al. 70/776, Friis I & Hansen OJ 2678 (EA). Vulnerable.

***Polystachyaconcreta* (Jacq.) Garay & H.R.Sweet** – Habit: Herb. Habitat: Forest epiphyte, ca. 1097 m. Voucher: Archer PG 601 (EA).

***Polystachyacultriformis* (Thouars) Lindl. ex Spreng.** – Habit: Herb. Habitat: Rainforest, 1425–2100 m. Vouchers: SAJIT–005328 (EA), Faden RB et al. NMK Exped. 794 & 1175, Bytebier B 1180, 1323 & 1516, Drummond RB & Hemsley JH 4388, Polhill RM & Verdcourt B 2735, Gillett JB, Burtt BL, Osborn RM 17100, Bally PRO 8793 (EA).

***Polystachyadendrobiiflora* Rchb.f.** – Habit: Herb. Habitat: Growing on *Vellozia* and on rocks, *Philippia* bushland, 609–1700 m. Vouchers: SAJIT–005341, Polhill RM & Kibuwa SP 949, Faden RB et al. 71/214, van Someren C 66, Gardner HM 2904, Joanna B 8901, Bally PRO 13373, Uhlig C 1a (EA).

***Polystachyadisiformis* P.J.Cribb** – Habit: Herb. Habitat: Montane forest, 1400–1450 m. Vouchers: Luke WRQ & Luke PA 5568, Bytebier B 1155, Drummond RB & Hemsley JH 4386 (EA). Endangered.

***Polystachyagolungensis* Rchb.f.** – Habit: Herb. Habitat: Evergreen, riverine forest. Voucher: None. Recorded in *Orchids of Kenya* (Stewart and Campbell 1996).

***Polystachyalindblomii* Schltr.** – Habit: Herb. Habitat: Dense forests and forest margins, 1350–1470 m. Vouchers: Bytebier B 1158, Seki T 85, Faden RB et al. 70/777 (EA).

***Polystachyamodesta* Rchb.f.** – Habit: Herb. Habitat: Riverine forest, woodland, bushland, wooded grassland, 500–700 m. Voucher: Archer PG 601 (EA, K).

***Polystachyapolychaete* Kraenzl.** – Habit: Herb. Habitat: Rainforest, ca. 1097 m. Voucher: Archer PG 599 (EA).

***Polystachyaspatella* Kraenzl.** – Habit: Herb. Habitat: Montane forest, drier woodland, 1700–1875 m. Voucher: Faden RB et al. NMK Exped. 195 (EA).

**^E^*Polystachyateitensis* P.J.Cribb** – Habit: Herb. Habitat: Mist forest, 800–975 m. Vouchers: Luke WRQ & Luke PA 9438A, Faden RB, Evans A & Githui M 70/166, Archer PG 612, Stewart J 1347 (EA). Endangered.

***Polystachyatransvaalensis* Schltr.** – Habit: Herb. Habitat: Upland rainforest and mist forest, 1425–2195 m. Vouchers: Bytebier B 1515, Faden RB et al. NMK Exped. 159 & 947, Verdcourt B & Polhill RM 2724, Friis I & Hansen OJ 2677, Faden RB & Faden AJ 72/254, Faden RB et al. 70/774 (EA).

***Porpaxrepens* (Rolfe) Schuit., Y.P.Ng & H.A.Pedersen var. repens** [*Stolziarepens* (Rolfe) Summerh.] – Habit: Herb. Habitat: Mossy trunks and branches in forest, 1425–1850 m. Vouchers: SAJIT–004262 (EA, HIB), Beentje HJ et al. NMK Exped. 1020, Faden RB et al. 71/57b (EA).

^**NE**^***Rhipidoglossumleedalii* (P.J.Cribb) Farminhão & Stévart** [*Margellianthaleedalii* P.J. Cribb] – Habit: Herb. Habitat: Montane or mist forest on mossy branches, ca. 1524 m. Voucher: Archer PG 548 (EA).

***Rhipidoglossumrutilum* (Rchb.f.) Schltr.** – Habit: Herb. Habitat: Montane and riverine forest, 820–1000 m. Voucher: Faden RB et al. s.n. (EA).

***Rhipidoglossumxanthopollinium* (Rchb.f.) Schltr.** – Habit: Herb. Habitat: Lower areas of montane forest, ca. 884 m. Voucher: Archer PG 608 (EA).

***Satyriumcrassicaule* Rendle** – Habit: Herb. Habitat: Montane forest glades, near streams, 1360–1573 m. Vouchers: Miyawa D et al. NMK 970, Mwachala G et al. EW3601 (EA).

***Solenangiswakefieldii* (Rolfe) P.J.Cribb & J.Stewart** – Habit: Herb. Habitat: Lowland bushland, ca. 945 m. Voucher: Archer PG 625 (EA).

***Sphyrarhynchusbrevilobus* (Summerh.) Bytebier** [*Angraecopsisbreviloba* Summerh.] – Habit: Herb. Habitat: Upland forest, light forest on hills, 807–1332 m. Vouchers: Miyawa D et al. NMK 962, Archer PG 631 (EA).

***Tridactyleanthomaniaca* (Rchb.f.) Summerh.** – Habit: Herb. Habitat: Riverine and lower montane forest, ca. 914 m. Voucher: Archer PG 715 (EA).

***Tridactylebicaudata* (Lindl.) Schltr.** – Habit: Herb. Habitat: Lowland to montane forest, riverine forest, ca. 1400 m. Voucher: Bally J 8863 (EA).

^**NE**^***Tridactylecruciformis* Summerh.** – Habit: Herb. Habitat: Upland rainforest, 1440–1680 m. Vouchers: SAJIT–003294 (EA, HIB), Faden RB, Evans A, Msafiri F & Smeenk C 71/46, Faden RB et al. 70/568, Bytebier B 1137, 1146, 1152 & 1198 (EA). Endangered.

***Tridactylefilifolia* (Schltr.) Schltr.** – Habit: Herb. Habitat: Upland rainforest, 1873 m. Voucher: SAJIT–004185 (EA, HIB).

***Vanillaroscheri* Rchb.f.** – Habit: Herb. Habitat: Open evergreen scrub, 550–700 m. Vouchers: Hucks M 456, Jex-Blake M 6872 (EA).

***Ypsilopusamaniensis* (Kraenzl.) D’haijère & Stévart** – Habit: Herb. Habitat: *Juniperusprocera* montane forest, open bushland, ca. 1829 m. Vouchers: SAJIT-004125 (EA, HIB), Joanna B 9052 (EA).

**^NE^*Ypsilopustanneri* (P.J.Cribb) D’haijère & Stévart** [*Tridactyletanneri* P.J. Cribb] – Habit: Herb. Habitat: Montane evergreen forest, 1480–1641 m. Vouchers: Luke WRQ & Luke PA 4159, Bytebier B 1143, Faden RB, Faden AJ, Smeenk C, Smeenk N & McNee J 71/971, Archer PG 580 & 595 (EA). Endangered.

**F37. POACEAE**

25 Genera, 47 species

***Andropogonheterantherus* Stapf** – Habit: Herb. Habitat: Deciduous woodland and scrub, 800–950 m. Voucher: Kabuye CHS et al. NMK Exped. 674 (EA).

***Andropogonschirensis* Hochst. ex A.Rich.** – Habit: Herb. Habitat: Deciduous bushland, wooded grassland, 1425–1850 m. Voucher: Beentje HJ et al. NMK Exped. 1194 (EA).

***Aristidaadscensionis* L.** – Habit: Herb. Habitat: Dry soils and waste ground, 548–945 m. Vouchers: Kirika P et al. EW306 & 921, Kirika P et al. 2188 & 8648B (EA).

***Aristidastenostachya* Clayton** – Habit: Herb. Habitat: Deciduous thicket and bushland, 485–600 m. Vouchers: Luke WRQ & Luke PA 16115, Leuthold W 122 (EA).

***Cenchrusciliaris* L.** – Habit: Herb. Habitat: Deciduous bushland, wooded grassland ca. 500–700 m. Vouchers: Muasya AM & Archibald S 2077, Bally PRO 8645, Greenway PJ 12631 (EA).

***Cenchrusmassaicus* (Stapf) Morrone** – Habit: Herb. Habitat: Deciduous bushland on alluvial black soils, ca. 548 m. Voucher: Bogdan A 3614 (EA).

***Cenchrustrachyphyllus* (Pilg.) Morrone** – Habit: Herb. Habitat: Glades, semi-shaded and wet sites in rainforest, pathsides, 1550–2000 m. Vouchers: Beentje HJ et al. NMK Exped. 890 & 941 (EA).

***Chlorisgayana* Kunth** – Habit: Herb. Habitat: Riverine woodland, scattered tree grassland, open grassland. Voucher: Rauh W Ke951 (EA).

*Coixlacryma-jobi L. – Habit: Herb. Habitat: Stream sides and swampy places, occasionally cultivated, 579–1350 m. Vouchers: Mwachala G et al. EW1244, Kabuye CHS 82/127, Faden RB, Evans A, Glover PE 70/459, Napier ER 998, McDonald J 864 (EA).

***Digitariaabyssinica* (Hochst. ex A.Rich.) Stapf** – Habit: Herb. Habitat: Ruderal sites, grazed lands, weed of cultivated areas, 1524–1646 m. Vouchers: Mrs. Robertson SA 2179, Frowd BH 2 (EA).

***Digitariaaridicola* Napper** – Habit: Herb. Habitat: Dry open deciduous bushland, ca. 600 m. Voucher: Leuthhold W 123 (EA).

***Digitariapearsonii* Stapf** – Habit: Herb. Habitat: Forest margins and open weedy places, 1400–1640 m. Vouchers: Faden RB et al. NMK Exped. 19, Mrs. Robertson SA 2182 (EA).

***Digitariapennata* (Hochst.) T.Cooke** – Habit: Herb. Habitat: Dry open deciduous bushland, ca. 487 m. Voucher: Archer PG 627 (EA).

***Digitariarivae* (Chiov.) Stapf** – Habit: Herb. Habitat: *Acacia*-*Terminalia*-*Commiphora* open bushland, 457–609 m. Greenway PJ & Kanuri K 12779 & 12796, Leuthold W 113, Sheldrick D NP21, Bogdan A 3902, FitzGerald V 4971 (EA).

***Ehrhartaerecta* Lam.** – Habit: Herb. Habitat: Forest glades, margins and pathsides, 1700–1875 m. Voucher: Faden RB et al. NMK Exped. 572 (EA).

***Eleusineafricana* Kenn.-O’Byrne** – Habit: Herb. Habitat: Disturbed ground, along roadsides, old cultivations, ca. 1000 m. Voucher: Mungai GM et al. EW2835 (EA).

***Enteropogonmacrostachyus* (Hochst. ex A.Rich.) Munro ex Benth.** – Habit: Herb. Habitat: Disturbed places, *Acacia*-*Commiphora* bushland, 600–950 m. Vouchers: Mwachala G et al. EW71 & 860, Kabuye CHS et al. NMK Exped. 683, Bally PRO 8648, Brown E 2139G, Frowd BH 11 (EA).

***Enteropogonrupestris* (J.A.Schmidt) A.Chev.** – Habit: Herb. Habitat: Scattered tree grassland on black clay, ca. 548 m. Voucher: Bogdan A 3613 (EA).

***Eragrostisciliaris* (L.) R.Br.** – Habit: Herb. Habitat: Farmlands, clearings, overgrazed areas, trodden weedy places, 800–950 m. Voucher: Kabuye CHS et al. NMK Exped. 703 (EA).

***Heteropogoncontortus* (L.) P.Beauv. ex Roem. & Schult.** – Habit: Herb. Habitat: Wooded grassland and deciduous bushland, ca. 1600 m. Voucher: Beentje HJ et al. NMK Exped. 1189 (EA).

***Hyparrheniacymbaria* (L.) Stapf** – Habit: Herb. Habitat: Open hillsides bordering upland evergreen forest, 1740–2000 m. Voucher: Beenje HJ et al. NMK Exped. 939 (EA).

***Isachnemauritiana* Kunth** – Habit: Herb. Habitat: In forest shade, 1500–1800 m. Vouchers: Beentje HJ 2131, Beentje HJ et al. NMK Exped. 1105, Bytebier B 1220, Bally PRO 8704 (EA).

***Megathyrsusinfestus* (Andersson) B.K.Simon & S.W.L.Jacobs** – Habit: Herb. Habitat: Deciduous woodland, bushland, grassland, ca. 550–600 m. Voucher: Greenway PJ & Kanuri K 12726 (EA).

***Megathyrsusmaximus* (Jacq.) B.K.Simon & S.W.L.Jacobs** – Habit: Herb. Habitat: Deciduous bushland, riverbanks and damp sites, roadsides, cultivations, 518–1010 m. Vouchers: Mwachala G et al. EW51, 70, 342 & 950, Vesey-Fitzgerald LD 4963, Mrs. Robertson SA 2198, Greenway PJ & Kanuri K 12784 (EA).

***Melinisminutiflora* P.Beauv.** – Habit: Herb. Habitat: Open hillsides, forest edges, 1425–1850 m. Vouchers: Beentje HJ et al. NMK Exped. 1179, Bally PRO 8581 (EA).

***Melinisrepens* (Willd.) Zizka** – Habit: Herb. Habitat: Weed of disturbed places and old farmlands. Voucher: Greenway PJ & Kanuri K 12972 (EA).

***Microchloakunthii* Desv.** – Habit: Herb. Habitat: Open places with bare and shallow soil in bushland or grassland, 1040–1100 m. Voucher: Mwachala G et al. EW238 (EA).

***Oplismenusundulatifolius* (Ard.) P.Beauv.** – Habit: Herb. Habitat: In forest shade, 1700–1875 m. Voucher: Faden RB et al. NMK Exped. 231 (EA).

***Panicumcalvum* Stapf** – Habit: Herb. Habitat: Forest shade and margins, 1040–2170 m. Vouchers: Mwachala G et al. EW219, Faden RB et al. NMK Exped. 102 (EA).

***Panicumhirtum* Lam.** – Habit: Herb. Habitat: Deciduous bushland, wooded grassland on poor sandy soils, 610–980 m. Vouchers: Mungai GM et al. EW2653, Napper DM 1965 (EA).

***Panicumhochstetteri* Steud.** – Habit: Herb. Habitat: Forest shade, ca. 1400 m. Voucher: Wakanene KM & Mwangangi OM 659 (EA).

***Panicumtrichocladum* Hack. ex K.Schum.** – Habit: Herb. Habitat: Bush or forest edge on sandy soils, 1400–1850 m. Vouchers: Faden RB et al. NMK Exped. 17 & 441 (EA).

***Pseudechinolaenapolystachya* (Kunth) Stapf** – Habit: Herb. Habitat: Forest shade and disturbed sites, 1425–1875 m. Vouchers: Faden RB et al. NMK Exped. 351 & 442 (EA).

***Pseudobromusafricanus* (Hack.) Stapf** – Habit: Herb. Habitat: Forest floor grass near forest margin, 1700–1875 m. Vouchers: Faden RB et al. NMK Exped. 234 & 558 (EA).

*Setariasulcata Raddi – Habit: Herb. Habitat: Forest and woodland shades, 1700–1875 m. Vouchers: Beentje HJ 2132, Faden RB et al. NMK Exped. 574, Bally PRO 13618 (EA).

***Sporobolusnatalensis* (Steud.) T.Durand & Schinz** – Habit: Herb. Habitat: Forest margins, roadsides, disturbed and grazed places, 1700–1875 m. Voucher: Faden RB et al. NMK Exped. 265 (EA).

***Tetrapogonbidentatus* Pilg.** – Habit: Herb. Habitat: Deciduous bushland, grassland, 549–853 m. Vouchers: Bogdan A 3903, Greenway PJ Kanuri K 12638 (EA).

***Tetrapogonroxburghiana* (Schult.) P.M.Peterson** – Habit: Herb. Habitat: Wooded grassland, bushland and disturbed habitats, on dry or stony sandy soils, 548–945 m. Vouchers: Trapnell CG 2226, Frowd BH F13, MacDonald J 850, Graham MD 5, Mrs. Robertson SA 2186 (EA).

***Themedatriandra* Forssk.** – Habit: Herb. Habitat: Open deciduous bushland, 1040–1100 m. Voucher: Mwachala G et al. EW349 (EA).

***Urochloabrizantha* (A.Rich.) R.D.Webster** – Habit: Herb. Habitat: Deciduous woodland, wooded grassland, upland grassland, ca. 1780 m. Voucher: Wakanene KM & Mwangangi OM 748 (EA).

***Urochloadeflexa* (Schumach.) H.Scholz** – Habit: Herb. Habitat: Margins of riverine forest, deciduous bushland, weedy places, 600–823 m. Vouchers: Bally PRO 8646, Polhill RM & Kibuwa SP 963 (EA).

***Urochloadictyoneura* (Fig. & De Not.) Veldcamp** – Habit: Herb. Habitat: Wooded grassland, deciduous bushland, ca. 998 m. Voucher: Mwachala G et al. EW301 (EA).

***Urochloadistachya* (L.) T.Q.Nguyen** – Habit: Herb. Habitat: Shrubland, grassland, weedy places. Voucher: Greiling 48 (EA).

***Urochloalachnantha* (Hochst.) A.M.Torres & C.M.Morton** – Habit: Herb. Habitat: Damp soils, ca. 487 m. Voucher: Greenway PJ & Kanuri K 12717 (EA).

***Urochloaleersioides* (Hochst.) A.M.Torres & C.M.Morton** – Habit: Herb. Habitat: Path sides, weedy places, old farmland, 600–1300 m. Vouchers: Muasya AM & Archibald S 2072, Christiaensen AR 205, Bally PRO 8643, Ciba-Geigy 2, Mrs. Robertson SA 2192, Hitchcock AS 24681 (EA).

***Urochloanigropedata* (Munro ex Ficalho & Hiern) A.M.Torres & C.M.Morton** – Habit: Herb. Habitat: Dry rocky and sandy soils in deciduous bushland, wooded grassland. Voucher: Rauh W Ke957 (EA).

**Urochloaxantholeuca(Hack.)H.Scholzvar.leucacrantha (K.Schum.) Sosef** – Habit: Herb. Habitat: Deciduous bushland, sandy soils in disturbed places, 550–700 m. Vouchers: Leuthold W 121, Vesey-FitzGerald LDEF 4966 (EA).

**F38. SMILACACEAE**

1 Genus, 1 species

***Smilaxanceps* Willd.** – Habit: Liana. Habitat: Forest edges, clearings and secondary associations of bushland, 1700–1875 m. Voucher: Gardner HM 1207 (EA).

**F39. VELLOZIACEAE**

1 Genus, 1 species

***Xerophytaspekei* Baker** – Habit: Herb/Shrub/Tree. Habitat: Rocky slopes and outcrops, 600–1000 m. Vouchers: Faden RB & Faden AJ 74/449, Gardner HM 2924, Medley KE p.r. (EA).

**F40. ZINGIBERACEAE**

1 Genus, 1 species

***Aframomumangustifolium* (Sonn.) K.Schum** – Habit: Herb. Habitat: Riverine forest, swampy places, 1260–1450 m. Vouchers: Kabuye CHS et al. NMK Exped. 801, Bally PRO 8799, Faden RB et al. 71/1001 (EA).

### ﻿Part 6. Magnoliopsida

**F41. ACANTHACEAE**

20 Genera, 37 species

***Barleriaacanthoides* Vahl** – Habit: Herb. Habitat: Roadside vegetation in savanna with many tall *Euphorbia* trees, 1400–1800 m. Voucher: Christenhusz MJM, Kamau P, Chase MW, Mbale M & Kyaa J 6677 (EA).

**BarleriaeranthemoidesR.Br. ex C.B.Clarkevar.eranthemoides** – Habit: Subshrub. Habitat: Open dry bushland, grassland on rocky slopes, disturbed and grazed areas, 800–1000 m. Vouchers: Watuma BM W0005 (EA, HIB), Kabuye CHS et al. NMK Exped. 746, Medley KE 507 (EA).

**BarleriaeranthemoidesR.Br. ex C.B.Clarkevar.agnewii I.Darbysh.** – Habit: Subshrub. Habitat: Open and dense dry bushland, grassland, degraded areas, ca. 823 m. Voucher: Polhill RM & Kibuwa SP 961 (EA).

***Barleriaprionitis* L.** – Habit: Subshrub. Habitat: Open *Euphorbia*-*Acacia*-*Commiphora* forest, rocky outcrops, 650–1000 m. Voucher: Medley KE 868 (EA).

***Barleriasubmollis* Lindau** – Habit: Subshrub. Habitat: Forest edges, bushland, roadside, 800–1350 m. Vouchers: Kabuye CHS et al. NMK Exped. 605, Mungai GM et al. EW3576 (EA).

***Barleriataitensis* S.Moore** – Habit: Subshrub. Habitat: *Terminalia*-*Combretum* woodland, *Acacia*-*Commiphora* bushland, rocky hillsides and sandy soils, 640–1206 m. Vouchers: Watuma BM W0013 (EA, HIB), Wakanene KM & Mwangangi OM 512, Polhill RM & Paulo S 935, Hucks M 894, Hildebrandt JM 2457 (EA).

***Barleriavolkensii* Lindau** – Habit: Subshrub. Habitat: *Acacia* bushland, drier parts of montane forest, ca. 1220 m. Voucher: Napier ER 1080 (EA).

***Blepharismaderaspatensis* (L.) B.Heyne ex Roth** – Habit: Herb. Habitat: Disturbed and secondary vegetation, woodland and forest habitats, 800–950 m. Voucher: Kabuye CHS et al. NMK Exped. 652 (EA).

***Brillantaisiacicatricosa* Lindau** – Habit: Subshrub. Habitat: Mist forest margins, ca. 1557 m. Voucher: Watuma BM W0214 (EA, HIB).

***Crabbeavelutina* S.Moore** – Habit: Herb. Habitat: Riverine vegetation, 800–950 m. Voucher: Kabuye CHS et al. NMK Exped. 715 (EA).

***Crossandrafriesiorum* Mildbr.** – Habit: Herb. Habitat: Moist and dry evergreen mist forest, 820–1850 m. Vouchers: Beentje HJ et al. NMK Exped. 996, Faden RB, Evans A, Githui M 70/173 (EA). Vulnerable.

***Crossandramucronata* Lindau** – Habit: Subshrub. Habitat: *Acacia*-*Commiphora* woodland and bushland on sandy soils and rocky outcrops, 609–750 m. Vouchers: Polhill RM & Kibuwa SP 942, Napier ER 890, Gillett JB 17223, McDonald JM 878 (EA).

***Crossandratridentata* Lindau** – Habit: Subshrub. Habitat: Undergrowth in upland rainforest, 1400–2135 m. Vouchers: SAJIT–003293, Watuma BM W0022 & W0053 (EA, HIB), Wakanene KM & Mwangangi OM 262, 393 & 688, De Block P et al. 256, 257 & 259, Faden RB et al. NMK Exped. 191, Faden RB et al. 69/860, Bally PRO 8753 & 13519 (EA), Joanna B 9067 (EA).

**^NE^*Dyschoristekeniensis* Malombe, Mwachala & Vollesen subsp. glandulifera Malombe, Mwachala & Vollesen** – Habit: Subshrub. Habitat: *Combretum*, *Acacia* & *Terminalia* woodland, wooded grassland and bushland, old cultivation, 800–1370 m. Vouchers: Watuma BM W0156 (EA, HIB), Malombe I et al. EW4004, 4013, 4041, 4047 & 4065, Mwachala G et al. EW756 & 846, Luke WRQ et al. 5389, Verdcourt B & Polhill RM 2738, Drummond RB & Hemsley JH 4409, Napier ER 1089 (EA). Vulnerable.

**EcboliumsubcordatumC.B.Clarkevar.glabratum Vollesen** – Habit: Herb. Habitat: *Acacia*-*Commiphora* bushland, riverbanks and riverbeds, 500–1000 m. Vouchers: SAJIT–005392 (EA, HIB), Napier ER 960 (EA).

***Hypoestesaristata* (Vahl) Sol. ex Roem. & Schult.** – Habit: Herb. Habitat: Forest margins and clearings, 609–1850 m. Vouchers: Kabuye CHS et al. NMK Exped. 591, Beentje HJ et al. NMK Exped. 446, Hildebrandt JM 2563, Johnston HH s.n. (EA).

^**NE**^***Isoglossacandelabrum* Lindau** – Habit: Shrub. Habitat: Moist lowland and mid-altitude forest, 1220–1350 m. Vouchers: Luke WRQ & Luke PA 4137, Hemp A 5375, Archer PG 570 (EA). Endangered.

***Isoglossagregorii* (S.Moore) Lindau** – Habit: Herb. Habitat: Forest margins and clearings, ericaceous bushland, 1800–2170 m. Vouchers: Faden RB et al. NMK Exped. 112, Drummond RB & Hemsley JH 4311, Gillett JB, Burtt BL & Osborn RM 17098 (EA).

**IsoglossalacteaLindau ex Engl.subsp.saccata I.Darbysh.** – Habit: Herb. Habitat: Undergrowth and margins of moist forest, 1200–1980 m. Vouchers: Luke WRQ et al 4129, Beentje HJ et al. NMK Exped. 871, Drummond RB & Hemsley JH 4373, Bally PRO 8771, Napier ER 1118 (EA).

***Isoglossalaxa* Oliv.** – Habit: Subshrub. Habitat: Montane forest understorey, clearances and secondary growth, 1700–1875 m. Voucher: Faden RB et al. NMK Exped. 349 (EA).

***Isoglossapunctata* (Vahl) Brummitt & J.R.I.Wood** – Habit: Herb. Habitat: Montane forest undergrowth, pathways and margins, ca. 1728 m. Voucher: SAJIT–006355 (EA, HIB).

***Isoglossasubstrobilina* C.B.Clarke** – Habit: Subshrub. Habitat: Montane forest undergrowth, glades, margins and disturbed places, 1700–1875 m. Voucher: Faden RB et al. NMK Exped. 298 (EA).

***Justiciaflava* (Forssk.) Vahl** – Habit: Shrub. Habitat: Forest margins and clearings, ca. 609 m. Voucher: Johnston HH s.n. (EA).

***Justiciagaleata* Hedrén** – Habit: Shrub. Habitat: Semi-deciduous lowland forest, *Acacia*-*Commiphora* bushland, 880–1200 m. Vouchers: Mwachala G et al EW984 & 1449, Luke WRQ 5515 & 6236 (EA). Vulnerable.

***Justiciaheterocarpa* T.Anderson** – Habit: Herb. Habitat: *Acacia*-*Commiphora* bushland, forest margins, roadsides, ca. 880 m. Vouchers: Mwachala G et al. EW959, Verdcourt B 5316 (EA).

***Justiciapseudorungia* Lindau** – Habit: Shrub. Habitat: Montane evergreen forest, riverine forest, 1300–1875 m. Vouchers: Faden RB et al. NMK Exped. 530, Faden RB et al. 71/260 (EA).

***Lepidagathisscariosa* Nees** – Habit: Subshrub. Habitat: Dry *Acacia*-*Commiphora*-*Grewia*-*Combretum*-*Euphorbia* bushland and woodland, 600–720 m. Vouchers: Medley KE 993, Mrs Robertson SA 6536 (EA).

***Monechmadebile* (Forssk.) Nees** – Habit: Herb. Habitat: Disturbed forest, *Acacia*-*Commiphora* bushland, roadsides, weed of cultivation, 609–1602 m. Vouchers: Watuma BM W0275 (EA, HIB), Kabuye CHS et al. NMK Exped. 625, Mwachala G et al. EW916, Hilderbrandt JM 2397, Johnston HH s.n. (EA).

**NeuracanthustephrophyllusBidgood & Brummittsubsp.tsavoensis Bidgood & Brummitt** – Habit: Subshrub. Habitat: *Acacia* and *Acacia*-*Commiphora* bushland, ca. 590 m. Voucher: Faden RB & Faden AJ 74/519 (EA). Endangered.

***Nicotebabetonica* (L.) Lindau** – Habit: Herb. Habitat: Evergreen forest clearings, disturbed areas ca. 1584 m. Voucher: Watuma BM W0135 (EA, HIB).

***Phaulopsisimbricata* (Forssk.) Sweet** – Habit: Herb. Habitat: Montane forest edges, secondary scrub, clearings and disturbed places, 1400–1850 m. Vouchers: Mungai GM et al. EW3197, Beentje HJ et al. NMK Exped. 999, Drummond RB & Hemsley JH 4381 (EA).

***Pseuderanthemumhildebrandtii* (C.B.Clarke) Lindau** – Habit: Subshrub. Habitat: Riverine forest, thicket and bushland, 800–950 m. Voucher: Kabuye CHS et al. NMK Exped. 733 (EA).

***Rhinacanthusangulicaulis* I.Darbysh.** – Habit: Herb. Habitat: Dense or open *Acacia*-*Commiphora* bushland, dry semi-evergreen forest, 900–1250 m. Voucher: SAJIT–005376 (EA, HIB).

***Ruelliaprostrata* Poir.** – Habit: Herb. Habitat: Woodland, bushland and grassland, ca. 800 m. Voucher: Mwachala G et al. EW3301 (EA).

***Ruttyafruticosa* Lindau** – Habit: Shrub. Habitat: Disturbed bushland, woodland, 620–650 m. Vouchers: Medley KE 548, 551 & 711 (EA).

***Thunbergiaalata* Bojer ex Sims** – Habit: Climber. Habitat: Wet forest margins, *Acacia*-*Commiphora* bushland, disturbed places, 1100–1925 m. Vouchers: Wakanene KM & Mwangangi OM 724, Faden RB & Evans A 69/856, 857 & 887, Bally PRO 13506 & 14203, Faden RB et al. NMK Exped. 255, Kluguess LM 25 (EA).

***Thunbergiaguerkeana* Lindau** – Habit: Climber. Habitat: *Acacia*-*Commiphora* bushland, 450–550 m. Vouchers: Medley KEM 980 (EA), Verdcourt B 1112, Holm K & Holm L 10, Dale IR K2048, Gillett JB 17214, Scott Elliott GF 6166 (EA).

***Thunbergiaschimbensis* S.Moore** – Habit: Herb. Habitat: *Acacia*-*Commiphora*, *Combretum*-*Terminalia* woodland, bushland, ca. 890 m. Voucher: Faden RB, Faden AJ & Smeenk C 71/988 (EA).

**F42. ACHARIACEAE**

2 Genera, 2 species

***Dasylepisintegra* Warb.** – Habit: Tree. Habitat: Understorey in rainforest, 1400–1925 m. Vouchers: SAJIT–003291 & 006356 (EA, HIB), Mwachala G et al. EW1445, Faden RB & Evans A 69/879, Faden RB et al. 71/19, Faden RB et al. NMK Exped. 242 & 1050, Medley KE 1006a, Drummond RB & Hemsley JH 4341, Gardner HM 1212 & 2937, Gardiner N 2949, Joanna B 8918 & 8759, Raler I 0011 (EA).

***Rawsonialucida* Harv. & Sond.** – Habit: Tree. Habitat: Understorey and shrub layer in upland rainforest, 1000–1750 m. Vouchers: Luke WRQ & Luke PA 4100 & 5489, Faden RB, Evans A & Rathbun G 69/479, Medley KE 1015 (EA).

**F43. AIZOACEAE**

2 Genera, 3 species

***Trianthemaceratosepalum* Volkens & Irmsch.** – Habit: Subshrub. Habitat: *Acacia* bushland on alkaline soils, ca. 600 m. Voucher: Hucks M 440 (EA).

***Trianthemaportulacastrum* L.** – Habit: Herb. Habitat: Weed of cultivation, disturbed places, sandy soils, 600–900 m. Vouchers: Hucks M 729, Mrs Robertson SA 3612 & 3680 (EA).

***Zaleyapentandra* (L.) C.Jeffrey** – Habit: Herb. Habitat: Open woodland and bushland, cultivation and roadside weed, 480–900 m. Vouchers: Napier ER 1000, Hildebrandt JM 2400, Mrs Robertson SA 3673, Leuthold W 109 (EA).

**F44. ALISMATACEAE**

1 Genus, 1 species

***Alismaplantago*-*aquatica* L.** – Habit: Herb. Habitat: Swampy places, water drainage, ca. 1200 m. Voucher: Lubai IK HB14 (EA).

**F45. AMARANTHACEAE**

15 Genera, 25 species

**AchyranthesasperaL.var.aspera** – Habit: Herb. Habitat: Open areas in mist forest patches, along paths, weed of cultivation or disturbed ground, 600–1957 m. Vouchers: Watuma BM W0266 (EA, HIB), Hucks M 885 & 1050 (EA).

**AchyranthesasperaL.var.sicula L.** – Habit: Herb. Habitat: Montane forest edges, 1450–1780 m. Vouchers: Wakanene KM, Mwangangi OM & Dunn B 300, 387 & 744, Bally JB 13613 (EA).

*Alternantherapungens Kunth – Habit: Herb. Habitat: Weed in trodden places, along tracks and roadsides, 500–950 m. Vouchers: Mwachala G et al. in EW502, Mrs Robertson SA 3678, Bally PRO B13249a, Robel B 125, Geigy Ciba 10, Ivens GW 2611 (EA).

*Alternantherasessilis (L.) R. Br. ex DC. – Habit: Herb. Habitat: Along river, damp forests, ca. 1372 m. Voucher: Kabuye CHS 82/42 (EA).

*AmaranthusgraecizansL.subsp.thellungianus (Nevski) Gusev – Habit: Herb. Habitat: Weed in disturbed and grazed lands, 550–610 m. Voucher: Napier ER 911 (EA).

*AmaranthushybridusL.subsp.hybridus – Habit: Herb. Habitat: Cultivations and ruderal lands, roadsides and forest edges, 800–950 m. Voucher: Kabuye CHS et al. NMK Exped. 620 (EA).

***Celosiaanthelminthica* Asch.** – Habit: Herb. Habitat: Bushes on forest edges, roadside, 800–1875 m. Vouchers: Watuma BM W0258 (EA, HIB), Kabuye CHS et al. NMK Exped. 604, Faden RB et al. NMK Exped. 534, Hucks M 738 (EA).

***Celosiahastata* Lopr.** – Habit: Herb. Habitat: Forest edges, 900–1875 m. Vouchers: Kabuye CHS 82/69, Wakanene KM & Mwangangi OM 232, Faden RB et al. NMK Exped. 352, Beentje HJ et al. NMK Exped. 402, Faden RB, Evans A 71/119 (EA).

***Celosiaschweinfurthiana* Schinz** – Habit: Herb. Habitat: Forest clearings, rocky scrubland, wooded grassland, 1150–1250 m. Vouchers: Mungai G et al. EW1409, Luke WRQ et al 2 (EA).

***Celosiatrigyna* L.** – Habit: Herb. Habitat: Roadsides, short grassland, weed of cultivation, 610–1580 m. Vouchers: Kabuye CHS 82/111, Napier ER 995 (EA).

***Centemopsiskirkii* (Hook.f.) Schinz** – Habit: Herb. Habitat: Deciduous woodland and bushland, waste ground, roadsides. Vouchers: Bally PRO 8657, Hittu 45 (EA).

***Chenopodiastrumfasciculosum* Aellen** – Habit: Herb. Habitat: Weed of forest margins, roadside, waste places, ca. 1700 m. Voucher: Gillett JB, Burtt BL, Osborn RM 17073 (EA).

***Cyathulacylindrica* Moq.** – Habit: Herb. Habitat: Mist forest, riverside, woodland, open bushland and rocky places, 998–2170 m. Vouchers: Mwachala G et al. EW48 (EA), Wakanene KM & Mwangangi OM 720, Faden RB et al. NMK Exped. 130 & 432, Klungness LM 60 (EA).

***Cyathulauncinulata* (Schrad.) Schinz** – Habit: Herb. Habitat: Dense forest, open bush, short grassland, disturbed places, near water, 1200–1780 m. Vouchers: Wakanene KM & Mwangangi OM 751, Napier ER 1115, Gillett JB 23890 (EA).

**Digeramuricata(L.)Mart.var.macroptera C.C.Towns.** – Habit: Herb. Habitat: *Acacia*-*Commiphora* bushland, 700–950 m. Vouchers: Kabuye CHS et al. NMK Exped. 736, Faden RB & Faden AJ 74/265 (EA).

**Digeramuricata(L.)Mart.subsp.trinervis C.C.Towns.** – Habit: Herb. Habitat: *Acacia*-*Commiphora* bushland, dry seasonal beds, ca. 900 m. Vouchers: Gardner H 2988, MacDonald J 869, Bally PRO 8803, ERV 906, Mrs Robertson SA 3674 (EA).

*Gomphrenacelosioides Mart. – Habit: Herb. Habitat: Trodden paths, roadsides, weed of cultivation, 520–1100 m. Vouchers: Mwachala G et al. EW912, Mungai GM et al. EW2837, Faden RB & Faden AJ 74/475, Greenway PJ & Kanuri K 12783, Hucks M 1023 (EA).

*Guillemineadensa (Willd. Ex Schult.) Moq. – Habit: Herb. Habitat: Trodden and waste ground, roadside, 800–950 m. Voucher: Kabuye CHS et al. NMK Exped. 682 (EA).

***Ouretlanata* (L.) Kuntze** – Habit: Herb. Habitat: Weed in open habitats, 800–1450 m. Vouchers: Mwachala et al. EW943, Wakanene KM & Mwangangi OM 294, Msafiri F & Ojiambo L 326 (EA).

***Psilotrichumcyathuloides* Suess. & Launert** – Habit: Herb. Habitat: Open grassy areas in forest, disturbed places. Voucher: Luke WRQ & Luke PA 4233 (EA).

***Psilotrichumelliotii* Baker** – Habit: Herb. Habitat: Semi-evergreen bushland and thicket, 960–1873 m. Vouchers: SAJIT–004562 (EA, HIB), Kabuye CHS 82/152, Luke WRQ & Luke PA 5520, Gillett JB 19568 (EA).

***Psilotrichumscleranthum* Thwaites** – Habit: Subshrub. Habitat: Riverine forest and scrub, secondary vegetation, roadsides, 650–1050 m. Vouchers: Kabuye CHS et al. NMK Exped. 632, Mwachala G et al. EW3296, Faden RB & Faden AJ 74/488, Medley KE 563, 690 & 756, Bally PRO 13587, Gilbert MG 6109 (EA).

***Psilotrichummajus* Peter** – Habit: Subshrub. Habitat: Forest floor and margins, ca. 1746 m. Voucher: Watuma BM W0056 (EA, HIB).

**Pupalialappacea(L.)Juss.var.argyrophylla C.C.Towns** – Habit: Herb. Habitat: Forest edges, clearings, bushland, abandoned cultivations, 600–1766 m. Vouchers: Watuma BM W0282 (EA, HIB), Mungai GM et al. EW2836, Napier ER 924, Hucks M 787 (EA).

***Sericocomopsishildebrandtii* Schinz** – Habit: Shrub. Habitat: Semi-desert scrub and deciduous bushland, 500–800 m. Vouchers: Hildebrandt JM 2584, Ossent JR 26 (EA).

***Sericocomopsispallida* (S.Moore) Schinz** – Habit: Shrub. Habitat: Semi-desert scrub and deciduous bushland, ca. 500–650 m. Vouchers: Napier ER 1006, Rauh W 30 (EA).

***Suaedamonoica* Forssk. ex J.F.Gmel.** – Habit: Shrub. Habitat: Open bushland and scrub, ca. 600 m. Voucher: Hucks M 872 (EA).

**F46. ANACARDIACEAE**

5 Genera, 11 species

***Lanneaalata* (Engl.) Engl.** – Habit: Tree. Habitat: *Acacia*-*Commiphora* bushland, rocky hill outcrops, 518–1700 m. Vouchers: Medley KE 509, Faden RB & Faden AJ 74/279, Friis I & Hansen OJ 2660, Dale IR 14565, Bally PRO 20505, Hucks M 455, Greenway PJ & Kanuri K 12687 (EA).

***Lannearivae* (Chiov.) Sacleux** – Habit: Tree. Habitat: Wooded grassland, deciduous bushland, rocky lava areas, 500–1000 m. Vouchers: Sacleux C 2353, Medley KE 973, Uhlig C 39 (EA).

***Lanneaschweinfurthii* (Engl.) Engl.** – Habit: Tree. Habitat: Deciduous woodland and bushland, river valleys, 950–1100 m. Vouchers: Watuma BM W0321 (EA, HIB), Mwachala G et al. EW187, Medley KE 426 & 851, Bally PRO 17117, Cheseny CMC 28/72, Greenway PJ & Kanuri K 8866, Gillett JB 19574 (EA).

**OzoroainsignisDelilesubsp.reticulata (Baker f.) J.B.Gillett** – Habit: Tree. Habitat: Wetter deciduous woodland and bushland, wooded grassland, 650–1200 m. Vouchers: Mwachala G et al. EW152, Mungai GM et al. EW1457, Medley KE 797 (EA).

***Searsiacrenulata* (A.Rich.) Moffett** – Habit: Tree. Habitat: Deciduous and evergreen woodland and bushland, 650–1200 m. Vouchers: Mungai GM et al. EW1293, Medley KE 447 & 574 (EA).

***Searsialongipes* (Engl.) Moffett** – Habit: Tree. Habitat: Upland evergreen bushland, forest edges, 650–1836 m. Vouchers: Watuma BM W0115 (EA, HIB), Medley KE 646 & 790, Heller E s.n. (EA).

**Searsiapyroides(Burch.)Moffettvar.pyroides** – Habit: Shrub. Habitat: Upland evergreen bushland, forest edges, 1700–2000 m. Vouchers: Faden RB et al. NMK Exped. 301, Beentje HJ et al. NMK Exped. 933 (EA).

***Searsiaquartiniana* (A.Rich.) A.J.Mill.** – Habit: Shrub. Habitat: Evergreen bushland, wooded grassland, riverine, 800–950 m. Voucher: Kabuye CHS et al. NMK Exped. 601 (EA).

***Searsiatenuinervis* (Engl.) Moffett** – Habit: Shrub. Habitat: Evergreen bushland, wooded grassland, riverine, 800–1800 m. Vouchers: Kabuye CHS et al. NMK Exped. 661, Christenhusz MJM, Kamau P, Chase MW, Mbale M & Kyaa J 6680 (EA).

**Sclerocaryabirrea(A.Rich.)Hochst.subsp.caffra (Sond.) Kokwaro** – Habit: Tree. Habitat: Bushland, 550–650 m. Vouchers: Medley KE 953, Mungai GM et al. EW s.n. (EA).

***Sorindeiamadagascariensis* DC.** – Habit: Tree. Habitat: Upland evergreen forest, 1425–1850 m. Vouchers: Wakanene KM & Mwangangi OM 381 & 653, Faden RB et al. NMK Exped. 381, Beentje HJ et al. NMK Exped. 880, Faden RB et al. 71/1012 (EA).

**F47. ANNONACEAE**

5 Genera, 7 species

*Annonacherimola Mill. – Habit: Shrub. Habitat: Cultivated, 700–900 m. Vouchers: Mungai GM et al. EW3281, Bally PRO 8598 (EA).

***Artabotrysmonteiroae* Oliv.** – Habit: Liana. Habitat: Forest edges in evergreen forest, 900–925 m. Vouchers: Mwachala G et al. EW902, Mungai GM et al. EW3540 (EA).

***Monanthotaxisbuchananii* (Engl.) Verdc.** – Habit: Shrub/Liana. Habitat: Riverine and hillside thicket, 1040–1100 m. Voucher: Mwachala G et al. EW201 (EA).

***Uvariaacuminata* Oliv.** – Habit: Shrub/Liana. Habitat: Wet evergreen forest, scrubby forest, woodland, 600–1075 m. Vouchers: SAJIT–005397 (EA, HIB), Kabuye CHS et al. NMK Exped. 615, Mwachala G et al. EW194, 512 & 3324, Muasya J et al. 02/2006/06, Faden RB & Faden AJ 74/465, Medley KE 505 & 872, Faden RB et al. 70/956 & 957, Gillett JB 19566, Bally PRO 13589 (EA).

***Uvarialucida* Bojer ex Benth.** – Habit: Shrub. Habitat: Moist montane forest, rocky outcrops, 1450–1875 m. Vouchers: Faden RB et al. NMK Exped. 560, Faden RB, Faden AJ & Smeenk C 71/1008 (EA).

***Uvariascheffleri* Diels** – Habit: Shrub/Liana. Habitat: Forest, evergreen thicket and scrub, 650–1350 m. Vouchers: Watuma BM W0010 (EA, HIB), Kabuye CHS et al. NMK Exped. 622, Mwachala G et al. EW50, 112, 135, 280, 397 & 1297, Mungai GM et al. EW2824 (EA), Medley KE 742 (EA).

***Xylopiaaethiopica* (Dunal) A.Rich.** – Habit: Tree. Habitat: Evergreen and deciduous bushland, riverine associations, ca. 1078 m. Voucher: SAJIT–004635 (EA, HIB).

**F48. APHLOIACEAE**

1 Genus, 1 species

***Aphloiatheiformis* (Vahl) Benn.** – Habit: Tree. Habitat: Montane evergreen forest, 1000–1950 m. Vouchers: SAJIT–006351 (EA, HIB), Faden RB et al. NMK Exped. 249 & 369, Drummond RB & Hemsley JH 4362, Raler I 0060, Faden RB & Faden AJ 71/1007, Gardner HM 2953, Joanna B 8939, Medley KEM s.n. (EA).

**F49. APIACEAE**

8 Genera, 8 species

***Afroligusticumlinderi* (C.Norman) P.J.D.Winter** – Habit: Herb. Habitat: Forest remnants, margins and glades, 2000–2170 m. Voucher: Faden RB et al. NMK Exped.155 (EA).

*Cyclospermumleptophyllum (Pers.) Sprague ex Britton & P.Wilson – Habit: Herb. Habitat: Weed of cultivated and waste ground, ca. 1316 m. Voucher: Kirika P et al. GBK 47990 (EA).

***Daucusincognitus* (C.Norman) Spalik, Reduron & Banasiak** – Habit: Herb. Habitat: Montane forest margins and clearings, cultivated areas, 1700–1875 m. Vouchers: Faden RB et al. NMK Exped. 477, Lubai lK HB19 (EA).

***Centellaasiatica* (L.) Urb.** – Habit: Herb. Habitat: Forest clearings, damp grasslands, ca. 1200 m. Voucher: Gillett JB & Burtt BL 17056 (EA).

***Heteromorphaarborescens* (Spreng.) Cham. & Schltdl.** – Habit: Tree. Habitat: Montane forest margins, open woodland, rocky hill slopes, 650–1850 m. Vouchers: SAJIT–005336 (EA, HIB), Mwachala G et al. in EW3524, Luke WRQ & Luke PA 6434, Wakanene KM & Mwangangi OM 258, Beentje HJ et al. NMK Exped. 394, Raler I 0102, Medley KE 489, 644 & 821 (EA).

***Lefebvrealongipedicellata* Engl.** – Habit: Herb. Habitat: Upland evergreen forest and margins, roadside, 810–1550 m. Vouchers: Kabuye CHS 82/90, Mwachala G et al. EW3561, Muasya J et al. GBK4799, Napier ER 1146 (EA).

***Saniculaelata* Buch.-Ham. ex D.Don** – Habit: Herb. Habitat: Montane forest, secondary forest, 1400–2170 m. Vouchers: SAJIT–005322 (EA, HIB), Luke WRQ et al. 4193B, Christenhusz MJM et al. 6645, Faden RB et al. NMK Exped. 99 & 417, Faden RB et al. 71/246, Gillett JB, Burtt BL & Osborn RM 17083 (EA).

***Steganotaeniaaraliacea* Hochst.** – Habit: Tree. Habitat: Dry woodland, deciduous bushland, rocky hillslopes, 720–1000 m. Vouchers: Mwachala G et al. EW566 & 967, Drummond RB & Hemsley JH 4413, Medley KE 750, Kluguess LM 46 (EA).

**F50. APOCYNACEAE**

34 Genera, 64 species

***Acokantheraoppositifolia* (Lam.) Codd** – Habit: Shrub. Habitat: Forest edges, dry forest, woodland, 1195–1875 m. Vouchers: SAJIT–004529 & 006398, Watuma BM W0274 (EA, HIB), Mwachala G et al. EW3057a, Medley KE 939, Taita Hills NMK Exped. 305, Joanna in CM 8902 (EA).

***Adeniumobesum* (Forssk.) Roem. & Schult.** – Habit: Shrub. Habitat: Dry woodland and bushland, 487–800 m. Vouchers: SAJIT–005404 (EA, HIB), Archer PG 622, Bally PRO 8675b, Alof E 318 (EA).

***Ancylobothryspetersiana* (Klotzsch) Pierre** – Habit: Liana. Habitat: Woodland and bushland, 650–1000 m. Voucher: Medley KE 553 (EA).

***Baseonemagregorii* Schltr. & Rendle** – Habit: Climber. Habitat: Dry bushland, evergreen forest on rocky outcrops, ca. 850 m. Voucher: Gilbert M & Gilbert C 6122 (EA).

*Calotropisgigantea (L.) W.T.Aiton – Habit: Shrub. Habitat: Bare red soils near roads and villages, 600–700 m. Vouchers: SAJIT–005337 & 005388 (EA, HIB).

***Calotropisprocera* (Aiton) W.T.Aiton** – Habit: Shrub. Habitat: Evergreen bushland and scrub on red soils, roadside, ca. 622 m. Voucher: SAJIT–005360 (EA, HIB).

***Carallumaarachnoidea* (P.R.O.Bally) M.G.Gilbert** – Habit: Climber. Habitat: Grass or succulent dominated communities, in the open or in between shrubs, 600–750 m. Vouchers: SAJIT–005411 (EA, HIB), Luke WRQ & Luke PA 4173, Newton LE 5589 (EA).

**CarallumaturneriE.A.Brucesubsp.tuneri** – Habit: Climber. Habitat: Sparse grassland, succulent bushland on sandy to loamy soil, ca. 1350 m. Voucher: Goyder DJ, Masinde PS, Ulrich M & Christopher WM 4033 (EA).

***Carissabispinosa* (L.) Desf. ex Merxm.** – Habit: Shrub. Habitat: Dry forest, 1000–1180 m. Vouchers: Mwachala G et al. in EW248, Mungai G et al. in EW1330 (EA).

***Carissaspinarum* L.** – Habit: Shrub. Habitat: Roadside, bushland, forest edge, 650–1786 m. Vouchers: Watuma BM W0264 (EA, HIB), Omino EA 73 & 86, Medley KE 511, 767 & 850 (EA).

***Carissatetramera* (Sacleux) Stapf** – Habit: Shrub. Habitat: Montane woodland, thicket and bushland, 650–1000 m. Vouchers: Medley KE 483 & 850 (EA).

***Carvalhoacampanulata* K.Schum.** – Habit: Shrub. Habitat: Moist and evergreen forest, riverine forest, 1000–1640 m. Voucher: Medley KE 437 (EA).

*Catharanthusroseus (L.) G.Don – Habit: Tree. Habitat: Cultivated in villages, ca. 670 m. Voucher: Medley KE 424 (EA).

***Ceropegiaaffinis* Vatke** – Habit: Climber. Habitat: *Acacia*-*Commiphora* woodland, ca. 500 m. Vouchers: Archer PG 110 & 397 (EA).

***Ceropegiaaffinis* Vatke × CeropegiaracemosaN.E.Br.subsp.racemosa** – Habit: Climber. Habitat: Dry evergreen forest, bushland and thicket, rocky slopes, 800–1060 m. Vouchers: Masinde PS 844, Kabuye CHS et al. NMK Exped. 587, Gilbert M & Gilbert C 6120 (EA).

**CeropegiaalbiseptaJum. & H.Perriervar.albisepta** – Habit: Climber. Habitat: Upland forest patches and riverine areas, 900–1300 m. Vouchers: Luke WRQ & Luke PA 5529, Archer PG 468 (EA).

***Ceropegiaampliata* E.Mey.** – Habit: Climber. Habitat: Forest edges, bushland on rocky ground, ca. 914 m. Voucher: Archer PG 558 (EA).

**CeropegiaaristolochioidesDecne.subsp.aristolochioides**– Habit: Climber. Habitat: Semi-evergreen scrub, thickets and dry forests, 800–1000 m. Vouchers: Mwachala G et al. EW3345, Gillett JB 19563, Napier ER 940, Mrs Robertson SA 3588 (EA).

***Ceropegiaballyana* Bullock** – Habit: Climber. Habitat: Forest patches, wooded bushland, riverine, 850–1250 m. Vouchers: SAJIT–006421 (EA, HIB), Kabuye CHS et al. NMK Exped. 681, Mungai GM et al. EW1296 & 1375, Faden RB, Evans A & Rathbun G 69/456, Napier ER 1145, Drummond RB & Hemsley JH 4419 (EA).

***Ceropegiabulbosa* Roxb.** – Habit: Climber. Habitat: Forest clearings and glades, wooded grassland, ca. 1873 m. Voucher: SAJIT–003299 (EA, HIB).

**CeropegiadenticulataK.Schum.var.denticulata** – Habit: Climber. Habitat: Forest edges and bushland, 862–1100 m. Vouchers: SAJIT–004620, Watuma BM W0191 (EA, HIB), Masinde PS and Meve 820 & 870 (EA).

^**NE**^**CeropegiadistinctaN.E.Br.var.rostrata Masinde** – Habit: Climber. Habitat: Forest edges and clearings, near streams and rivers, ca. 985 m. Voucher: Faden RB & Faden AJ 74/494 (EA).

***Ceropegiainornata* P.R.O.Bally ex Masinde** – Habit: Climber. Habitat: Rocky deciduous bushland, 700–1050 m. Vouchers: Masinde PS, Goyder DJ, Meve U, Christopher MW 838, 839 & 853, Faden RB & Faden AJ 74/493, Archer PG 391 & 587, Gillett JB 19565 (EA).

^**NE**^***Ceropegiakonasita* Masinde** – Habit: Climber. Habitat: Bushland and thickets, sandy soil with high humus content, 985–1050 m. Vouchers: Masinde PS & Meve U 819 & 854, Faden RB & Faden AJ 74/492, Archer PG 428 (EA).

***Ceropegiameyeri-johannis* Engl.** – Habit: Climber. Habitat: Forest edges and clearings, thickets on hills, 800–1000 m. Voucher: Masinde PS & Meve U 936, Kabuye CHS et al. NMK Exped. 748, Luke WRQ & Luke PA 4109 (EA).

***Ceropegianilotica* Kotschy** – Habit: Climber. Habitat: *Acacia*-*Commiphora* bushland, forest edges, 1000–1220 m. Vouchers: Masinde PS 836 & 840, Mwachala G et al. EW3267, Faden RB et al. 69/405, Napier ER 1137, Unknown collector KEN1002 (EA).

***Ceropegiaspeciosa* H.Huber** – Habit: Climber. Habitat: Moist forest, 1795–1800 m. Voucher: Christenhusz MJM, Kamau P, Chase MW, Mbale M & Kyaa J 6671 (EA).

**^E^*Ceropegiaverticillata* Masinde** – Habit: Climber. Habitat: Dense and open evergreen forest, 1600–1800 m. Vouchers: Christenhusz MJM, Kamau P, Mbale M, Chase MW & Kyaa J 6671, Bytebier B 1160, Faden RB & Faden AJ 72/277, Joanna B 8790 & 8988 (EA).

***Cryptolepisnigrescens* (Wennberg) L.Joubert & Bruyns** – Habit: Climber. Habitat: Gallery forests, thickets, 800–1200 m. Vouchers: SAJIT–005387 (EA, HIB), Mwachala G, Mungai GM & Lusweti A 261, Verdcourt B 3598A (EA).

**Cynanchumgerrardi(Harv.)Liedesubsp.gerrardi** – Habit: Climber. Habitat: *Acacia* scrub, dry bushland, 850–930 m. Voucher: Goyder DJ & Masinde PS 3964 (EA).

**Cynanchumgerrardi(Harv.)Liedesubsp.lenewtonii (Liede) Goyder** – Habit: Climber. Habitat: *Acacia*-*Commiphora* bushland, 610–1300 m. Voucher: Napier ER 1064 (EA).

***Cynanchuminsipidum* (E.Mey.) Liede & Khanum** – Habit: Climber. Habitat: Upland rainforest, *Acacia*-*Commiphora* bushland, open grassland, 750–1850 m. Vouchers: Gillett JB 17228, Christenhusz MJM et al. 6658, Beentje HJ et al. NMK Exped. 1048 (EA).

***Cynanchumstoloniferum* (B.R.Adams & R.W.K.Holland) Goyder** – Habit: Climber. Habitat: Rocky ground, ca. 1400 m. Voucher: Gilbert MG 5824 (EA).

**Cynanchumviminale(L.)L.subsp.crassicaule Liede & Meve** – Habit: Herb/Climber. Habitat: Montane forest margins over rocks, ca. 1150 m. Vouchers: Goyder DJ, Masinde PS, Meve U & Whitehouse C 4029, Joanna B 9015 (EA).

**Cynanchumviminale(L.)L.subsp.odontolepis (Balf.f.) Goyder** – Habit: Herb/Climber. Habitat: Dry *Acacia*-*Commiphora* woodland. Voucher: Bally PRO 16663 (EA).

**Cynanchumviminale(L.)L.subsp.suberosum (Meve & Liede) Goyder** – Habit: Herb/Climber. Habitat: *Acacia*-*Commiphora* bushland, sandy soils, rocky ground, ca. 1050 m. Voucher: Godyer DJ, Masinde PS, Meve U & Whitehouse C 4031 (EA).

**Cynanchumviminale(L.)L.subsp.viminale** – Habit: Herb/Climber. Habitat: *Commiphora* bushland, 487–950 m. Vouchers: Watuma W0189 (EA, HIB), Mwachala G et al. EW769, Gillett JB 19585, Bally PRO 13566, Ossent JR 125, Greenway PJ & Kanuri K 12679 (EA).

***Desmidorchisretrospiciens* Ehrenb.** – Habit: Shrub. Habitat. Semi-arid, open overgrazed lands, 600–800 m. Voucher: Hucks M 1045 (EA).

***Desmidorchisspeciosa* (N.E.Br.) Plowes** – Habit: Herb. Habitat: Dry bushland and wooded grassland, ca. 590 m. Voucher: Faden RB & Faden AJ 74/521 (EA).

*Gomphocarpusphysocarpus E.Mey. – Habit: Herb. Habitat: Seasonally wet pastures and disturbed lands, ca. 1400 m. Voucher: Mungai GM et al. EW1323 (EA).

***Gymnemasylvestre* (Retz.) R.Br. ex Schult.** – Habit: Liana/Shrub. Habitat: Riverine forest margins, thicket and dry bushland, 900–1200 m. Voucher: SAJIT–005395 (EA, HIB).

***Holarrhenapubescens* Wall. ex G.Don** – Habit: Shrub/Tree. Habitat: Riverine forest, woodland, evergreen bushland, 650–1400 m. Vouchers: Kabuye CHS et al. NMK Exped. 810 & 948, Mungai GM et al. EW2815, Medley KE 459, Bally PRO 13610 (EA).

**^NE^*Huerniaandreaeana* (Rauh) L.C.Leach** – Habit: Herb. Habitat: Dry deciduous bushland on rocky ground, ca. 480 m. Voucher: Luke WRQ & Luke PA 5576 (EA).

***Hunteriazeylanica* (Retz.) Gardner ex Thwaites** – Habit: Tree. Habitat: Riverine and evergreen forest, 1500–1875 m. Vouchers: Faden RB et al. NMK Exped. 217, Faden RB et al. 69/881 (EA).

***Landolphiabuchananii* (Hallier f.) Stapf** – Habit: Liana. Habitat: Upland rainforest margins, riverine forest, 1200–1875 m. Vouchers: SAJIT–004606 (EA, HIB), Beentje HJ 2192, Faden RB et al. NMK Exped. 509, Medley KE 687 (EA).

***Marsdeniaabyssinica* (Hochst.) Schltr.** – Habit: Liana. Habitat: Forest margins, montane woodland, 650–1000 m. Voucher: Medley KE 771 (EA).

***Mondiawhitei* (Hook.f.) Skeels** – Habit: Liana. Habitat: Moist habitats, riverine and disturbed forests, 1200–1300 m. Voucher: SAJIT–005352 (EA, HIB).

***Orbeasemota* (N.E.Br.) L.C.Leach** – Habit: Herb. Habitat: Grassland on steep slope, rocky places, ca. 1200 m. Voucher: Luke WRQ & Mrs. Robertson SA 5511 (EA).

***Orbeasubterranea* (E.A.Bruce & P.R.O.Bally) Bruyns** – Habit: Climber. Habitat: Bare ground, thin grassland, succulent communities, 600–680 m. Vouchers: Faden RB, Evans A & Rathbun G 69/480B, Joanna s.n. in Bally S20 (EA).

**^NE^*Orbeataitica* Bruyns** – Habit: Herb. Habitat: Rocky outcrops and inselbergs, ca. 1300 m. Voucher: Luke WRQ & Luke PA 5561 (EA).

***Pachycarpuslineolatus* (Decne.) Bullock** – Habit: Herb. Habitat: Open deciduous woodland, seasonally waterlogged grasslands, ca. 1158 m. Voucher: Bally PRO 8577 (EA).

**Pergulariadaemia(Forssk.)Chiov.subsp.daemia** – Habit: Climber. Habitat: Dry bushland, 450–1200 m. Vouchers: Watuma BM W0026 (EA, HIB), Goyder DJ & Masinde PS 3956 & 3957, Gillett JB 17210 & 17221 (EA).

***Pleiocarpabicarpellata* Stapf** – Habit: Shrub/Tree. Habitat: Moist evergreen forest, 1465–1616 m. Vouchers: SAJIT–006384 (EA, HIB), Omino EA 82 & 157, Luke WRQ & Luke PA 4202 (EA).

***Rauvolfiacaffra* Sond.** – Habit: Tree. Habitat: Moist forest, riverine forest, 650–1640 m. Voucher: Medley KEM s.r. (EA).

***Rauvolfiamannii* Stapf** – Habit: Shrub/Tree. Habitat: Moist forest, 1000–1925 m. Vouchers: SAJIT–005347, Watuma BM W0049 (EA, HIB), Omino EA 52 & 81, Harvey YB, Mwachala G & Vollesen KB 32, Faden RB et al. NMK Exped. 172 & 354, Beentje HJ et al. NMK Exped. 851, Faden RB & Faden AJ 77/325, Drummond RB & Hemsley JH 4329, Faden RB & Evans A 69/876, Raler I 0042, Faden RB, Evans A, Msafiri F & Smeenk C 71/20, Lubai lK HB27, Medley KE 700 & 882 (EA).

***Sabacomorensis* (Bojer ex A.DC.) Pichon** – Habit: Liana. Habitat: Moist forest, forest edges, secondary forest, woodland, rocky places, 500–1350 m. Vouchers: Watuma BM W0153 (EA, HIB), Kabuye CHS 82/131, Mwachala G et al. EW583 & 910, Muasya J 2096, Omino EA 87, Kluguess LM 45, Bally PRO 1940, 1949, 8580, 13612, Greenwayi PJ 8771, Skinner FE & Mcgough JM s.n. (EA).

***Sacleuxianewii* (Benth.) Bullock** – Habit: Shrub. Habitat: *Acacia*-*Commiphora*-*Euphorbia* deciduous bushland on rocky outcrops, 600–1000 m. Vouchers: Faden RB and Faden AJ 74/450, Luke WRQ & Luke PA 16117, Goyder et al. 4030, Medley KE 895, Gillett JB 16877 & 17237 (EA).

***Secamonealpini* Schult.** – Habit: Liana. Habitat: Montane forest, 1400–1875 m. Vouchers: Wakanene KM & Mwangangi OM 665, Faden RB et al. NMK Exped. 552 (EA).

***Secamonepunctulata* Decne.** – Habit: Liana. Habitat: Forest, riverine and thickets, 800–1875 m. Vouchers: SAJIT–005357 (EA, HIB), Kabuye CHS et al. NMK Exped. 723B, Mwachala G et al. EW81, Mungai GM et al. EW1666, Beentje HJ et al. NMK Exped. 1012, Faden RB et al. NMK Exped. 563, Medley KE 753 (EA).

***Secamoneschweinfurthii* K.Schum** – Habit: Liana. Habitat: *Acacia*-*Commiphora* bushland and woodland, 650–1000 m. Vouchers: Kabuye CHS et al. NMK Exped. 611 & 723A, Mwachala G et al. EW948, Faden RB & Faden AJ 74/463, Medley KE 488, 500 & 864 (EA).

***Strophanthusmirabilis* Gilg** – Habit: Shrub. Habitat: *Acacia*-*Commiphora* bushland, 550–650 m. Vouchers: Medley KE 653 & 785 (EA).

***Tabernaemontanastapfiana* Britten** – Habit: Tree. Habitat: Montane forest, 1400–1875 m. Vouchers: SAJIT–004597, Watuma BM W0125 (EA, HIB), Beentje HJ et al. NMK Exped. 869, Faden RB et al. NMK Exped. 235 & 278, Drummond RB & Hemsley JH 4368, Gillett JB, Burtt BL & Osborn RM 17102, Faden RB, Faden AJ, Smeenk C 71/980, Raler I 0001 (EA).

***Tabernaemontanaventricosa* Hochst. ex A.DC.** – Habit: Tree. Habitat: Forest margins, riverine and grassy areas. Voucher: Omino EA 85 (EA).

***Tacazzeaapiculata* Oliv.** – Habit: Liana. Habitat: Mist forest patches, stream sides, 1800–2000 m. Voucher: SAJIT–004583 (EA, HIB).

***Tacazzeaconferta* N.E.Br.** – Habit: Liana. Habitat: *Podocarpus* afromontane forest, moist bushland, 1700–1875 m. Voucher: Faden RB et al. NMK Exped. 339 (EA).

***Telosmaafricana* (N.E.Br.) N.E.Br.** – Habit: Liana. Habitat: Margins of wet forest, 800–850 m. Voucher: Mwachala G et al. EW3338 (EA).

***Vincetoxicumfleckii* (Schltr.) Meve & Liede** – Habit: Liana. Habit: Dry forest remnants, 800–950 m. Vouchers: Kabuye CHS et al. NMK Exped. 594, Verdcourt B & Polhill RM 2737 (EA).

**^E^*Vincetoxicum* sp. B of FTEA** [*Tylophora* sp. B of FTEA] – Habit: Climber. Habitat: Montane forest, ca. 1760 m. Voucher: Luke WRQ & Luke PA 5494 (EA).

**F51. AQUIFOLIACEAE**

1 Genus, 1 species

***Ilexmitis* (L.) Radlk.** – Habit: Tree. Habitat: Upland rainforest, dry evergreen forest, derived thicket, 1350–1850 m. Vouchers: Luke WRQ & Luke PA 4220, Wakanene KM & Mwangangi OM 620 & 624, Faden RB et al. NMK Exped. 377, Medley KE 923, Beentje HJ et al. NMK Exped. 1102, Raler I 0123 (EA).

**F52. ARALIACEAE**

3 Genera, 5 species

***Astropanaxmyrianthus* (Baker) Lowry, G.M.Plunkett & al.** – Habit: Liana/Tree: Habitat: Wet upland forest edges, 1400–2195 m. Vouchers: SAJIT–004578 (EA, HIB), Faden RB et al. NMK Exped. 140, Beentje HJ et al. NMK Exped. 884, Drummond RB & Hemsley JH 4323, Raler I 0052, Medley KE 926, Verdcourt B & Polhill RM 2720, Gillett JB, Burtt BL, Osborn RM 17090, Dale IR 11539, Gardner HM 2931, Faden RB et al. s.n. (EA) Near Threatened.

***Cussoniaholstii* Harms ex Engl.** – Habit: Tree. Habitat: Upland dry evergreen forest, evergreen montane woodland, 650–1000 m. Voucher: Medley KE 1009 (EA).

***Cussoniaspicata* Thunb.** – Habit: Tree. Habitat: Upland rainforest and dry evergreen forest, 1200–1640 m. Vouchers: Watuma BM W0127 (EA, HIB), Medley KE 1007, Verdcourt B & Polhill RM EAH11987, Raler I 0018 (EA).

***Polysciasfulva* (Hiern) Harms** – Habit: Tree. Habit: Upland and lowland rainforest, 1400–1875 m. Vouchers: Faden RB et al. NMK Exped. 264, Drummond RB & Hemsley JH 4378, Raler I 0023, Medley KEM p.r. (EA).

***Polysciasstuhlmannii* Harms** – Habit: Tree. Habitat: Rain- and mist-forest, 1425–1981 m. Vouchers: Watuma BM W0305 (EA, HIB), Wakanene KM & Mwangangi OM 630, Faden RB et al. NMK Exped. 288, 374 & 461, Raler I 0048, Faden RB & Githui M 70/745, Faden RB & Faden AJ 72/266 & 77/320, Faden RB, Faden AJ, Smeenk C 71/1004, Dale IR 1130, Gardner HM 2922 (EA). Endangered.

**F53. ASTERACEAE**

46 Genera, 77 species

***Acmellacaulirhiza* Delile** – Habit: Herb. Habitat: Forest margins, riverbanks, seasonally wet areas, cultivated areas, 1000–1560 m. Vouchers: Gillett JB & Burtt BL 17069, Sacleux C 1386 (EA).

*Acmellauliginosa (Sw.) Cass. – Habit: Herb. Habitat: Damp moist forest zone, grassland near rivers ca. 1100–1200 m. Voucher: Mwachala G et al. EW570 (EA).

***Adenostemmamauritianum* DC.** – Habit: Herb. Habitat: Margins of pathways through forest, forest edges, 1371–1875 m. Vouchers: SAJIT–006372 (EA, HIB), Faden RB et al. NMK Exped. 292 & 954, Drummond RB & Hemsley JH 4383, Dale IR 3808, Napier ER 1117 (EA).

***Ambassahochstetteri* (Sch.Bip. ex Walp.) Steetz** – Habit: Subshrub. Habitat: Forest and forest margins, 1500–2170 m. Vouchers: SAJIT–004106 (EA, HIB), Drummond RB & Hemsley JH 4297, Faden RB et al. NMK Exped. 144 (EA).

**AnisopappuschinensisHook. & Arn.subsp.oliverianus (Wild) S.Ortíz, Paiva & Rodr.Oubiña** – Habit: Herb. Habitat: Forest margins and ruderal sites, 1100–2000 m. Vouchers: Luke WRQ & Luke PA 5357, Drummond RB & Hemsley JH 4347, Faden RB et al. NMK Exped. 439, Sacleux C 1397, Lewis WH 5933, Dale IR 3805 (EA).

***Aspiliamossambicensis* (Oliv.) Wild** – Habit: Subshrub. Habitat: Forest margins, woodland, bushland and grassland, ruderal sites, 500–1359 m. Vouchers: Watuma BM W0250 (EA, HIB), Medley KE 900 (EA).

***Baccharoideslasiopus* (O.Hoffm.) H.Rob.** – Habit: Shrub. Habitat: Forest clearings or margins, secondary bush derived from forest, ca. 700–1121 m. Vouchers: Medley KE 831, Hildebrandt JM 2551 (EA).

***Bidenshildebrandtii* O.Hoffm.** – Habit. Subshrub. Habitat: Scrub on hillsides, rock crevices, 609–950 m. Vouchers: Kabuye CHS et al. NMK Exped. 649, Hildebrandt JM 2432, Napier ER 1016, Verdcourt B 3890A (EA).

***Bidensholstii* Sherff** – Habit: Subshrub. Habitat: Forest margins, disturbed forests, open bushland next to forest, 1000–2195 m. Vouchers: Luke WRQ & Luke PA 4168, Ojiambo 31, Beentje HJ 2136, Faden RB et al. NMK Exped. 14, Hildebrandt JM 2432a, Sacleux C 2503, Gillett JB 18769, Verdcourt B & Polhill RM 2718, Napier ER 1321, Faden RB et al. 71/130, Lewis WH 5934, Lynes H 282 (EA).

***Bidenskilimandscharica* (O.Hoffm.) Sherff** – Habit: Herb. Habitat: Forest margins, wooded grassland, 750–1582 m. Vouchers: Watuma BM W0255 (EA, HIB), Mwachala G et al. EW3141, Kirika P et al. 1/2006/07, Muasya J & Medley KE 817, Sacleux C 2424 (EA).

***Blepharispermumzanguebaricum* Oliv. & Hiern** – Habit: Shrub. Habitat: Dry forest, forest margins, woodland, bushland, 650–1000 m. Vouchers: Watuma BM W0027 (EA, HIB), Medley KE 543 & 570, Gillett JB 17225 (EA).

***Bothrioclineargentea* (O.Hoffm.) Wild & G.V.Pope** – Habit: Shrub. Habitat: Montane forest in rock crevices, 1700–2000 m. Vouchers: Faden RB et al. NMK Exped. 291, Bally PRO 13510, Drummond RB & Hemsley JH 4348 (EA).

^**NE**^***Bothrioclineglomerata* (O.Hoffm. & Muschl.) C.Jeffrey** – Habit: Shrub. Habitat: Evergreen forest, 1600–2000 m. Vouchers: Luke WRQ et al. 4209, Drummond RB & Hemsley JH 4361 (EA). Endangered.

***Bothrioclinelongipes* (Oliv. & Hiern) N.E.Br.** – Habit: Subshrub. Habitat: Forest margins and clearings, disturbed places, 1650–1850 m. Vouchers: Wakanene K, Mwangangi & Dunn B 388, Drummond RB & Hemsley JH 4287, Kluguess LM 1 (EA).

***Brachylaenahuillensis* O.Hoffm.** – Habit: Shrub/Tree. Habitat: Dry evergreen and semi-deciduous forest, bushland on sandy soils, 650–1000 m. Vouchers: Faden RB & Faden AJ 74/467, Medley KE 921 (EA). Near Threatened.

***Conyzanewii* Oliv. & Hiern** – Habit: Subshrub. Habitat: Forest margins and grassy clearings, 1500–2057 m. Vouchers: Lewis WH 5935, Murray High School 25, Klungness LM 57 (EA).

***Crassocephalumcrepidioides* (Benth.) S.Moore** – Habit: Herb. Habitat: Moist forest margins, secondary vegetation, disturbed places, cultivation weed, 1000–2000 m. Vouchers: Sacleux C 894 & 2506 (EA).

***Crassocephalummontuosum* (S.Moore) Milne-Redh.** – Habit: Herb. Habitat: Moist and evergreen forest in edges and clearings, 1350–1400 m. Voucher: Joanna B 8866 (EA).

***Dichrocephalachrysanthemifolia* (Blume) DC.** – Habit: Herb. Habitat: Secondary vegetation, margins of cultivated lands, ca. 1800 m. Voucher: Drummond RB & Hemsley JH 4318 (EA).

***Emiliadiscifolia* (Oliv.) C.Jeffrey** – Habit: Herb. Habitat: Grassland, disturbed areas, roadside, cultivated areas, 900–1300 m. Voucher: Sacleux C 2606 (EA).

***Emiliasomalensis* (S.Moore) C.Jeffrey** – Habit: Herb. Habitat: Short grassland on rocky slopes, mountain summits, 1330–1750 m. Vouchers: Mwachala G et al. EW3149, Wakanene KM & Mwangangi OM 561, Hilderbrandt JM 2557 (EA).

*Erigeronkarvinskianus DC. – Habit: Herb. Habitat: Montane forest, roadside banks and clearings, 1150–2170 m. Vouchers: Faden RB et al. NMK Exped. 85, Friis I & Hansen OJ 2668, Bally PRO 13629, Kluguess LM 62 (EA).

***Eriosemanutans* Schinz** – Habit: Herb. Habitat: Forest edges, grassland with *Acacia*, 1675–1725 m. Voucher: SAJIT–003298 (EA, HIB).

***Erythrocephalummarginatum* (O.Hoffm.) S.Ortíz & A.P.Cout.** – Habit: Herb. Habitat: Subshrub. Dry forest, riverine forest margins, rocky slopes, deciduous woodland, 800–1700 m. Vouchers: Kabuye CHS et al. NMK Exped. 623, Mwachala G et al. EW521, Wakanene KM & Mwangangi OM 447, Gillett JB, Burtt B & Osborn RM 17162, Hildebrandt JM 2559 (EA).

***Eschenbachiasubscaposa* (O.Hoffm.) G.L.Nesom** – Habit: Herb. Habitat: Upland grassland, rocky areas, 1300–2170 m. Vouchers: Faden RB et al. NMK Exped. 152, Gilbert MG 7094 (EA).

*Flaveriatrinervia (Spreng.) C.Mohr – Habit: Herb. Habitat: Waste ground, weed of cultivation, 800–1500 m. Vouchers: SAJIT–004121 (EA, HIB), Wakanene KM & Mwangangi OM 710, Bally PRO 13905 (EA).

*Galinsogaparviflora Cav. – Habit: Herb. Habitat: Forest margins, roadside, waste places, weed of cultivation. Voucher: Cheseny CMC 24/72 (EA).

***Gutenbergiacordifolia* Benth. ex Oliv.** – Habit: Herb. Habitat: Forest margins and roadsides, grassland, rocky hillsides, cultivated lands, 950–1700 m. Vouchers: Mwachala G et al EW98, 331, 339, 804, 917 & 3121, Gillett JB, Burtt BL & Osborn RM 17074, Klungness LM 3 (EA).

***Gymnanthemumamygdalinum* (Delile) Sch.Bip.** – Habit: Liana/Shrub/Tree. Habitat: Forest margins, woodland, secondary bushland, ca. 1400 m. Voucher: Wakanene KM & Mwangangi OM 657 (EA).

***Gymnanthemumauriculiferum* (Hiern) Isawumi** – Habit: Shrub. Habitat: Forest clearings and margins, woodland, secondary bush, 1700–1875 m. Voucher: Faden RB et al. NMK Exped. 330 (EA).

***Gymnanthemummyrianthum* (Hook.f.) H.Rob.** – Habit: Subshrub. Habitat: Evergreen forest clearings and margins, cultivated areas, 1676–1700 m. Vouchers: Drummond RB & Hemsley JH 4371, Klungness LM 54 (EA).

***Helichrysumforskahlii* (J.F.Gmel.) Hilliard & B.L.Burtt** – Habit: Herb. Habitat: Upland grassland, forest margins and secondary vegetation, 610–2000 m. Vouchers: Kimeu JM et al. KEFRI 500, Beentje HJ et al. NMK Exped. 437b, Drummond RB & Hemsley JH 4308, Knox EB 2693 & 2712, Sacleux C 2428, Lisowski S 7301 (EA).

***Helichrysumglumaceum* DC.** – Habit: Herb. Habitat: Grassland in dry bushland on hillsides, 450–950 m. Vouchers: Mwachala G et al. EW1275, Luke WRQ & Luke PA 16119, Kokwaro JO 2601, Napier ER 942, Bremer K 13, Gillett JB 21003B, Leuthold W 65 (EA).

***Helichrysumluteoalbum* (L.) Rchb.** – Habit: Herb. Habitat: Montane grassland, streambanks, cultivation weed, 1150–1800 m. Vouchers: Joanna B & Opiko B 8994, Fleuret A 15 (EA).

***Helichrysumkilimanjari* Oliv.** – Habit: Herb. Habitat: Montane grassland, shallow rocky soil on exposed hilltops, ca. 1676 m. Voucher: Joanna B 9004 (EA).

**Helichrysumnudifolium(L.)Less.var.oxyphyllum (DC.) Beentje** – Habit: Herb. Habitat: Grassland or scattered tree grassland, 1100–1500 m. Voucher: Sacleux C 2378 (EA).

***Helichrysumodoratissimum* (L.) Sweet** – Habit: Herb. Habitat: Montane grassland or bushland, forest margins, 1100–1900 m. Vouchers: SAJIT–006403 (EA, HIB), Mwachala G et al. EW1188, Knox EB 2708 (EA).

***Helichrysumpanduratum* O.Hoffm.** – Habit: Herb. Habitat: Grassland or forest margins, secondary bush, 1400–1600 m. Voucher: Luke WRQ et al. 4212 (EA).

***Helichrysumstenopterum* DC.** – Habit: Herb. Habitat: Forest margin, montane bushland in rocky sites, cultivations, 1100–2000 m. Vouchers: Beentje HJ et al. NMK Exped. 437a, Drummond RB & Hemsley JH 4307, Sacleux C 2501, Klungness LM 10, Joanna B s.n. (EA).

***Hoffmannanthusabbotianus* (O.Hoffm.) H.Rob., S.C.Keeley & Skvarla** – Habit: Herb. Habitat: Dry forest margins, riverine forests, secondary bushland derived from forest, 610–1584 m. Vouchers: Watuma BM W0136 (EA, HIB), Medley KE 726 & 888, Hildebrandt JM 2466 & 2499 (EA).

***Jeffreyciausambarensis* (O.Hoffm.) H.Rob., S.C.Keeley & Skvarla** – Habit: Shrub. Habitat: Secondary bushland, grassland, roadside, abandoned cultivations, 900–1875 m. Vouchers: Kabuye CHS 82/9, Mwachala G et al. EW798, 817 & 1249, Wakanene KM & Mwangangi OM 389 & 529, Faden RB et al. NMK Exped. 490A & 490B, Napier ER 1126, Freidberg A & Kaplan 91/45, Murray High School 30 (EA).

**Kleiniaabyssinica(A.Rich.)A.Bergervar.hildebrandtii (Vatke) C.Jeffrey** – Habit: Herb. Habitat: Bushland, dry grassland, woodland, open forest, 609–950 m. Vouchers: SAJIT–004618, Watuma BM W0184 (EA, HIB), Bally PRO 13953, Napier ER 898A (EA).

***Kleiniagrantii* Hook.f.** – Habit: Herb. Habitat: Open bushland on stony sites, scattered tree grassland, ca. 609 m. Voucher: Napier ER 899 (EA).

***Kleiniaimplexa* (P.R.O.Bally) C.Jeffrey** – Habit: Herb. Habitat: *Acacia*-*Commiphora* bushland, sandy or rocky sites, ca. 775 m. Voucher: Faden RB & Faden AJ 77/314 (EA).

***Kleiniaodora* (Forssk.) DC.** – Habit: Herb. Habitat: *Acacia* bushland on rocky hillsides, overgrazed sites, 560–700 m. Vouchers: Medley KE 761, Faden RB & Faden AJ 74/271 (EA).

***Kleiniasquarrosa* Cufod.** – Habit: Shrub. Habitat: Dry and moist bushland, 609–1750 m. Vouchers: Joanna B 8984, Archer PG 563, Gillett JB, Burtt BL, Osborn RM 17108 & 20730, Napier ER 1057, Bally J 13524 (EA).

***Lactucaglandulifera* Hook.f.** – Habit: Herb. Habitat: Forest margins, derived bushland, roadsides, ca. 1591 m. Voucher: Watuma BM W0219 (EA, HIB).

***Lactucainermis* Forssk.** – Habit: Herb. Habitat: Open forest path sides, grassland, weed of cultivation, ca. 1814 m. Voucher: SAJIT–006400 (EA, HIB).

***Laggerabrevipes* Oliv. & Hiern** – Habit: Subshrub. Habitat: Forest edges, 1425–1850 m. Voucher: Faden RB et al. NMK Exped. 440 (EA).

**Lipotrichescandens(Schumach. & Thonn.)Orchardsubsp.madagascariensis (Baker) D.J.N.Hind** – Habit: Herb. Habitat: Swamp and swamp margins, ca. 1200 m. Voucher: Lubai lK HB11 (EA).

***Microglossadensiflora* Hook.f.** – Habit: Shrub. Habitat: Forest margins and clearings, 1773–2170 m. Vouchers: Watuma BM W0285 (EA, HIB), Drummond RB & Hemsley JH 4313, Faden RB et al. NMK Exped. 158 (EA).

***Microglossapyrifolia* (Lam.) Kuntze** – Habit: Shrub. Habitat: Drier forest margins, secondary bushland, abandoned cultivations, ca. 1818 m. Voucher: SAJIT–004541 (EA, HIB).

***Microglossapyrrhopappa* (Sch.Bip. ex A.Rich.) Agnew** – Habit: Herb. Habitat: Forest margins, secondary bushland, grassland, 750–2024 m. Vouchers: SAJIT–006359 (EA, HIB), Hildebrandt JM 2508, Bally PRO 8714, Sacleux C 134 (EA).

***Mikaniachenopodiifolia* Willd.** – Habit: Herb. Habitat: Forest, stream banks, ca. 1632 m. Voucher: SAJIT–004590 (EA, HIB).

***Orbivestuscinerascens* (Sch.Bip.) H.Rob.** – Habit: Subshrub. Habitat: *Acacia*-*Commiphora* bushland, 550–650 m. Vouchers: Medley KE 834 (EA).

***Osteospermumvaillantii* (Decne.) Norl.** – Habit: Herb. Habitat: Montane heath and scrub, *Acacia*-*Commiphora* bushland, ruderal sites, 1000–1500 m. Vouchers: Mwachala G et al. EW1187, Sacleux C 2368 (EA).

***Piloselloideshirsuta* (Forssk.) C.Jeffrey ex Cufod.** – Habit: Herb. Habitat: Deciduous forest, open deciduous woodland, grassland, ca. 1400–1800 m. Vouchers: Kirika P et al. 01/2006/33, Gillett JB 17256 (EA).

***Pseudoconyzaviscosa* (Mill.) D’Arcy** – Habit: Herb. Habitat: Moist sites along river, weed of cultivation, ca. 610 m. Voucher: Napier ER 981 (EA).

***Psiadiapunctulata* (DC.) Vatke** – Habit: Shrub. Habitat: Evergreen bushland, dry forest margins, secondary bushland, 1700–1875 m. Vouchers: Faden RB et al. NMK Exped. 539, Gardner HM 3005 (EA).

***Seneciodeltoideus* Less.** – Habit: Liana. Habitat: Moist forest margins, secondary bushland in forest margin, 1500–1900 m. Vouchers: SAJIT–004115 (EA, HIB), Knox EB 2713, Sacleux C 2430 (EA).

***Seneciohadiensis* Forssk.** – Habit: Liana. Habitat: Dry forest or forest margins, evergreen or semi-deciduous bushland, 800–1200 m. Vouchers: Mwachala G et al. EW197, Luke WRQ & Luke PA 6426, Drummond RB & Hemsley JH 4418, Sacleux C 2417, Medley KE 808 (EA).

***Seneciosyringifolius* O.Hoffm.** – Habit: Liana. Habitat: Evergreen forest, forest margins, 1500–2170 m. Vouchers: Watuma BM W0140 (EA, HIB), Wakanene KM & Mwangangi OM 645 & 723, Faden RB et al. NMK Exped. 134, Beentje HJ et al. NMK Exped. 1110 (EA).

*Seneciovulgaris L. – Habit: Herb. Habitat: N/A. Voucher: Verdcourt B & Polhill RM 1418 (EA).

***Sigesbeckiaorientalis* L.** – Habit: Herb. Habitat: Ruderal sites, weed of cultivation, 1200–1400 m. Voucher: Bally PRO 8788 (EA).

***Solanecioangulatus* (Vahl) C.Jeffrey** – Habit: Climber. Habitat: Riverine forest, bushland and thicket, 800–1583 m. Vouchers: Watuma BM W0194 & W0254 (EA, HIB), Kabuye CHS et al. NMK Exped. 726 (EA).

**^NE^*Solaneciobuchwaldii* (O.Hoffm.) C.Jeffrey** – Habit: Shrub. Habitat: Moist forest, rock outcrops and crevices, 700–1875 m. Vouchers: Luke WRQ & Luke PA 4222, Gillett JB, Burtt BL & Osborn RM 17108, Faden RB et al. NMK Exped. 331, Newton LE 3247, Archer PG 563 & 14092, Bally PRO 13524 (EA).

***Solaneciomannii* (Hook.f.) C.Jeffrey** – Habit: Shrub. Habitat: Forest glades and margins, near cultivated areas, 1400–1937 m. Vouchers: Watuma BM W0092 (EA, HIB), Beentje HJ 2140, Christenhusz MJM, Kamau P, Chase MW, Mbale M & Kyaa J 6630, Faden RB et al. NMK Exped. 22 (EA).

***Solaneciomirabilis* (Muschl.) C.Jeffrey** – Habit: Herb. Habitat: Moist upland forest, 1525–1850 m. Vouchers: Bytebier B 1279, Faden RB & Githui M 70/712 (EA). Endangered.

***Sonchusoleraceus* L.** – Habit: Herb. Habitat: Ruderal sites, roadsides, weed of cultivation, ca. 1295 m. Voucher: Murray High School 101 (EA).

***Sphaeranthuskirkii* Oliv. & Hiern** – Habit: Herb. Habitat: Seasonally wet sites, ditches in bushland, 500–600 m. Vouchers: Faden RB, Faden AJ & Holland P 72/143, Rauh W Ke68 (EA).

*Tagetesminuta L. – Habit: Herb. Habitat: Weed of cultivated lands, ruderal sites, 600–1200 m. Vouchers: Cheseny CMC 104, Rauh W KE69a (EA).

*Tithoniadiversifolia (Hemsl.) A.Gray – Habit: Subshrub. Habitat: Roadside, waste ground, hedge plant, 1524–1950 m. Vouchers: Watuma BM W0216 (EA, HIB), Kluguess LM 39 (EA).

*Tridaxprocumbens L. – Habit: Herb. Habitat: Weed of cultivation and waste ground, 503–800 m. Vouchers: Wakanene KM & Mwangangi OM 704, Bremer K 14, Gardner HM 3003, Ciba-Geigy 8, Napier ER 934, Hucks M et al. 90, Greenway PJ & Kanuri K 13023 (EA).

**Vernoniagalamensis(Cass.)Less.subsp.galamensis** – Habit: Herb. Habitat: Forest clearings, secondary bushland, 1624–1875 m. Vouchers: SAJIT–004126 (EA, HIB), Faden RB et al. NMK Exped. 570 (EA).

**Vernoniagalamensis(Cass.)Less.subsp.mutomoensis M.G.Gilbert** – Habit: Herb. Habitat: *Acacia*-*Commiphora* bushland and woodland, rocky outcrops, 609–1150 m. Vouchers: Watuma BM W0007 (EA, HIB), Gilbert MG & Gilbert CI 6107, Polhill RM & Kibuwa SP 955, Hildebrandt JM 2455 & 2847 (EA).

***Vernoniaholstii* O.Hoffm.** – Habit: Subshrub. Habitat: Margins and clearings of dry evergreen forest, secondary bushland, 650–1440 m. Vouchers: SAJIT–005344 (EA, HIB), Beentje HJ et al. NMK Exped. 1101, Medley KE 596, Gillett JB 18757 (EA).

***Vernoniawakefieldii* Oliv.** – Habit: Shrub. Habitat: Evergreen forest, deciduous bushland and thicket on rocky slopes, 600–1450 m. Vouchers: Kabuye CHS et al. NMK Exped. 745, Mwachala G et al. EW3117A & 3310, Kokwaro JO 2602, Faden RB & Faden AJ 74/479, Medley KE 701, Gardner HM 3007, Gillett JB 16873 & 19576, Gilbert MG 6111, Parker I 552H (EA).

***Vernoniastrumaemulans* (Vatke) H.Rob.** – Habit: Herb. Habitat: Disturbed sites, roadsides, old cultivations, 500–1524 m. Vouchers: Kluguess LM 3A, Hucks M 680 & 762, Bremer K 7 (EA).

**F54. BALSAMINACEAE**

1 Genus, 8 species

**^E^ImpatiensengleriGilgsubsp.pubescens Grey-Wilson** – Habit: Herb. Habitat: Densely shaded forests, 1425–2134 m. Vouchers: SAJIT–05335 (EA, HIB), Wakanene KM & Mwangangi OM 687, Faden RB et al. NMK Exped. 279, 481, 767 & 946, Faden RB et al. 70/709 & 71/973, Bally PRO 8769, Drummond RB & Hemsley JH 4356, Knox EB 2689 (EA).

***Impatiensnana* Engl. & Warb.** – Habit: Herb. Habitat: Moist and shaded places in upland rainforest, ca. 1750 m. Vouchers: Wakanene KM & Mwangangi OM 370, Stella W 173 (EA).

***Impatienspseudoviola* Gilg** – Habit: Herb. Habitat: Damp shaded upland rainforest, on mossy river banks, 1000–2170 m. Vouchers: SAJIT–004534 & 004542, Watuma BM W0021 & W0116 (EA, HIB), Mwachala G et al. 1051 & EW1340, Luke WRQ & Luke PA 4156, De Block P et al. 497, Faden RB et al. NMK Exped. 1, 97 & 395, Beentje HJ 2138, Beentje HJ et al. NMK Exped. 850, Bally PRO 8699, Faden RB & Githui M 70/734, Knox EB 2638, Gillett JB, Burtt BL & Osborn RM 17082 & 18760 (EA).

***Impatienssodenii* Engl. & Warb.** – Habit: Herb. Habitat: Upland rainforest in damp shaded or open areas, 1400–2200 m. Vouchers: Mwachala G, Nyaboke & Saidi S 1052 (EA), Luke WRQ & Luke PA 4192, Bytebier B 1676, De Block P et al. 307, Faden RB et al. NMK Exped. 36 & 289, Drummond RB & Hemsley JH 4294, Knox EB 2699, Gardner HM 2908 (EA).

***Impatiensstuhlmannii* Warb.** – Habit: Herb. Habitat: Damp areas in upland rainforest, 1650–1900 m. Voucher: Knox EB 2697 (EA).

**^E^*Impatiensteitensis* Grey-Wilson** – Habit: Herb. Habitat: Upland rainforest, 1425–2170 m. Vouchers: SAJIT–005316 (EA, HIB), Luke WRQ & Luke PA 5492, Wakanene KM & Mwangangi OM 215 & 373, Beentje HJ 2137, Faden RB et al. NMK Exped. 60 & 361, De Block P et al. 300, 313 & 496, Drummond RB & Hemsley JH 4339, Gillett JB, Burtt BL & Osborn RM 17097, Knox EB 2697, Faden RB et al. 71/972, Bally PRO 8584, 12714 & 18714, Gardner HM 2941, Joanna B & Opiko 8775 (EA).

**ImpatienstinctoriaA.Rich.subsp.elegantissima (Gilg) Grey-Wilson** – Habit: Herb. Habitat: Upland rainforest in moist shaded places, ca. 1500 m. Voucher: Bally PRO 13606 (EA).

***Impatienswalleriana* Hook.f.** – Habit: Herb. Habitat: Damp shaded places in upland rainforest, 1000–1450 m. Vouchers: Mwachala G et al. EW1341, Luke WRQ & Bytebier B 5328, Beentje HJ et al. NMK Exped. 1065, Knox EB 2715, Faden RB, Faden AJ, Smeenk C, Smeenk N & Kichoi D 71/963, Bally PRO 8700, Archer PG 661 (EA).

**F55. BASELLACEAE**

1 Genus, 2 species

***Basellaalba* L.** – Habit: Climber. Habitat: Forest and cultivation margins, forest-grassland transitions, 1500–1875 m. Vouchers: Watuma BM W0071 (EA, HIB), Wakanene KM & Mwangangi OM 721, Faden RB et al. NMK Exped. 562 & 847 (EA).

***Basellapaniculata* Volkens** – Habit: Climber. Habitat: Margins of dry evergreen forest and bushland, ca. 1371 m. Voucher: Joanna B 8878 (EA).

**F56. BEGONIACEAE**

1 Genus, 1 species

***Begoniajohnstonii* Oliv. ex Hook.f.** – Habit: Herb. Habitat: Moist sites in rock crevices, 800–1515 m. Vouchers: Kabuye CHS et al. NMK Exped. 588, Mbale M et al. NMK 967, Luke WRQ et al. 4181, Faden RB, Evans A & Smeenk C 71/32, Faden RB, Evans A & Msafiri F 70/989, Faden RB et al. 71/215 (EA).

**F57. BIGNONIACEAE**

1 Genus, 1 species

**Kigeliaafricana(Lam.)Benth.subsp.africana** – Habit: Tree. Habitat: Grassland with scattered trees, woodland, remnants in farmed lands, 800–950 m. Vouchers: Watuma BM W0202 (EA, HIB), Kabuye CHS et al. NMK Exped. 671 (EA).

**F58. BORAGINACEAE**

6 Genera, 10 species

***Bourreriateitensis* (Gürke) Thulin** – Habit: Shrub. Habitat: Dry evergreen forest, *Acacia*-*Commiphora* bushland, 500–1367 m. Vouchers: Watuma BM W0232 (EA, HIB), Faden RB et al. 69/423, Medley KE 429 & 492, Hildebrandt JM 2359 (EA).

***Ehretiabakeri* Britten** – Habit: Shrub/Tree. Habitat: Mist forest on rocky slopes, deciduous woodland and bushland, 550–1450 m. Vouchers: Mwachala G et al. EW1410, Gardner HM 2986, Medley KE 520 & 728, Faden RB et al. s.n. (EA).

**EhretiacymosaThonn.var.silvatica (Gürke) Brenan** – Habit: Tree. Habitat: Rainforest, riverine forest, derived grassland and bushland, 1425–1875 m. Vouchers: Faden RB et al. NMK Exped. 491 & 564, Beentje HJ et al. NMK Exped. 855 & 957 (EA).

***Cordiamonoica* Roxb.** – Habit: Tree. Habitat: *Acacia* woodland, *Acacia*-*Commiphora* bushland, 550–650 m. Vouchers: Medley KE 524 & 533, Verdcourt B 3895B (EA).

***Cordiasinensis* Lam.** – Habit: Tree. Habitat: *Acacia*-*Commiphora* open bushland, riverine, 550–650 m. Voucher: Medley KE 497 (EA).

**CynoglossumcoeruleumHochst. ex A.DC.var.mannii (Baker & C.H.Wright) Verdc.** – Habit: Herb. Habitat: Submontane forest edges, 1700–2170 m. Vouchers: Faden RB et al. NMK Exped. 118 & 322, Drummond RB & Hemsley JH 4315 (EA).

***Cynoglossumlanceolatum* Forssk.** – Habit: Herb. Habitat: Forest edge, grassland, bushland, ca. 1581 m. Vouchers: Watuma BM W0218 (EA, HIB), Gillett JB & Burtt BL 17053 (EA).

**HeliotropiumsteudneriVatkesubsp.steudneri** – Habit: Herb. Habitat: *Combretum*, *Acacia* and *Commiphora* bushland, grassland, 500–750 m. Vouchers: Gillett JB, Burtt BL & Dunn B 17172 & 17190, Bally PRO 8649, Thulin M 295 (EA).

**HeliotropiumsteudneriVatkesubsp.bullatum Verdc.** – Habit: Herb. Habitat: *Acacia*-*Commiphora* bushland and woodland, roadside, 970–1180 m. Voucher: Mwachala G et al. EW380 (EA).

***Heliotropiumzeylanicum* (Burm.f.) Lam.** – Habit: Herb. Habitat: *Acacia*-*Commiphora* bushland, roadsides and cultivation, 950–1050 m. Voucher: Mwachala G et al. EW1446 (EA).

**Trichodesmazeylanicum(Burm.f.)R.Br.var.zeylanicum** – Habit: Herb. Habitat: *Commiphora*-*Grewia* bushland, disturbed ground and cultivations, 1750–1800 m. Voucher: Christenhusz MJM, Kamau P, Chase MW, Mbale M & Kyaa J 6678 (EA).

**F59. BRASSICACEAE**

5 Genera, 6 species

*Brassicarapa L. – Habit: Herb. Habitat: Cultivated land, abandoned fields, ca. 1524 m. Voucher: Klungness LM 9 (EA).

***Cardamineafricana* L.** – Habit: Herb. Habitat: Montane forest, clearings, along road and paths, 1700–2170 m. Vouchers: Faden RB et al. NMK Exped. 151 & 332 (EA).

***Erucastrumarabicum* Fisch. & C.A.Mey.** – Habit: Herb. Habitat: Disturbed places in upland forest, ca. 1295 m. Voucher: Murray High School 103 (EA).

***Farsetiastenoptera* Hochst.** – Habit: Herb. Habitat: Open bushland, overgrazed and cultivated lands. Voucher: Cheseny CMS 33/73 (EA).

**FarsetiastenopteraHochst.subsp.boivinii (E.Fourn.) Jonsell** – Habit: Subshrub. Habitat: Open grassland on sandy soils, ca. 487 m. Voucher: Greenway PJ & Kanuri K 12722 (EA).

***Farsetiaundulicarpa* Jonsell** – Habit: Shrub. Habitat: Bushland and wooded grasslands on volcanic soils, ca. 925 m. Voucher: Kuchar P 11069 (EA).

*Lepidiumbonariense L. – Habit: Herb. Habitat: Open grassland, roadside, waste places, settlements, 1585–1814 m. Vouchers: SAJIT–004655, Watuma BM W0252 (EA, HIB).

**F60. BURSERACEAE**

2 Genera, 12 species

***Boswellianeglecta* S.Moore** – Habit: Tree. Habitat: *Acacia*-*Commiphora* bushland, 400–850 m. Vouchers: SAJIT–005400 (EA, HIB), Medley KE 966, Faden RB & Faden AJ 74/250, Engler A 1973, Davey PRA 36 Dale IR 3897 (EA).

***Commiphoraafricana* (A.Rich.) Engl.** – Habit: Tree. Habitat: *Acacia*-*Commiphora* bushland, bushed grassland, 660–950 m. Vouchers: SAJIT–005384, Watuma BM W0176 (EA, HIB), Mwachala G et al. EW151, 338, 526 & 1177, Cheseny CMC 49/75, Faden RB & Faden AJ 74/282, Medley KE 526, Gillett JB 19584, Bally PRO 16895, Dale IR 2000 (EA).

**Commiphoraafricana(A.Rich.)Engl.var.glaucidula (Engl.) J.B.Gillett** – Habit: Tree. Habitat: Bushed grassland, *Acacia*-*Commiphora* open bushland, 540–990 m. Vouchers: Gillett JB & Glover PE 16857, 19577, 19592, 19624 & 19632, Dale IR 2002, Leippert H 5645, Ivens GW 410 (EA).

**Commiphoraafricana(A.Rich.)Engl.var.venosa (Mattick) Govaerts** – Habit: Tree. Habitat: Bushed grassland, ca. 600 m. Vouchers: Cheseny CMC 44/74, Mrs. Robertson SA 6552 & 6556 (EA).

***Commiphorabaluensis* Engl.** – Habit: Tree. Habitat: Deciduous woodland and dry forest, often on rocky ground, 650–990 m. Vouchers: Medley KE 970, Gillett JB & Glover PE 19643, Verdcourt B & Polhill RM 2743 (EA).

**CommiphoracampestrisEngl.subsp.campestris** – Habit: Tree. Habitat: *Acacia*-*Commiphora* bushland, 550–650 m. Vouchers: Medlely KEM 775 (EA), Gillett JB & Glover PE 19623 (EA), Dale IR 3881 & 3894 (EA), Hildebrandt JM 2516 (EA), Bally PRO 8551 (EA). Near Threatened.

**CommiphoracampestrisEngl.var.heterophylla (Engl.) J.B.Gillett** – Habit: Tree: Habitat: *Acacia*-*Commiphora* bushland. Voucher: Gilbert MG & Gilbert CI 6096 (EA).

**Commiphoraedulis(Klotzsch)Engl.subsp.boiviniana (Engl.) J.B.Gillett** – Habit: Shrub. Habitat: *Acacia*-*Commiphora* and semi-evergreen bushland, 600–770 m. Vouchers: SAJIT–005399 (EA, HIB), Mwachala G et al. EW1513, Medley KE 84, Faden RB & Faden AJ 74/278, Verdcourt B 3895, Leakey PH s.n. (EA).

**Commiphoraedulis(Klotzsch)Engl.subsp.holosericea (Engl.) J.B.Gillett** – Habit: Shrub. Habitat: *Acacia*-*Commiphora* bushland, ca. 762 m. Vouchers: Dale IR 3880, Verdcourt B 1113 (EA).

**CommiphoraeminiiEngl.subsp.trifoliolata (Engl.) J.B.Gillett** – Habit: Tree. Habitat: Dry evergreen forest and forest margins, 650–1250 m. Vouchers: SAJIT–005372, Watuma BM W0243 (EA, HIB), Luke WRQ & Luke PA 4099, Medley KE 446 & 600, Faden RB, Evans A, Smeenk C & Kariuki B 131 (EA).

***Commiphorakataf* (Forssk.) Engl.** – Habit: Tree. Habitat: Montane woodland, *Acacia*-*Commiphora* bushland, 550–1024 m. Vouchers: Watuma BM W0317 (EA, HIB), Faden RB & Faden AJ 74/276, Medley KE 525, 721 & 774, Dale IR 3892, Engler A 1945 (EA).

***Commiphorakua* (R.Br. ex Royle) Vollesen** – Habit: Shrub/Tree. Habitat: *Acacia*-*Commiphora* bushland, rocky places, bushed grassland, 550–940 m. Vouchers: SAJIT–005398, Watuma BM W0185 (EA, HIB), Medley KE 523, 550 & 723, Greenway PJ & Kanuri K 12658, 12893 & 12894, Hucks M 892 (EA).

***Commiphoraoblongifolia* J.B.Gillett** – Habit: Shrub/Tree. Habitat: *Acacia*-*Commiphora* bushland in rocky places, 600–840 m. Vouchers: Luke WRQ & Luke PA 16116, Cheseny CMC 11/72, Greenway PJ & Kanuri K 12661, Gillett JB & Glover PE 17233 & 19644, Verdcourt B 3889, Napier ER 1015 (EA). Near Threatened.

***Commiphorapaolii* Chiov.** – Habit: Shrub/Tree. Habitat: *Acacia*-*Commiphora* bushland, ca. 600 m. Voucher: Verdcourt B & Polhill R 2709 (EA).

***Commiphorasamharensis* Schweinf.** – Habit: Tree. Habitat: *Acacia*-*Commiphora* bushland and woodland, 750–870 m. Voucher: Medley KE 815 (EA).

***Commiphoraschimperi* (O.Berg) Engl.** – Habit: Shrub/Tree. Habitat: *Acacia*-*Commiphora* open deciduous woodland and bushland, 600–1000 m. Vouchers: Medley KE 684, Gillett JB & Glover PE 19582, 19588 & 19639 (EA).

**F61. CACTACEAE**

2 Genera, 2 species

*Opuntiahumifusa (Raf.) Raf. – Habit: Shrub. Habitat: Dry deciduous bushland and thicket, 1019–1600 m. Vouchers: Watuma BM W0316 (EA, HIB), Beentje HJ et al. NMK Exped. 1144 (EA).

***Rhipsalisbaccifera* (J.S.Muell.) Stearn** – Habit: Shrub. Habitat: Upland rainforest, evergreen bushland, rocky cliffs, 650–1875 m. Vouchers: Watuma BM W0203 (EA, HIB), Kabuye CHS 82/155, Mungai G et al. EW2830, Wakanene KM & Mwangangi OM 272, Mbale M et al. NMK 964, Faden RB et al. NMK Exped. 227, 450 & 779, Bally PRO 8794 & 16662, Faden RB, Evans A, Smeenk C 71/1013, Gillett JB, Burtt BL & Osborn RM 17101 (EA).

**F62. CAMPANULACEAE**

2 Genera, 5 species

***Lobeliagiberroa* Hemsl.** – Habit: Shrub. Habitat: Upland secondary forest, forest edges, streamsides, 1500–2170 m. Vouchers: Kirika P et al. NMK 814, Christenhusz MJM et al. 6659, Faden RB et al. NMK Exped. 88 & 243, Gillett JB, Burtt BL & Osborn RM 17076, Bally PRO 8757, Knox EB 2694 & 2696, Dale IR 3797 (EA).

***Lobeliaholstii* Engl.** – Habit: Herb. Habitat: Upland grassland, forest edges, rocky or disturbed places, 1425–2100 m. Vouchers: Watuma BM W0017 (EA, HIB), Christenhusz MJM et al. 6631, Beentje HJ 2146, Faden RB et al. NMK Exped. 290 & 438, Drummond RB & Hemsley JH 4303, Hildebrandt JM 2463, Knox EB 2692, 2695 & 2702 (EA).

**LobeliatrullifoliaHemsl.subsp.trullifolia** – Habit: Herb. Habitat: Upland grassland, forest margins, stream sides, roadsides, 1600–1640 m. Voucher: Faden RB et al. 71/188 (EA).

**WahlenbergiakrebsiiCham.subsp.arguta (Hook.f.) Thulin** – Habit: Herb. Habitat: Forest margins, upland grassland, ca. 1250 m. Voucher: Wakanene KM & Mwangangi OM 443 (EA).

***Wahlenbergianapiformis* (A.DC.) Thulin** – Habit: Herb. Habitat: Deciduous woodland, grassland, old cultivations, roadsides, ca. 1600 m. Voucher: Beentje HJ et al. NMK Exped. 1141 & 1158 (EA).

**F63. CANELLACEAE**

1 Genus, 1 species

**WarburgiaugandensisSpraguesubsp.ugandensis** – Habit: Tree. Habitat: Lowland rainforest, secondary bushland and grassland, ca. 1219 m. Vouchers: Kishewitsch S s.n., Stephen K s.n. (EA).

**F64. CANNABACEAE**

3 Genera, 4 species

***Celtisafricana* Burm.f.** – Habit: Tree. Habitat: Understorey in mist forest, riverine, 1050–1875 m. Vouchers: Mwachala G et al. EW2567, Faden RB et al. NMK Exped. 528 (EA).

***Celtisgomphophylla* Baker** – Habit: Tree. Habitat: Mist forest, by forest streams in valleys, 1170–1850 m. Vouchers: Mwachala G et al. EW3205, Faden RB et al. NMK Exped. 775, Faden RB, Evans A, Msafiri F, Smeenk C 71/58 (EA).

***Chaetachmearistata* Planch.** – Habit: Shrub/Tree. Habitat: Lowland and upland forest, 1000–1640 m. Vouchers: Mwachala G et al. EW2579, Medley KE 619 (EA).

***Tremaorientale* (L.) Blume** – Habit: Tree. Habitat: Upland and lowland rainforest margins, 800–2000 m. Vouchers: SAJIT–005343, Watuma BM W0081 (EA, HIB), Mwachala G et al. EW2573, Faden RB et al. NMK Exped. 398, Beentje HJ et al. NMK Exped. 940, Raler I 0089, Medley KE 629 (EA).

**F65. CAPPARACEAE**

6 Genera, 27 species

***Bosciacoriacea* Pax** – Habit: Tree. Habitat: Deciduous bushland, grassland with scattered trees, 500–690 m. Vouchers: Muasya J et al. KEFRI 82, Ochung HO 63/BE, Medley KE 657, Zùmer M 38, Hildebrandt JM 2478 (EA).

***Bosciaintegrifolia* J.St.-Hil.**– Habit: Tree. Habitat: Deciduous woodland and bushland, 500–1000 m. Vouchers: Muaysa J & Medley KE 655 & 769, Gardner HM 2911 (EA).

***Bosciasalicifolia* Oliv.** – Habit: Tree. Habitat: Deciduous woodland and bushland, ca. 900 m. Voucher: Mwachala G et al. EW900 (EA).

***Cadabacarneoviridis* Gilg & Gilg-Ben.** – Habit: Shrub. Habitat: Evergreen bushland, grassland with scattered trees, 500–650 m. Vouchers: Medley KE 503, 685, 842 & 876, Ossent JR 113 (EA).

***Cadabafarinosa* Forrsk.** – Habit: Shrub. Habitat: *Acacia* thornbush, bushland, grassland with scattered trees, 500–650 m. Vouchers: Mwachala G et al. EW198, Drummond RB & Hemsley JH 4250, Ossent J 46, Gillett JB 16872, Medley KE 502 & 838 (EA).

***Cadabaglandulosa* Forssk.** – Habit: Shrub. Habitat: Deciduous bushland, grassland with scattered trees. Vouchers: Njoroge T 21, Rauh W s.n. (EA).

***Cadabalinearifolia* (J.Graham) M.R.Almeida** – Habit: Tree. Habitat: *Acacia* bushland. Vouchers: Greenway PJ & Kanuri 9786, Ossent JR 40 (EA).

***Cadabamirabilis* Gilg** – Habit: Shrub. Habitat: Deciduous bushland and semi-desert scrub, Voucher: Ochung H H012 (EA).

***Cadabaruspolii* Gilg** – Habit: Shrub. Habitat: Deciduous bushland, grassland with scattered trees. Vouchers: Wamukoya OW 103, Greenway PG & Kanuri 10 (EA).

***Cadabastenopoda* Gilg & Gilg-Ben.** – Habit: Shrub. Habitat: Deciduous bushland, riverine forest, ca. 600 m. Vouchers: Drummond RB & Hemsley JH 4276, Ossent JR 112 (EA).

***Cappariscartilaginea* Decne.** – Habit: Shrub/Tree. Habitat: Deciduous bushland, exposed rocks. Voucher: Ossent JR 702 (EA).

**CappariserythrocarposIsertvar.rosea (Klotzsch) DeWolf** – Habit: Liana. Habitat: Deciduous woodland, bushland and secondary scrub, ca. 925 m. Voucher: Mungai GM et al. EW3585 (EA).

**CapparisfascicularisDC.var.fascicularis** – Habit: Liana/Shrub. Habitat: Deciduous bushland, grassland with scattered trees, 500–1180 m. Vouchers: Mwachala G et al. EW259, Medley KE 951 (EA).

**CapparisfascicularisDC.var.scheffleri (Gilg & Gilg-Ben.) DeWolf** – Habit: Shrub. Habitat: Lowland dry evergreen forest, deciduous bushland and secondary scrub. Voucher: Ossent JR 136 (EA).

**CapparissepiariaL.var.fischeri (Pax) DeWolf** – Habit: Shrub. Habitat: Deciduous bushland, grassland with scattered trees. Voucher: Gillet JB 19579 (EA).

**CapparissepiariaL.var.stuhlmannii (Gilg) DeWolf** – Habit: Shrub. Habitat: Deciduous woodland and bushland, riverine forest. Voucher: Napper D 1469 (EA).

***Capparistomentosa* Lam.** – Habit: Shrub. Habitat: Deciduous bushland, 500–650 m. Vouchers: SAJIT–005410 (EA, HIB), Medley KE 611, 725 & 762, Gardner N 2923, Bally PRO 8640 (EA).

***Maeruaangolensis* DC.** – Habit: Shrub/Tree. Habitat: Deciduous bushland and woodland, edges of upland rainforest, 550–1000 m. Vouchers: Medley KE 504b, 827, 871 & 933 (EA).

***Maeruacrassifolia* Forssk.** – Habit: Tree. Habitat: Deciduous bushland, thicket and semi-desert scrub, 550–650 m. Vouchers: Medley KE 512 & 957, Ossent JR 66 (EA).

***Maeruadecumbens* (Brongn.) DeWolf** – Habit: Shrub. Habitat: Deciduous bushland, 550–865 m. Vouchers: Watuma BM W0200 (EA, HIB), Kimeu JM et al. KEFRI 506, Chepkwony & Esilaba 29, Medley KE 857, Bally PRO 13370, Gardner HM 3011, Ossent JR EAH 1092S & 11468 (EA).

***Maeruadenhardtiorum* Gilg** – Habit: Shrub. Habitat: Deciduous bushland, termite mounds. Voucher: Hucks M 119 (EA).

***Maeruaendlichii* Gilg & Gilg-Ben.** – Habit: Shrub. Habitat: Deciduous bushland and grassland with scattered trees or shrubs, 550–650 m. Vouchers: Medley KE 765 & 786, Leuthhold W 89, Gillett JB 16871, Bally J 13392 (EA).

***Maeruagrantii* Oliv.** – Habit: Shrub. Habitat: Deciduous woodland, bushland, grassland with scattered trees, 450–600 m. Voucher: Bally PRO 13298 (EA).

***Maeruaholstii* Pax** – Habit: Shrub. Habitat: Deciduous woodland in dry sandy places, 550–650 m. Vouchers: Medley KE 734 & 779, Ossent JR 110 (EA).

***Maeruakirkii* (Oliv.) F.White** – Habit: Shrub. Habitat: High altitude bushland, riverine forest, 600–1180 m. Vouchers: Mwachala G et al. EW286 & 1506, Kokwaro JO 2614, Faden RB & Faden AJ 74/435, Medley KE 848, Bally PRO 8746, Ossent JR 41 & 11470 (EA).

**MaeruatriphyllaA.Rich.var.calophylla (Gilg) DeWolf** – Habit: Tree. Habitat: Deciduous and evergreen bushland, riverine forest on rocky ground, 1080–1250 m. Vouchers: Wakanene KM & Mwangangi OM 488, Faden RB & Evans A 71/123 (EA).

**MaeruatriphyllaA.Rich.var.johannis (Volkens & Gilg) DeWolf** – Habit: Tree. Habitat: Deciduous bushland, grassland with scattered trees, termite mounds, 650–1371 m. Vouchers: SAJIT–005386 & 005394 (EA, HIB), Kabuye CHS 82/33, Medley KE 578, 599, 685, 731, 739 & 896 (EA).

***Ritchieaalbersii* Gilg** – Habit: Tree. Habit: Upland rainforest, often at margins, 1000–1875 m. Vouchers: SAJIT–006385, Watuma BM W0132 (EA, HIB), Raler I 0087, Faden RB et al. 71/127 & 72/208, Medley KE 1011 (EA).

***Thilachiumafricanum* Lour.** – Habit: Shrub/Tree. Habitat: Deciduous woodland, bushland and thicket, 550–1180 m. Vouchers: SAJIT–004633 (EA, HIB), Mwachala G et al. EW273, 2591 & 3312, Wakanene KM & Mwangangi OM 713, Medley KE 431, 853 & 964, Joanna B 9017, Gardner HM 2995, Dyson WG 538, Forest Dept. 2995 (EA).

***Thilachiumthomasii* Gilg** – Habit: Shrub/Tree. Habitat: Deciduous woodland and bushland, 550–1000 m. Vouchers: Mwachala G, Nyaboke B & Matheka K 1031, Kirika 1, Faden RB & Faden AJ 74/235, Agnew ADQ & Part 1 Botany 7341, Mildbraed 17, Murray High School 106, Medley KEM s.n. (EA).

**F66. CARYOPHYLLACEAE**

4 Genera, 4 species

***Cerastiumlanceolatum* (Poir.) Volponi** – Habit: Herb. Habitat: Forest margins & glades, bushland, roadsides, weed of cultivated areas, 1371–2100 m. Vouchers: Napier ER 1130, Kluguess LM 59, Drummond RB Hemsley JH 4283, Lavranos JJ 11921 (EA).

***Drymariacordata* (L.) Willd. ex Roem. & Schult.** – Habit: Herb. Habitat: Forest and bushland margins, roadsides, cultivated lands, 1160–2170 m. Vouchers: SAJIT–006366, Watuma BM W0107 (EA, HIB), Mwachala G et al. EW3552, Faden RB et al. NMK Exped. 40, 126 & 435, Beentje HJ 2141, Napier ER 1113, Gillett JB, Burtt BL & Osborn RM 17068 (EA).

***Pollichiacampestris* Aiton** – Habit: Shrub. Habitat: Open woodland, bushland, waste places, ca. 1400 m. Voucher: Gilbert MG 5823 (EA).

***Silenemacrosolen* Steud. ex A.Rich.** – Habit: Herb. Habitat: Grassland, rocky places, 1430–1829 m. Vouchers: Mungai GM et al. EW3095A, Gardner HM 2952 (EA).

**F67. CELASTRACEAE**

8 Genera, 15 species

***Elaeodendronbuchananii* (Loes.) Loes.** – Habit: Tree. Habitat: Evergreen forest, deciduous woodland, sometimes secondary forest, 1700–1875 m. Vouchers: Faden RB et al. NMK Exped. 544, Raler I 0126 (EA).

***Elaeodendronschlechterianum* (Loes.) Loes.** – Habit: Tree. Habitat: Dry deciduous woodland, fringing forest, 640–1000 m. Vouchers: Medley KE 642, 718, 773, 825, 837 & 1039 (EA).

***Elaeodendronschweinfurthianum* (Loes.) Loes.** – Habit: Tree. Habitat: Deciduous and semi-evergreen woodland, 650–1000 m. Vouchers: Watuma BM W0003 (EA, HIB), Medley KE 791 & 915 (EA).

***Gymnosporiagracilis* Loes.** – Habit: Shrub. Habitat: Evergreen forest margins, thickets, 600–1402 m. Vouchers: Luke WRQ et al. 5284, Faden RB, Evans A, Msafiri F et al. 70/960, Medley KE 451, 452 & 472, Joanna B 8868 (EA).

***Gymnosporiaheterophylla* (Eckl. & Zeyh.) Loes.** – Habit: Shrub/Tree. Habitat: 650–1300 m. Vouchers: SAJIT–005351 & 005405 (EA, HIB), Faden RB et al. 71/118, Medley KE 522, 525, 526, 626 & 720 (EA).

***Gymnosporiaputterlickioides* Loes.** – Habit: Shrub. Habitat: Dry deciduous woodland, wooded grassland often in rocky places, 650–1000 m. Voucher: Medley KE 738 (EA).

***Gymnosporiasenegalensis* (Lam.) Loes.** – Habit: Tree. Habitat: Deciduous woodland, thickets and scrub, 650–2170 m. Vouchers: SAJIT–006354 (EA, HIB), Mwachala G et al. EW384, 523, 773, 909, 3145, Mungai GM et al. EW3579, Wakanene KM & Mwangangi OM 460, Faden RB et al. NMK Exped. 122, Medley KE 510, 586 & 636, Hucks M 820 (EA).

***Loeseneriellaafricana* (Willd.) R.Wilczek** – Habit: Liana. Habitat: Moist evergreen forest, forest margin, riverine forest. 900–1300 m. Vouchers: SAJIT–005393 (EA, HIB), Mungai G et al. EW1406, Hildebrandt JM 2581 (EA).

***Maytenusacuminata* (L.f.) Loes.** – Habit: Tree. Habitat: Understorey and margin of upland of riverine forest, 1605–1902 m. Vouchers: SAJIT–005330 (EA, HIB), Mwachala G, Nyaboke & Saidi S 1044, Faden RB et al. 71/52, Drummond RB & Hemsley JH 4357 (EA).

***Maytenusundata* (Thunb.) Blakelock** – Habit: Tree. Habitat: Forest, evergreen bushland and woodland, 800–1050 m. Vouchers: SAJIT–004631 (EA, HIB), Medley KE 717, 748, 803, 866 & 891 (EA).

***Mystroxylonaethiopicum* (Thunb.) Loes.** – Habit: Tree. Habitat: Evergreen forest, fringing forest, open woodland. 800–1875 m. Vouchers: Faden RB et al. NMK Exped. 543, Raler I 0128, Medley KE 632, 668, 898 & 932 (EA).

***Pleurostyliaafricana* Loes.** – Habit: Tree. Habitat: Forest margins, deciduous woodland, termite mounds on rocky hillsides, 800–950 m. Vouchers: Kabuye CHS et al. NMK Exped. 612, Mungai GM et al. EW1294 (EA).

***Pristimeragoetzei* (Loes.) R.H.Archer**– Habit: Liana. Habitat: Evergreen forest, 1400–1814 m. Vouchers: SAJIT–004515 (EA, HIB), Faden RB et al. NMK Exped. 5, Faden RB & Faden AJ 71/1011, Raler I 0036 (EA).

***Salaciamadagascariensis* (Lam.) DC.** – Habit: Shrub/Liana. Habitat: Lowland evergreen forest, deciduous woodland, 1350–1450 m. Voucher: SAJIT–005339 (EA, HIB).

***Salaciastuhlmanniana* Loes.** – Habit: Shrub/Liana. Habitat: Rainforest, riverine, deciduous woodland, 650–1000 m. Vouchers: Cheseny CMC 72/1, Medley KE 946, 947 & 975 (EA).

**F68. CLEOMACEAE**

1 Genus, 6 species

***Cleomebriquetii* Polhill** – Habit: Herb. Habitat: Deciduous bushland and thickets on rocky ground, 500–823 m. Vouchers: Napier ER 978, Gillett JB 17231, Polhill RM & Kibuwa SP 958 (EA).

***Cleomegynandra* L.** – Habit: Herb. Habitat: Deciduous bushland, waste, disturbed and cultivated lands. Voucher: Hucks M 711 (EA).

***Cleomehirta* (Klotzsch) Oliv.** – Habit: Herb. Habitat: Deciduous bushland and grassland, 550–700 m. Vouchers: Polhill RM & Kibuwa SP 934, Rauh W Ke 57, Ossent JR 24, MacDonald J 866, Hucks M 707 (EA).

***Cleomemonophylla* L.** – Habit: Herb. Habitat: Grassland, deciduous woodland and bushland, roadside, disturbed areas, 1371–1580 m. Vouchers: Kabuye CHS 82/112, Napier ER 1102 (EA).

***Cleomesilvatica* Gilg & Gilg-Ben.** – Habit: Herb. Habitat: Upland rainforest clearings, rocky grasslands & roadsides. Voucher: Gardiner N 2932 (EA).

***Cleomeusambarica* Pax** – Habit: Herb. Habitat: Bushland and thicket, streamside, cultivated ground. Voucher: Joanna B 8843 (EA).

**F69. CLUSIACEAE**

1 Genus, 1 species

***Garciniavolkensii* Engl.** – Habit: Tree. Habitat: Understorey in evergreen forest, 1000–1900 m. Vouchers: SAJIT–003292 & 005320, Watuma BM W0162 (EA, HIB), Kabuye CHS 82/52, Faden RB et al. NMK Exped. 304 & 400, Beentje HJ et al. NMK Exped. 1115, De BlocK P et al. 243, 299, 316 & 490, Faden RB et al. 71/129, 226, 979 & 77/319, Drummond RB & Hemsley JH 4344, Medley KE 793 & 997, Bally PRO 13624, Gardner HM 2967, Joanna B 9401, Raler I 0026 (EA).

**F70. COMBRETACEAE**

2 Genera, 12 species

***Combretumaculeatum* Vent.** – Habit: Shrub. Habitat: Deciduous bushland and woodland, grassy slopes, 580–750 m. Vouchers: Medley KE 457, Faden RB & Faden AJ 74/260, Hooper SS & Townsend CC 1260 (EA).

***Combretumexalatum* Engl.** – Habit: Shrub/Tree. Habitat: *Acacia*-*Commiphora* bushland, thicket and wooded grassland, 575–1000 m. Vouchers: Watuma BM W0028 (EA, HIB), Kabuye CHS et al. NMK Exped. 740, Mwachala G et al. EW554, Mungai G et al. EW1767, Kuchar P, Msafiri F & Karime N 5695, Medley KE 1019, Hildebrandt JM 2561 (EA).

**CombretumhereroenseSchinzsubsp.hereroense** – Habit: Tree. Habitat: Wooded grassland, *Acacia*-*Commiphora* bushland, ca. 700 m. Voucher: Mwachala G et al. EW3298 (EA).

**CombretumhereroenseSchinzvar.parvifolium (Engl.) Wickens** – Habit: Tree. Habitat: *Acacia*-*Commiphora* bushland, wooded grassland. Voucher: Ossent JR 133 (EA).

**CombretumhereroenseSchinzsubsp.volkensii (Engl.) Wickens** – Habit: Shrub/Tree. Habitat: *Acacia*-*Commiphora* bushland, wooded grassland, 600–950 m. Vouchers: Mwachala G et al. EW858, Medley KE 547, Ossent JR 145 (EA).

***Combretummolle* R.Br. ex G.Don** – Habit: Tree. Habitat: Wooded grassland and bushland, 600–1250 m. Vouchers: Watuma BM W0008 (EA, HIB), Mwachala G et al. EW220 & 283, Wakanene KM & Mwangangi OM 450, Medley KE 637 & 780, Bally PRO 17123 (EA).

***Combretummossambicense* (Klotzsch) Engl.** – Habit: Shrub/Tree. Habitat: Deciduous and secondary bushland, wooded grassland, 610–950 m. Vouchers: Mungai G et al. EW1765, Lavranos JJ 12340A, Gardner HM 1198 (EA).

***Combretumpsidioides* Welw.** – Habit: Tree. Habitat: Deciduous woodland, rocky hill slopes, ca. 1300 m. Voucher: Mungai G et al. EW1322 (EA).

***Terminaliabrownii* Fresen.** – Habit: Tree. Habitat: Deciduous woodland, bushland, grassland, riverine forest, 650–1200 m. Vouchers: SAJIT–005367, Watuma BM W0009 (EA, HIB), Mwachala G et al. EW72, 949 & 1556, Wakanene KM & Mwangangi OM 483, Medley KE 419 & 854 (EA).

***Terminaliakilimandscharica* Engl.** – Habit: Tree. Habitat: Deciduous woodland, bushland and wooded grassland, rocky outcrops, 548–1200 m. Vouchers: Greenway PJ & Kanuri K 12779, Small R 823 (EA).

***Terminaliaorbicularis* Engl. & Diels** – Habit: Tree. Habitat: *Acacia*-*Commiphora* bushland, 520–914 m. Vouchers: Faden RB & Faden AJ 74/245 & 515, Dale IR 3896, Agnew ADQ & Part 1 Botany 7316, Verdcourt B Ke19, Rauh W 12521, Ossent JR 147 & 1195 (EA).

***Terminaliaparvula* Pamp.** – Habit: Shrub/Tree. Habitat: *Acacia*-*Commiphora* bushland, 426–853 m. Vouchers: Greenway PJ & Kanuri K 12641 & 12919 (EA).

***Terminaliaprunioides* M.A.Lawson** – Habit: Shrub/Tree. Habitat: *Acacia*-*Commiphora* and *Acacia*-*Combretum* bushland, 550–1067 m. Vouchers: Mwachala G et al. EW975, Mungai GM et al. EW1769, Musya J & Safary 28, Faden RB & Faden AJ 74/473, Medley KE 427, 652 & 841, Napier ER 897, Dale IR 2005, Ossent JR 158, Greenway PJ & Kanuri K 12640, Mr & Mrs Hucks HP 318 (EA).

***Terminaliaspinosa* Engl.** – Habit: Tree. Habitat: *Acacia*-*Commiphora* bushland, 500–620 m. Vouchers: Medley KE 710, 759 & 840, Bally PRO 17121 (EA).

**F71. CONNARACEAE**

2 Genera, 2 species

***Agelaeapentagyna* (Lam.) Baill.** – Habit: Liana. Habitat: Upland rainforest, riparian forest, 1275–1850 m. Vouchers: SAJIT–006383 (EA, HIB), Mwachala G et al. EW1869, Drummond RB & Hemsley JH 4367, Bally PRO 8783, Joanna B 9079, Beentje HJ et al. NMK Exped. 866 & 1014, Raler I 0137 (EA), Faden RB et al. 71/44 (EA).

***Roureathomsonii* (Baker) Jongkind** – Habit: Liana. Habitat: Montane forest remnants, upland rainforest, 1400–2000 m. Vouchers: Watuma BM W0143 (EA, HIB), Drummond RB & Hemsley JH 4337 & 4375, Joanna B 8998 (EA).

**F72. CONVOLVULACEAE**

7 Genera, 19 species

**Astripomoeahyoscyamoides(Vatke)Verdc.var.hyoscyamoides** – Habit: Subshrub. Habitat: Deciduous bushland, roadside, cultivated or disturbed ground, ca. 600 m. Voucher: Hucks M 576 (EA).

**CuscutaplanifloraTen.var.holstii Baker & Rendle** – Habit: Climber. Habitat: On herbs in grasslands, ca. 1710 m. Voucher: Gardner HM 2964 (EA).

***Hildebrandtiasepalosa* Rendle** – Habit: Shrub. Habitat: *Acacia*-*Commiphora*-*Euphorbia*-*Sanseveria* bushland, 500–650 m. Vouchers: Faden RB & Faden AJ 74/461, Ossent JR 10, 50 & 54, Lubai lK HB20 (EA).

***Ipomoeabiflora* (L.) Pers.** – Habit: Climber. Habitat: Dry bush, woodland, grassland, cultivated lands, 1056 m. Voucher: Watuma BM W0158 (EA, HIB).

***Ipomoeabullata* Oliv.** – Habit: Climber. Habitat: Dry bushland, 548–914 m. Vouchers: Joanna B 8877, Ossent JR 126, Bally PRO 74 & 8613, Rauh W 840 (EA).

***Ipomoeaeriocarpa* R.Br.** – Habit: Climber. Habitat: Grassland, hedgerows, cultivated lands, ca. 610 m. Voucher: Napier ER 1049 (EA).

***Ipomoeahartmannii* Vatke** – Habit: Herb. Habitat: *Acacia*-*Commiphora* deciduous bushland, 600–950 m. Vouchers: Kabuye CHS et al. NMK Exped. 603, Verdcourt B & Polhill RM 2763, Hildebrandt JM 2564, Ossent JR 10952 (EA).

**IpomoeahildebrandtiiVatkesubsp.hildebrandtii** – Habit: Shrub. Habitat: *Acacia*-*Commiphora* deciduous bushland, 914–1371 m. Vouchers: Watuma BM W0151 (EA, HIB), Napier ER 1078, Bally PRO 8117, Verdcourt B & Polhill RM 2739 & 2744, Irwin PH 156 (EA).

***Ipomoeainvolucrata* P.Beauv.** – Habit: Herb. Habitat: Forest, secondary forest, abandoned cultivations, 2000–2170 m. Vouchers: SAJIT–006360 (EA, HIB), Faden RB et al. NMK Exped. 120, Gillett JB et al. 17096 (EA).

**IpomoeakituiensisVatkevar.kituiensis** – Habit: Herb/Climber. Habitat: Deciduous bushland and woodland, 650–950 m. Vouchers: Watuma BM W0001 (EA, HIB), Mwachala G et al. EW847, Medley KE 420 (EA).

**IpomoeamombassanaVatkevar.mombassana** – Habit: Climber. Habitat: *Commiphora*, *Combretum* bushland and woodland, secondary bushland, ca. 610 m. Vouchers: Ossent JR 11003, Jeffrey GW 815 (EA).

***Ipomoeaochracea* (Lindl.) G.Don** – Habit: Climber. Habitat: Forest clearings, thickets, rocky places, ca. 950 m. Voucher: Mwachala G et al. EW766 (EA).

**Ipomoeapes-tigridisL.var.pes-tigridis** – Habit: Climber. Habitat: Grassland, bushland and plantations, ca. 610 m. Voucher: Napier ER 970 (EA).

**Ipomoeapes-tigridisL.var.longibracteata Vatke** – Habit: Climber. Habitat: Deciduous bushland and thickets. Vouchers: Hildebrandt JM 2420, Bally PRO 8801 (EA).

***Ipomoea* sp. A of FTEA** – Habit: Climber. Habitat: Woodland, ca. 866 m. Voucher: Mwachala G, Nyaboke B & Matheka K 1059 (EA).

***Ipomoeastenosiphon* Hallier f.** – Habit: Liana. Habitat: *Acacia*-*Commiphora* deciduous bushland, rocky areas, ca. 1219 m. Voucher: Napier ER 1125 (EA).

**Ipomoeawightii(Wall.)Choisyvar.kilimandschari (Dammer) Verdc.** – Habit: Climber. Habitat: Open forest and scrub, 800–1550 m. Vouchers: Kabuye CHS et al. NMK Exped. 613, Mwachala G et al. EW3055A, Beentje HJ et al. NMK Exped. 845, Klungness LM 5 (EA).

***Lepistemonowariensis* (P.Beauv.) Hallier f.** – Habit: Climber. Habitat: Rainforest, riverine forest, 1100–1220 m. Vouchers: Mwachala G et al. EW2572, Napier ER 1138 (EA).

***Metaporanadensiflora* (Hallier f.) N.E.Br.** – Habit: Liana. Habitat: Secondary evergreen bushland, abandonded cultivated ground, 650–1000 m. Vouchers: Kimeu JM, Meso M & Wafula A KEFRI 507, Medley KEM 636 (EA).

***Stictocardiaincomta* (Hallier f.) Hallier f.** – Habit: Climber. Habitat: *Terminalia*-*Combretum*-*Acacia* bushland on rocky slopes, 500–950 m. Vouchers: Kabuye CHS et al. NMK Exped. 613, Hucks M 1074 (EA).

**F73. CRASSULACEAE**

3 Genera, 13 species

***Cotyledonbarbeyi* Schweinf. ex Baker** – Habit: Herb. Habitat: Rocky slopes, termite mounds in *Acacia* bushland, 1000–1330 m. Vouchers: Mungai GM et al. EW3092A, Bally PRO 13404 (EA).

**Crassulaalata(Viv.)A.Bergersubsp.pharnaceoides (Fisch. & C.A.Mey.) Wickens & Bywater** – Habit: Herb. Habitat: Moist rock crevices, stream banks, ca. 1600 m. Voucher: Mwachala G et al. EW3136 (EA).

***Crassulaalba* Forssk.** – Habit: Herb. Habitat: Rock crevices and rocky outcrops, 1270–1600 m. Vouchers: Mwachala G et al. EW3158, Beentje HJ et al. NMK Exped. 1139 (EA).

***Crassulaalsinoides* (Hook.f.) Engl.** – Habit: Herb. Habitat: Upland forest margins, moist grounds, streams, 1700–1950 m. Vouchers: SAJIT–004543 & 006405 (EA, HIB), Faden RB et al. NMK Exped. 899, Gillett JB & Burtt BL 17066, Mwachala G s.n. (EA).

***Crassulaglobularioides* Britten** – Habit: Herb. Habitat: Rock crevices. Vouchers: Bally PRO 13525, Joanna B 9062 (EA).

**CrassulaschimperiFisch. & C.A.Mey.subsp.phyturus (Mildbr.) R.Fern.** – Habit: Herb. Habitat: Rocks in upland forest and grassland, 1400–1640 m. Voucher: Faden RB et al. NMK Exped. 2 (EA).

**CrassulaschimperiFisch. & C.A.Mey.subsp.schimperi** – Habit: Herb. Habitat: Moist rocks, rock crevices, epiphyte on tree trunks, ca. 1800 m. Voucher: Kabuye CHS 82/61 (EA).

**Crassulavolkensiisubsp.coleae (Baker) Wickens & Bywater** – Habit: Herb. Habitat: Moist thicket, volcanic rock crevices, 980–1371 m. Vouchers: Mungai GM et al. EW2650, Faden RB & Faden AJ 74/486, Napier ER 756 (EA).

**CrassulavolkensiiEngl.subsp.volkensii** – Habit: Herb. Habitat: Shady rocky ground, 950–2000 m. Vouchers: Kabuye CHS 82/65, Mwachala G et al. EW1274, Luke WRQ & Luke PA 6413, Wakanene KM & Mwangangi OM 324, Bally PRO 13414, Gardner HM 3013 (EA).

***Kalanchoe×auriculata* (Raadts) V.V.Byalt** – Habit: Herb. Habitat: Dense or open bushland on rocky slopes, ca. 914 m. Voucher: Magius in EA Polhill RM 404 (EA).

***Kalanchoedensiflora* Rolfe** – Habit: Herb. Habitat: Upland rainforest margins, glades and riverbanks, 1450–1800 m. Vouchers: Kabuye CHS 82/106, Wakanene KM & Mwangangi OM 236 & 641, Bytebier B 1280, Faden RB et al. NMK Exped. 495 (EA).

***Kalanchoelateritia* Engl.** – Habit: Herb. Habitat: Deciduous and semi-deciduous woodland and forest, 760–1150 m. Vouchers: Kabuye CHS et al. NMK Exped. 651, Mungai G et al. EW2659, Faden RB & Faden AJ 74/535, Faden RB, Evans A & Githui M 70/150 (EA).

***Kalanchoeminiata* Hilsenb. & Bojer ex Tul.** – Habit: Herb. Habitat: Wooded grassland, bushland and scrub, ca. 1060 m. Voucher: Bally PRO 13533 (EA).

***Kalanchoenyikae* Engl.** – Habit: Herb. Habitat: Deciduous bushland, 760–1040 m. Vouchers: Mungai GM et al. EW2657, Faden RB et al. 70/151 (EA).

***Kalanchoeprittwitzii* Engl.** – Habit: Herb. Habitat: Forest, open bush and grassland, ca. 1350 m. Voucher: Mwachala G et al. EW2666 (EA).

**F74. CUCURBITACEAE**

13 Genera, 29 species

***Citrulluslanatus* (Thunb.) Matsum. & Nakai** – Habit: Climber. Habitat: Grassland and bushland, old plantations and cultivation, 610–1150 m. Voucher: Ogola B & Mwangangi OM et al. 25 & 26 (EA), Napier ER 1048 (EA).

***Cocciniagrandiflora* Cogn.** – Habit: Climber. Habitat: Upland rainforest, 1425–1850 m. Vouchers: Watuma BM W0209 (EA, HIB), Faden RB et al. NMK Exped. 423 & 799, Beentje HJ et al. NMK Exped. 879, Bally 13759 (EA).

***Cocciniagrandis* (L.) Voigt** – Habit: Climber. Habitat: Deciduous bushland, woodland & wooded grassland, riverine, 600–1250 m. Vouchers: Beentje HJ et al. NMK Exped. 423, Muchelle WN HB 16/94, Sanganyi SK HB 14, Joanna B 8929 (EA).

***Cocciniamicrophylla* Gilg** – Habit: Climber. Habitat: Deciduous dry bushland and *Combretum* wooded grassland, ca. 950 m. Vouchers: Mungai GM et al. EW1318 & 1319 (EA).

***Cocciniatrilobata* (Cogn.) C.Jeffrey** – Habit: Climber. Habitat: Wooded grassland, deciduous bushland, 685–823 m. Vouchers: SAJIT–005338 (EA, HIB), Polhill RM & Kibuwa SP 962, Verdcourt B 3888 (EA).

***Corallocarpusepigaeus* (Rottler) Hook.f.** – Habit: Climber. Habitat: Margins of upland forest, wooded grassland, deciduous bushland, 600–800 m. Vouchers: Verdcourt B 1111 (EA), Hucks M 554 (EA).

***Cucumisdipsaceus* Ehrenb. ex Spach** – Habit: Climber. Habitat: Bushland, woodland, wooded grassland, weed of cultivation, 610–1540 m. Vouchers: Wakanene KM & Mwangangi OM 331, Bernard O & Mwangangi OM et al. 8, 21 & 23, Napier ER 967 (EA).

***Cucumisficifolius* A.Rich.** – Habit: Climber: Habitat: Upland grassland, 1250–1600 m. Vouchers: Beentje HJ et al. NMK Exped. 1154, Greenway PJ 8951 (EA).

***Cucumismaderaspatanus* L.** – Habit: Climber. Habitat: Riverine margins, seasonal swamps, damp woodland and bushland. Vouchers: Lubai lK HB13, Muchelle WN HB14/94 (EA).

***Diplocyclospalmatus* (L.) C.Jeffrey** – Habit: Climber. Habitat: Rain and ground water forest, seasonal swamp grasslands, ca. 1170 m. Voucher: Mwachala G et al. EW3208 (EA).

**^NE^*Gerrardanthusgrandiflorus* Cogn.** – Habit: Herb. Habitat: Upland and lowland rainforest, 640–1850 m. Vouchers: SAJIT–004628 (EA, HIB), Luke WRQ & Luke PA 16118, Faden RB et al. NMK Exped. 1121B & 1216, Faden RB & Faden AJ 74/234 (EA). Near Threatened.

***Gerrardanthuslobatus* (Cogn.) C.Jeffrey** – Habit: Climber. Habitat: Deciduous bushland, woodland and wooded grassland. 600–850 m. Vouchers: Mwachala G et al. EW3307, Faden RB & Faden AJ 74/446, Polhill RM & Kibuwa SP 939, Archer PG 567 (EA).

***Kedrostisfoetidissima* (Jacq.) Cogn.** – Habit: Climber. Habitat: Rainforest margins, semi-evergreen woodland and bushland, wooded grassland, 600–950 m. Vouchers: Watuma BM W0173 (EA, HIB), Mwachala G et al. EW262, 1171 & 1225, Verdcourt B 3887, Napier ER 922, Hucks M 795 (EA).

***Kedrostisleloja* (J.F.Gmel.) C.Jeffrey** – Habit: Climber. Habitat: Deciduous bushland, thicket woodland, 600–700 m. Vouchers: Ossent JR 118, Hucks M 560 (EA).

***Kedrostispseudogijef* (Gilg) C.Jeffrey** – Habit: Liana. Habitat: Deciduous bushland and woodland, 609–800 m. Vouchers: Faden RB & Faden AJ 74/253, Drummond RB & Hemsley JH 4414, Verdcourt B 3888A, Gardner HM 2587, Hucks M 444 (EA).

***Lagenariasphaerica* (Sond.) Naudin** – Habit: Climber. Habitat: Riverine, ground water forest, old cultivations, 1000–1665 m. Vouchers: Watuma BM W0117 (EA, HIB), Mwachala G et al. EW267, Ogola B & Mwangangi OM 3, Napier ER 969, Joanna B et al. 9104 (EA).

***Momordicaboivinii* Baill.** – Habit: Climber. Habitat: Deciduous bushland and woodland, 998–1370 m. Vouchers: SAJIT–004626 (EA, HIB), Kabuye CHS 82/8 & 144, Mwachala G et al. EW3162, Mungai GM et al. EW3170 & 3583, Cheseny CMC 72/3, Napier ER 1095, Joanna B 9100, Fleuret A 7 (EA).

***Momordicacalantha* Gilg** – Habit: Climber. Habitat: Secondary forest undergrowth and margins on steep rocky hill, 1550–1580 m. Voucher: Watuma BM W0257 (EA, HIB).

***Momordicafoetida* Schumach.** – Habit: Climber. Habitat: Forest edges and clearings, riverine forests, secondary thickets, 1270–1814 m. Vouchers: SAJIT–006409, Watuma BM W0118 (EA, HIB), Mwachala G et al. EW3193 & 3203, Ogola B & Mwangangi OM et al. 23 (EA).

***Momordicafriesiorum* (Harms) C.Jeffrey** – Habit: Climber. Habitat: Upland rainforest, margins and clearings, 1851–2170 m. Vouchers: SAJIT–006368 (EA, HIB), Faden RB et al. NMK Exped. 121 (EA).

***Momordicarostrata* Zimm.** – Habit: Climber. Habitat: Deciduous bushland, thicket, woodland and wooded grassland, 579–1039 m. Vouchers: Watuma BM W0312 (EA, HIB), Mwachala G et al. EW265, Muasya J et al. KEFRI 78, Verdcourt B & Polhill RM 2736, Ossent JR 27 & 45, Napier ER 1023 (EA).

***Momordicaspinosa* (Gilg) Chiov.** – Habit: Shrub/Climber. Habitat: Deciduous bushland, 420–600 m. Vouchers: Faden RB & Faden AJ 74/285, Napier ER 1026, Hucks M 698 & 876, Joanna B 9070 (EA).

***Oreosyceafricana* Hook.f.** – Habit: Climber. Habitat: Disturbed rainforests, edges and clearings, 1500–2170 m. Vouchers: SAJIT–004525 (EA, HIB), Faden RB et al. NMK Exped. 95, Beentje HJ et al. NMK Exped. 926, Joanna B 9103 (EA).

***Peponiumvogelii* (Hook.f.) Engl.** – Habit: Climber. Habitat: Upland rainforest, ground water forest, woodland and bushland near open water, 750–1875 m. Vouchers: SAJIT–006394, Watuma BM W0209 & W0267 (EA, HIB), Faden RB et al. NMK Exped. 221 & 463, Gillett JB 17230 (EA).

***Trochomeriamacrocarpa* (Sond.) Hook.f.** – Habit: Climber. Habitat: Deciduous woodland and bushland, 859–1200 m. Vouchers: SAJIT–006420 (EA, HIB), Mwachala G et al. EW1443, Joanna B & Opiko 9122 (EA).

***Zehneriaemirnensis* (Baker) Keraudren** – Habit: Climber. Habitat: Rainforest, 1700–1875 m. Vouchers: Gillett JB, Burtt BL & Osborn RM 17103 (EA), Faden RB et al NMK Exped 259 (EA).

***Zehneriascabra* (L.f.) Sond** – Habit: Climber. Habitat: Rainforest, moist places in deciduous bushland, roadside, cultivations, 1115–2000 m. Vouchers: Watuma BM W0265 (EA, HIB), Mungai GM et al. EW1320, Luke WRQ & Luke PA et al. 5306 & 5385, Drummond RB & Hemsley JH 4326, Klungness LM 41 (EA).

***Zehneriaoligosperma* C.Jeffrey** – Habit: Climber. Habitat: Rainforest, moist places in deciduous bushland, 1800–1950 m. Voucher: Drummond RB & Hemsely JH 4333 (EA).

**^E^*Zehneriatuberifera* G.W.Hu & Q.F.Wang** – Habit: Climber. Habitat: Understorey and at forest edges of moist forests, 1700–1950 m. Vouchers: SAJIT–006350, Watuma BM W0044, W0298 & W0299 (EA, HIB), Faden RB et al. NMK Exped. 553 (EA).

**F75. DICHAPETALACEAE**

1 Genus, 2 species

**^NE^*Dichapetalumeickii* Ruhland** – Habit: Liana. Habitat: Upland wet evergreen forest, 1400–1875 m. Vouchers: SAJIT–005327 (EA, HIB), Kabuye CHS 84/73, Mwachala G & Nyaboke 1042, Breteler FJ 7508, Christenhusz MJM et al. 6635, Faden RB et al. NMK Exped. 324 & 489, Beentje HJ et al. NMK Exped. 951, Faden RB et al. 71/21 & 891, Drummond RB & Hemsley JH 4330, Raler I 0097 (EA).

***Dichapetalumruhlandii* Engl.** – Habit: Liana. Habitat: Forest, secondary regrowth on rocky sites, bushland, 1500–1600 m. Vouchers: Watuma BM W0137 (EA, HIB), Breteler FJ 7506, Bally PRO 8775 (EA).

**F76. DIDIEREACEAE**

1 Genus, 1 species

***Calyptrothecataitense* (Pax & Vatke) Brenan** – Habit: Shrub. Habitat: Deciduous bushland, open woodland and grassland with *Acacia*, 500–650 m. Vouchers: Medley KE 877, Hilderbrandt JM 2577 (EA).

**F77. EBENACEAE**

2 Genera, 7 species

**Diospyrosabyssinica(Hiern)F.Whitesubsp.abyssinica** – Habit: Tree. Habitat: Fringing and dry evergreen forest, open montane bushland and woodland, 900–1875 m. Vouchers: Christenhusz MJM et al. 6660, Faden RB et al. NMK Exped. 241, Kimuzi D 4, Medley KE 675, Raler I 0056 (EA).

***Diospyrosconsolatae* Chiov.** – Habit: Shrub/Tree. Habitat: Evergreen dry forest, *Acacia*, *Terminalia*, *Combretum* woodland, 600–1200 m. Vouchers: SAJIT–005401 (EA, HIB), Kabuye CHS et al. NMK Exped. 626, Wakanene KM & Mwangangi OM 523, Medley KE 430 & 847, Dale IR 3883 (EA).

***Diospyrosmespiliformis* Hochst. ex A.DC.** – Habit: Tree. Habitat: Fringing forest, *Combretum* woodland, deciduous bushland, 550–650 m. Voucher: Medley KE 460 (EA).

***Diospyrosnatalensis* (Harv.) Brenan** – Habit: Tree. Habitat: Moist semi-deciduous forest, 650–1400 m. Vouchers: Faden RB et al. 71/136, Medley KE 892, Joanna B 8865 (EA).

***Diospyroszombensis* (B.L.Burtt) F.White** – Habit: Tree. Habitat: *Euphorbia*, *Terminalia*, *Combretum* and *Acacia* woodland, rocky slopes, 800–950 m. Vouchers: Kabuye CHS et al. NMK Exped. 624, Joanna B 8766 (EA).

***Eucleadivinorum* Hiern** – Habit: Shrub/Tree. Habitat: Evergreen forest margins on rocky slopes, secondary forest, open bushland, 450–1900 m. Vouchers: Mwachala G et al. EW509, 1246 & 3254, Mungai GM et al. EW1430, Wakanene KM & Mwangangi OM 451, Christenhusz MJM et al. 6681, Medley KE 471, 615 & 912, Bally PRO 13297 (EA).

**EuclearacemosaL.subsp.schimperi (A.DC.) F.White** – Habit: Shrub/Tree. Habitat: Bushland, grassland with scattered trees, 550–650 m. Vouchers: Medley KE 435 & 763 (EA).

**F78. ERICACEAE**

2 Genera, 3 species

***Agaristasalicifolia* (Lam.) G.Don** – Habit: Tree. Habitat: Forest remnants and margins, 1400–2170 m. Vouchers: SAJIT–004576 (EA, HIB), Wakanene KM & Mwangangi OM 664, Faden RB et al. NMK Exped. 31 & 92, Joanna B 9091, Faden RB et al. 70/573, Gardner HM R2977 (EA).

***Ericaarborea* L.** – Habit: Shrub. Habitat: Above forest on mountains, open forest especially in rocky areas, 1450–1750 m. Vouchers: Wakanene KM & Mwangangi OM 312 & 637 (EA).

**Ericamannii(Hook.f.)Beentjesubsp.usambarensis (Alm & T.C.E.Fr.) Beentje** – Habit: Shrub. Habitat: Rocky forest edges, hill forest clearings and secondary association, 1000–2170 m. Vouchers: Mungai GM et al. EW3559, Faden RB et al. NMK Exped. 27 & 107, Medley KE 1018, Gillett JB et al. 17095, Raler I 0070, Joanna B 8874 (EA).

**F79. ERYTHROXYLACEAE**

1 Genus, 1 species

***Erythroxylumemarginatum* Thonn.** – Habit: Shrub/Tree. Habitat: Evergreen and mist forest, 900–1875 m. Vouchers: SAJIT–005417 (EA, HIB), Faden RB, Evans A & Githui M 70/178, Faden RB et al. NMK Exped. 516, Medley KE 475, 623, 645 & 908, Dale IR 3822 (EA).

**F80. EUPHORBIACEAE**

13 Genera, 55 species

**AcalyphafruticosaForssk.var.eglandulosa Radcl.-Sm.** – Habit: Shrub. Habitat: Montane forest and associated woodland, deciduous bushland, 580–1776 m. Vouchers: Mwachala G et al. EW29, Medley KE 983, Christenhusz MJM et al. 6637 (EA).

***Acalyphapaniculata* Miq.** – Habit: Herb. Habitat: Forests, clearings and edges, disturbed areas, ca. 1250 m. Voucher: Mwachala G et al. EW2582 (EA).

***Acalyphavolkensii* Pax** – Habit: Subshrub. Habitat: Forest undergrowth, edges and associated bushland, montane woodland, 650–1750 m. Vouchers: Watuma BM W0226 (EA, HIB), Wakanene KM & Mwangangi OM 306 & 562, Faden RB et al. NMK Exped. 30, Medley KE 994, Mbale M et al. NMK 960 (EA).

***Argomuelleramacrophylla* Pax** – Habit: Subshrub. Habitat: Forest undergrowth, 1425–1850 m. Vouchers: Beentje HJ et al. NMK Exped. 1043, Gardner 2948, Raler I 0091 (EA).

***Crotondichogamus* Pax** – Habit: Shrub/Tree. Habitat: Dry forest, bushland and thicket, 970–1150 m. Vouchers: Mwachala G et al. EW208, Mungai GM et al. EW2652, Greenway PJ & Kanuri K 12825, Morawetz JJ 406, Hucks M 915 (EA).

***Crotonmacrostachyus* Hochst. ex Delile** – Habit: Tree. Habitat: Secondary forests and forest edges, 1250–1550 m. Vouchers: Mwachala G et al. 1257, Beentje HJ et al. NMK Exped. 852 (EA).

***Crotonmegalocarpus* Hutch.** – Habit: Tree. Habitat: Evergreen forest, 1644–2000 m. Vouchers: Watuma BM W0261 (EA, HIB), Wakanene KM & Mwangangi OM 77, Faden RB et al. NMK Exped. 527, Raler I 0062 (EA).

***Crotonmenyharthii* Pax** – Habit: Shrub. Habitat: Deciduous bushland and thicket, 550–650 m. Voucher: Medley KE 544b (EA).

***Crotonpseudopulchellus* Pax** – Habit: Shrub. Habitat: Dry evergreen forest, deciduous woodland, bushland and thicket, 550–1110 m. Vouchers: SAJIT–005414 (EA, HIB), Mwachala G et al. EW1289, Mungai GM et al. EW1372, Faden RB & Faden AJ 74/483, Medley KE 545, 722 & 754, Joanna B 8835 (EA).

***Crotonsylvaticus* Hochst.** – Habit: Tree. Habitat: Forest edges and secondary forests, 1700–1875 m. Vouchers: Watuma BM W0284, Faden RB et al. NMK Exped. 546 (EA).

***Erythrococcabongensis* Pax** – Habit: Shrub. Habitat: Forest edges and associated bushland along rivers, 650–1000 m. Voucher: Medley KE 694 (EA).

***Erythrococcakirkii* (Müll.Arg.) Prain** – Habit: Shrub. Habitat: Bushland and thicket along streams, ca. 1050 m. Vouchers: Watuma BM W0152 (EA, HIB), Bally PRO 12694 (EA).

***Euphorbiabicompacta* Bruyns** – Habit: Tree. Habitat: Open deciduous bushland and woodland on rocky slopes, 800–1826 m. Vouchers: Watuma BM W0104 (EA, HIB), Luke WRQ et al. 5344, Medley KE 965, Beentje HJ et al. NMK Exped. 743, Napier ER 1322 (EA).

**EuphorbiabreviarticulataPaxvar.breviarticulata** – Habit: Shrub. Habitat: Dry open *Acacia*-*Commiphora* bushland, 465–600 m. Vouchers: Kirika P, Kamau P & Ndiege C GBK 44, Leach LC & Bayliss R 10241 (EA).

***Euphorbiaburuana* Pax** – Habit: Herb. Habitat: Open *Acacia*-*Commiphora* bushland, 500–1150 m. Vouchers: Engler 1930a (EA), Bally C 14138 (EA), Bally PRO 13407 (EA), Morawets JJ & Wabuyele E 405 (EA), Leach LC 10301 (EA).

**EuphorbiabusseiPaxvar.bussei** – Habit: Tree. Habitat: Open deciduous bushland, rocky slopes, sandy soils, ca. 953 m. Voucher: Mwachala G, Nyaboke, Saidi S 1033 (EA). Endangered.

**EuphorbiabusseiPaxvar.kibwezensis (N.E.Br.) S.Carter** – Habit: Tree. Habitat: Deciduous woodland, steep rocky slopes, 570–1067 m. Vouchers: Mwachala G et al. EW501, Morawets JJ & Wabuyele E 400, Dyson WG 537, Leach LC et al. 10299, Bally PRO 14158, 14210, 14211 & 14214, Baslev H 50, Robertson SA 6774, Polhill RM & Paulo S 472 (EA).

^**E**^***Euphorbiaclassenii* P.R.O.Bally & S.Carter** – Habit: Shrub. Habitat: Exposed rock faces with deciduous woodland, 700–800 m. Vouchers: Luke WRQ & Luke PA 4169, Classen SA 70 (EA).

**EuphorbiacrotonoidesBoiss.subsp.crotonoides** – Habit: Herb. Habitat: Open woodland, scattered bushland, ca. 874 m. Voucher: Watuma BM W0178 (EA, HIB).

***Euphorbiacryptospinosa* P.R.O.Bally** – Habit: Shrub. Habitat: Open dry deciduous bushland, 426–600 m. Vouchers: Gillett JB 21004, Hucks M 604, Greenway PJ & Kanuri K 12665 & 13021 (EA).

**EuphorbiacuneataVahlsubsp.spinescens (Pax) S.Carter** – Habit: Shrub. Habitat: Dry sandy soils in open *Acacia*-*Commiphora* bushland, 460–750 m. Vouchers: Geldermalsen-de Jongh, E van 131, Hucks M 842, Bally PRO 13444, Morawetz JJ & Wabuyele E 408 (EA).

**EuphorbiacuneataVahlvar.pumilans S.Carter** – Habit: Shrub. Habitat: Grasslands, open *Acacia*-*Commiphora* bushland, 950–1400 m. Vouchers: Gillett JB 19589, Bally PRO 13412 & 13812, Gilbert MG 5818 (EA).

***Euphorbiaengleri* Pax** – Habit: Shrub. Habitat: Forest undergrowth and dense bushland, 1425–2164 m. Vouchers: Watuma BM W0089 (EA, HIB), Kabuye CHS 82/75, Mwachala G & Nyaboke 1041, Wakanene KM & Mwangangi OM 41, Christenhusz MJM et al. 6627, Faden RB et al. NMK Exped. 206, 380 & 557, Faden RB & Faden AJ 71/1027 & 77/ 338, Beentje HJ 2129, Drummond RB & Hemsley JH 4359, Gillett JB et al. 17105, Heller E s.n. (EA).

**^NE^*Euphorbiafurcata* N.E.Br.** – Habit: Herb. Habitat: *Acacia* bushland in dry rocky sandy soils, ca. 700 m. Voucher: Luke WRQ & Luke PA 4180 (EA). Vulnerable.

***Euphorbiaguentheri* (Pax) Bruyns** – Habit: Herb. Habitat: Amongst grass in open scrubland, 915–1000 m. Vouchers: Bally PRO E92 & 13405, Uhlig C 48 (EA).

**EuphorbiaheterochromaPaxsubsp.heterochroma** – Habit: Shrub. Habitat: *Acacia*-*Commiphora* bushland, 600–900 m. Voucher: Lubai IK HB23 (EA).

**EuphorbiaheterochromaPaxsubsp.tsavoensis S.Carter** – Habit: Shrub. Habitat: Deciduous bushland, rocky outcrops, 650–1000 m. Vouchers: Morawetz JJ & Wabuyele E 402, Bally PRO 13351, Hucks M 978, Medley KEM p.r. (EA).

***Euphorbiaingens* E.Mey. ex Boiss.** – Habit: Tree. Habitat: Open wooded grasslands, rocky slopes, 550–1000 m. Voucher: Medley KE p.r. (EA).

^**NE**^***Euphorbianeoglaucescens* Bruyns** – Habit: Shrub. Habitat: Dry deciduous woodland, rocky slopes, 800–950 m. Voucher: Kabuye CHS et al. NMK Exped. 743 (EA).

**EuphorbianyikaePax ex Engl.var.neovolkensii (Pax) S.Carter** – Habit: Tree. Habitat: Open deciduous woodland, *Commiphora* scrub, 570–900 m. Vouchers: Polhill RM & Kibuwa SP 472, Balslev H 50, Dyson WG 537 (EA).

^**NE**^***Euphorbiapetricola* P.R.O.Bally & S.Carter** – Habit: Herb. Habitat: Dry *Acacia*-*Commiphora* forest on rocky hillside, ca. 1050 m. Voucher: Luke WRQ & Luke PA 6448 (EA). Endangered.

***Euphorbiapolyacantha* Boiss.** – Habit: Herb. Habitat: Shrub. Habitat: *Commiphora*-*Sansevieria* bushland thicket, ca. 520 m. Voucher: Faden RB & Faden AJ 74/477 (EA).

***Euphorbiapolyantha* Pax** – Habit: Subshrub. Habitat: Dry deciduous bushland on sandy stony soils, ca. 540 m. Voucher: Gillett JB 17211 (EA).

***Euphorbiapseudomollis* Bruyns** – Habit: Tree. Habitat: Open deciduous woodland on slopes, succulent thicket, 700–900 m. Vouchers: Luke WRQ & Luke PA 6432, Bally PRO 12725, Leach LC & Bayliss R 10258 (EA).

***Euphorbiaquinquecostata* Volkens** – Habit: Tree. Habitat: Mixed deciduous woodland on rocky hillsides, 650–1000 m. Vouchers: Verdcourt B 3891, Bally PRO 8658, Dyson WG 536 (EA).

***Euphorbiarobecchii* Pax** – Habit: Tree. Habitat: Open *Acacia*-*Commiphora* bushland, 457–650 m. Vouchers: Morawetz JJ & Wabuyele E 409, Greenway PJ & Kanuri K 12831, Bally PRO E15, Leach LC & Bayliss B 10225, Medley KE p.r. (EA).

***Euphorbiascheffleri* Pax** – Habit: Subshrub. Habitat: Open *Acacia*-*Commiphora* bushland on sandy stony soils, 640–1000 m. Vouchers: Medley KE 521, Polhill RM & Kibuwa SP 933, Drummond RB & Hemsley JH 4415, Gillett JB 17207 (EA).

**EuphorbiaschimperianaScheelevar.pubescens (N.E.Br.) S.Carter** – Habit: Shrub. Habitat: Forest clearings and edges, in grass, ca. 1000 m. Voucher: Mwachala G et al. EW2564 (EA).

*Euphorbiaserpens Kunth – Habit: Shrub. Habitat: Roadsides, ca. 560 m. Voucher: Bally PRO 13250 (EA).

***Euphorbiasystyloides* Pax** – Habit: Herb. Habitat: Open woodland, disturbed sites in grass, ca. 970 m. Voucher: Mwachala G et al. EW20 (EA).

**EuphorbiatenuispinosaGillivar.robusta P.R.O.Bally & S.Carter** – Habit: Herb. Habitat: Dry deciduous bushland in rock sandy soils, 550–800 m. Voucher: Luke WRQ et al. 4256, Medley KEM p.r. (EA).

**EuphorbiatenuispinosaGillivar.tenuispinosa** – Habit: Herb. Habitat: *Acacia*-*Commiphora* bushland, open evergreen forest, 609–1100 m. Vouchers: Napier ER 988, Bally PRO 13415 & 14139, Gilbert MG 5816, Tweedie 86 (EA). Vulnerable.

***Euphorbiatirucalli* L.** – Habit: Shrub/Tree. Habitat: Grassland, thin woodland, homesteads, 998–1219 m. Vouchers: Watuma BM W0318 (EA, HIB), Bally PRO & Carter S 14136, Napier ER 1147, Medley KEM p.r. (EA).

***Euphorbiatransvaalensis* Schltr.**– Habit: Herb. Habitat: Dense bushland, light woodland near stream beds, 450–500 m.Voucher: Greenway PJ & Kanuri K 12678 (EA).

***Euphorbiaumbellata* (Pax) Bruyns** – Habit: Tree. Habitat: Rocky slopes with dry open woodland, 1700–1875 m. Voucher: Faden RB et al. NMK Exped. 340 (EA).

**EuphorbiausambaricaPaxsubsp.usambarica** – Habit: Shrub. Habitat: Understorey in open montane forest, 950–1850 m. Vouchers: Morawetz JJ & Wabuyele E 401, Gillett JB, Burtt BL & Osborn RM 17105, Faden RB et al. NMK Exped. 1000, Beentje HJ et al. NMK Exped. 1072, Bally PRO 8776 & 13599 (EA).

*Jatrophacurcas L. – Habit: Shrub/Tree. Habitat: Hedge plant escaping into the wild. Voucher: Otieno GO 77A (EA).

***Jatrophaspicata* Pax** – Habit: Subshrub. Habitat: Deciduous bushland and thicket, shallow soil on rocky hillsides, 487–1000 m. Vouchers: Luke WRQ & Luke PA 4175, Faden RB & Faden AJ 74/434, Medley KE 479, 508 & 805, Hildebrandt JM 2428, Napier ER 920, Bally PRO 8674, Verdcourt B & Polhill RM 2710, Greenway PJ & Kanuri K 12657 & 12719, Verdcourt B 5320, Mrs. Robertson SA 7503 (EA).

^**NE**^***Jatrophavelutina* Pax & K.Hoffm.** – Habit: Subshrub. Habitat: *Acacia*-*Commiphora* bushland and mixed thicket, 550–700 m. Vouchers: Luke WRQ & Luke PA 4171, Medley KE 504 (EA).

***Macarangacapensis* (Baill.) Sim** – Habit: Tree. Habitat: Evergreen forest, 1200–1850 m. Vouchers: Raler I 0015, Faden RB et al. 70/515, NMK Taita Hills Exped. 1183 (EA).

**^NE^*Macarangaconglomerata* Brenan** – Habit: Tree. Habitat: Upland evergreen forest, 1350–1875 m. Vouchers: Watuma BM W0062 (EA, HIB), Wakanene KM & Mwangangi OM 213 & 623, Drummond RB & Hemsley JH 4372, Faden RB et al. NMK Exped. 202 & 462, Beentje HJ et al. NMK Exped. 1097, Faden RB et al. 71/18, 247, 72/200 & 77/318, Raler 0147 (EA). Vulnerable.

***Macarangakilimandscharica* Pax** – Habit: Tree. Habitat: Evergreen forest, secondary forest, 1000–1800 m. Vouchers: Mungai GM et al. EW3557, Bally PRO 13601 (EA).

**Mildbraediacarpinifolia(Pax)Hutchvar.carpinifolia** – Habit: Shrub. Habitat: Forest undergrowth and edges, riverine, 800–950 m. Voucher: Kabuye CHS et al. NMK Exped. 617 (EA). Vulnerable.

***Neoboutoniamacrocalyx* Pax** – Habit: Tree. Habitat: Forest edges, openings and regrowth, 1700–2000 m. Vouchers: Raler I 0136, Faden RB et al. NMK Exped. 346 & 500, Gillett JB 17244, Heller E s.n. (EA).

*Ricinuscommunis L. – Habit: Herb. Habitat: Evergreen bushland, roadsides, near homesteads, 1040–1778 m. Vouchers: Watuma BM W0286 (EA, HIB), Mwachala G et al. EW1219 & 1360 (EA).

***Spirostachysafricana* Sond.** – Habit: Shrub/Tree. Habitat: Deciduous woodland and bushland, 650–1000 m. Voucher: Medley KE 914 (EA).

***Suregadazanzibariensis* Baill.** – Habit: Tree. Habitat: Forest, woodland and bushland, 650–1150 m. Vouchers: Mungai GM et al. EW1654, Medley KE 536, 538, 542, 598, 676 & 712, Faden RB et al. 69/447 (EA).

***Tragiabrevipes* Pax** – Habit: Climber. Habitat: Forest edges and thickets, riverine, disturbed sites, 1048–1250 m. Vouchers: Watuma BM W0161 (EA, HIB), Mwachala G et al. EW2576 (EA).

***Tragiaimpedita* Prain** – Habit: Subshrub. Habitat: Roadsides, rocky or disturbed deciduous bushland and wooded grassland, ca. 600 m. Voucher: Faden RB & Faden AJ 74/453 (EA).

**F82. FABACEAE**

55 Genera, 125 species

**AbrusprecatoriusL.subsp.africanus Verdc.** – Habit: Liana. Habitat: Woodland, bushland, grassland with scattered thicket, 650–1000 m. Vouchers: SAJIT–004528 (EA, HIB), Kabuye CHS et al. NMK Exped. 614, Medley KE 584 (EA).

***Abrusfruticulosus* Wall. ex Wight & Arn.** – Habit: Shrub. Habitat: Acacia woodland, bushland on rocky slopes, forest edge, 700–950 m. Vouchers: Kabuye CHS et al. NMK Exped. 728, Mwachala G et al. EW780, Polhill RM & Kibuwa SP 957, Bally J 12705 (EA).

*Acaciamearnsii De Wild. – Habit: Tree. Habitat: Forest and forest remnant margins, 1830–2170 m. Vouchers: Watuma BM W0094 (EA, HIB), Faden RB et al. NMK Exped. 90 (EA).

*Acaciamelanoxylon R.Br. – Habit: Tree. Habitat: Remnant forest patches, 2000–2170 m. Voucher: Faden RB et al. NMK Exped. 91 (EA).

***Adenocarpusmannii* (Hook.f.) Hook.f.** – Habit: Shrub. Habitat: Margins of upland rainforest, ca. 1814 m. Voucher: SAJIT–004549 (EA, HIB).

***Aeschynomeneschimperi* Hochst. ex A.Rich.** – Habit: Subshrub. Habitat: Riverine vegetation, 800–950 m. Vouchers: Kabuye CHS et al. NMK Exped. 698, Mwachala G et al. EW1683 (EA).

***Afroamphicaafricana* (Hook.f.) H.Ohashi & K.Ohashi** – Habit: Climber. Habitat: Evergreen forest in open areas and path sides, 1100–1980 m. Vouchers: SAJIT–004571 & 006363 (EA, HIB), Mungai GM et al. EW1307, Drummond RB & Hemsley JH 4288 (EA).

**Albiziaadianthifolia(Schumach.)W.Wightvar.intermedia (De Wild. & T.Durand) Villiers** – Habit: Tree. Habitat: Lowland rainforest, deciduous woodland, ca. 900 m. Voucher: Mwachala G et al. EW1689 (EA).

***Albiziaamara* (Roxb.) Boivin** – Habit: Tree. Habitat: Edge of deciduous bushland near stream, bushed grassland, ca. 1059 – 1070 m. Vouchers: Watuma BM W0150 (EA, HIB), Mwachala G et al. EW1367 (EA).

***Albiziaanthelmintica* (A.Rich.) Brongn.** – Habit: Tree. Habitat: Deciduous bushland, 550–650 m. Voucher: Medley KE 943 (EA).

***Albiziagummifera* (J.F.Gmel.) C.A.Sm.** – Habit: Tree. Habitat: Evergreen forest, 1220–1875 m. Vouchers: Watuma BM W0291 (EA, HIB), Faden RB et al. NMK Exped. 328, Joanna 8945 & 8961, Faden RB et al. 71/1003, Medley KE 919 (EA).

***Albiziaharveyi* E.Fourn.** – Habit: Tree. Habitat: Bushland, 500–650 m. Vouchers: Medley KE 1004, Medley KE s.n. (EA).

***Albiziapetersiana* (Bolle) Oliv.** – Habit: Tree. Habitat: Ground water forests, bushland, ravines in deciduous woodland. Voucher: Joanna B 8796 (EA).

***Albiziazimmermannii* Harms** – Habit: Tree. Habitat: Secondary bush near lowland rainforest, 650–1000 m. Vouchers: Medley 689 & 716, Joanna B 8905 (EA).

***Antopetitiaabyssinica* A.Rich.** – Habit: Herb. Habitat: Forest clearings, roadsides, weed in cultivations, 1817–2170 m. Vouchers: SAJIT–004550 & 006401 (EA, HIB), Faden RB et al. NMK Exped. 110 (EA).

***Bauhiniataitensis* Taub.** – Habit: Shrub. Habitat: Deciduous bushland, dry scrub with trees, 457–640 m. Vouchers: Mwangangi 7, Faden RB & Faden AJ 74/444, Greenway PJ & Kanuri K 12728, Bally PRO 8852, Hildebrandt JM 2603, Ossent JR 148 (EA).

***Bauhiniatomentosa* L.** – Habit: Shrub. Habitat: Woodland, deciduous bushland and thicket along river, 800–1061 m. Vouchers: SAJIT–005358, Watuma BM W0163 (EA, HIB), Faden RB & Faden AJ 74/468, Medley KE 486 & 818, Drummond RB & Hemsley JH 4407, Sacleux C 2366 (EA).

* ***Biancaeadecapetala* (Roth) O.Deg.** – Habit: Shrub. Habitat: Mist forest margin next to cultivated lands, pathside, ca. 1851 m. Voucher: Watuma BM W0091 (EA, HIB).

*Calliandrahoustoniana (Mill.) Standl. – Habit: Shrub. Habitat: Margins of relict montane forest, cultivated lands, ca. 1348 m. Voucher: Watuma BM W0228 (EA, HIB).

***Cassiaabbreviata* Oliv.** – Habit: Tree. Habitat: Deciduous bushland and dry scrub with trees, 500–1000 m. Vouchers: Faden RB et al. 69/417, Medley KE 1020, Friis I & Hansen OJ 2663, Vesey-FitzGerald LDEF 41, Hucks M 431, Bally PRO 13283, Tim C & Oakley s.n. (EA).

***Chamaecristakirkii* (Oliv.) Standl.** – Habit: Herb. Habitat: Forest edges and clearings, grassland, ca. 1500 m. Voucher: SAJIT–004116 (EA, HIB).

***Chamaecristamimosoides* (L.) Greene** – Habit: Herb. Habitat: Forest clearings and margins, wooded grassland, cultivated and waste grounds, ca. 1220 m. Voucher: Napier ER 1073 in CM 2242 (EA).

***Chamaecristazambesica* (Oliv.) Lock** – Habit: Subshrub. Habitat: Wooded grassland, grassland, 914–1600 m. Vouchers: Beentje HJ et al. NMK Exped. 1156, Hildebrandt JM 2464 (EA).

***Craibiabrevicaudata* (Vatke) Dunn** – Habit: Tree. Habitat: Evergreen forest, semi-deciduous woodland, dry forest on rocky slopes, riverine, 487–1640 m. Vouchers: Luke WRQ & Luke PA 5526, Faden RB, Evans A & Msafiri F 70/958, Verdcourt B & Polhill RM 2715, Greenway PJ & Kanuri K 13027, Medley KE 556, 581, 719 & 995 (EA). Near Threatened.

***Craibiazimmermannii* (Harms) Dunn** – Habit: Tree. Habitat: Forest, 1425–1875 m. Vouchers: Watuma BM W0270 (EA, HIB), Beentje HJ et al. NMK Exped. 877, Faden RB et al. NMK Exped. 384 & 537, Faden RB et al. 70/529, 748 & 71/970, Raler I 0013 (EA).

***Crotalariaaxillaris* Aiton** – Habit: Subshrub. Habitat: Forest margins, deciduous woodland and bushland, abandoned cultivations, 1250–1875 m. Vouchers: SAJIT–005342 (EA, HIB), Mwachala G et al. EW3526, Wakanene KM & Mwangangi OM 347 & 436, Faden RB et al. NMK Exped. 188 (EA).

**CrotalariabarkaeSchweinf.subsp.barkae** – Habit: Herb. Habitat: Deciduous bushland and grassland, ca. 900 m. Voucher: Mwachala G et al. EW2810 (EA).

**CrotalariabarkaeSchweinf.subsp.cordisepala Polhill** – Habit: Herb. Habitat: Roadside in bracken zone, sandy places in grassland and stream sides, 900–1360 m. Vouchers: Kabuye CHS 82/146, Faden RB & Faden AJ 72/257, Flauret A 13 (EA).

**CrotalariabarkaeSchweinf.subsp.teitensis (Sacleux) Polhill** – Habit: Herb. Habitat: Deciduous woodland and bushland, grassland, 530–950 m. Vouchers: Kabuye CHS et al. NMK Exped. 653, Muasya S 45, Sacleux C 2406, Soest LU EA16062 (EA).

***Crotalariadistantiflora* Baker f.** – Habit: Herb. Habitat: Upland rainforest clearings and margins, 1371–1780 m. Vouchers: Wakanene KM & Mwangangi OM 613a & 740, Napier ER 1100 (EA).

***Crotalariagoodiiformis* Vatke** – Habit: Shrub. Habitat: Margins and clearings of upland rainforest, dry evergreen forest, deciduous woodland, 720–950 m. Vouchers: Kabuye CHS et al. NMK Exped. 735, Gillett JB 18744, Hildebrandt JM 2548 (EA).

***Crotalariagreenwayi* Baker f.** – Habit: Herb. Habitat: Grassland, deciduous bushland, persisting on cultivated ground, 620–853 m. Vouchers: Greenway PJ & Kanuri K 10428, Gillett JB & Burtt BL 17175 (EA).

**CrotalariaincanaL.subsp.incana** – Habit: Subshrub. Habitat: 950–1580 m. Vouchers: SAJIT–004109 (EA, HIB), Mwachala G et al. EW2806 & 3066A (EA).

***Crotalarialaburnifolia* L.** – Habit: Herb. Habitat: Deciduous woodland, bushland, secondary scrub, grassland, roadsides, 700–1773 m. Vouchers: SAJIT–005369, Watuma BM W0263 (EA, HIB), Kirika P, Rory M, Mbale M NMK 594, Luke WRQ et al. 4230, Gillett JB 17219 (EA).

**^NE^*Crotalarialukwangulensis* Harms** – Habit: Shrub/Liana. Habitat: Rainforest margins and clearings, 1425–2195 m. Vouchers: Wakanene KM & Mwangangi OM 375, Mwasaru G 24, Faden RB et al. NMK Exped. 129 & 950, Faden RB et al. 71/1005, Lavranos JJ & Newton 17642, Verdcourt B & Polhill RM 2716, Drummond RB & Hemsley JH 4358, Gillett JB, Burtt BL & Osborn RM 17088 (EA).

**CrotalarianatalitiaMeisn.var.rutshuruensis De Wild.** – Habit: Subshrub. Habitat: Margins of upland rainforest and grassland, deciduous bushland and woodland, 1350–2000 m. Vouchers: Mwachala G et al. EW2665, Beentje HJ et al. NMK Exped. 883 & 927, Drummond RB & Hemsley JH 4300 (EA).

***Crotalariapolysperma* Kotschy** – Habit: Herb. Habitat: Deciduous woodland and bushland, *Acacia*-*Commiphora* scrub, 600–701 m. Vouchers: Polhill RM & Kibuwa SP 967, Mrs. Robertson SA 6542 (EA).

***Crotalariascassellatii* Chiov.** – Habit: Shrub. Habitat: Forest margins, deciduous bushland and grassland, rocky outcrops, 610–950 m. Vouchers: Kirika P, Rory M & Mbale M NMK 593, Polhill RM & Kibuwa SP 936 (EA).

***Crotalariaukambensis* Vatke** – Habit: Herb. Habitat: Deciduous bushland and scattered tree grassland, 609–1100 m. Vouchers: Cheseny CMC 32/72, Polhill RM & Kibuwa SP 932, Gillett JB 17232, Napier ER 930, Rauh W KE71 (EA). Endangered.

***Crotalariavallicola* Baker f.** – Habit: Herb. Habitat: Grassland and secondary bushland, rock outcrops, cleared and cultivated lands, 1371–1480 m. Vouchers: Napier ER 1129 (EA), Hemp A 4566 (EA).

**^NE^*Cynometra* sp. A of FTEA** – Habit: Tree. Habitat: Moist forest, 1425–1850 m. Vouchers: Faden RB et al. 70/564, Faden RB et al. NMK Exped. 783, Raler I 0014 (EA).

***Dalbergialactea* Vatke** – Habit: Shrub. Habitat: Upland forest, forest margins and evergreen bushland, 1425–1851 m. Vouchers: SAJIT–006341, Watuma BM W0087 (EA, HIB), Beentje HJ et al. NMK Exped. 425, Hildebrandt JM 2439, Gardner HM 2945 (EA).

***Dalbergiamelanoxylon* Guill. & Perr.** – Habit: Shrub/Tree. Habitat: Deciduous woodland, rocky slopes and valleys, 650–1400 m. Vouchers: Kabuye CHS 82/95, Kabuye CHS et al. NMK Exped. 680, Mwachala G et al. EW104, 964, 1237, 1326, Wakanene KM & Mwangangi OM 457, Medley KE 865 & 916 (EA). Near Threatened.

***Dalbergiamicrophylla* Chiov.** – Habit: Shrub/Tree. Habitat: Deciduous woodland, 710–960 m. Vouchers: Medley KE 559 & 860, Gillett JB 19564, Joanna B 8834 (EA).

***Dalbergiavacciniifolia* Vatke** – Habit: Shrub/Tree. Habitat: Deciduous bushland and woodland, 600–850 m. Voucher: Medley KE 858b (EA).

***Delonixelata* (L.) Gamble** – Habit: Tree. Habitat: Deciduous thickets and bushland, 550–650 m. Vouchers: Medley KE 415, Napier ER Bax TNP/E/36 (EA).

**Dichrostachyscinerea(L.)Wight & Arn.subsp.cinerea** – Habit: Shrub. Habitat: Deciduous bushland and woodland, grassy slopes near forest, 944–1150 m. Vouchers: Watuma BM W0004 (EA, HIB), Mungai GM et al. EW1392 & 1668 (EA).

**Dichrostachyscinerea(L.)Wight & Arn.subsp.forbesii (Benth.) Brenan & Brummitt** – Habit: Shrub. Habitat: Evergreen bushland, clump grassland, 550–1250 m. Vouchers: Wakanene KM & Mwangangi OM 439, Medley KE 464 (EA).

**DolichossericeusE.Mey.subsp.glabrescens Verdc.** – Habit: Climber. Habitat: Bushland and dry evergreen forest, 1219–1400 m. Vouchers: Mwachala G et al. EW3108A, Napier ER 1085 & 1088, Animal Husbandry Division EA 14194 (EA).

***Entadaleptostachya* Harms** – Habit: Liana. Habitat: Deciduous bushland, dry scrub with trees, 550–884 m. Vouchers: Watuma BM W0177 (EA, HIB), Medley KE 519, Faden RB & Faden AJ 74/267, Braun K 1540 (EA).

***Eriosemamontanum* Baker f.** – Habit: Herb. Habitat: Forest edges, bushland, grassland, 914–2000 m. Vouchers: Luke WRQ et al. 4221, Beentje HJ et al. NMK Exped. 907, Hildebrandt JM 2445 (EA).

***Eriosemapsoraleoides* (Lam.) G.Don** – Habit: Shrub. Habitat: Grassland with scattered *Acacia*-*Commiphora*, bushland, cultivation margins, 1050–1259 m. Vouchers: Kirika P et al. 01/2006/09, Mungai GM et al. EW1754, Bally PRO 8576, Napier ER 1075 (EA).

***Erythrinaabyssinica* Lam. ex DC.** – Habit: Tree. Habitat: Scattered tree grassland, open woodland, 600–1563 m. Vouchers: Watuma BM W0280 (EA, HIB), Drummond RB & Hemsley JH 4403, Medley KEM p.r. (EA).

***Erythrinaburttii* Baker f.** – Habit: Tree. Habitat: *Acacia*-*Commiphora*-*Combretum* woodland, derived grassland, ca. 960 m. Voucher: Gillett JB 19561 (EA).

***Erythrinamelanacantha* Taub. ex Harms** – Habit: Tree. Habitat: *Acacia*-*Commiphora* bushland, ca. 420 m. Voucher: Faden RB & Faden AJ 74/286 (EA).

***Faidherbiaalbida* (Delile) A.Chev.** – Habit: Tree. Habitat: Riverine, ground water forest and woodland. Voucher: Grenfell (EA).

***Gelrebiatrothaei* (Harms) Gagnon & G.P.Lewis**– Habit: Shrub. Habitat: Deciduous bushland, dry scrub with trees, ca. 487 m. Voucher: Bally PRO 8681 (EA).

***Gronabarbata* (L.) H.Ohashi & K.Ohashi** – Habit: Herb. Habitat: Deciduous woodland and grassland, 1425–1850 m. Voucher: Faden RB et al. NMK Exped. 445 (EA).

***Hylodesmumrepandum* (Vahl) H.Ohashi & R.R.Mill** – Habit: Herb. Habitat: Mist forest in clearings and margins, 920–2170 m. Vouchers: Watuma BM W0038 (EA, HIB), Mwachala G et al. EW1691, 2594 & 3064A, Napier ER 1112 (EA).

**Indigastrumcostatum(Guill. & Perr.)Schriresubsp.goniodes (Hochst. ex Baker) Schrire** – Habit: Subshrub. Habitat: *Terminalia*-*Sansevieria* bushland, grassland, ca. 600 m. Voucher: Faden RB & Faden AJ 74/244 (EA).

***Indigoferaarrecta* Hochst. ex A.Rich.** – Habit: Subshrub. Habitat: Forest margins, secondary growth, deciduous and upland evergreen bushland, 2000–2170 m. Voucher: Faden RB et al. NMK Exped. 123 (EA).

***Indigoferaatriceps* Hook.f.** – Habit: Herb. Habitat: Upland grassland, forest margins, along rivers, 900–2000 m. Vouchers: Mwachala G et al. EW1694, Drummond RB & Hemsley JH 4298 (EA).

***Indigoferakaessneri* Baker f.** – Habit: Subshrub. Habitat: Grassland, bushland and forest margins, 1250–2170 m. Vouchers: Faden RB et al. NMK Exped. 124, Napier ER 1087 (EA).

***Indigoferalupatana* Baker f.** – Habit: Shrub. Habitat: Deciduous bushland and thicket on rocky slopes, 650–1000 m. Vouchers: Watuma BM W0170 (EA, HIB), Kabuye CHS et al. NMK Exped. 642, Mrs. Robertson SA 3587, Medley KE 453, 756 & 810 (EA).

***Indigoferasisalis* J.B.Gillett** – Habit: Herb. Habitat: Deciduous woodland and bushland, sisal plantations, cultivated lands, 600–762 m. Vouchers: Sampson HC 97 (EA), Hucks M 686 (EA).

***Indigoferaspicata* Forssk.** – Habit: Herb. Habitat: Deciduous bushland, cultivated areas and waste places, ca. 550 m. Voucher: Medley KE 517 (EA).

**IndigoferasubulataVahl ex Poir.var.scabra (Roth) Meikle** – Habit: Subshrub. Habitat: Bushland, secondary growth, 550–650 m. Vouchers: Medley KEM s.n., Grenfell (EA).

**IndigoferaswaziensisBolusvar.perplexa (N.E.Br.) J.B.Gillett** – Habit: Shrub. Habitat: Upland evergreen bushland and forest margins, 900–1200 m. Voucher: Gillett JB 18751 (EA).

**^NE^*Indigoferatanganyikensis* Baker f.** – Habit: Subshrub. Habitat: *Acacia*-*Commiphora* open bushland, 600–1000 m. Vouchers: Faden RB & Faden AJ 74/451, Uhlig C 33 (EA).

*Indigoferatinctoria L. – Habit: Shrub. Habitat: Bush margins, cultivations and secondary growth, ca. 1130 m. Voucher: Medley KE 649 (EA).

***Kotschyaaeschynomenoides* (Welw. ex Baker) Dewit & P.A.Duvign.** – Habit: Shrub. Habitat: Upland forest, evergreen bushland, rocky outcrops on hills, 1371–1800 m. Vouchers: Kabuye CHS et al. 82/55, Dale in FD 3782, Bally PRO 13639, Drummond RB & Hemsley JH 4328 (EA).

**KotschyaafricanaEndl.var.bequaertii (De Wild.) Verdc.** – Habit: Shrub. Habitat: Forest edges, fringing forest, thicket, scrub, 1500–1550 m. Voucher: Beentje HJ et al. NMK Exped. 885 (EA).

*Leucaenaleucocephala (Lam.) de Wit – Habit: Tree. Habitat: Forest edges, roadside, settlements, ca. 1567 m. Voucher: Watuma W0208 (EA, HIB).

**Macrotylomaaxillare(E.Mey.)Verdc.var.axillare** – Habit: Climber. Habitat: Grassland with scattered trees, thicket, 1000–1550 m. Vouchers: Mwachala G et al EW3056A (EA), Mungai G et al EW1339 (EA).

**Macrotylomaaxillare(E.Mey.)Verdc.var.glabrum (E.Mey.) Verdc.** – Habit: Climber. Habitat: Grassland, bushland, open forest, ca. 2050 m. Voucher: Gillett JB 17250 (EA).

***Macrotylomamaranguense* (Taub.) Verdc.** – Habit: Climber. Habitat: Grassland with scattered trees, 1220–1371 m. Vouchers: Napier ER 1090, Bally PRO 12736 (EA).

**Macrotylomauniflorum(Lam.)Verdc.var.uniflorum** – Habit: Climber. Habitat: Old cultivations, ca. 1240 m. Voucher: Mungai GM et al. EW3194 (EA).

**Macrotylomauniflorum(Lam.)Verdc.var.stenocarpum (Brenan) Verdc.** – Habit: Climber. Habitat: *Acacia* bushland and thicket, old cultivations, ca. 450–610 m. Vouchers: Napier ER 1011, Gillett JB 21009 (EA).

**^NE^MillettiaoblataDunnsubsp.burttii J.B.Gillett** – Habit: Tree. Habitat: Deciduous thicket, ca. 890 m. Vouchers: Mwachala G et al. EW1680, Lubai lK HB17 (EA).

**^NE^MillettiaoblataDunnsubsp.teitensis J.B.Gillett** – Habit: Tree. Habitat: Upland evergreen forest and scrub, 1225–1875 m. Vouchers: SAJIT–003296 & 006386, Watuma BM W0072 (EA, HIB), Wakanene KM & Mwangangi OM 546, Maina 17, Bytebier B 1430, Faden RB et al. NMK Exped. 39, 276, 420 & 485, Beentje HJ et al. NMK Exped. 1184, Drummond RB & Hemsley JH 4390, Gillett JB, Burtt BL & Osborn RM 17118, Dale IR 3781, Bally PRO 8587, Raler I 0022, Gardner HM 1201, Heller E s.n. (EA). Vulnerable.

***Millettiausaramensis* Taub.**– Habit: Tree. Habitat: Wooded grassland, 800–950 m. Voucher: Kabuye CHS et al. NMK Exped. 630 (EA).

***Mucunagigantea* (Willd.) DC.** – Habit: Liana. Habitat: Bushland, forest edges along river, ca. 1056 m. Voucher: Watuma BM W0159 (EA, HIB).

***Munduleasericea* (Willd.) A.Chev.** – Habit: Shrub. Habitat: Deciduous woodland and bushland on rocky hills, 650–1000 m. Vouchers: Medley KEM 468, 745 & 858a (EA).

**Neonotoniawightii(Wight & Arn.)J.A.Lackeyvar.mearnsii (De Wild.) J.A.Lackey** – Habit: Climber. Habitat: Forest, grassland with scattered trees and secondary grassland, ca. 900 m. Voucher: Mwachala G et al. EW2809 (EA).

***Newtoniabuchananii* (Baker f.) G.C.C.Gilbert & Boutique** – Habit: Tree. Habitat: Upland rainforest near steams, ground water and riverine forests, 1200–1640 m. Vouchers: Beentje HJ et al. NMK Exped. 1078, Raler I 0005, Medley KE 940 (EA).

***Newtoniahildebrandtii* (Vatke) Torre** – Habit: Tree. Habitat: Riverine forests, dry areas with high water table, 600–800 m. Vouchers: Drummond RB & Hemsley JH 4275, Hildebrandt JM 2492, Medley KE 869 & 959, Bally PRO 8777 (EA).

***Ormocarpumkirkii* S.Moore** – Habit: Tree. Habitat: *Acacia*-*Commiphora* bushland, grassland, 750–980 m. Vouchers: Faden RB & Faden AJ 74/506, Medley KE 639 (EA).

***Philenopteraeriocalyx* (Harms) Schrire** – Habit: Shrub/Tree. Habitat: Dry evergreen forest, deciduous bushland, 550–650 m. Voucher: Medley KE 781 (EA).

***Platycelyphiumvoense* (Engl.) Wild** – Habit: Tree. Habitat: *Acacia*-*Commiphora* deciduous bushland, 500–670 m. Vouchers: Medley KE 969, Engler A 1958, Bally PRO 8851 (EA).

***Pleurolobussalicifolius* (Poir.) H.Ohashi & K.Ohashi** – Habit: Shrub. Habitat: Wet areas in grassland, near rivers, ca. 1000 m. Voucher: Mungai GM et al. EW2819 (EA).

**PseudarthriahookeriWight & Arn.var.hookeri** – Habit: Subshrub. Habitat: Grassland with scattered trees and thicket, old cultivations, 1030–1220 m. Vouchers: Mwachala G et al. EW795, Napier ER 1098 (EA).

***Pseudarthriahookeri* Wight & Arn. var. A** – Habit: Subshrub. Habitat: Deciduous woodland and bushland, ca. 1000 m. Voucher: Mwachala G et al. EW1569 (EA).

***Rhynchosiahirta* (Andrews) Meikle & Verdc**. – Habit: Climber. Habitat: Forest edges, hillside bushland, cultivation margins, ca. 1000 m. Voucher: Mungai GM et al EW3558 (EA).

***Rhynchosianyasica* Baker** – Habit: Herb. Habitat: Grassland with scattered trees, woodland, ca. 1200 m. Voucher: Napier ER 1094 (EA).

***Rhynchosiapulchra* (Vatke) Harms** – Habit: Shrub. Habitat: Bushland, open woodland, 600–1100 m. Vouchers: Luke WRQ & Luke PA 5507, Greenway PJ & Kanuri K 12585, Hildebrandt JM 2528 (EA).

**RhynchosiausambarensisTaub.var.usambarensis** – Habit: Herb. Habitat: Forest edges, bushland, riverine thicket, 1500–1750 m. Vouchers: SAJIT–004112 (EA, HIB), Mwachala G et al. EW3054 (EA).

***Senegaliabrevispica* (Harms) Seigler & Ebinger** – Habit: Tree. Habitat: Bushland, thickets and scrubs, 850–1200 m. Vouchers: Kabuye CHS et al. NMK Exped. 732, Mwachala G et al. EW222 & 1201, Wakanene KM & Mwangangi OM 481, Faden RB & Faden AJ 74/481, Medley KE 423 & 458, Joanna B 8867 (EA).

**Senegaliamellifera(Vahl)Seigler & Ebingersubsp.mellifera** – Habit: Tree. Habitat: Deciduous bushland, dry scrub with trees, 457–700 m. Vouchers: Faden RB & Faden AJ 74/273, Hucks M 871, King AL 14/82 & 13/83 (EA).

**Senegaliapolyacantha(Willd.)Seigler & Ebingersubsp.campylacantha (Hochst. ex. A.Rich.) Kyal. & Boatwr.** – Habit: Tree. Habitat: Grassland and riverbank, sandy soil, 600–800 m. Voucher: Jeffery GW K808 (EA).

***Senegaliasenegal* (L.) Britton** – Habit: Shrub. Habitat: Deciduous bushland, dry scrub with trees, 485–950 m. Vouchers: Faden RB & Faden AJ 74/269, Medley KE 425, Muasya AM & Safary 64 & 65, Hildebrandt JM 2486, Bally PRO 17124, Leippert H 5643, Friis I & Hansen OJ 2661 (EA).

*Sennabicapsularis (L.) Roxb. – Habit: Shrub. Habitat: Bushland, grassland, old cultivations, 600–1250 m. Vouchers: Mungai GM et al. EW1403, Bally PRO 8744 (EA).

***Sennadidymobotrya* (Fresen.) H.S.Irwin & Barneby** – Habit: Shrub. Habitat: Forest margins, 1700–1875 m. Vouchers: Watuma BM W0067 (EA, HIB), Wakanene KM & Mwangangi OM 718, Faden RB et al. NMK Exped. 475 (EA).

***Sennialongiracemosa* (Vatke) Lock** – Habit: Shrub. Habitat: Dry scrub with trees, deciduous bushland, 600–750 m. Vouchers: Hildebrandt JM 2506, Lubai IK HB 6 (EA).

*Sennaoccidentalis (L.) Link – Habit: Subshrub. Habitat: Riverine vegetation, weed of cultivation, roadside and waste ground, 600–950 m. Vouchers: Kabuye CHS et al. NMK Exped. 731, Napier ER 971 (EA).

***Sennasingueana* (Delile) Lock** – Habit: Shrub/Tree. Habitat: Woodland, wooded grassland, bushland, 1200–1371 m. Vouchers: Wakanene KM & Mwangangi OM 494, Napier ER 1123 (EA).

***Sesbaniasesban* (L.) Merr.** – Habit: Tree. Habitat: Evergreen bushland along streams, ca. 1206 m. Voucher: Watuma BM W0012 (EA, HIB).

***Spathionemakilimandscharicum* Taub.** – Habit: Climber. Habitat: Mixed deciduous bushland, 450–660 m. Vouchers: Faden RB & Faden AJ 74/277, Napier ER 900, Kuchar P et al. 5697, Ossent J 98, Gillett JB 16874 & 21003 (EA).

***Stylosanthesfruticosa* (Retz.) Alston** – Habit: Subshrub. Habitat: Bushland, grassland with scattered *Acacia*, old cultivations, 700–1100 m. Vouchers: SAJIT–005382 (EA, HIB), Mwachala G et al. EW94, Jongh EG 129 (EA).

***Tamarindusindica* L.** – Habit: Tree. Habitat: Remnant in cleared farmland, open woodland and bushland, ca. 859 m. Voucher: Watuma BM W0197 (EA, HIB).

***Tephrosiaaequilata* Baker** – Habit: Shrub. Habitat: Upland grassland, heathland and scrubland, 1676–2000 m. Vouchers: Faden RB et al. NMK Exped. 257, Dale IR 3779, Gillett JB, Burtt BL & Osborn RM 17139 (EA).

**Tephrosiavillosa(L.)Pers.subsp.ehrenbergiana (Schweinf.) Brummitt** – Habit: Herb. Habitat: Open places in *Acacia*-*Commiphora* bushland, 700–865 m. Vouchers: Watuma BM W0172 (EA, HIB), Jongh EG 130 (EA).

***Tephrosiavogelii* Hook.f.** – Habit: Subshrub. Habitat: Forest margin, waste grounds, old cultivations, 1350–1550 m. Voucher: Kabuye CHS 82/188 (EA).

***Tylosemafassoglense* (Kotschy ex Schweinf.) Torre & Hillc.** – Habit. Climber. Habitat: Wooded grassland, deciduous bushland, 600–865 m. Vouchers: Watuma BM W0198 (EA, HIB), Polhill RM & Kibuwa SP 960, Bally PRO 8591 (EA).

***Vachelliaancistroclada* (Brenan) Kyal. & Boatwr.** – Habit: Tree. Habitat: Dry scrub with trees, 550–1000 m. Vouchers: Medley KE 729 & 909 (EA).

***Vachelliabussei* (Harms ex Y.Sjöstedt) Kyal. & Boatwr.** – Habit: Shrub/Tree. Habitat: Deciduous bushland and dry scrub, 460–650 m. Vouchers: Dale IR 3884, King A 16/83, Medley KEM p.r. (EA).

**Vachelliaetbaica(Schweinf.)Kyal. & Boatwr.subsp.platycarpa (Brenan) Kyal. & Boatwr.** – Habit: Tree. Habitat: Deciduous bushland, dry scrub with trees, 550–1000 m. Vouchers: Faden RB & Faden AJ 74/268, Medley KE 845 (EA).

**Vachelliagerrardii(Benth.)P.J.H.Hurtervar.gerrardii** – Habit: Tree. Habitat: Woodland and wooded grassland, ca. 914 m. Voucher: Watuma BM W0324 (EA, HIB).

**Vachelliagerrardii(Benth.)P.J.H.Hurtervar.calvescens (Brenan) Kyal. & Boatwr.** – Habit: Shrub/Tree. Habitat: Riverine forest, wooded grassland. Voucher: Bally PRO 12013 (EA).

***Vachelliahockii* (De Wild.) Seigler & Ebinger** – Habit: Tree. Habitat: Secondary forest, *Acacia*-*Commiphora* woodland and bushland, 950–1371 m. Vouchers: Kabuye CHS 82/6, Mwachala G et al. EW776 & 2804, Mungai GM et al. EW1978 (EA).

**Vachelliahorrida(L.)Kyal. & Boatwr.subsp.benadirensis (Harms ex Y.Sjöstedt) Kyal. & Boatwr.** – Habit: Shrub/Tree. Habitat: *Acacia*-*Commiphora* bushland, open grassland, 609–1370 m. Vouchers: Kabuye CHS 82/122, Dale IR 3891 (EA).

**Vachellianilotica(L.)P.J.H.Hurter & Mabb.subsp.subalata (Vatke) Kyal. & Boatwr.** – Habit: Tree. Habitat: Woodland, open and clump bushland, 500–700 m. Vouchers: Kabuye CHS et al. NMK Exped. 639, Chemusto 9, Faden RB & Faden AJ 74/272, Medley KE 433, Hildebrandt JM 2591, Greenway PJ & Kanuri K 12958 (EA).

**Vachelliareficiens(Wawra & Peyr.)Kyal. & Boatwr.subsp.misera (Vatke) Kyal. & Boatwr.** – Habit: Shrub. Habitat: Dry *Acacia* bushland, 500–650 m. Voucher: Medley KE 758 (EA).

**Vachelliarobusta(Burch.)Kyal. & Boatwr.subsp.usambarensis (Taub.) Kyal. & Boatwr.** – Habit: Tree. Habitat: Riverine forest, woodland and bushland, 640–1000 m. Vouchers: Medley KE 972, Hucks M 967, Fagg CW 594 (EA).

***Vachelliastuhlmannii* (Taub.) Kyal. & Boatwr.** – Habit: Shrub. Habitat: Wooded grassland, deciduous bushlands, ca. 500 m. Voucher: Medley KE 733 (EA).

**Vachelliatortilis(Forssk.)Galasso & Banfisubsp.spirocarpa (Hochst. ex. A.Rich.) Kyal. & Boatwr.** – Habit: Tree. Habitat: Deciduous woodland and bushland on red sandy soil, 500–700 m. Vouchers: Faden RB & Faden AJ 74/267, Medley KE 428 (EA).

***Vachelliazanzibarica* (S.Moore) Kyal. & Boatwr.** – Habit: Shrub/Tree. Habitat: In wooded grassland, 457–650 m. Vouchers: Kakande M 36, King AL 12/83, Medley KE p.r. (EA).

***Vignakirkii* (Baker) J.B.Gillett** – Habit: Climber. Habitat: Sandy soils by rivers in semi-desert bushland, old cultivations, ca. 579 m. Voucher: Napier ER 994 (EA).

**VignamembranaceaA.Rich.subsp.caesia (Chiov.) Verdc.** – Habit: Climber. Habitat: Acacia-Commiphora bushland, ca. 600 m. Vouchers: Verdcourt B 5265, Friis F 97, Ossent JR 159, Leuthold W 116 (EA).

**VignaparkeriBakersubsp.maranguensis (Taub.) Verdc.** – Habit: Climber. Habitat: Forest, thicket, weed of cultivation, homesteads, 914–2000 m. Vouchers: SAJIT–004524, Kabuye CHS 82/101 & 115, Faden RB et al. NMK Exped. 32, Napier ER 1110, Bally PRO 8778, Gillett JB & Burtt BL 17070, Agriculture Dept. B867 (EA).

**Vignaunguiculata(L.)Walp.subsp.dekindtiana (Harms) Verdc.** – Habit: Herb. Habitat: Forest edges, grassland, 1100–1550 m. Vouchers: Wakanene KM & Mwangangi OM 329, Beentje HJ et al. NMK Exped. 841, Fleuret A 8 (EA).

**Vignavexillata(L.)A.Rich.var.vexillata** – Habit: Climber. Habitat: *Acacia*-*Commiphora* bushland, grassland, cultivations, 1040–1372 m. Vouchers: Watuma BM W0149 (EA, HIB), Mwachala G et al. EW376, Osborn RM 1 (EA).

***Wajirapraecox* (Verdc.) Thulin & Lavin** – Habit: Liana. Habitat: Dry *Acacia*, *Terminalia*, *Commiphora* bushland, ca. 640 m. Vouchers: Faden RB & Faden AJ 74/236, Mr & Mrs Hucks M 449 & 840 (EA).

**ZorniacapensisPers.subsp.tropica Milne-Redh.** – Habit: Herb. Habitat: Rocky places in dry scrub on shallow soil, ca. 500 m. Voucher: Luke WRQ & Luke PA 5577 (EA).

**F82. FRANCOACEAE**

1 Genus, 1 species

***Bersamaabyssinica* Fresen.** – Habit: Tree. Habitat: Secondary evergreen rainforest, 1140–1850 m. Vouchers: Watuma BM W0241 (EA, HIB), Faden RB et al. NMK Exped. 555 (EA).

**F83. GELSEMIACEAE**

1 Genus, 1 species

***Mostueabrunonis* Didr.** – Habit: Shrub. Habitat: Upland rainforest. Voucher: Bally PRO 8710 (EA).

**F84. GENTIANACEAE**

1 Genus, 1 species

***Anthocleistagrandiflora* Gilg** – Habit: Herb. Habitat: Upland rainforest, riverine forest, 1225–1550 m. Vouchers: Wakanene KM & Mwangangi OM 690, Mwachala G et al. EW1868, Beentje et al. NMK Exped. 1171, Faden RB et al. 70/521 (EA).

**F85. GERANIACEAE**

2 Genera, 3 species

***Geraniumarabicum* Forssk.** – Habit: Herb. Habitat: Upland rainforest margins, bushland and grassland, 1400–2170 m. Vouchers: Faden RB et al. NMK Exped. 6, Faden RB et al. 70/506, Christenhusz MJM, Kamau P, Mbale M & Chase MW 6654 (EA).

***Pelargoniummultibracteatum* Hochst. ex A.Rich.** – Habit: Herb. Habitat: Upland rainforest edges, wooded and open grassland, bushland, 875–1800 m. Vouchers: Watuma BM W0192 (EA, HIB), Bally PRO 8758 (EA).

***Pelargoniumquinquelobatum* Hochst. ex A.Rich.** – Habit: Herb. Habitat: Forest edges, bushland and thicket in rocky slopes, cultivated ground, 800–950 m. Voucher: Kabuye CHS et al. NMK Exped. 744 (EA).

**F86. GESNERIACEAE**

1 Genera, 6 species

***Streptocarpuscaulescens* Vatke** – Habit: Herb. Habitat: Moist areas in forest and forest margins, 914–2195 m. Vouchers: SAJIT–004124, 006381, 006390 & 006397, Watuma BM W0016 & W0095 (EA, HIB), Luke WRQ & Luke PA 4110 & 5549, Kirika P et al. NMK 589, Wakanene KM & Mwangangi OM 259 & 409, Christenhusz MJM et al. 6650, Beentje HJ 2150, Faden RB et al. NMK Exped. 4, 194 & 393, Bytebier B 1936 & 1941, Faden RB et al. 69/439, 71/38 & 47, Gillett JB, Burtt BL & Osborn RM 17049, Verdcourt B & Polhill RM 2727, Drummond RB & Hemsley JH 4295, Burtt BL 3823, Hildebrandt JM s.n. (EA).

***Streptocarpusglandulosissimus* Engl.** – Habit: Herb. Habitat: Damp shaded areas in forest and forest margins. Vouchers: Joanna B 8997, Bally PRO 13513 (EA).

^**NE**^***Streptocarpuskirkii* Hook.f.** – Habit: Herb. Habitat: Forest, 1425–1850 m. Vouchers: SAJIT–006393 & 006424 (EA, HIB), Luke WRQ & Luke PA 4154, Faden RB et al. NMK Exped. 360, Bytebier B 1938, Faden RB et al. 71/38A, Burtt BL 3831, Faden RB & Githui M 70/751, Bally PRO 13605 (EA).

***Streptocarpusmontanus* Oliv.** – Habit: Herb. Habitat: Shaded areas in wet montane or sub-montane forest, 1000–2170 m. Vouchers: SAJIT–006347 & 006414 & 006425 (EA, HIB), Luke WRQ & Luke PA 4200, Faden RB et al. NMK Exped. 83 & 196, Bytebier B 1937 & 1940, Drummond RB & Hemsley JH 4319, Gillett JB, Burtt BL & Osborn RM 17091, Faden RB et al. 69/480A & 71/151, Lavranos J 11806, Burtt BL 3830 (EA). Near Threatened.

**^NE^*Streptocarpussaxorum* Engl.** – Habit: Herb. Habitat: Semi-deciduous woodland thicket on cliff faces with constant mist, 600–1070 m. Vouchers: Faden RB, Evans A & Msafiri F 70/972, Knox EB 2716, Gardner HM 3006, Glover 2648, Burtt BL 4824 (EA).

**^E^*Streptocarpusteitensis* (B.L.Burtt) Christenh.** – Habit: Herb. Habitat: Forest rock crevices, cliff faces, 1400–1750 m. Vouchers: SAJIT–005323 & 006426, Watuma BM W0024 (EA, HIB), Simiyu 174, Joanna B 8982, Beentje HJ et al. NMK Exped. 1170 (EA). Critically Endangered.

**F87. GISEKIACEAE**

1 Genus, 1 species

***Gisekiapharnaceoides* L.** – Habit: Herb. Habitat: Ruderal sites, roadsides, cultivated lands, bushland, woodland, 600–914 m. Vouchers: Napier ER 941, Hucks M 708 & 786, MacDonald J 858 (EA).

**F88. HAMAMELIDACEAE**

1 Genus, 1 species

**TrichocladusellipticusEckl. & Zeyh.subsp.malosanus (Baker) Verdc.** – Habit: Tree. Habitat: Upland rainforest, often by streams, 900–1200 m. Voucher: Medley KE 648 (EA).

**F89. HYDNORACEAE**

1 Genus, 1 species

***Hydnoraabyssinica* A.Br.** – Habit: Herb. Habitat: Dry woodland, wooded grassland, bushland, 800–1150 m. Vouchers: Mkala EM 0001 (EA, HIB), Mrs. Robertson SA 6537 (EA).

**F90. HYPERICACEAE**

1 Genus, 3 species

***Hypericumbequaertii* De Wild.** – Habit: Shrub. Habitat: Upland evergreen bushland, ca. 1250 m. Voucher: Mungai GM et al. EW3201 (EA).

***Hypericumconjungens* N.Robson** – Habit: Herb. Habitat: Forest margins, grassland, dry bush, ca. 1240 m. Voucher: Mwachala G et al. EW3191 (EA).

***Hypericumpeplidifolium* A.Rich.** – Habit: Herb. Habitat: Upland grassland, streams and marshes, roadside banks, 1466–1814 m. Vouchers: SAJIT–006399 (EA, HIB), Kabuye CHS 82/109, Mwachala G et al. EW210 (EA).

**F91. ICACINACEAE**

1 Genus, 1 species

***Pyrenacanthamalvifolia* Engl.** – Habit: Liana. Habitat: Dry *Acacia*-*Commiphora* or semi-evergreen bushland and scrub, 500–1650 m. Vouchers: Muchiri J 245, Semlingwa J 20, Leeuwenberg AJM 10783, Hucks M 458, Jeffrey C et al. EPK269, Hildebrandt JM 2355 (EA).

**F92. LAMIACEAE**

20 Genera, 50 species

***Aeollanthusdensiflorus* Ryding** – Habit: Subshrub. Habitat: Rock crevices, 1700–1875 m. Vouchers: Gillett JB 18750, Drummond & Hemsley 4389, Faden RB et al. NMK Exped. 300 (EA).

***Aeollanthusrepens* Oliv.** – Habit: Herb. Habitat: Forest edges, shallow soils on rocks, 810–1875 m. Vouchers: SAJIT–006395, Watuma BM W0113 (EA, HIB), Mwachala G et al. EW757 & 1327, Wakanene KM & Mwangangi OM 739, Beentje HJ et al. NMK Exped. 814 & 858, Medley KE 463, Faden RB et al. NMK Exped. 488 (EA).

**ClerodendrumcephalanthumOliv.subsp.cephalanthum** – Habit: Shrub. Habitat: Evergreen forest, 1425–1850 m. Voucher: Faden RB et al. NMK Exped. 359 (EA).

**ClerodendrumhildebrandtiiVatkevar.hildebrandtii** – Habit: Shrub/Tree. Habitat: Forest edges, bushland, thickets in scattered tree grassland, 600–1000 m. Vouchers: Medley KE 704 & 945, Hildebrandt JM 2389 (EA).

**ClerodendrumjohnstoniiOliv.subsp.johnstonii** – Habit: Shrub/Liana. Habitat: Moist forest edges, secondary growth, 1600–1875 m. Vouchers: Watuma BM W0130 (EA, HIB), Faden RB et al. NMK Exped. 216 & 505, Drummond RB & Hemsley JH 4366, Raler I 0028 (EA).

**Clinopodiumabyssinicum(Hochst. ex Benth.)Kuntzevar.abyssinicum** – Habit: Subshrub. Habitat: Upland evevrgreen bushland and grassland, ca. 1600 m. Voucher: Beentje HJ et al. NMK Exped. 1160 (EA).

***Coleusaegyptiacus* (Forssk.) A.J.Paton** – Habit: Subshrub. Habitat: Rocky *Acacia*-*Commiphora* bushland, abandoned cultivations, 800–950 m. Voucher: Kabuye CHS et al. NMK Exped. 713 (EA).

**Coleus sp. nr. amboinicus Lour.** – Habit: Herb. Habitat: Dry open scrub on red sandy soils, ca. 777 m. Voucher: Kirika P, Rory M, Mbale M NMK 592 (EA).

***Coleusautranii* Briq.** – Habit: Shrub. Habitat: Forest, secondary forest, *Philippia* bushland, 1900–2170 m. Vouchers: Faden RB et al. NMK Exped. 135, Gillett JB, Burtt BL & Osborn RM 17094, Drummond RB & Hemsley JH 4312 (EA).

***Coleusbarbatus* (Andrews) Benth. ex G.Don** – Habit: Shrub. Habitat: Forest margins, moist bushland, cultivations, 650–1792 m. Vouchers: Watuma BM W0100 (EA, HIB), Sacleux C 2580, Drummond RB & Hemsley JH 4370, Medley KE 824, Kluguess LM 2, Bally J 13531, Nattrass MS B1158 (EA).

**Coleuscaninus(B.Heyne ex Roth)Vatkesubsp.flavovirens (Gürke) A.J.Paton** – Habit: Herb. Habitat: Base of rock outcrops, eroded grassland and *Commiphora* scrub, ca. 1220 m. Voucher: Mungai GM et al. EW3196 (EA).

***Coleuscomosus* Hochst. ex Gürke** – Habit: Subshrub. Habitat: *Philippia* heath, rock crevices, cliffs, 2000–2070 m. Vouchers: Drummond RB & Hemsley JH 4305, Bjørnstad IN IB733 (EA).

***Coleuscylindraceus* (Hochst. ex Benth.) A.J.Paton.** – Habit: Herb. Habitat: *Acacia*-*Commiphora* and dry evergreen bushland on rocky outcrops, 609–777 m. Vouchers: Kirika P, Rory M, Mbale M NMK 591, Warao M 63 (EA).

***Coleusgracilis*** Gürke – Habit: Shrub. Habitat: Scrub or thicket on rocky slopes on dry riverbeds, 800–950 m. Voucher: Kabuye CHS et al. NMK Exped. 645 (EA).

***Coleusgrandicalyx* E.A.Bruce** – Habit: Herb. Habitat: Crevices on rocky outcrops on hillslopes, 1740–2100 m. Voucher: Beentje HJ et al. NMK Exped. 903 (EA).

***Coleushadiensis* (Forssk.) A.J.Paton** – Habit: Herb. Habitat: *Acacia*-*Commiphora* and dry evergreen bushland on rocky outcrops, 985–1600 m. Vouchers: Mwachala G et al. EW3177, Faden RB & Faden AJ 74/496, Drummond RB & Hemsley JH 4387, Greenway PJ 10464 (EA).

***Coleusigniarius* Schweinf.** – Habit: Shrub. Habitat: Dry rocky hillsides, *Acacia*-*Commiphora* bushland, ca. 920 m. Voucher: Mwachala G et al. EW3257 (EA).

***Coleuslactiflorus* Vatke** – Habit: Shrub. Habitat: Rocky hillsides, open forest, Acacia woodland, roadsides, 600–1850 m. Vouchers: Beentje HJ et al. NMK Exped. 1042, Gillett JB 18758, Hildebrandt JM 2522 (EA).

***Coleuslanuginosus* Hochst. ex Benth.** – Habit: Herb. Habitat: Forest and forest clearings on rocky outcrops, roadsides, 1400–1640 m. Vouchers: Faden RB et al. NMK Exped. 20 (EA).

***Coleuslongipetiolatus*** Gürke – Habit: Subshrub. Habitat: Openings and margins of evergreen forest, 900–1300 m. Voucher: Kokwaro JO 4244 (EA).

***Coleusmelleri* (Baker) A.J.Paton & Phillipson** – Habit: Subshrub. Habitat: Moist montane margins and clearings, semi-deciduous forest, 700–1600 m. Vouchers: Medley KE 935, Bally PRO 13576 (EA).

***Coleusmeyeri* (Gürke) A.J.Paton** – Habit: Shrub. Habitat: Montane forest and associated bushland, 1800–2100 m. Vouchers: SAJIT–004586 (EA, HIB), Drummond RB & Hemsley JH 4317 (EA).

***Coleusnyikensis* Baker** – Habit: Shrub. Habitat: Rocks in grassland or steep slopes, ca. 1500 m. Voucher: Luke WRQ & Luke PA 4198 (EA).

***Coleusotostegioides* (Schweinf. ex Gürke) A.J.Paton** – Habit: Subshrub. Habitat: Dry *Acacia*-*Commiphora* bushland, often in rocky areas, ca. 600–1100 m. Vouchers: Mwachala G et al. EW212, Gillett JB & Burtt BL 17174, Mrs. Robertson SA 6541 (EA).

***Coleusprostratus* (Gürke) A.J.Paton** – Habit: Herb. Habitat: *Acacia* scrub often on rocks, ca. 914 m. Vouchers: Ochung HK 95, Greensmith P 16236 (EA).

**^NE^*Coleustriangularis* (A.J.Paton) A.J.Paton** – Habit: Herb. Habitat: *Ocotea*-*Podocarpus* evergreen forest, 1425–2100 m. Vouchers: Luke WRQ & Luke PA 5497 & 10806, Faden RB et al. NMK Exped. 222, Beentje HJ et al. NMK Exped. 789, Gillett JB & Burtt BL 17112, Faden RB et al. 70/735, Seki AH et al. JKCAT 1586, Drummond RB & Hemsley JH 4338, Bally PRO 8752, Dale IR 3777, Karimi s.n. (EA). Near Threatened.

***Coleusumbrosus* Vatke** – Habit: Herb. Habitat: Deciduous woodland and bushland, 610–850 m. Vouchers: Gilbert MG & Gilbert CI 6124, Hildebrandt JM 2424 (EA).

***Equilabiumkamerunense* (Gürke) Mwany. & A.J.Paton** – Habit: Herb. Habitat: Forest margins, ca. 1800 m. Voucher: Drummond RB & Hemsley JH 4310 (EA).

***Equilabiumlaxiflorum* (Benth.) Mwany. & A.J.Paton** – Habit: Herb. Habitat: Forest margins and clearings, 1400–2170 m. Vouchers: Faden RB et al. NMK Exped. 96 & 268, Verdcourt B & Polhill RM 2733, Gillett JB 17247, Lavranos JJ & Newton LE 17643, Drummond RB & Hemsley JH 4310, Napier ER 1133 (EA).

***Equilabiumlongipes* (Baker) Mwany. & A.J.Paton** – Habit: Subshrub. Habitat: Seasonally dry bushland or woodland in rocky places, 520–1850 m. Vouchers: Beentje HJ et al. NMK Exped. 367, Faden RB & Faden AJ 74/478, 497 & 508, Gillett JB 17206 (EA).

***Hoslundiaopposita* Vahl** – Habit: Shrub. Habitat: Forest, woodland, open grassland, 550–1371 m. Vouchers: Mwachala G et al. EW97, Msafiri F & Ojiambo L 279, Napier ER 953 & 1079, Rauh W 67, Medley KE 493 (EA).

***Leucasgrandis* Vatke** – Habit: Herb. Habitat: Upland evergreen bushland, forest undergrowth and margins, 610–1792 m. Vouchers: Watuma BM W0101 (EA, HIB), Hilderbrandt JM 2423, Johnston HH s.n. (EA).

***Leucasneuflizeana* Courbon** – Habit: Herb. Habitat: Deciduous bushland, roadsides, rock outcrops and hillsides, 610–950 m. Vouchers: Kabuye CHS et al. NMK Exped. 597, Hildebrandt JM 2404 (EA).

**^NE^LeucasoligocephalaHook.f.var.usambarica Sebald** – Habit: Herb. Habitat: Phillipia thicket openings, grassland, ca. 2050 m. Voucher: Gillett JB 17257 (EA).

*Mesosphaerumpectinatum (L.) Kuntze – Habit: Herb. Habitat: Edge of rivers and swamps in thickets and disturbed places, ca. 900 m. Voucher: Mrs. Robertson SA 3568 (EA).

**Micromeriaimbricata(Forssk.)C.Chr.var.imbricata** – Habit: Subshrub. Habitat: Open woodland or shrubland on sloping ground, 1200–1580 m. Vouchers: Kabuye CHS 82/110, Joanna B 8821 (EA).

***Ocimumamericanum* L.** – Habit: Herb. Habitat: Bushland, cultivated and disturbed ground, 503–1010 m. Vouchers: Mwachala G et al. EW131, Napier ER 1002, Medley KE 492, Hucks M 719, Greenway PJ & Kanuri K 13024 (EA).

**OcimumgratissimumL.subsp.gratissimum** – Habit: Herb. Habitat: Edges of dry montane forest, disturbed places along river, 900–1850 m. Vouchers: Watuma BM W0109 (EA, HIB), Mwachala G et al. EW818, Wakanene KM & Mwangangi OM 611, Murray High School 18 (EA).

***Orthosiphonthymiflorus* (Roth) Sleesen** – Habit: Herb. Habitat: Open dry forest, wooded grassland, disturbed places, ca. 1000 m. Voucher: Drummond RB & Hemsley JH 4408 (EA).

***Platostomahildebrandtii* (Vatke) A.J.Paton & Hedge** – Habit: Subshrub. Habitat: Open *Acacia*-*Commiphora* or *Combretum* bushland on rocky ground, 800–1000 m. Vouchers: Kabuye CHS et al. NMK Exped. 598, Hildebrandt JM 2414, Drummond RB & Hemsley JH 4412, Polhill RM & Kibuwa SP 959 (EA).

***Plectranthusalboviolaceus*** Gürke – Habit: Shrub. Habitat: Evergreen forest, by paths or rivers, 900–1200 m. Voucher: Bally PRO 13300 (EA).

***Premnahildebrandtii*** Gürke – Habit: Shrub/Tree. Habitat: Woodland, riverine thicket, 800–1525 m. Vouchers: Joanna B & Opiko 8777, Bally PRO 8639 (EA).

***Premnaoligotricha* Baker** – Habit: Shrub. Habitat: *Acacia*-*Commiphora* bushland and woodland, 520–1000 m. Vouchers: Faden RB & Faden AJ 74/476 & 507, Medley KE 481 & 772, Elliot S 6202 (EA).

**Rothecamyricoides(Hochst.)Steane & Mabb.subsp.myricoides** – Habit: Shrub. Habitat: Forest, grassland, scrub thicket, open woodland, 1250–1400 m. Vouchers: Mwachala G et al. EW385, 591 & 1252, Luke WRQ et al. 5550, Bally JB 13508 (EA).

**StachysaculeolataHook.f.var.aculeolata** – Habit: Herb. Habitat: Forest and secondary forest margins, 1800–2100 m. Vouchers: Drummond RB & Hemsley JH 4325, Faden RB et al. NMK Exped. 137 & 1132 (EA).

***Tetradeniaurticifolia* (Baker) Phillipson** – Habit: Shrub. Habitat: Forest margins, woodland and bushland, 700–1571 m. Vouchers: Watuma BM W0206 (EA, HIB), Nattrass RM B1156 (EA).

**TinneaaethiopicaKotschy ex Hook.f.subsp.aethiopica** – Habit: Subshrub. Habitat: Deciduous woodland and bushland, riverine forest and scrub, 750–1200 m. Vouchers: Watuma BM W0148 (EA, HIB), Goyder et al. 4019 (EA).

**TinneaaethiopicaKotschy ex Hook.f.subsp.litoralis Vollesen** – Habit: Subshrub. Habitat: *Acacia*-*Commiphora* bushland, 550–650 m. Vouchers: Medley KE 482 & 984 (EA).

**TinneaaethiopicaKotschy ex Hook.f.subsp.stolzii (Robyns & Lebrun) Vollesen** – Habit: Subshrub. Habitat: Deciduous woodland and bushland, ca. 900 m. Voucher: Luke WRQ & Luke PA 4096 (EA).

**VitexfischeriGürkevar.keniensis (Turrill) Meerts** – Habit: Tree. Habitat: Evergreen forest, 1386–1875 m. Vouchers: Watuma BM W0236 (EA, HIB), Faden RB et al. NMK Exped. 466 (EA). Endangered.

***Vitexstrickeri* Vatke & Hildebr.** – Habit: Shrub. Habitat: Forest edges, bushland, thickets, rocky outcrops, 850–1875 m. Vouchers: Kabuye CHS 82/51, Mwachala G et al. EW1206, Faden RB et al. NMK Exped. 515 & 1106, Gardner HM 2989, Kluguess LM 50 (EA).

***Volkameriaeriophylla* (Gürke) Mabb. & Y.W.Yuan** – Habit: Shrub. Habitat: Grassland, *Acacia*-*Commiphora*-*Combretum* bushland and scrub, 762–1100 m. Vouchers: Cheseny CMC 27/72, Polhill RM & Kibuwa SP 973 (EA).

**F93. LAURACEAE**

4 Genera, 4 species

*Cinnamomumcamphora (L.) J.Presl – Habit: Tree. Habitat: Remnant mist forest fragment, 1700–1800 m. Vouchers: Mwachala G et al. EW106, Christenhusz MJM, Kamau P, Mbale M, Chase MW & Kyaa J 6679 (EA).

***Cryptocaryaliebertiana* Engl.** – Habit: Tree. Habitat: Evergreen forest, by rivers, 1425–1850 m. Voucher: Faden RB et al. NMK Exped. 957 (EA).

***Kuloausambarensis* (Engl.) Trofimov & Rohwer** – Habit: Tree. Habitat: Intermediate to montane evergreen wet forest, 1600–1978 m. Vouchers: Watuma BM W0306 (EA, HIB), Faden RB & Githui M 70/746, Faden RB, Faden AJ & Smeenk C 71/1019, Faden RB & Faden AJ 72/267 (EA).

***Ocoteakenyensis* (Chiov.) Robyns & R.Wilczek** – Habit: Tree. Habitat: Upland rainforest, 1440–1875 m. Vouchers: Faden RB et al. NMK Exped. 212, Faden RB, Evans A, Msafiri & Smeenk C 71/48 (EA). Vulnerable.

**F94. LINDERNIACEAE**

3 Genera, 3 species

***Craterostigmaplantagineum* Hochst.** – Habit: Herb. Habitat: Shallow soil over rock, 1850–1870 m. Voucher: Watuma BM W0065 (EA, HIB).

^**NE**^***Linderniellabrevidens* (Skan) Eb.Fisch., Schäferh. & Kai Müll.** – Habit: Herb. Habitat: Rocky hillsides, forest clearings, weed of cultivation, 1700–2200 m. Vouchers: Faden RB et al. NMK Exped. 494, Friis I & Hansen OJ 2676 (EA).

***Stemodiopsisbuchananii* Skan** – Habit: Herb. Habitat: Crevices in boulders and rock cliffs, 686–750 m. Vouchers: Faden RB & Faden AJ 74/258, Verdcourt B 3896, Bally PRO 8800 (EA).

**F95. LOGANIACEAE**

1 Genus, 7 species

***Strychnosdecussata* (Pappe) Gilg** – Habit: Tree. Habitat: Deciduous bushland near rocks, 450–880 m. Vouchers: Medley KE 416, 465 & 654, Gillett JB 21066, Joanna B 8853 (EA).

***Strychnoshenningsii* Gilg** – Habit: Shrub/Tree. Habitat: Upland rainforest, semi-evergreen bushland, riverine forest, 650–1850 m. Vouchers: SAJIT–005413 (EA, HIB), Faden RB et al. NMK Exped. 785, Faden RB, Faden AJ, Smeenk C, Smeenk N & McNee J 71/974, Medley KE 484 & 917 (EA).

***Strychnosmadagascariensis* Poir.** – Habit: Shrub/Tree. Habitat: Deciduous bushland and woodland, 650–1140 m. Vouchers: Mwachala G et al. EW141, Mungai GM et al. EW2658, Medley KE 541, 724 & 749, Joanna B 8895 (EA).

***Strychnosspinosa* Lam.** – Habit: Shrub/Tree. Habitat: Deciduous bushland and woodland, 550–1000 m. Vouchers: Kabuye CHS et al. NMK Exped. 600, Mwachala G et al. EW963, 1571 & 3329, Medley KE 576, 613 & 950 (EA).

***Strychnosmitis* S.Moore** – Habit: Tree. Habitat: Upland rainforest, riverine forest, 1200–1850 m. Vouchers: SAJIT–005389 (EA, HIB), Raler I 0066, Faden RB & Githui M 70/723 (EA).

***Strychnospanganensis* Gilg** – Habit: Shrub. Habitat: Rainforest, evergreen bushland, ca. 1150 m. Voucher: Mwachala G et al. EW582 (EA).

***Strychnosusambarensis* Gilg** – Habit: Shrub/Tree. Habitat: Upland rainforest, evergreen bushland, ca. 950 m. Voucher: Mwachala G et al. EW1276 (EA).

**F96. LOPHIOCARPACEAE**

1 Genus, 1 species

***Corbichoniadecumbens* (Forssk.) Exell** – Habit: Herb. Habitat: Open bushland, grassland, roadside and cultivations, 579–600 m. Voucher: Napier ER 989 (EA).

**F97. LORANTHACEAE**

8 Genera, 15 species

***Agelanthusheteromorphus* (A.Rich.) Polhill & Wiens** – Habit: Shrub. Habitat: Bushland, 960–1000 m. Vouchers: Mwachala G et al. EW3555, van Someren C et al. NMK Exped. 1033, 1034 & 1035 (EA).

***Agelanthusoehleri* (Engl.) Polhill & Wiens** – Habit: Shrub. Habitat: Deciduous bushland, grassland with scattered trees, 600–1200 m. Vouchers: Wakanene KM & Mwangangi OM 482, Gillett JB 16875 (EA).

***Agelanthussansibarensis* (Engl.) Polhill & Wiens** – Habit: Shrub. Habitat: Montane and dry hill forest, bushland, 609–1402 m. Vouchers: SAJIT–005403 (EA, HIB), Mwachala G et al. EW762 & 1347, Joanna B 8889, Josnell I 744 (EA).

***Emelianthepanganensis* (Engl.) Danser** – Habit: Shrub. Habitat: Deciduous bushland on *Euphorbia*, *Adansonia* & Sterculia, 500–650 m. Vouchers: van Someren C et al. NMK Exped. 1038, Polhill RM 4772, Archer PG 450, Gardiner N 3012, Napier ER 1003 (EA).

**Emelianthepanganensis(Engl.)Dansersubsp.commiphorae Wiens & Polhill** – Habit: Shrub. Habitat: Deciduous bushland, wooded grassland on *Commiphora*, *Sterculia* and *Lannea*, ca. 500–700 m. Voucher: Hucks M 451 (EA).

***Englerinadrummondii* Balle ex Polhill & Wiens** – Habit: Herb. Habitat: Dry evergreen forest, hilltop mist forest, wooded grasslands, 1040–1640 m. Vouchers: Kabuye CHS 82/46, Mwachala G et al. 223, 224 & EW810, Luke WRQ et al. 4133 (EA). Vulnerable.

**Englerina sp. nr. kagehensis (Engl.) Polhill & Wiens** – Habit: Herb. Habitat: Upland forest, 1230–1300 m. Voucher: Faden RB et al. 71/999 (EA).

***Englerinawoodfordioides* (Schweinf.) Balle** – Habit: Shrub. Habitat: Montane or riverine forest and associated bushland, 1371–1402 m. Vouchers: Joanna B 8876 & 8925 (EA).

***Erianthemumcommiphorae* (Engl.) Danser** – Habit: Shrub. Habitat: Deciduous bushland on *Acacia*, *Commiphora*, *Euphorbia* and *Lannea*, 600–800 m. Voucher: Joanna B 8993 (EA).

***Erianthemumdregei* (Eckl. & Zeyh.) Tiegh.** – Habit: Shrub. Habitat: Forest edges, woodland, bushland and disturbed places, 960–1875 m. Vouchers: Mwachala G et al. EW608 & 1000, Wakanene KM & Mwangangi OM 449, Joanna B & Opiko B 8768 & 8768A, Faden RB et al. NMK Exped. 336, Medley KE 819, Hucks M 895 (EA).

***Helixantherakirkii* (Oliv.) Danser** – Habit: Shrub. Habitat: Deciduous woodland, 700–900 m. Voucher: SAJIT–005406 (EA, HIB).

***Oliverellahildebrandtii* (Engl.) Tiegh.** – Habit: Shrub. Habitat: Deciduous bushland, 650–1850 m. Vouchers: Mwachala G et al. EW3115A, Luke WRQ & Bytebier B 5258, Hildebrandt JM 2523, Beentje HJ et al. NMK Exped. 1016, Faden RB, Evans A, Rathbun G 69/418, Medley KE 798, Napper D 950, Gilbert MG 6100, Joanna B & Opiko 8778 & 9078 (EA).

***Oncocalyxfischeri* (Engl.) M.G.Gilbert** – Habit: Shrub. Habitat: Dry evergreen forest, 950–1270 m. Vouchers: Mwachala G et al. EW3155, Gillett JB 19581 (EA).

***Plicosepaluscurviflorus* (Benth. ex Oliv.) Tiegh.** – Habit: Shrub. Habitat: Deciduous bushland and *Acacia* woodland, 503–1402 m. Vouchers: Mwachala G et al. EW382 & 382A, Gilbert MG & Gilbert CI 6123 & 6123A, Medley KE 764, Bally PRO 8552, Joanna B & Opiko 8870 (EA).

***Plicosepalusmeridianus* (Danser) Wiens & Polhill** – Habit: Shrub. Habitat: Deciduous bushland on *Commiphora* and *Acacia*, 610–900 m. Vouchers: SAJIT–005370 (EA, HIB), Medley KE 782 (EA).

***Plicosepalussagittifolius* (Engl.) Danser** – Habit: Shrub. Habitat: Deciduous bushland and *Acacia* woodland, 600–1000 m. Vouchers: Mungai GM et al. EW1317, Cheseny CMC 16/72, Gillett JB 19587, Hucks M 959 (EA).

**F98. LYTHRACEAE**

1 Genus, 1 species

***Ammanniaprieuriana* Guill. & Perr.** – Habit: Herb. Habitat: Along rivers and damp places, ca. 1100 m. Voucher: Mungai G et al. EW1307 (EA).

**F99. Malphigiaceae**

2 Genera, 2 species

***Caucanthusauriculatus* (Radlk.) Nied.** – Habit: Liana. Habitat: Dry evergreen forest, deciduous woodland and bushland, 650–1000 m. Vouchers: Watuma BM W0182 (EA, HIB), Mwachala G et al. EW857, Medley KE 867 & 937 (EA).

^**E**^***Acridocarpustaitensis* Mwadime, Ngumbau & Q.Luke** – Habit: Liana. Habitat: Forest margins, 1728–1800 m. Vouchers: Watuma BM W0061 (EA, HIB), Mwadime N 2379, Faden RB et al. NMK Exped. 525, Drummond & Hemsley 4331 (EA).

**F100. MALVACEAE**

21 Genera, 58 species

***Abutilonlongicuspe* Hochst. ex A.Rich.** – Habit: Subshrub. Habitat: *Acacia* woodland, forest and forest edges, 1250–1676 m. Vouchers: Mwachala G et al. EW1250, Kluguess LM 22 (EA).

**Abutilonmauritianum(Jacq.)Medik.subsp.mauritianum** – Habit: Subshrub. Habitat: Upland bushland, forest edges, *Acacia* woodland, roadside, cultivations, 686–1569 m. Vouchers: Watuma BM W0281 (EA, HIB), Verdcourt B 3890, Hildebrandt JM 2633 (EA).

**Abutilonmauritianum(Jacq.)Medik.var.epilosum Verdc.** – Habit: Subshrub. Habitat: *Acacia*-*Commiphora* bushland and thicket, forest edges and clearings, ca. 1050 m. Voucher: Luke WRQ & Luke PA 5309 (EA).

**^NE^Abutilonmauritianum(Jacq.)Medik.var.grandiflorum Verdc.** – Habit: Subshrub. Habitat: Forest, near stream, secondary scrub, bushland, ca. 1350–1650 m. Vouchers: Jex-Blake M 2313, Joanna B 8965, Gardner HM 2984 (EA).

***Adansoniadigitata* L.** – Habit: Tree. Habitat: Woodland, deciduous bushland, 600–1250 m. Vouchers: Joanna B 9056, Medley KE p.r. (EA).

*Callianthemegapotamica (A.Spreng.) Dorr – Habit: Shrub. Habitat: Hillslopes around homes and cultivated lands, ca. 1500 m. Voucher: SAJIT–004120 (EA, HIB).

***Colagreenwayi* Brenan** – Habit: Tree. Habitat: Evergreen forest, 1425–1875 m. Vouchers: Faden RB et al. NMK Exped. 458 & 771, Beentje HJ et al. NMK Exped. 886, Faden RB, Evans A Rathbun G 69/463, Faden RB & Githui M 70/721, Faden RB & Faden AJ 71/975, Medley KE 440 & 999 (EA).

***Corchorustridens* L.** – Habit: Herb. Habitat: Cultivated lands, sandy and water-logged soils, ca. 609 m. Voucher: Napier ER 904 (EA).

***Corchorusurticifolius* Wight & Arn.** – Habit: Herb. Habitat: Dense *Commiphora* bushland on sandy soils, 800–1100 m. Voucher: Mwachala G et al. EW569 (EA).

***Dombeyakirkii* Mast.** – Habit: Shrub/Tree. Habitat: Forest edge, *Acacia* bushland on rocky slopes, 650–1000 m. Vouchers: SAJIT–005378 (EA, HIB), Kabuye CHS et al. NMK Exped. 737, Mwachala G et al. EW969, Maikweki J 2, Medley KE 455, Gillett JB 17229, Gardner HM 2985, Verdcourt B 5319, Agnew ADQ & Agnew S 11185 (EA).

***Dombeyarotundifolia* (Hochst.) Planch.** – Habit: Tree. Habitat: Forest edge, woodland, wooded grassland, 1200–1676 m. Vouchers: Kabuye CHS 82/147, Mungai GM et al. EW3171, Gardner HM 1200 (EA).

***Dombeyatorrida* (J.F.Gmel.) Bamps** – Habit: Tree. Habitat: Open forest and forest edges, disturbed forest, 2000–2170 m. Voucher: Faden RB et al. NMK Exped. 81 (EA).

***Gossypioideskirkii* (Mast.) Skovst. Ex J.B.Hutch.** – Habit: Shrub. Habitat: Forest edges, bushland with scattered trees, 650–1000 m. Voucher: Medley KE 579 (EA).

***Grewiaarborea* (Forssk.) Lam.** – Habit: Shrub. Habitat: Open woodland, *Acacia*-*Commiphora* bushland, thicket, 650–1000 m. Voucher: Goyder DJ, Masinde PS, Meeve U, Whitehouse C et al. 4016, Faden RB & Faden AJ 74/270, Medley KE 849 (EA).

***Grewiabicolor* Juss.** – Habit: Shrub. Habitat: Woodland, *Commiphora*-*Acacia* bushland, 500–950 m. Vouchers: SAJIT–005390 (EA, HIB), Kabuye CHS et al. NMK Exped. 640, Mwachala G et al. EW77 & 508, Bally PRO 8624, Napier ER 986, Zùmer M 54 (EA).

***Grewiaforbesii* Harv. ex Mast.** – Habit: Shrub/Liana. Habitat: Evergreen dense bushland, deciduous woodland, secondary vegetation, 550–1000 m. Vouchers: SAJIT– 004630 & 005407, Watuma BM W0011 (EA, HIB), Medley KE 450 & 530, Faden RB & Faden AJ 74/501 (EA).

***Grewiamollis* Juss.** – Habit: Shrub. Habitat: Forest, open woodland, *Combretum* open grassland, 1200–1467 m. Vouchers: Watuma BM W0242 (EA, HIB), Wakanene KM & Mwangangi OM 475 (EA).

***Grewianematopus* K.Schum.** – Habit: Shrub. Habitat: Woodland, bushland, open bushland, 600–950 m. Vouchers: Medley KE 902, Faden RB & Faden AJ 74/439, Gillett JB, Burtt BL 17176, Engler A 1941, Archer PG 262, Ossent J s.n. (EA).

***Grewiaplagiophylla* K.Schum.** – Habit: Shrub. Habitat: Forest, riverine thicket, *Acacia*-*Commiphora* bushland, 450–1000 m. Vouchers: Medley KE 513, 697, 811 & 1021, Lavranos JJ 11892 (EA).

***Grewiasimilis* K.Schum.** – Habit: Shrub. Habitat: Woodland, thicket, bushland and grassland, 750–1750 m. Vouchers: SAJIT–004123, Watuma BM W0169 & W0320 (EA, HIB), Kabuye CHS 82/38 & 153, Mwachala G et al. EW47, 1195, 1253 & 3182, Wakanene KM & Mwangangi OM 545, Beentje HJ 3280, Medley KE 442, 575 & 982 (EA).

***Grewiatephrodermis* K.Schum.** – Habit: Shrub. Habitat: *Acacia* or *Commiphora* bushland, along dry water courses, 550–1070 m. Vouchers: Mwachala G et al. EW1371, Medley KE 421, 539 & 752 (EA).

***Grewiatrichocarpa* Hochst. ex A.Rich.** – Habit: Shrub. Habitat: Riverine forest, scattered tree grassland, *Acacia* bush, ca. 950 m. Voucher: Mwachala G et al. EW957 (EA).

***Grewiatruncata* Mast.** – Habit: Shrub. Habitat: Thicket and bushland, along river courses, 650–1000 m. Voucher: Medley KE 568 (EA).

***Grewiavillosa* Willd.** – Habit: Shrub. Habitat: Dry *Acacia*-*Commiphora* bushland, 550–882 m. Vouchers: Watuma BM W0180 (EA, HIB), Faden RB & Faden AJ 74/462, Lubai lK HB21, Medley KE p.r. (EA).

***Hermanniaexappendiculata* (Mast.) K.Schum. ex Engl.** – Habit: Shrub. Habitat: *Acacia*-*Commiphora* bushland, roadsides, cultivations, 660–900 m. Vouchers: Mwachala G et al. EW942, Faden RB, Evans A & Smeenk C 71/115, Faden RB & Faden AJ 74/280, Gilbert MG & Gilbert CI 6099, Napier ER 982 (EA).

***Hermanniaglanduligera* K.Schum.** – Habit: Herb. Habitat: *Acacia* bushland, woodland, road edges, old cultivation, 600–915 m. Vouchers: Hildebrandt JM 2385, Verdcourt B 5313 (EA).

***Hermanniaoliveri* K.Schum.** – Habit: Subshrub. Habitat: Dry *Acacia* or *Commiphora* bushland, 500–650 m. Vouchers: Greenway PJ & Kanuri K 10463, MacDonald J 868 (EA).

***Hermanniauhligii* Engl.** – Habit: Shrub. Habitat: *Acacia*-*Commiphora* bushland or grassland, 600–950 m. Voucher: Luke WRQ & Luke PA 4249 (EA).

***Hibiscuscalyphyllus* Cav.** – Habit: Shrub. Habitat: Forest edges, riverine forest, bushland, 650–1048 m. Vouchers: Watuma BM W0165 (EA, HIB), Kabuye CHS et al. NMK Exped. 738, Medley KE 740 (EA).

***Hibiscuscannabinus* L.** – Habit: Herb. Habitat: Forest margin, *Acacia* woodland and scrub, wooded grassland, 1795–1800 m. Voucher: Christenhusz MJM, Kamau P, Chase MW, Mbale M & Kyaa J et al. 6676 (EA).

***Hibiscusdiversifolius* Jacq.** – Habit: Shrub. Habitat: Edges of gallery forest, riverbanks, drainage paths in forests, 650–1000 m. Vouchers: Medley KE 597 & 806 (EA).

***Hibiscusfuscus* Garcke** – Habit: Herb. Habitat: Forest, grassland, bushland, 1250–1582 m. Vouchers: Watuma BM W0248 & W0256 (EA, HIB), Mwachala G et al. EW1250 (EA).

***Hibiscusgreenwayi* Bakerf.** – Habit: Shrub. Habitat: *Acacia*-*Commiphora* bushland, 600–1370 m. Vouchers: Mwachala G et al. EW191, Tengecho 2, Joanna B 8894 (EA). Vulnerable.

***Hibiscuskabuyeanus* Mwachala** – Habit: Herb. Habitat: Edges of dry woodland, 500–1000 m. Vouchers: Mwachala G et al. EW192, Medley KE 816, Greenway PJ & Kanuri K 12683 (EA).

*Hibiscuslunariifolius Willd. – Habit: Herb. Habitat: Seasonally flooded woodland and disturbed habitats, 850–1050 m. Voucher: Mwachala G et al. EW933 (EA).

***Hibiscusmacranthus* Hochst. ex A.Rich.** – Habit: Subshrub. Habitat: Forest edges, cleared forest, bushland, 1030–1150 m. Vouchers: Watuma BM W0323 (EA, HIB), Mungai G et al. EW1394 (EA).

***Hibiscusmicranthus* L.f.** – Habit: Shrub. Habitat: *Acacia* bushland and grassland, 500–650 m. Vouchers: Hucks M 903, Medley KE p.r. (EA).

***Hibiscusovalifolius* (Forssk.) Vahl** – Habit: Shrub. Habitat: Forest, woodland, bushland, grassland, 800–1050 m. Vouchers: Mwachala G et al. EW936 (EA).

***Hibiscusphysaloides* Guill. & Perr.** – Habit: Herb. Habitat: Woodland, abandoned cultivations, 800–850 m. Voucher: Mwachala G et al. EW750 (EA).

***Hibiscussurattensis* L.** – Habit: Herb. Habitat: Bushland, grassland and forest, 600–1150 m. Vouchers: SAJIT–005350 (EA, HIB), Napier ER 991 (EA).

***Hibiscusvitifolius* L.** – Habit: Herb. Habitat: *Acacia*-*Commiphora* bushland, grassland, ca. 600–700 m. Vouchers: Gillett JB & Burtt BL 17173, Hildebrandt JM 2851 (EA).

***Kosteletzkyaadoensis* (Hochst. ex A.Rich.) Mast.** – Habit: Subshrub. Habitat: Montane forest edges, secondary growth and clearings, near streams, ca. 1030 m. Voucher: Mwachala G et al. EW800 (EA).

***Leptonychiausambarensis* K.Schum.** – Habit: Tree. Habitat: Moist forest, 1500–1925 m. Vouchers: Watuma BM W0059 & W0134 (EA, HIB), Wakanene KM & Mwangangi OM 675, Faden RB et al. NMK Exped. 236 & 459, Beentje HJ et al. NMK Exped. 868, von Someren & Beentje HJ et al. NMK Exped. 1050, Faden RB & Evans A 69/882, Raler I 0142 (EA).

*Malvaverticillata L. – Habit: Herb. Habitat: Forest, bushland, stream banks, cultivation and waste grounds, ca. 1800 m. Voucher: Drummond RB & Hemsley JH 4286 (EA).

***Melhaniarotundata* Hochst. ex Mast.** – Habit: Herb. Habitat: Open to dense *Acacia*-*Commiphora* woodland or bushland, ca. 650 m. Voucher: Faden RB & Faden AJ 74/456 (EA).

***Melhaniavelutina* Forssk.** – Habit: Herb. Habitat: *Acacia*, *Commiphora*, *Combretum*, *Terminalia* woodland, bushland, cultivated areas, 800–1180 m. Vouchers: Kabuye CHS et al. NMK Exped. 688, Mwachala G et al. EW121, 355, 1238 & 1687, Friis I & Hansen OJ 2647, Bally PRO 1304 (EA).

***Pavoniaburchellii* (DC.) R.A.Dyer** – Habit: Shrub. Habitat: Forest margins, woodland, rocky places and cultivations, 960–1050 m. Vouchers: Mwachala G et al. EW1348, Mungai GM et al. EW1364 (EA).

***Pavoniaurens* Cav.** – Habit: Subshrub. Habitat: Forest margins and clearings, 1250–2000 m. Vouchers: Mwachala G et al. EW1258, Faden RB et al. NMK Exped. 8 & 816, Beentje HJ et al. NMK Exped. 932 (EA).

***Sidaacuta* Burm.f.** – Habit: Subshrub. Habitat: Forest edges, bushland, grassland, 1059–1599 m. Vouchers: Watuma BM W0142 & W0155 (EA, HIB).

***Sidaovata* Forssk.** – Habit: Subshrub. Habitat: *Acacia*-*Commiphora* bushland and thicket, 550–650 m. Vouchers: Medley KE 971 & 1006b (EA).

***Sidarhombifolia* L.** – Habit: Subshrub. Habitat: Forest margins, 1700–1875 m. Voucher: Faden RB et al. NMK Exped. 520 (EA).

***Sidaschimperiana* Hochst. ex A.Rich.** – Habit: Shrub. Habitat: Forest edges, plantation forest, *Acacia* woodland, rocky places, 1600–1875 m. Vouchers: Faden RB et al. NMK Exped. 533, Beentje HJ et al. NMK Exped. 1157 (EA).

***Sparrmanniaricinocarpa* (Eckl. & Zeyh.) Kuntze** – Habit: Subshrub. Habitat: Forest patches, secondary bushland, 1900–2170 m. Vouchers: Faden RB et al. NMK Exped. 127, Christenhusz MJM, Kamau P, Chase MW, Mbale M & Kyaa J et al. 6657 (EA).

***Sterculiaafricana* (Lour.) Fiori** – Habit: Tree. Habitat: *Acacia*-*Combretum* dry bushland, 500–914 m. Vouchers: Mwachala G et al. EW1515, Mwachala G, Mungai G & Peris K 213, Muasya S 47, Battiscombe E 166 (EA).

***Sterculiarhynchocarpa* K.Schum.** – Habit: Tree. Habitat: *Acacia*-*Commiphora* woodland and bushland, 420–760 m. Vouchers: Kabuye CHS 82/96, Medley KE 434, Faden RB & Faden AJ 74/287, Napier ER 1053, Braun 1541 (EA).

***Sterculiastenocarpa* H.J.P.Winkl.** – Habit: Tree. Habitat: *Commiphora*-*Terminalia*-*Combretum*-*Acacia* dry bushland, 600–750 m. Vouchers: Faden RB & Faden AJ 74/259, Winkler 4047, Greenway PJ & Kanuri K 12676, Hucks M 448 (EA).

***Thespesiagarckeana* F.Hoffm.** – Habit: Tree. Habitat: Wooded grassland, bushland, roadside, 950–1080 m. Vouchers: Mwachala et al. EW1230 & 3179 (EA).

***Triumfettaannua* L.** – Habit: Subshrub. Habitat: Forest clearings and tracks, shade in woodland and bushland, 1587–1875 m. Vouchers: Watuma BM W0227 (EA, HIB), Faden RB et al. NMK Exped. 529 (EA).

***Triumfettarhomboidea* Jacq.** – Habit: Subshrub. Habitat: Rainforest clearings, weed of cultivation, ca. 1480 m. Voucher: Hemp A 5236 (EA).

***Waltheriaindica* L.** – Habit: Herb. Habitat: Weed of dry bushland and grassland, disturbed places, cultivation, 500–1580 m. Vouchers: Kabuye CHS 82/116, Mwachala G et al. EW1210, Mungai GM et al. EW1362, Wakanene KM & Mwangangi OM 292, Waita J 41, Jonsell BE 2044, Medley KE 985, Hildebrandt JM 2468, Napier ER 963, Hucks M 1133 (EA).

**F101. MELASTOMATACEAE**

3 Genera, 3 species

***Heterotisrotundifolia* (Sm.) Jacq.-Fél**. – Habit: Herb. Habitat: Margins of rainforest, riverine thickets, 620–920 m. Vouchers: Mwachala G et al. EW1552 & 1674, Stone RD, Gitau D & Mwadime P 2413, Muasya J, Nyamongo D & Sang’any S 02/2006/16, Bally 13572 (EA).

^**NE**^***Memecylongreenwayi* Brenan** – Habit: Tree. Habitat: Rainforest, 1040–1480 m. Vouchers: Luke WRQ & Luke PA 4160, Stone G, Gitau D & Mwadime P 2412, De Block P et al. 369, Medley KE 670 (EA).

**^E^*Memecylonteitense* Wickens** – Habit: Tree. Habitat: Upland rainforest, 1440–1950 m. Vouchers: Watuma BM W0290 (EA, HIB), Faden RB et al. NMK Exped. 303, Faden RB et al. 71/45, 984, 70/537 & 741, Drummond RB & Hemsley JH 4363, De Block P et al. 494, Stone RD et al. 2411 & 2414, Seki T et al. 1592, Raler I 0052 (EA). Vulnerable.

**F102. MELIACEAE**

6 Genera, 12 species

***Azadirachtaindica* A.Juss.** – Habit: Tree. Habitat: Cultivated in villages. Voucher: Ossent JR s.n. (EA).

***Ekebergiacapensis* Sparrm.** – Habit: Tree. Habitat: Understorey often at edges in montane and mid-altitude forests, 900–1875 m. Vouchers: Mwachala G et al. EW914, Faden RB et al. NMK Exped. 344, 460, 541 & 881, Faden RB et al. 72/207, Raler I 0047 (EA).

***Lepidotrichiliavolkensii* (Gürke) J.-F.Leroy** – Habit: Tree. Habitat: Montane and mid-altitude forest, 1425–1875 m. Vouchers: SAJIT–006388, Watuma BM W0088 & W0210 (EA, HIB), Faden RB et al. NMK Exped. 219 & 399, Beentje HJ et al. NMK Exped. 43, Raler I 0030 (EA).

*Meliaazedarach L. – Habit: Tree. Habitat: Secondary grasslands and thickets, 850–1100 m. Voucher: Kluguess LM 13 (EA).

***Meliavolkensii*** Gürke – Habit: Tree. Habitat: *Acacia*-*Commiphora* deciduous bushland, 550–1000 m. Voucher: Medley KEM p.r. (EA).

***Trichiliaemetica* Vahl** – Habit: Tree. Habitat: Riparian forest and woodland, 800–1400 m. Vouchers: Kabuye CHS et al. NMK Exped. 657, Mwachala G et al. EW519, 1345, 2555 & 2590, Medley KE 879, Hucks M 878 (EA).

***Turraeaholstii*** Gürke – Habit: Shrub/Tree. Habitat: Understorey and margins of montane and mid-altitude forest, 1000–1985 m. Vouchers: Watuma BM W0077 (EA, HIB), Wakanene KM & Mwangangi OM 643 & 732, De Block P et al. 297, Raler I 0044, Faden RB et al. NMK Exped. 220, Beentje HJ et al. NMK Exped. 849 & 1111, Faden RB & Evans A 69/880, Christenhusz MJM et al. 6632, Medley KE 661 & 936, Bally PRO 8784, Gardner HM 2992 (EA).

**TurraeamombassanaHiern ex C.DC.subsp.cuneata (Gürke) Styles & F.White** – Habit: Shrub. Habitat: Dry forest, bushland and thicket, rocky slopes, ca. 1640 m. Voucher: Kabuye CHS 82/45 (EA).

**TurraeamombassanaHiern ex C.DC.subsp.mombassana** – Habit: Shrub. Habitat: Evergreen forest undergrowth and margins, secondary forest, 650–1100 m. Vouchers: Mwachala G et al. EW3507, Medley KE 924 (EA).

***Turraeanilotica* Kotschy & Peyr.** – Habit: Shrub/Tree. Habitat: Woodland and bushland, 650–1500 m. Vouchers: Medley KE 558, Joanna B 8906, Sacleux C 2355 (EA).

***Turraeaparvifolia* Deflers** – Habit: Shrub. Habitat: *Acacia*-*Commiphora* bushland, rocky outcrops, ca. 1500 m. Voucher: Mwachala G et al. EW3052A (EA).

***Turraearobusta*** Gürke – Habit: Shrub/Tree. Habitat: Evergreen forest, edges of mid-altitude and riparian forest, 800–1875 m. Vouchers: SAJIT–004114, Watuma BM W0080 & W0204 (EA, HIB), Kabuye CHS 82/91, Mwachala G et al. EW525 & 935, Mungai GM et al. EW1298 & 1404, Napier ER 1093, Dale IR 2007 (EA).

***Turraeawakefieldii* Oliv.** – Habit: Shrub. Habitat: Secondary forest and bushland, ca. 600 m. Vouchers: Ossent JR 130, Wilson J 53 (EA).

**F103. MENISPERMACEAE**

5 Genera, 6 species

***Chasmantheradependens* Hochst.** – Habit: Liana. Habitat: Lowland rainforest, montane woodland, rocky outcrops and dry riverbeds, 650–1100 m. Vouchers: Mwachala G et al. EW209, Archer PG 623, Medley s.r. (EA).

***Cissampelosmucronata* A.Rich.** – Habit: Liana. Habitat: Deciduous bushland, riverine forest, cultivated lands, 600–1000 m. Vouchers: Mungai GM et al. EW1667, Medley KE 776 (EA).

***Cissampelospareira* L.** – Habit: Climber. Habitat: Upland and lowland rainforest, bushed grassland, 850–1875 m. Vouchers: Mwachala G et al EW 62, 911, 1199A & 1200 (EA), Faden RB et al. NMK Exped. 592 (EA).

***Hyalosepalumcaffrum* (Miers) Troupin** – Habit: Liana. Habitat: Upland rainforest, deciduous bushland, rocky outcrops, 823–862 m. Vouchers: SAJIT-004627 (EA, HIB), Polhill RM & Kibuwa SP 965 (EA).

***Tiliacorafunifera* (Miers) Oliv.** – Habit: Liana. Habitat: Upland rainforest, riverine forest, moist shady places in woodland, 1300–1875 m. Vouchers: De Block P, Muasya, Stieperaere H & Bytebier B 287, Raler I 0096, Faden RB et al. NMK Exped. 510, Medley KE 614 & 1017, Faden RB et al. 71/238 (EA).

***Triclisiasacleuxii* (Pierre) Diels** – Habit: Liana. Habitat: Lowland rainforest, riverine forest, 1050–1200 m. Voucher: Bally PRO 8575 (EA).

**F104. METTENIUSACEAE**

1 Genus, 1 species

***Apodytesdimidiata* E.Mey. ex Arn.** – Habit: Tree. Habitat: Evergreen forest, mostly disturbed, 650–1550 m. Vouchers: SAJIT–005348 (EA, HIB), Beentje HJ et al. NMK Exped. 840 & 1113, Medley KE 679 & 788 (EA).

**F105. MOLLUGINACEAE**

2 Genera, 2 species

***Hypertelisumbellata* (Forssk.) Thulin** – Habit: Herb. Habitat: Weed of roadsides and cultivated places, ca. 609 m. Voucher: Napier ER 902 (EA).

***Paramollugonudicaulis* (Lam.) Thulin** – Habit: Herb. Habitat: Weed of roadsides waste and cultivated places, ca. 609 m. Voucher: Napier ER 1020 (EA).

**F106. MONIMIACEAE**

1 Genus, 1 species

***Xymalosmonospora* (Harv.) Baill.** – Habit: Tree. Habitat: Upland rainforest, 1400–2170 m. Vouchers: SAJIT–004536 & 005313, Watuma BM W0103 (EA, HIB), Wakanene KM & Mwangangi OM 380 & 667, Faden RB et al. NMK Exped. 24, 105 & 372, Beentje HJ et al. NMK Exped. 873 & 936, Drummond RB & Hemsley JH 4380, Medley KE 622, Joanna B 9026, Dale IR 3784, Mutangah JG & Nakamura E JKCAT 1599, Gilbert MG 7108 (EA).

**F107. MORACEAE**

4 Genera, 22 species

***Dorsteniabrownii* Rendle** – Habit: Herb. Habitat: Evergreen forest, wet places, often among rocks, 1425–1850 m. Vouchers: SAJIT–005326 (EA, HIB), Faden RB et al. NMK Exped. 763, Faden RB et al. 70/224 & 552 (EA).

^**E**^***Dorsteniachristenhuszii* M.W.Chase & M.F.Fay** – Habit: Herb. Habitat: Upland rainforest near streams, 1425–1850 m. Vouchers: Christenhusz MJM et al. 6664, Malombe & Matheka 1233, Kamau P 457, Matheka K & Miyawa D 841, Faden RB et al. NMK Exped. 765, Koen TW s.n. (EA).

***Dorsteniagoetzei* Engl.** – Habit: Herb. Habitat: Wet and dry forests on hill slopes among rocks, 800–1200 m. Vouchers: Kabuye CHS et al. NMK Exped. 669, Luke WRQ & Luke PA 5517, Luke WRQ et al. 6414 (EA). Near Threatened.

**DorsteniahildebrandtiiEngl.var.hildebrandtii** – Habit: Herb. Habitat: Forest, woodland, bushland near streams, 670–1000 m. Vouchers: SAJIT–005379 (EA, HIB), Faden RB, Evans A & Msafiri F 70/955, Sacleux C 2288 (EA).

**DorsteniahildebrandtiiEngl.var.schlechteri (Engl.) Hijman** – Habit: Herb. Habitat: Moist forest, along streams into woodland and thicket, 670–1550 m. Vouchers: Faden RB et al. 70/181 & 483, Faden RB & Faden AJ 74/500, Archer PG 310 (EA).

**^NE^*Dorsteniawarneckei* Engl.** – Habit: Herb. Habitat: Forest, in shady, wet and rocky places, ca. 1500 m. Voucher: Wakanene KM & Mwangangi OM 678 (EA). Near Threatened.

***Dorsteniazanzibarica* Oliv.** – Habit: Herb. Habitat: Forest on ground humus and rocks, deciduous bushland, 700–1554 m. Vouchers: Luke WRQ, Bytebier B, Pakia M 5326, Wakanene KM & Mwangangi OM 678, Faden RB, Evans A, Githui M 70/152, Archer PG 311 & 492, Gilbert MG 5828 (EA).

***Ficusbubu* Warb.** – Habit: Tree. Habitat: Forest, montane woodland, riverine or ground water, disturbed places, ca. 905 m. Voucher: Medley KE 732 (EA).

***Ficusexasperata* Vahl** – Habit: Tree. Habitat: Forest edges, rocky slopes, along rivers, disturbed areas, 1260–1380 m. Vouchers: Kabuye CHS et al. NMK Exped. 805, Bally PRO 8773 (EA).

***Ficusglumosa* Delile** – Habit: Tree. Habitat: Rocky outcrops in wooded grasslands and deciduous bushlands, 650–1000 m. Vouchers: Medley KE 514, 555 & 813 (EA).

***Ficusingens* (Miq.) Miq.** – Habit: Tree. Habitat: Rock outcrops, stream sides, disturbed forests and wooded grasslands, 650–1000 m. Vouchers: Kabuye CHS 82/138, Kabuye et al. NMK Exped. 672, Mwachala G et al. EW924 & 3181, Medley KE 686 & 814 (EA).

***Ficuslutea* Vahl** – Habit: Tree. Habitat: Forest, montane woodland, riverine, cleared lands, 650–1000 m. Voucher: Medley KE 833 (EA).

***Ficusnatalensis* Hochst.** – Habit: Tree. Habitat: Wet and dry forest, riverine forest, high rainfall woodland, 900–1500 m. Vouchers: Luke WRQ & Luke PA 4097, Wakanene KM & Mwangangi OM 348 (EA).

***Ficuspopulifolia* Vahl** – Habit: Tree. Habitat: Riparian, rocky outcrops in deciduous bushland and wooded grassland, ca. 600–700 m. Vouchers: Polhill RM & Kibuwa SP 938, Hildebrandt JM 2842 (EA).

**Ficus sp. nr. recurvata De Wild.** – Habit: Tree. Habitat: Lowland forest, riverine and flood plains, 550–700 m. Voucher: Greenway PJ & Kanuri K 12703 (EA).

***Ficussalicifolia* Vahl** – Habit: Tree. Habitat: Seasonal streams, rocky outcrops, 500–950 m. Vouchers: Kabuye CHS et al. NMK Exped. 606, Ossent JR 119 (EA).

***Ficussur* Forssk.** – Habit: Tree. Habitat: Forest, riverine, wooded grassland, cleared places, 1000–1850 m. Vouchers: Watuma BM W0126 (EA, HIB), Kabuye CHS 82/139, Wakanene KM & Mwangangi OM 640, Faden RB et al. 71/217, Beentje HJ et al. NMK Exped. 1176, Raler I 0029, Medley KE 443, 628, 832 & 1008 (EA).

***Ficussycomorus* L.** – Habit: Tree. Habitat: Forest edges, riverine, montane woodland, 650–1000 m. Voucher: Medley KEM p.r. (EA).

***Ficusthonningii* Blume** – Habit: Tree. Habitat: Forest, woodland, bushland, wooded grassland, 700–1875 m. Vouchers: Watuma BM W0120 (EA, HIB), Faden RB et al. NMK Exped. 15 & 337, Medley KE 885, Raler I 0054 (EA).

**FicustremulaWarb.subsp.acuta (De Wild.) C.C.Berg** – Habit: Liana/Tree. Habitat: Upland rainforest, montane woodland, 650–1000 m. Vouchers: Medley KE 672 & 812 (EA).

***Ficuswakefieldii* Hutch.** – Habit: Tree. Habitat: Riverine, grassland, rocky outcrops, ca. 1300 m. Voucher: Luke WRQ & Luke PA 5531 (EA).

***Hijmaniaalta* (Engl.) M.D.M.Vianna** – Habit: Shrub. Habitat: Understorey in evergreen forest near streams, ca. 1180 m. Voucher: Mwachala G et al. EW282 (EA).

***Miliciaexcelsa* (Welw.) C.C.Berg** – Habit: Tree. Habitat: Rainforest, riverine and ground water forest, ca. 600 m. Vouchers: Mrs. Robertson SA 4693, Fleuret A 5 (EA). Near Threatened.

**F108. MORINGACEAE**

1 Genus, 1 species

***Moringaborziana* Mattei** – Habit: Subshrub. Habitat: Deciduous bushland on sandy soils, ca. 600 m. Voucher: Mrs. Robertson SA 7395 (EA).

**F109. MYRICACEAE**

1 Genus, 2 species

***Myricakilimandscharica* Engl.** – Habit: Shrub/Tree. Habitat: Upland rainforest, mist forest, 998–2170 m. Vouchers: Mwachala G et al EW98 & 1260, Wakanene KM & Mwangangi OM 342 & 438, Faden RB et al. NMK Exped. 79 & 302, Gardner HM 2947, Bally PRO 8719 (EA).

***Myricasalicifolia* Hochst. ex A.Rich.** – Habit: Shrub/Tree. Habitat: Upland rainforest, evergreen bushland, secondary thicket, 1400–2195 m. Vouchers: Rukunga G et al. 022/04, Medley KE 931, Christenhusz MJM et al. 6649 (EA).

**F110. MYRTACEAE**

4 Genera, 10 species

*Eucalyptuscamaldulensis Dehnh. – Habit: Tree. Habitat: Evergreen forest margins, ca. 1799 m. Voucher: Watuma BM W0287 (EA, HIB). Near Threatened.

***Eugeniauniflora* L.** – Habit: Tree. Habitat: Upland evergreen forest slopes, ca. 1500 m. Voucher: SAJIT–004108 (EA, HIB).

***Eugeniaverdcourtii* Byng** – Habit: Shrub/Tree. Habitat: Dry evergreen forest and bushland, 900–1200 m. Vouchers: Luke WRQ et al. 5332, Faden RB, Evans A & Githui M 70/179 (EA).

*Psidiumcattleyanum Sabine – Habit: Tree. Habitat: Mist forest edge, near homesteads, 1564–1632 m. Vouchers: SAJIT–004593, Watuma BM W0215 (EA, HIB).

*Psidiumguajava L. – Habit: Tree. Habitat: Mist forest edge, near homesteads, ca. 1577 m. Voucher: Watuma BM W0225 (EA, HIB).

**^NE^*Syzygiummicklethwaitii* Verdc.** – Habit: Tree. Habitat: Mist forest, dry evergreen forest, rocky slopes, 1000–1900 m. Vouchers: Watuma BM W0141 (EA, HIB), Luke WRQ & Luke PA 4152, Kabuye CHS 82/126, Faden RB et al. NMK Exped. 335, Drummond RB & Hemsley JH 4360, & 1002, Bally PRO 8711, Medley KE 922 & 1014 (EA). Vulnerable.

^**NE**^***Syzygiumsubcordatum* (Verdc.) Byng & N.Snow** – Habit: Tree. Habitat: Upland rainforest, 1400–1910 m. Vouchers: Mwachala G, Nyaboke B & Matheka K 1048, Faden RB, Evans A, Msafiri F & Smeenk C 71/17, Faden RB et al. NMK Exped. 386, Raler I 0053 (EA). Vulnerable.

***Syzygiumafromontanum* (F.White) Byng** – Habit: Tree. Habitat: Evergreen forest slopes, 1500–1600 m. Voucher: Dale IR 13529 (EA).

***Syzygiumguineense* (Willd.) DC.** – Habit: Tree. Habitat: Forest, riverine forest, *Acacia*-*Combretum*-*Terminalia* woodland, 950–1875 m. Vouchers: Mwachala G et al. EW2805, Medley KE 441, Faden RB et al. NMK Exped. 187, Raler I 0050 (EA).

*Syzygiumjambos (L.) Alston – Habit: Tree. Habitat: Cultivated, 1300–1500 m. Voucher: Kimuzi D 1 (EA).

**F111. NYCTAGINACEAE**

2 Genera, 7 species

***Boerhaviacoccinea* Mill.** – Habit: Herb. Habitat: Disturbed rocky ground in open bushland, waste places, roadsides, cultivated areas, 560–800 m. Vouchers: Wakanene KM & Mwangangi OM 707, Kuchar P, Msafiri F & Karime N 5689, Hucks M 709 (EA).

***Boerhaviadiffusa* L.** – Habit: Herb. Habitat: Disturbed ground in cultivation, waste places, grassland, along paths, ca. 750 m. Voucher: Goyder DJ, Masinde PS & Whitehouse C 4017 (EA).

***Boerhaviaerecta* L.** – Habit: Herb. Habitat: Roadsides in steep grassy slopes, alluvial soils, 556–750 m. Vouchers: Faden RB & Faden AJ 74/264, Goyder DJ, Masinde PS, Whitehouse C 4013, Bally B 13248, Ciba-Geigy 6, Hucks M 765, Napier ER 909, Belsky JB 367, Friis IB 98 (EA).

***Commicarpusgrandiflorus* (A.Rich.) Standl.** – Habit: Herb. Habitat: Grassland, open woodland and scrub, ca. 950 m. Vouchers: Luke WRQ & Luke PA 6446 (EA).

***Commicarpushelenae* (Roem. & Schult.) Meikle** – Habit: Herb. Habitat: *Commiphora*-*Sansevieria* bushland and bushland thicket, ca. 600 m. Vouchers: Faden RB & Faden AJ 74/531, Hucks M 766, Belsky JB 637 (EA).

***Commicarpuspedunculosus* (A.Rich.) Cufod.** – Habit: Herb. Habitat: Riverine in dry grassland, open bushland, scrub and rocky hillsides, ca. 800 m. Voucher: Wakanene KM & Mwangangi OM 712 (EA).

***Commicarpusplumbagineus* (Cav.) Standl.** – Habit: Herb. Habitat: Dry bush, scrub and riverine forest, 579–860 m. Vouchers: Watuma BM W0168 (EA, HIB), Mwachala G et al. EW938, 938A & 979, Goyder DJ, Masinde PS & Whitehouse C 4018, Gardner HM 3002, Napier ER 1051 (EA).

**F112. NYMPHAEACEAE**

1 Genus, 1 species

**NymphaeanouchaliBurm.f.var.caerulea (Savigny) Verdc.** – Habit: Herb. Habitat: Swamps, seasonal ponds, ca. 1617 m. Voucher: Watuma BM W0302 (EA, HIB). Endangered.

**F113. OCHNACEAE**

2 Genera, 7 species

***Campylospermumsacleuxii* (Tiegh.) Farron** – Habit: Tree. Habitat: Evergreen forest, ground-water, semi-dry and riverine forest, ca. 1750 m. Voucher: Wakanene KM & Mwangangi OM 638 (EA).

**^NE^*Campylospermumscheffleri* (Engl. & Gilg) Farron** – Habit: Tree. Habitat: Evergreen forest, *Ocotea*-*Podocarpus* mist forest, 1425–1875 m. Vouchers: Watuma BM W0023 (EA, HIB), Luke WRQ et al. 5364, Christenhusz MJM et al. 6673, Faden RB et al. NMK Exped. 407, 571 & 758, Beentje HJ et al. NMK Exped. 952, Drummond RB & Hemsley JH 4342, Faden RB et al. 71/0183, Raler I 0105 (EA). Vulnerable.

***Ochnaholstii* Engl.** – Habit: Tree. Habitat: Rainforest, moist and dry upland evergreen forest, 1000–1875 m. Vouchers: Watuma BM W0279 (EA, HIB), Mwachala G et al. EW138, 181, 188 & 246, Wakanene KM & Mwangangi OM 461, Faden RB et al. NMK Exped. 250, Raler I 0020, Faden RB, Evans A & Smeenk C 71/151, Medley KE 554 & 881 (EA).

***Ochnainermis* (Forssk.) Schweinf.** – Habit: Shrub. Habitat: Mixed bushland and scrub in rocky places, 500–750 m. Vouchers: Faden RB, Evans A, Rathbun B 69/422, Drummond, RB & Hemsley JH 4270, Ossent JR 140, Jex-Blake M 5705, Hucks M 462 (EA).

***Ochnainsculpta* Sleumer** – Habit: Shrub. Habitat: Evergreen forest, riverine forest, woodland at forest edge. Voucher: Joanna B 8903 (EA).

***Ochnaatropurpurea* DC.** – Habit: Shrub. Habitat: Mixed thicket and bushland, Vouchers: Luke WRQ & Festo L 2429, Fleuret A 16, Safary M 66 (EA).

***Ochnaovata* F.Hoffm.** – Habit: Shrub. Habitat: Dry evergreen forest, *Acacia*-*Commiphora* woodland and bushland, 550–1400 m. Vouchers: Mwachala G et al. EW1278, Mungai GM et al. EW3285, Luke WRQ & Luke PA 5361, Drummond RB & Hemsley JH 4397 & 4411, Medley KE 499 (EA).

**F114. OLACACEAE**

1 Genus, 1 species

***Strombosiascheffleri* Engl.** – Habit: Tree. Habitat: Rainforest, 1000–1900 m. Vouchers: Drummond RB & Hemsley JH 4354, Raler I 0009, Medley KE 904, De Block P, Muasya, Stieperaere H & Bytebier B 315, Faden RB et al. NMK Exped. 233 (EA).

**F115. OLEACEAE**

4 Genera, 5 species

**JasminumfluminenseVell.subsp.fluminense** – Habit: Shrub. Habitat: Forest, woodland, bushland and grassland, 550–1613 m. Vouchers: Watuma BM W0268 (EA, HIB), Kabuye CHS et al. NMK Exped. 741, Mwachala G et al. EW2803, Mungai GM et al. EW1655 & 2827, Drummond RB & Hemsley JH 4391, Medley KE 454 & 878, Hildebrandt HJ 2500 (EA).

***Jasminumstreptopus* E.Mey.** – Habit: Liana. Habitat: Forest margins, moist bushland and thicket, ca. 985 m. Voucher: Faden RB & Faden AJ 74/485 (EA).

***Noronhiamildbraedii* (Gilg & G.Schellenb.) Hong-Wa & Besnard** – Habit: Tree. Habitat: Evergreen moist forest on basement complex, 1425–1850 m. Voucher: Faden RB et al. NMK Exped. 382 (EA).

**OleaeuropaeaL.subsp.cuspidata (Wall. ex G.Don) Cif.** – Habit: Tree. Habitat: Evergreen woodland, upland rainforest, wooded grasslands, 650–1640 m. Vouchers: Kabuye CHS 82/44, Medley KE 617, 631 & 638 (EA).

***Schreberaalata* (Hochst.) Welw.** – Habit: Tree. Habitat: Bushland and forest, 1000–1875 m. Vouchers: Mwachala G et al. EW3083/A, Faden RB et al. NMK Exped. 548, Hildebrandt JM 2532, Raler I 0121 (EA).

**F116. ONAGRACEAE**

2 Genera, 4 species

***Epilobiumhirsutum* L.** – Habit: Herb. Habitat: Swampy and marshy areas by streams and rivers, ca. 1550 m. Voucher: Kabuye CHS 82/148 (EA).

***Ludwigiaabyssinica* A.Rich.** – Habit: Shrub. Habitat: Swampy ground by ditches and rivers, 1000–1372 m. Vouchers: Mwachala G et al. EW3553, Kabuye CHS 82/43 & 128, Luke WRQ et al. 5299, Mungai G et al. EW2813, Wakanene KM & Mwangangi OM 539 & 617, Faden RB, Evans A, Smeenk C 70/574 (EA).

***Ludwigiajussiaeoides* Desr.** – Habit: Shrub. Habitat: Swampy and seasonally flooded places, streams, 823–1372 m. Vouchers: Kabuye CHS 82/29, Archer PG 559 (EA).

***Ludwigialeptocarpa* (Nutt.) H.Hara** – Habit: Herb. Habitat: Swamp, ditches, along rivers, ca. 850 m. Vouchers: Mwachala G et al. EW760, Lubai lK HB10 (EA).

**F117. OPILIACEAE**

1 Genus, 1 species

***Opiliaamentacea* Roxb.** – Habit: Liana. Habitat: *Acacia* woodland, along temporary streams, upland rainforest, 550–1875 m. Vouchers: Watuma BM W0175 (EA, HIB), Mwachala G et al. EW216, Medley KE 498, 567, 577, 608, 873, 890 & 981, Faden RB et al. NMK Exped. 559 (EA).

**F118. OROBANCHACEAE**

7 Genera, 13 species

***Alectrasessiliflora* (Vahl) Kuntze** – Habit: Herb. Habitat: Forest undergrowth, path sides and edges, montane grassland, 1400–2000 m. Vouchers: SAJIT–004570 & 005409 (EA, HIB), Drummond RB & Hemsley JH 4346, Beentje HJ et al. NMK Exped. 356, Faden RB et al. NMK Exped. 1003 (EA).

***Alectravogelii* Benth.** – Habit: Herb. Habitat: Cultivations, riverbanks and grasslands, 457–609 m. Voucher: Nattrass MS 796 (EA).

***Asepalumeriantherum* (Vatke) Marais** – Habit: Shrub. Habitat: *Commiphora* woodland, deciduous bushland, 490–609 m. Vouchers: Muasya J GBK 004/025/2001, Dale IR 3647, Faden RB & Faden AJ 74/522, Hucks M 480, Ossent JR 137 (EA).

***Buttonianatalensis* McKen ex Benth.** – Habit: Liana. Habitat: Dry rocky places in woodland and riverbanks, 579–690 m. Vouchers: Gillett JB 18740, Archer PG 449, Greenway PJ 9829 (EA).

***Cycniumherzfeldianum* (Vatke) Engl.** – Habit: Herb. Habitat: Forest margins, wet grassland, 1000–1780 m. Vouchers: SAJIT–004107 (EA, HIB), Kabuye CHS 82/5, Mwachala G et al. EW625, 1193, 1356, 2814, 3075A, 3139, Wakanene KM & Mwangangi OM 749, Cheseny CMC 34/72, Friis I & Hansen OJ 2643, Ossent JR 91, Nappier ER 1101 (EA).

***Cycniumveronicifolium* (Vatke) Engl.** – Habit: Herb. Habitat: Open hillsides, degraded forests, grassland, wooded grassland, 1740–2100 m. Vouchers: SAJIT–004552 (EA, HIB), Gillett JB, Burtt BL, Osborn RM 17085, Bally PRO 13561, Beentje HJ et al. NMK Exped. 905, Uhlig C 5 (EA).

^**NE**^***Harveyakenyensis* Hepper** – Habit: Herb. Habitat: Thickets and forests, shaded ground, 500–900 m. Vouchers: Muasya AM 2097 (EA), Leach LC & Bayliss B 10250 (EA).

***Harveyaobtusifolia* (Benth.) Vatke** – Habit: Herb. Habitat: Grass in thickets and bushland, ca. 950 m. Voucher: Mwachala G et al. EW1272 (EA).

***Pseudosopubiahildebrandtii* (Vatke) Engl.** – Habit: Herb. Habitat: River margins, grassland with trees, ca. 620 m. Voucher: Gillett JB & Burtt BL 17180 (EA).

***Strigaasiatica* (L.) Kuntze** – Habit: Herb. Habitat: Woodland, plantation forest, cultivated lands, 1000–1600 m. Vouchers: Mwachala G et al. EW603, 995 & 1568, Mungai GM et al. EW3195, Beentje HJ et al. NMK Exped. 1140 (EA).

***Strigaforbesii* Benth.** – Habit: Herb. Habitat: Grassy areas with cracking clay soil, 1150–1600 m. Vouchers: Mwachala G et al. EW605, Beentje HJ et al. NMK Exped. 1137 (EA).

***Strigagesnerioides* (Willd.) Vatke.** – Habit: Herb. Habitat: Grassland and cultivated grounds, 610–1050 m. Vouchers: SAJIT–005385 (EA, HIB), Polhill RM & Kibuwa SP 950, Joana B 8947, Gillett JB 17238 & 18741 (EA).

***Strigalatericea* Vatke** – Habit: Herb. Habitat: Seasonally wet grassland, cultivated lands, 600–900 m. Voucher: Joanna B & Opiko 8928 (EA).

**F119. OXALIDACEAE**

1 Genus, 2 species

***Oxaliscorniculata* L.** – Habit: Herb. Habitat: Weed in cultivation and disturbed areas, roadsides, 920–1000 m. Vouchers: Mwachala G et al. EW1351 & 1690 (EA).

***Oxalisdebilis* Kunth** – Habit: Herb. Habitat: Shaded cultivated places and waste places. Voucher: Kluguess LM 15 (EA).

**F120. PAPAVERACEAE**

1 Genus, 1 species

***Fumariaabyssinica* Hammar** – Habit: Herb. Habitat: Glades in upland rainforest, weed of cultivations, ca. 1814 m. Voucher: SAJIT–006396 (EA, HIB).

**F121. PASSIFLORACEAE**

3 Genera, 9 species

***Adeniaglobosa* Engl.** – Habit: Liana. Habitat: Dry evergreen bushland, montane woodland, scrubland, 650–1000 m. Vouchers: Watuma BM W0186 (EA, HIB), Bally PRO 13406, Medley s.r. (EA).

***Adeniacissampeloides* (Planch. ex Hook.) Harms** – Habit: Climber. Habitat: Forest, bushland, ca. 950–998 m. Vouchers: Mwachala G et al. EW49 & 845 (EA).

***Adeniakeramanthus* Harms** – Habit: Subshrub. Habitat: Deciduous woodland & bushland, 650–1000 m. Vouchers: Mwachala et al. EW3308, Ossent JR 98, Bally JB 13409, Medley KE 573 (EA).

**AdenialanceolataEngl.subsp.scheffleri (Engl. & Harms) W.J. de Wilde** – Habit: Climber. Habitat: Deciduous bushland, 600–800 m. Voucher: Joanna B 9083 (EA).

***Adeniavolkensii* Harms** – Habit: Herb. Habitat: Grassland, deciduous bushland, ca. 1150 m. Voucher: Mwachala G et al. EW3189 (EA).

**Adeniawightiana(Wall. ex Wight & Arn.)Engl.subsp.africana W.J. de Wilde** – Habit: Herb. Habitat: Forest edges, deciduous bushland, 800–950 m. Vouchers: Kabuye CHS et al. NMK Exped. 727, Archer PG 14782 (EA).

***Basananthehanningtoniana* (Mast.) W.J. de Wilde** – Habit: Climber. Habitat: Open areas in forest and margins, deciduous bushland, 862–1801 m. Vouchers: SAJIT–004624, Watuma BM W0046 (EA, HIB), Luke PA & Luke WRQ 6458, Kabuye CHS et al. NMK Exped. 809, Faden RB & Faden AJ 74/455 (EA).

***Basananthesubsessilicarpa* J.B. Gillett ex Verdc.** – Habit: Climber. Habitat: Open bushland, 950–1400 m. Vouchers: Mwachala G et al. EW961, Wakanene KM & Mwangangi OM 658, Bally 13291 (EA).

*Passifloraedulis Sims – Habit: Climber. Habitat: Escaped in forest edges, thickets and disturbed places, 1500–1875 m. Voucher: Faden RB et al NMK Exped 479 (EA).

**F122. PEDALIACEAE**

1 Genus, 1 species

***Sesamothamnusrivae* Engl.** – Habit: Shrub/Tree. Habitat: *Acacia*-*Commiphora* scrub, 550–1000 m. Vouchers: Medley KE 714 & 874, Dale IR 3877, Faden RB & Faden AJ 74/256 (EA).

**F123. PENAEACEAE**

1 Genus, 1 species

***Oliniarochetiana* A.Juss.** – Habit: Shrub/Tree. Habitat: Disturbed upland dry and moist evergreen forest, forest edges, 1000–1640 m. Vouchers: Muasya J GBK 48007, Mwachala G s.n., Medley KE s.n. (EA).

**F124. PERACEAE**

1 Genus, 1 species

**ClutiaabyssinicaJaub. & Spachvar.usambarica Pax & K.Hoffm.** – Habit: Shrub. Habitat: Open areas in evergreen forest, secondary associations, 1700–1950 m. Vouchers: SAJIT–004535, Watuma BM W0068 (EA, HIB), Faden RB et al. NMK Exped. 318, Bally 8798, Mwachala G et al. EW1676, Christenhusz MJM, Kamau P, Mbale M, Chase MW & Kyaa J 6656, Medley KE 589, 625, 702 & 799 (EA).

**F125. PHYLLANTHACEAE**

6 Genera, 11 species

***Antidesmavenosum* E.Mey. ex Tul.** – Habit: Tree. Habitat: Forest edges, open forest, deciduous woodland, ca. 1050 m. Voucher: Mungai GM et al. EW1750 (EA).

***Brideliacathartica* Bertol.** – Habit: Shrub. Habitat: Woodland, bushland, thicket, ca. 990 m. Voucher: Mwachala G et al. EW1570 (EA).

***Brideliamicrantha* (Hochst.) Baill.** – Habit: Tree. Habitat: Edges of evergreen forest, bushland and thicket, 650–1206 m. Vouchers: Watuma BM W0014 (EA, HIB), Mwachala G et al. EW228, Kabuye CHS et al. NMK Exped. 668, Medley KE 692 & 829 (EA).

***Brideliataitensis* Vatke & Pax** – Habit: Shrub/Tree. Habitat: Upland and dry evergreen bushland, forest edges, deciduous bushland, 426–1200 m. Vouchers: SAJIT–005361 & 005391 (EA, HIB), Mwachala G et al. EW527 & 1511, Kabuye CHS et al. NMK Exped. 647, Wakanene KM & Mwangangi OM 520, Kitaka W 35, Medley KE 418 & 582, Faden RB & Faden AJ 74/254, Greenway PJ & Kanuri K 12892, Hildebrandt JM 2415 (EA).

**Flueggeavirosa(Roxb. ex Willd.)Voigtsubsp.virosa** – Habit: Shrub. Habitat: Forest edges and associated bushland, 550–650 m. Vouchers: Medley KE 532 (EA).

**Margaritariadiscoidea(Baill.)G.L.Webstervar.discoidea** – Habit: Tree. Habitat: Upland moist forest, deciduous woodland, fringing forest, 1700–1875 m. Voucher: Faden RB et al. NMK Exped. 486 (EA).

**^E^*Meineckiaovata* (E.A.Bruce) J.F.Brunel** – Habit: Shrub. Habitat: Undergrowth in evergreen montane mist forest, 1372–1900 m. Vouchers: Watuma BM W0288 (EA, HIB), Kabuye CHS 82/74, Faden RB et al. NMK Exped. 524, Faden RB & Faden AJ et al. 71/204, De Block P et al. 288 & 290, Gardner HM 2909, Raler I 0068 (EA). Vulnerable.

***Phyllanthusleucanthus* Pax** – Habit: Herb. Habitat: Forest edges, deciduous woodland and bushland, rocky and ruderal sites, ca. 500–700 m. Voucher: Leuthhold W 57 (EA).

***Phyllanthusmaderaspatensis* L.** – Habit: Herb. Habitat: Deciduous woodland, bushland, edges of wet areas, 1040–1100 m. Voucher: Mwachala G et al. EW234 (EA).

**^NE^*Phyllanthusmittenianus* Hutch.** – Habit: Shrub. Habitat: Evergreen forest, often in rocky places, riverine, 650–1480 m. Vouchers: Kabuye CHS et al. NMK Exped. 676, Mwachala G et al. EW1681 & 2561, Kamau P et al. EW3231, Luke WRQ et al. 5366, Mungai GM et al. EW1337, Gilbert MG & Gilbert CI 6101, Napier ER 1092 (EA).

***Phyllanthussepialis*** Müll.Arg. – Habit: Shrub. Habitat: Forest margins and pathways, upland bushland, along rivers in drier areas, 800–1573 m. Vouchers: Watuma BM W0211 (EA, HIB), Kabuye CHS et al. NMK Exped. 734, Mbale M, Muthoka P, Chesire C & Hay F NMK 957 (EA).

**F126. PHYTOLACCACEAE**

1 Genus, 1 species

***Phytolaccadodecandra* L’Herit.** – Habit: Shrub. Habitat: Forest, woodland, bushland and thicket, 1700–1875 m. Vouchers: Faden RB et al. NMK Exped. 343, Bally PRO 8760 (EA).

**F127. PIPERACEAE**

2 Genera, 8 species

***Peperomiaabyssinica* Miq.** – Habit: Herb. Habitat: Intermediate and montane forests, rocky places, 1525–2205 m. Vouchers: SAJIT–006391 (EA, HIB), Wakanene KM, Mwangangi OM & Dunn B 369, Christenhusz MJM et al. 6667, Faden RB et al. NMK Exped. 82 & 226, Faden RB & Faden AJ 72/253, Faden RB & Githui M 70/710 (EA).

***Peperomiablanda* (Jacq.) Kunth** – Habit: Herb. Habitat: Evergreen scrub, dry forest, bare and shady rocky places, 1050–1750 m. Vouchers: Wakanene KM & Mwangangi OM 584, Mungai GM et al. EW1753 (EA).

***Peperomiabangroana* C.DC.** – Habit: Herb. Habitat: Mossy tree trunks in upland forest and rainforest, 1400–1750 m. Vouchers: Bytebier B 1739, Faden RB & Faden AJ 72/264 (EA).

***Peperomialeptostachya* Hook. & Arn.** – Habit: Herb. Habitat: Mist forest, moist thicket, cliff faces, 800–985 m Vouchers: Kabuye CHS et al. NMK Exped. 655, Faden RB, Evans A & Githui M 70/160, Faden RB & Faden AJ 74/498 (EA).

***Peperomiaretusa* (L.f.) A.Dietr.** – Habit: Herb. Habitat: Mossy banks in mist and rainforest, rocky places in streams, 1000–1875 m. Vouchers: Luke WRQ & Luke PA 4201, Wakanene KM & Mwangangi OM 257, Christenhusz MJM et al. 6622, Faden RB et al. NMK Exped. 218, Faden RB et al. 71/169, Gillett JB, Burt BL & Osborn RM 17149 (EA).

***Peperomiatetraphylla* (G.Forst.) Hook. & Arn.** – Habit: Herb. Habitat: Mist forests, drier evergreen forests, semi-evergreen bushland, 1250–1875 m. Vouchers: Kabuye CHS 82/47, Faden RB et al. NMK Exped. 246, Faden RB et al. 70/711 & 71/139 (EA).

***Pipercapense* L.f.** – Habit: Shrub. Habitat: Moist forest undergrowth, 1150–2170 m. Vouchers: SAJIT–005332, Watuma BM W0055 (EA, HIB), Mwachala G et al. EW108, Wakanene KM & Mwangangi OM 221, 368 & 467, Muchele WN HB25/94, Drummond RB & Hemsley JH 4377, Faden RB et al. NMK Exped. 77 & 215, Raler I 0032, Christenhusz MJM et al. 6629, Medley KE 439, Faden RB et al. 71/985, Bally PRO 8764 & 13607, Murray High School 31 (EA).

***Piperumbellatum* L.** – Habit: Shrub. Habitat: Evergreen forest undergrowth, riverbanks, 1350–1800 m. Vouchers: Luke WRQ & Luke PA 4140, Christenhusz MJM, Chase MW, Kamau P Mbale M & Kyaa J 6663 (EA).

**F128. PITTOSPORACEAE**

1 Genus, 1 species

***Pittosporumviridiflorum* Sims** – Habit: Shrub/Tree. Habitat: Upland rainforest, riverine forest, 900–1370 m. Vouchers: Mwachala G et al. EW249, 577, 788 & 915, Kabuye CHS 82/10, Wakanene KM & Mwangangi OM 433, Gardner HM 2929 (EA).

**F129. PLANTAGINACEAE**

2 Genera, 3 species

***Misopatesorontium* (L.) Raf.** – Habit: Herb. Habitat: Weed of cultivations, sandy soils, open grassy places, roadsides, 2000–2100 m. Voucher: Drummond RB & Hemsley JH 4296 (EA).

***Veronicaabyssinica* Fresen.** – Habit: Herb. Habitat: Montane forest, stream banks, 1560–2170 m. Vouchers: SAJIT–006406 (EA, HIB), Faden RB et al. NMK Exped. 109, Gillett JB, Burtt BL & Osborn RM 17071 (EA).

***Veronicajavanica* Blume** – Habit: Herb. Habitat: Damp grasses, ditches, cultivations, near homesteads, 1000–1100 m. Voucher: Drummond RB & Hemsley JH 4400 (EA).

**F130. PLUMBAGINACEAE**

1 Genus, 1 species

***Plumbagozeylanica* L.** – Habit: Subshrub. Habitat: Deciduous woodland, grassland and scrub, often by rivers, 800–1000 m. Vouchers: Kabuye CHS et al. NMK Exped. 607, Mwachala G et al. EW929, Drummond RB & Hemsley JH 4406 (EA).

**F131. POLYGALACEAE**

1 Genus, 8 species

***Polygalaamboniensis*** Gürke – Habit: Herb. Habitat: Bushland, secondary grassland, cultivated grounds, 575–625 m. Vouchers: Hucks M 798, Gillett JB & Burtt BL 17186 (EA).

***Polygalaehlersii*** Gürke – Habit: Herb. Habitat: Forest clearings and forest margins, dry open places, ca. 2100 m. Voucher: Gillett JB, Burtt BL & Osborn RM 17081 (EA).

***Polygalaerioptera* DC.** – Habit: Herb. Habitat: Open bushland, woodland and grassland, rocky ground, 950–1000 m. Voucher: Mwachala G et al. EW320 (EA).

***Polygalakilimandjarica* Chodat** – Habit: Herb. Habitat: *Acacia*-*Commiphora* bushland, wooded and bushed grassland, ca. 610 m. Vouchers: Napier ER 2272, Johnstone 1884 (EA).

***Polygalamuratii* Jacq.-Fél.** – Habit: Herb. Habitat: Rocky slopes along gullies, thin soil over rock, 600–700 m. Voucher: Verdcourt B & Polhill RM 2711 (EA).

***Polygalapetitiana* A.Rich.** – Habit: Herb. Habitat: Woodland and grassland, 1040–1350 m. Vouchers: Mwachala G et al. EW371 & 2664 (EA).

***Polygalasenensis* Klotzsch** – Habit: Herb. Habitat: Scattered tree grassland, grassland, 1350–1550 m. Vouchers: Kabuye CHS 82/98 & 140 (EA).

***Polygalasphenoptera* Fresen**. – Habit: Herb. Habitat: Evergreen bushland, wooded grassland, grassland, 800–2170 m. Vouchers: SAJIT–006364, Watuma BM W0157 (EA, HIB), Kabuye CHS et al. NMK Exped. 749, Mwachala G et al. EW99, 119, 621, 958 & 3244, Wakanene KM & Mwangangi OM 531, Faden RB et al. NMK Exped. 116, Beentje HJ et al. NMK Exped. 1142, Gillett JB, Burtt BL & Osborn RM 17081, Gilbert MG 5825 & 6105, Joanna B 8818, Ossent JR 89 (EA).

**F132. POLYGONACEAE**

3 Genera, 8 species

***Oxygonumsalicifolium* Dammer** – Habit: Herb. Habitat: Grassland and disturbed ground, 800–1371 m. Vouchers: Mwachala G et al. EW1310 & 1344, Kabuye CHS 82/24, Kabuye CHS et al. NMK Exped. 662 (EA).

***Oxygonumsinuatum* (Hochst. & Steud. ex Meisn.) Dammer** – Habit: Herb. Habitat: Cultivated and other ground suited to weed growth, 575–625 m. Vouchers: Hucks M 987, Kuchar P, Msafiri F & Karime N 5699 (EA).

***Oxygonumstuhlmannii* Dammer** – Habit: Herb. Habitat: Cultivated or disturbed ground, 600–762 m. Vouchers: Jeffery GW 714, Hucks M 774 & 775 (EA).

***Persicariadecipiens* (R.Br.) K.L.Wilson** – Habit: Herb. Habitat: Damp places, often growing in water, 800–1371 m. Vouchers: Mwachala G et al. EW1344, 1693 & 2565, Kabuye CHS 82/24, Kabuye CHS et al. NMK Exped. 662, Mungai GM et al. EW1310 & 2832 (EA).

***Persicariasenegalensis* (Meisn.) Soják** – Habit: Herb. Habitat: By riversides, damp places, growing in water, 800–1650 m. Vouchers: SAJIT–005359 (EA, HIB), Kabuye CHS 82/54, Mwachala G et al. EW1692, Jeffrey C K813, Lubai lK HB12 (EA).

***Persicariastrigosa* (R.Br.) H.Gross** – Habit: Herb. Habitat: Riverbanks, wet places, ca. 1200 m. Voucher: Wakanene KM & Mwangangi OM 500 (EA).

***Rumexabyssinicus* Jacq.** – Habit: Herb. Habitat: Margins of upland rainforest, secondary bushland, abandoned cultivations, 1323–1817 m. Vouchers: Watuma BM W0102 & W0251 (EA, HIB).

***Rumexusambarensis* (Dammer) Dammer** – Habit: Herb. Habitat: Open mist-forest, grassland, bushland, exposed rocky slopes, 1400–1900 m. Vouchers: SAJIT–004547 (EA, HIB), Wakanene KM & Mwangangi OM 242 (EA).

**F133. PORTULACACEAE**

1 Genera, 5 species

***Portulacagrandis* Peter** – Habit: Herb. Habitat: Deciduous bushland, rocky outcrops, 875–1400 m. Vouchers: Watuma BM W0193 (EA, HIB), Gilbert MG 5822, Bally PRO 13403 (EA).

***Portulacakermesina* N.E.Br.** – Habit: Herb. Habitat: Open bushland, rock crevices, 600–1300 m. Vouchers: Kiambati M 49, Verdcourt B & Polhill RM 2707, Faden RB, Evans A & Rathbun G 69/408 (EA).

***Portulacaoleracea* L.** – Habit: Herb. Habitat: Bushland, disturbed places, cultivated areas, 550–610 m. Vouchers: Napier ER 921 & 1046, Hucks M 595 (EA).

***Portulacaquadrifida* L.** – Habit: Herb. Habitat: Bare patches among rocks, disturbed places, weed of cultivation, 600–1027 m. Vouchers: Watuma BM W0313 (EA, HIB), Mwachala G et al. EW3317, Hucks M 757 (EA).

***Portulacawightiana* Wall. ex Wight & Arn.** – Habit: Herb. Habitat: Rocky outcrop, sandy soils, 590–700 m. Voucher: Faden RB, Evans A & Rathbun G 69/407 (EA).

**F134. PRIMULACEAE**

4 Genera, 5 species

***Ardisiandrawettsteinii* J.Wagner** – Habit: Herb. Habitat: Upland rainforest and evergreen bushland on roadsides and pathsides, 1400–2200 m. Vouchers: SAJIT–006367 (EA, HIB), Drummond & Hemsley 4284, Faden RB et al. NMK Exped. 47 & 146, Beentje HJ et al. NMK Exped. 908, Friis I & Hansen OJ 2674, Gillett JB et al. 17078, Verdcourt B & Polhill 2729, Christenhusz MJM et al. 6653 (EA).

***Embeliaschimperi* Vatke** – Habit: Liana. Habitat: Rainforest, forest edge, upland evergreen bushland, 1170–1400 m. Vouchers: Mwachala G et al. EW196 & 3206 (EA).

***Maesalanceolata* Forssk.** – Habit: Tree. Habitat: Forest edges, secondary and riverine forest, 1400–2170 m. Vouchers: SAJIT–006389, Watuma BM W0041 & W0074 (EA, HIB), Wakanene KM & Mwangangi OM 216 & 663, Faden RB et al. NMK Exped. 50, 113 & 325, Beentje HJ et al. NMK Exped. 842 & 917, Medley KE 978, Raler I 0003, Joanna B 8784, Greenway PG 14671, Sanganyi SKS 1 (EA).

***Myrsineafricana* L.** – Habit: Shrub. Habitat: Upland forest edges, 1400–2137 m. Vouchers: Beentje HJ 2135, De Block P et al. 303 & 308, Faden RB et al. NMK Exped. 87 & 299, Drummond RB & Hemsley JH 4290, Medley KE 1000, Bally PRO 8756 (EA).

***Myrsinemelanophloeos* (L.) R.Br. ex Sweet** – Habit: Tree. Habitat: Upland forest, open woodland, scrub near streams, 1225–1923 m. Vouchers: SAJIT–006352 (EA, HIB), Watuma BM W0085 (EA, HIB), Wakanene KM & Mwangangi OM 283 & 441, Faden RB et al. NMK Exped. 208, Beentje HJ et al. NMK Exped. 1172, Faden RB et al. 70/575, 744 & 71/1026, Seki T et al. JKCAT 1583, Dale IR 1133, Mwachala G s.n., Medley KE s.n. (EA).

**F135. PROTEACEAE**

3 Genera, 3 species

***Faureasaligna* Harv.** – Habit: Tree. Habitat: Woodland, grassland with scattered trees, 1080–1200 m. Vouchers: Mwachala G et al. EW584 & 811, Mungai GM et al. EW1439 & 3577 (EA).

*Grevillearobusta A.Cunn. ex R.Br. – Habit: Tree. Habitat: Woodland, grown in settlements, 650–1000 m. Voucher: Medley KEM s.r. (EA).

***Proteagaguedi* J.F.Gmel.** – Habit: Tree. Habitat: Woodland, exposed rocky places, ca. 1200 m. Voucher: Mungai GM et al. 1439 (EA).

**F136. PUTRANJIVACEAE**

1 Genus, 1 species

**DrypetesgerrardiiHutch.var.gerrardii** – Habit: Tree. Habitat: Upland dry evergreen forest, riverine, 883–1875 m. Vouchers: Watuma BM W0188 (EA, HIB), Christenhusz MJM & Kamau P et al. 6634, Faden RB et al. NMK Exped. 326, Beentje HJ et al. NMK Exped. 1070, Medley KE 669, 696 & 1010, Raler I 0067 (EA).

**F137. RANUNCULACEAE**

1 Genus, 2 species

***Clematisbrachiata* Thunb.** – Habit: Climber. Habitat: Forest edges and clearings, 1120–1900 m. Vouchers: Mwachala G et al. EW1699 & 3129, Wakanene KM & Mwangangi OM 551, 644 & 727, Faden RB et al. NMK Exped. 239, 365 & 1047, Raler I 0137, De Block P, Muasya, Stieperaere H & Bytebier 283 & 319 (EA).

***Clematissimensis* Fresen.** – Habit: Liana. Habitat: Forest edge and bushland, 1300–1848 m. Voucher: SAJIT–005354, Watuma BM W0086 (EA, HIB).

**F138. RHAMNACEAE**

6 Genera, 10 species

***Berchemiadiscolor* (Klotzsch) Hemsl.** – Habit: Tree. Habitat: Montane woodland, thicket, semi-desert grassland, wooded grassland, 500–1000 m. Vouchers: Dale IR 3676, Medley KE 449 & 705 (EA).

***Gouanialongispicata* Engl.** – Habit: Liana. Habitat: Forest margins and disturbed areas, riverine thickets and wooded grasslands, 1500–2170 m. Vouchers: SAJIT–004568 (EA, HIB), Faden RB et al. NMK Exped. 80 & 1063, Gardner HM 2979 (EA).

***Helinusintegrifolius* (Lam.) Kuntze** – Habit: Liana. Habitat: Thickets in wooded grassland, forest margins, 600–750 m. Vouchers: Faden RB & Faden AJ 74/262, Mrs. Robertson SA 6531, Greenway PJ & Kanuri K 12816, Dale IR 2046 (EA).

***Helinusmystacinus* (Aiton) E.Mey. ex Steud.** – Habit: Liana. Habitat: Forest margins, secondary bushland, wooded grassland, 650–1000 m. Vouchers: SAJIT–005383, Watuma BM W0167 (EA, HIB), Medley KE 826 (EA).

***Maesopsiseminii* Engl.** – Habit: Tree. Habitat: Rainforest, riverine forest, 1700–1875 m. Vouchers: Faden RB et al. NMK Exped. 507, Raler I 0082 (EA).

***Scutiamyrtina* (Burm.f.) Kurz** – Habit: Shrub. Habitat: Forest margins, montane woodland, bushland, 650–1100 m. Vouchers: Mwachala G et al. EW378, Medley KE 792 (EA).

***Ziziphusabyssinica* Hochst.** – Habit: Shrub/Tree. Habitat: Scattered tree grassland, bushland, 670–900 m. Vouchers: Medley KE 963, Johnston HH (EA).

***Ziziphusmauritiana* Lam.** – Habit: Shrub/Tree. Habitat: Woodland, bushland, disturbed areas near settlements, along roads, 650–1000 m. Voucher: Medley KE 528 (EA).

***Ziziphusmucronata* Willd.** – Habit: Tree. Habitat: *Acacia*-*Terminalia* woodland and bushland, 650–1000 m. Vouchers: Medley KE 414, 417 & 893, Bally PRO 8641 (EA).

***Ziziphuspubescens* Oliv.** – Habit: Shrub/Tree. Habitat: Riverine, deciduous woodland and bushland, 640–1372 m. Vouchers: Kabuye CHS et al. NMK Exped. 610, Joanna B & Opiko 8869, Medley KE 836 & 1029 (EA).

**F139. RHIZOPHORACEAE**

1 Genus, 4 species

***Cassipoureacelastroides* Alston** – Habit: Shrub/Tree. Habitat: Montane woodland, on rocky hillsides, 610–1200 m. Vouchers: Mwachala G et al. EW3330, Luke WRQ et al. 5519 & 6418, Medley KE 852 & 886, Faden RB et al. 69/415, Faden RB & Faden AJ 74/469, Gillett JB 19567 & 19573, Hildebrandt JM 2465 (EA).

***Cassipoureagummiflua* Tul.** – Habit: Tree. Habitat: Upland forest, 1100–1200 m. Vouchers: Luke WRQ & Bytebier B et al. 5324, Medley KE 624 & 665 (EA).

***Cassipoureamalosana* (Baker) Alston** – Habit: Shrub/Tree. Habitat: Dry upland forest, under canopy of moist forest, 1350–1500 m. Voucher: Mwachala G et al. EW310 (EA).

***Cassipourearotundifolia* (Engl.) Alston** – Habit: Tree. Habitat: Dry evergreen mist forest, 900–1000 m. Vouchers: Faden RB, Evans A & Githui M 70/175, Archer PG 14346 (EA).

**F140. ROSACEAE**

2 Genera, 6 species

***Prunusafricana* (Hook.f.) Kalkman** – Habit: Tree. Habitat: Upland rainforest, forest edge, riverine forest, 1250–2000 m. Vouchers: SAJIT–004566 & 004596, Watuma BM W0307 (EA, HIB), Mwachala G et al. EW2577, Faden RB et al. NMK Exped. 306, Beentje HJ et al. NMK Exped. 838 & 918 (EA). Vulnerable.

*Rubusniveus Thunb. – Habit: Shrub. Habitat: Disturbed areas in woodland and scrub, 1700–1850 m. Vouchers: SAJIT–006404, Watuma BM W0098 (EA, HIB).

***Rubuspinnatus* Willd.** – Habit: Shrub. Habitat: Edges of upland rain-forest, clearings in forests by streams, secondary moist forests, 1220–1850 m. Vouchers: Faden RB et al. NMK Exped. 403, Napier ER 1116, Raler I 0101 (EA).

*Rubusrosifolius Sm. – Habit: Shrub. Habitat: Upland rainforest edges, secondary bushlands and abandoned cultivations, 1576–1875 m. Vouchers: Watuma BM W0099 (EA, HIB), Faden RB et al. NMK Exped. 347, Gillett JB, Burtt BL & Osborn RM 17065 (EA).

***Rubusscheffleri* Engl.** – Habit: Shrub. Habitat: Upland rainforest margins and evergreen bushland, ca. 2211 m. Voucher: SAJIT–006375 (EA, HIB).

**RubussteudneriSchweinf.var.dictyophyllus (Oliv.) R.A.Graham** – Habit: Shrub. Habitat: Edges and clearings in upland rainforest, upland evergreen bushland, 1800–2200 m. Voucher: Mwachala G et al. EW1723 (EA).

**F141. RUBIACEAE**

41 Genera, 75 species

***Anthospermumherbaceum* L.f.** – Habit: Herb. Habitat: Forest edges, woodland, roadside weed, 1800–2170 m. Vouchers: Faden RB et al. NMK Exped. 136, Drummond RB & Hemsley JH 4302, Gillett JB 17260, Lewis WH 5932 & 5937 (EA).

***Breonadiasalicina* (Vahl) Hepper & J.R.I.Wood** – Habit: Tree. Habitat: Gallery forest by rivers & streams, 1372–1500 m. Vouchers: Kabuye CHS 82/36, Raler I 0138, Bally PRO 8579 (EA).

***Bullockiadyscritos* (Bullock) Razafim., Lantz & B.Bremer** – Habit: Shrub. Habitat: Rocky area, roadside banks, grazed areas, 762–1330 m. Vouchers: Mwachala G et al. EW3252, Gilbert MG & Gilbert CI 6102, Gardner HM 3000 (EA).

***Bullockiamombazensis* (Baill.) Razafim., Lantz & B.Bremer** – Habit: Shrub. Habitat: Wooded grassland to forest, bushland, 650–1000 m. Vouchers: Faden RB, Evans A & Githui M 70/176, Medley KE 506, 899 & 986 (EA).

**CanthiumoligocarpumHiernsubsp.friesiorum (Robyns) Bridson** – Habit: Tree. Habitat: Mist forest, 1600–1640 m. Voucher: Faden RB & Evans A 71/181 (EA).

**CanthiumoligocarpumHiernsubsp.intermedium Bridson** – Habit: Shrub/Tree. Habitat: Mist forest understorey & margins, 1700–1875 m. Vouchers: Wakanene KM, Mwangangi OM, Dunn B 367, Faden RB et al. NMK Exped. 253, Faden RB et al. 71/1009 (EA). Vulnerable.

**CanthiumoligocarpumHiernsubsp.oligocarpum** – Habit: Shrub. Habitat: Forest, 1540–1900 m. Vouchers: De Block P, Muasya AM, Stieperaere H & Bytebier B 276, 478 & 479 (EA).

***Catunaregamnilotica* (Stapf) Tirveng.** – Habit: Shrub/Tree. Habitat: Montane woodland, riverine bushland, thicket edges, 650–1000 m. Vouchers: Kabuye CHS et al. NMK Exped. 739, Medley KE 760, 889 & 1003 (EA).

**Chamaepentashindsioides(K.Schum.)Kårehed & B.Bremervar.glabrescens (Verdc.) Kårehed & B.Bremer** – Habit: Shrub. Habitat: Mist forest, ca. 1480 m. Voucher: Luke WRQ, Bytebier B & Pakia M 5359 (EA).

**Chamaepentashindsioides(K.Schum.)Kårehed & B.Bremervar.hindsioides** – Habit: Subshrub. Habitat: Upland forests margins & bushlands, 1425–1981 m. Vouchers: Watuma BM W0039 (EA, HIB), Luke WRQ et al. 4196, De Block P, Muasya, Stieperaere H & Bytebier B 301A, 334, 363 & 366, Faden RB et al. NMK Exped. 254, Beentje HJ et al. NMK Exped. 906 & 1009, Gillett JB, Burtt BL & Osborn RM 17151, Gillett JB 18770, Dale IR 3813, Bally J 13573 & 13596, Hemp s.n. (EA).

^**E**^**ChassaliadiscolorK.Schum.subsp.taitensis Verdc.** – Habit: Shrub. Habitat: Understorey in rainforest, 1450–1900 m. Vouchers: SAJIT–004603, 006342 & 006343, Watuma BM W0057 (EA, HIB), Wakanene KM & Mwangangi OM 276, Luke WRQ et al. 5490, Christenhusz MJM et al. 6638, De Block P, Muasya, Stieperaere H & Bytebier B 277 & 326, Faden RB et al. NMK Exped. 189, Bytebier B 1199, Hemp A 5253, Dale IR 3837 (EA).

***Chassaliaparvifolia* K.Schum.** – Habit: Shrub. Habitat: Evergreen forest, 1350–1900 m. Vouchers: SAJIT–006344 (EA, HIB), Luke WRQ, Bytebier B & Pakia M 5350, Faden RB et al. NMK Exped. 561, Beentje HJ et al. NMK Exped. 882B & 1071, De Block P, Muasya, Stieperaere H & Bytebier B 251 & 471, Drummond RB & Hemsley JH 4332 (EA).

^**NE**^***Coffeafadenii* Bridson** – Habit: Tree. Habitat: Mist forest, 1400–1850 m. Vouchers: Wakanene KM & Mwangangi OM 462 & 651, Faden RB et al. NMK Exped. 342 & 404, Faden RB & Faden AJ 72/269, Faden RB, Evans A, Msafiri F & Smeenk C et al. 71/56, Faden RB & Githui M 70/743, Faden RB et al. 70/559, Raler I 0034 & 0039, Goodrich J EAH 17158 (EA). Endangered.

**CoffeasessilifloraBridsonsubsp.sessiliflora** – Habit: Shrub. Habitat: Moist and dry evergreen forest, 1080–1370 m. Vouchers: Watuma BM W0295 (EA, HIB), Mwachala G et al. EW3178, Luke WRQ et al. 5532 (EA).

***Coffeazanguebariae* Lour.** – Habit: Shrub. Habitat: Riverine, ca. 800 m. Voucher: Mwachala G et al. EW3333 (EA). Vulnerable.

**Coptospermagraveolens(S.Moore)Degreefvar.graveolens** – Habit: Shrub/Tree. Habitat: Montane woodland, evergreen bushland and thicket, 600–1000 m. Vouchers: SAJIT–005362 (EA, HIB), Kabuye CHS et al. NMK Exped. 619, Medley KE 529, 546, 571, 715 & 736, Gilbert MG & Gilbert CI 6115 (EA).

***Cordylostigmaprolixipes* (S.Moore) Groeninckx & Dessein** – Habit: Herb. Habitat: Rocky areas, *Euphorbia*-*Croton* deciduous bushland, 1000–1067 m. Vouchers: Faden RB & Evans A 71/43, Bally PRO 8715 (EA).

**Cremasporatriflora(Thonn.)K.Schum.subsp.confluens (K.Schum.) Verdc.** – Habit: Shrub/Tree. Habitat: Swamp forest, plantation forest, thicket, ca. 1225 m. Voucher: Beentje HJ et al. NMK Exped. 1173 (EA, NMNH, WAG).

**Cremasporatriflora(Thonn.)K.Schum.subsp.triflora** – Habit: Shrub/Tree. Habitat: Understorey in evergreen and riverine forest, 1200–1640 m. Vouchers: Kabuye CHS et al. NMK Exped. 803, Faden RB et al. 70/444, Medley KE 604, Aver’yanov L 200 (EA).

***Dirichletiaglaucescens* Hiern** – Habit: Shrub/Tree. Habitat: Deciduous woodland, bushland and thicket, rocky outcrops, 450–1000 m. Vouchers: Medley KE 491 & 707, Faden RB & Faden AJ 74/530, Verdcourt B 5312 (EA), Ossent JR 134, Hildebrandt JM 2595, Lavranos JJ 11896, Gillett JB & Burtt BL 17170, Hooper SS & Townsend CC 1258, Belsky JB 492 (EA).

**Empogonaovalifolia(Hiern)Tosh & Robbr.var.glabrata (Oliv.) Tosh & Robbr.** – Habit: Shrub. Habitat: Evergreen forest, dry thickets and wooded grassland, 960–1875 m. Vouchers: SAJIT–004636 & 005377 (EA, HIB), Wakanene KM & Mwangangi OM 522, Luke WRQ et al. 6419, Faden RB et al. NMK Exped. 523 & 1180, Faden RB & Faden AJ 74/480, Gillett JB 19571, Archer PG 14344, Joanna B 8882 (EA). Vulnerable.

**Empogonaovalifolia(Hiern)Tosh & Robbr.var.ovalifolia** – Habit: Shrub. Habitat: Evergreen forest, montane woodland, secondary vegetation, 650–1000 m. Vouchers: Mwachala G et al. EW86, Medley KE 470, 601, 783, 809 & 903 (EA).

**Empogonaovalifolia(Hiern)Tosh & Robbr.var.taylorii (S.Moore) Tosh & Robbr.** – Habit: Shrub. Habitat: Dry forest, bushland with scattered trees, thicket, ca. 800 m. Voucher: Kabuye CHS 82/94 (EA). Vulnerable.

***Galinierasaxifraga* (Hochst.) Bridson** – Habit: Tree. Habitat: Forest, 1740–2170 m. Vouchers: Faden RB et al. NMK Exped. 150, Beentje HJ et al. NMK Exped. 919 (EA).

^**NE**^***Galiumbrenanii* Ehrend. & Verdc.** – Habit: Herb. Habitat: Heavily disturbed evergreen forest, 1900–2205 m. Vouchers: Verdcourt B & Polhill RM 2732(S), Faden RB & Faden AJ 72/248, Faden RB et al. NMK Exped. 94 (EA).

**GaliumspuriumL.subsp.africanum Verdc.** – Habit: Herb. Habitat: Cultivations, forest edges, bushlands and roadsides, 1400–1640 m. Voucher: Faden RB et al. NMK Exped. 10 (EA).

**GardeniavolkensiiK.Schum.subsp.volkensii** – Habit: Tree. Habitat: Montane woodland, grassland with scattered trees, 487–1000 m. Vouchers: Medley KE 870, Greenway PJ & Kanuri K 13026 (EA).

***Heinseniadiervilleoides* K.Schum.** – Habit: Shrub/Tree. Habitat: Rainforest, moist evergreen forest, 1500–1875 m. Vouchers: Faden RB et al. NMK Exped. 209, Faden RB et al. 71/982 (EA).

**Heinsiacrinita(Wennberg)G.Taylorsubsp.parviflora (K.Schum. & K.Krause) Verdc.** – Habit: Shrub. Habitat: Bushland, 550–650 m. Voucher: Medley KEM 961 (EA).

**HymenodictyonparvifoliumOliv.subsp.parvifolium** – Habit: Shrub. Habitat: Acacia-Combretum thicket, mixed bushland, rocky outcrops, 610–1000 m. Vouchers: SAJIT–005365, Watuma BM W0181 (EA, HIB), Medley KEM 537, 560 & 703, Sacleux C 2298, Dale IR 3761, Greenway PJ & Kanuri K 12680 (EA)

**HymenodictyonparvifoliumOliv.subsp.scabrum (Stapf) Verdc.** – Habit: Shrub. Habitat: Open woodland, thicket, rocky hills, ca. 520 m. Voucher: Faden RB & Faden AJ 74/247 (EA).

***Keetiagueinzii* (Sond.) Bridson** – Habit: Liana/Shrub/Tree. Habitat: Forest and woodland, often on swampy ground, 1400–1900 m. Vouchers: SAJIT–004602 & 005321, Watuma BM W0240 (EA, HIB), Faden RB et al. NMK Exped. 385, Beentje HJ et al. NMK Exped. 843, De Block P, Muasya, Stieperaere H & Bytebier B 305, 324, 325, 333 & 472, Faden RB, Faden AJ & Smeenk C 71/1014, Raler I 0059 (EA).

**LasianthuskilimandscharicusK.Schum.subsp.glabrescens Jannerup** – Habit: Shrub/Tree. Habitat: Montane forest, often by streams, forest edge clearings and secondary vegetation, 1425–1850 m. Vouchers: Beentje HJ et al. NMK Exped. 955, Faden RB, Faden AJ, Smeenk C, Smeenk N & McNee J et al. 71/983 (EA).

**LasianthuskilimandscharicusK.Schum.subsp.hirsutus Jannerup** – Habit: Shrub/Tree. Habitat: Upland evergreen forest, 1040–1660 m. Vouchers: De Block P, Muasya, Stieperaere H & Bytebier B 360, 361, 480 & 486 (EA).

**LasianthuskilimandscharicusK.Schum.subsp.kilimandscharicus** – Habit: Shrub/Tree. Habitat: Understorey of upland rainforest, 1000–1950 m. Vouchers: SAJIT–006346 (EA, HIB), De Block P, Muasya, Stieperaere H & Bytebier B 247, 249 & 260, Drummond RB & Hemsley JH 4352, Faden RB et al. NMK Exped. 192, Bally PRO 8748, Medley KE 605 & 680 (EA).

**Meynatetraphylla(Schweinf. ex Hiern)Robynssubsp.comorensis (Robyns) Verdc.** – Habit: Shrub/Tree. Habitat: Dry or moist evergreen forest and woodland, riverine, by waterholes, 650–1000 m. Vouchers: SAJIT–005408 (EA, HIB), Medley KE 968 (EA).

***Mitragynarubrostipulata* (K.Schum.) Havil.** – Habit: Tree. Habitat: Fringing forest, wet upland forest, along streams, 1425–1850 m. Vouchers: Faden RB et al. NMK Exped. 770, Faden RB et al. 71/241 (EA).

**Oldenlandiaherbacea(L.)Roxb.var.holstii (K.Schum.) Bremek.** – Habit: Herb. Habitat: *Philippia* heath, grassland, open woodland, rock outcrops, 914–1900 m. Vouchers: SAJIT–005346 (EA, HIB), De Block P, Muasya, Stieperaere H & Bytebier B 306 & 310, Faden RB et al. NMK Exped. 3, 230 & 362, Lewis WH 5931, Hildebrandt JM 2435, Ossent JR 87 (EA).

***Oldenlandiarupicola* (Sond.) Kuntze** – Habit: Herb. Habitat: Rock crevices and outcrops in *Philippia* scrub and upland forest, 2000–2170 m. Vouchers: Drummond RB & Hemsley JH 4299, Verdcourt B & Polhill RM 2722, Faden RB et al. NMK Exped. 76 (EA).

**OxyanthusgoetzeiK.Schum.subsp.keniensis Bridson** – Habit: Shrub/Tree. Habitat: Forest, 825–1875 m. Vouchers: Mwachala G et al. EW3327, Faden RB et al. NMK Exped. 551, Joanna B 8883, Faden RB, Evans A & Rathbun G 69/437, Thulin MI 551 (EA).

**Oxyanthuspyriformis(Hochst.)Skeelssubsp.brevitubus Bridson** – Habit: Shrub/Tree. Habitat: Dry forest, ca. 1200 m. Voucher: Medley KE 666 (EA). Vulnerable.

**^NE^Oxyanthuspyriformis(Hochst.)Skeelssubsp.longitubus Bridson** – Habit: Shrub/Tree. Habitat: Moist forest, ca. 1040 m. Voucher: De Block P, Muasya, Stieperaere H & Bytebier B 356 (EA). Endangered.

**OxyanthusspeciosusDC.subsp.stenocarpus (K.Schum.) Bridson** – Habit: Shrub/Tree. Habitat: Forest, 1540–2117 m. Vouchers: SAJIT–003289 & 004521 (EA, HIB), Bytebier B 1218, De Block P, Muasya, Stieperaere H & Bytebier B 248, 263, 265, 317, 335, 475, 481A, 485A, 488 & 499A, Faden RB et al. NMK Exped. 240, Beentje HJ et al. NMK Exped. 893, Raler I 0008 (EA).

**^NE^*Parapentasbattiscombei* Verdc.** – Habit: Herb. Habitat: Forest floor and clearings in upland evergreen forest, 1040–1981 m. Vouchers: Luke WRQ & Luke PA 4139 & 9431, Christenhusz MJM et al. 6662, Beentje HJ et al. NMK Exped. 828 & 1064, De Block P et al. 354, Bally PRO 8768 & 138706 (EA).

***Pauridianthapaucinervis* (Hiern) Bremek.** – Habit: Tree. Habitat: Upland evergreen forest, 1200–1875 m. Vouchers: SAJIT–005309, Watuma BM W0018 & W0050 (EA, HIB), Christenhusz MJM et al. 6633, Joanna B 9030, De Block P et al. 244, 250, 253, 264, 353, 359, 570, Faden RB et al. NMK Exped. 213 & 364, Faden RB & Faden AJ 77/328, Gillett JB, Burtt BL, Osborn RM 17125, Medley KE 930, Lubai lK HB29 (EA).

**PavettaabyssinicaFresen.subsp.abyssinica** – Habit: Shrub/Liana. Habitat: Forest and associated scrub, ca. 1630 m. Voucher: Christenhusz MJM, Kamau P, Chase MW, Mbale M & Kyaa J 6666 (EA).

**PavettacrebrifoliaHiernvar.crebrifolia** – Habit: Shrub. Habitat: Upland and lowland evergreen forest, 660–1640 m. Vouchers: Beentje HJ et al. NMK Exped. 887, Gillett JB 18747, Medley KE 436 & 883 (EA).

***Pavettadolichantha* Bremek.** – Habit: Shrub. Habitat: Bushland and forest edges, ca. 762 m. Voucher: Polhill RM & Kibuwa SP 966 (EA).

**PavettagardeniifoliaA.Rich.var.gardeniifolia** – Habit: Shrub. Habitat: Bushland to forest, often on rocky ground, 820–1400 m. Vouchers: Faden RB, Evans A & Githui M 70/164, De Block P, Muasya, Stieperaere H & Bytebier B 347, Medley KE 671, Hildebrandt JM 2570 (EA).

**PavettasepiumK.Schum.var.sepium** – Habit: Shrub. Habitat: Ground-water forests, bushland on hillsides, ca. 1219 m. Voucher: Napier ER 1139 (EA). Vulnerable.

***Pavettateitana* K.Schum.** – Habit: Shrub. Habitat: Forest margins on rocky ground, 660–1250 m. Vouchers: Faden RB, Evans A & Rathbun G 69/430, De Block P et al. 370, Lavranos JJ 11897, Medley KE 469, 730 & 941, Gillett JB 18738, Bally J 13560, Joanna B 9402 (EA). Vulnerable.

**Pentaslanceolata(Forssk.)Deflerssubsp.lanceolata** – Habit: Subshrub. Habitat: Evergreen forest, bushland, thicket, grassland, 1700–1827 m. Vouchers: Watuma BM W0090 (EA, HIB), Beentje HJ 2133 (EA).

**Pentaslanceolata(Forssk.)Deflerssubsp.quartiniana (A.Rich.) Verdc.** – Habit: Subshrub. Habitat: Edges of paths and glades in secondary evergreen forest, 1350–1560 m. Vouchers: Beentje HJ et al. NMK Exped. 828 & 1064, Bytebier B 1219 (EA).

***Pentaspubiflora* S.Moore** – Habit: Subshrub. Habitat: Dry riverine forest and forest edges, scrub, grassland, lava slopes, 1189–1875 m. Vouchers: Faden RB et al. NMK Exped. 11 & 549, Lewis WH 5930 (EA).

**Pentaszanzibarica(Klotzsch)Vatkevar.intermedia Verdc.** – Habit: Subshrub. Habitat: Grassland, rocky hillsides, 1600–1830 m. Voucher: Lubai lK HB16 (EA).

***Pentaszanzibarica* (Klotzsch) Vatke subsp.. *zanzibarica*** – Habit: Subshrub. Habitat: Forest glades and edges, open grassland with scattered trees, 1040–2170 m. Vouchers: Watuma BM W0217 (EA, HIB), Faden RB et al. NMK Exped. 114, De Block P, Muasya, Stieperaere H & Bytebier B 281, 289 & 364, Bally PRO 8707 (EA).

***Pentodonpentandrus* (Schumach. & Thonn.) Vatke** – Habit: Herb. Habitat: Seasonally wet ground, moist forest margins, along rivers, 800–1200 m. Vouchers: SAJIT–005353 (EA, HIB), Kabuye CHS et al. NMK Exped. 666 (EA).

***Phyllopentasschimperi* (Hochst.) Y.D.Zhou & Q.F.Wang** – Habit: Subshrub. Habitat: Evergreen forest in volcanic soils, 1425–2000 m. Vouchers: Beentje HJ et al. NMK Exped. 911 & 953 (EA).

**PolysphaeriamultifloraHiernsubsp.pubescens Verdc.** – Habit: Tree. Habitat: Riverine forest and bushland, 800–1158 m. Vouchers: Kabuye CHS et al. NMK Exped. 660, Bally PRO 8578 (EA).

***Polysphaeriaparvifolia* Hiern** – Habit: Tree. Habitat: Dry evergreen forest, woodland, 1000–1640 m. Voucher: Medley KE 699 (EA).

^**NE**^***Psychotriaalsophila* K.Schum.** – Habit: Shrub. Habitat: Evergreen forest, 1650–1900 m. Vouchers: Watuma BM W0283 (EA, HIB), Christenhusz MJM et al. 6616, De Block P, Muasya, Stieperaere H & Bytebier B 293, 294, 296 & 320, Beentje HJ 2191, Faden RB et al. NMK Exped. 519, Faden RB, Faden AJ, Smeenk C & Geer T 72/213 (EA). Vulnerable.

**Psychotriacapensis(Eckl.)Vatkesubsp.riparia (K.Schum. & K.Krause) Verdc.** – Habit: Shrub/Tree. Habitat: Evergreen bushland, forest edges, riparian forest, 800–1640 m. Vouchers: Kabuye CHS et al. NMK Exped. 641, Medley KE 474, Gilbert MG & Gilbert CI 6103 (EA).

**^NE^*Psychotriacrassipetala* E.M.A.Petit** – Habit: Tree. Habitat: Evergreen forest, 1000–1925 m. Vouchers: Wakanene KM & Mwangangi OM 440, 619A, 680 & 736, De Block P et al. 271 & 484, Drummond RB & Hemsley JH 4336, Faden RB et al. NMK Exped. 214 & 426, Faden RB, Evans A, Githui M & Smeenk C 71/258, Faden RB & Faden AJ 72/274, Faden RB & Evans A 69/875, Dale IR 3814 (EA). Endangered.

***Psychotriacyathicalyx* E.M.A.Petit** – Habit: Tree. Habitat: Upland evergreen forest, 1900–2205 m. Faden RB & Faden AJ 72/256 (EA). Vulnerable.

***Psychotrialauracea* (K.Schum.) E.M.A.Petit** – Habit: Shrub. Habitat: Evergreen forest, 1000–1900 m. Vouchers: Watuma BM W0048 (EA, HIB), De Block P et al. 340, 342, 343, 346, 373 & 374, Beentje HJ 2143, Faden RB et al. NMK Exped. 37, 205 & 333, Beentje HJ et al. NMK Exped. 863 & 998, Faden RB & Faden AJ 77/339, Dale IR 3851, Raler I 0049, Medley KE 593, 616, 647, 650 & 894 (EA).

**^E^*Psychotriapetitii* Verdc.** – Habit: Tree. Habitat: Evergreen forest, 1432–2180 m. Vouchers: SAJIT–006348, Watuma BM W0034 & W0045 (EA, HIB), Luke WRQ & Luke PA 5498, Wakanene KM & Mwangangi OM 334 & 473, Drummond RB & Hemsley JH 4343, Christenhusz MJM et al. 6626 & 6668, De Block P et al. 246, 311, 469 & 477, Faden RB et al. NMK Exped. 165 & 210, Hemp A 5187, Raler I 0012, Faden RB et al. 70/139, 739 & 72/256, Bally PRO 8712 & 8751, Dale IR 3815 (EA). Endangered.

**^NE^*Psychotriapseudoplatyphylla* E.M.A.Petit** – Habit: Shrub. Habitat: Evergreen forest, 1450–1960 m. Vouchers: SAJIT–004516, 004565 & 004595, Wakanene KM, Mwangangi OM & Dunn B 336, 371, 639 & 736, Mutiso 29, Christenhusz, MJM et al. 6625, Drummond RB & Hemsley JH 4334, De Block P et al. 242, 254, 258, 261, 262, 269, 272, 279, 328, 332, 491 & 492, Faden RB et al. NMK Exped. 207, Beenjte HJ et al. NMK Exped. 889, Hemp A 5192, Faden RB et al. 71/233 & 77/336, Dale IR 3816, Raler I 0002 (EA). Vulnerable.

**PsychotriapunctataVatkevar.punctata**– Habit: Shrub. Habitat: Forest edges, Bushland, open woodland, often in rocky places, *Acacia* thicket, grassland, cultivated hillsides, 800–1400 m. Vouchers: Mwachala G et al. EW3321, Kabuye CHS 82/143, Kabuye CHS et al. NMK Exped. 656, Faden RB & Faden AJ 74/511, Drummond RB & Hemsley JH 4398, Bally PRO 8583 (EA).

**^E^*Psychotria* sp. B of FTEA** – Habit: Shrub. Habitat: Upland evergreen forest, 1650–1800 m. Vouchers: Faden RB et al. 72/211, Gilbert MG & Gilbert CI 6103 (EA).

**^E^*Psychotriataitensis* Verdc.** – Habit: Tree. Habitat: Mist forest with *Syzygium* and *Rapanea* sp., 1060–1600 m. Vouchers: Luke WRQ & Luke PA 4151 & 9435, De Block P, Muasya, Stieperaere H & Bytebier B 350 & 365, Faden RB, Evans A, Smeenk C & Kariuki B 71/153 (EA). Endangered.

***Psydraxlividus* (Hiern) Bridson** – Habit: Shrub. Habitat: Thicket and scrub, rocky hillsides, 836–960 m. Vouchers: Watuma BM W0201 (EA, HIB), Gillett JB 19572 (EA).

**Psydraxparviflorus(Afzel.)Bridsonsubsp.rubrocostata (Robyns) Bridson** – Habit: Shrub/Tree. Habitat: Forest, 1400–1875 m. Vouchers: Faden RB & Faden AJ 72/271, Gillett JB, Burtt BL & Osborn RM 17115, Beenje HJ et al. NMK Exped. 844, Faden RB et al. NMK Exped. 308 & 888, Medley KE 663 (EA).

***Psydraxpolhillii* Bridson** – Habit: Shrub. Habitat: Woodland and thicket, 550–650 m. Vouchers: Luke WRQ & Luke PA 4246, Medley KE 544 (EA). Vulnerable.

**Psydraxschimperianus(A.Rich.)Bridsonsubsp.schimperianus** – Habit: Shrub/Tree. Habitat: Forest, montane woodland, thicket or bushland, 650–1040 m. Vouchers: De Block P, Muasya, Stieperaere H & Bytebier B 348, Medley KE 635, 643, 735 & 859 (EA).

***Pyrostriaphyllanthoidea* (Baill.) Bridson** – Habit: Shrub. Habitat: In *Acacia*, *Commiphora* bushland, often on rocks, 650–1000 m. Vouchers: SAJIT–005402 (EA, HIB), Medley KE 487, 778, 855 & 962 (EA).

***Pyrostria* sp. A of FTEA** – Habit: Tree. Habitat: Dry evergreen forest. Voucher: Joanna B 9403 (EA).

***Rhodopentasparvifolia* (Hiern) Kårehed & B.Bremer** – Habit: Subhrub. Habitat: Dry evergreen forest, grassland with scattered trees, 701–1004 m. Vouchers: Watuma BM W0314 (EA, HIB), Polhill RM & Kibuwa SP 956 (EA).

**Rothmanniafischeri(K.Schum.)Bullock ex Oberm.subsp.verdcourtii Bridson** – Habit: Tree. Habitat: Bushland on rocky hillsides, 609–1371 m. Vouchers: Kabuye CHS 82/35, Kabuye CHS et al. NMK Exped 637, Mwachala G et al. EW913 & 1181, Kokwaro JO 2608, Cheseny CMC 102, Hildebrandt JM 2531, Joanna B 8987, Archer PG 635, Medley KE 549 & 564 (EA).

***Rothmanniaravae* (Chiov.) Bridson** – Habit: Tree. Habitat: Dry evergreen forest, thicket, 900–1900 m. Vouchers: De Block P, Muasya, Stieperaere H & Bytebier B 318, Faden RB, Githui M & Evans A 70/177, Archer PG 619 (EA).

^**NE**^***Rytigyniaeickii* (K.Schum. & K.Krause) Bullock** – Habit: Shrub. Habitat: Sub-montane forest, open bushland in granite areas, 1400–1875 m. Vouchers: Watuma BM W0079 (EA, HIB), Kabuye CHS 82/92, Luke WRQ et al. 5502, Chemusto 28, Faden RB et al. NMK Exped. 334, 473 & 535, Beentje HJ et al. NMK Exped. 875, Faden RB & Faden AJ 77/335, Raler I 0055 (EA). Vulnerable.

***Rytigyniauhligii* (K.Schum. & K.Krause) Verdc.** – Habit: Shrub. Habitat: Montane evergreen forest, 1000–2170 m. Vouchers: SAJIT–003300, 006408, Watuma BM W0054, W0078 & W0276 (EA, HIB), De Block P et al. 284A, 295, 298, 323, 330, 331 & 341, Beentje HJ et al. NMK Exped. 1075 & 1118, Faden RB et al. NMK Exped. 51, 119, 253, 262 & 511, Drummond RB & Hemsley JH 4399, Faden RB & Faden AJ 77/334, Gillett JB 23891, Dale IR 1999, Medley KE 913 & 992 (EA).

***Spermacoceprinceae* (K.Schum.) Verdc.** – Habit: Herb. Habitat: Grassland, forest and woodland clearings, 1427–2170 m. Vouchers: SAJIT–006410 (EA, HIB), De Block P, Muasya, Stieperaere H & Bytebier B 322, Faden RB et al. NMK Exped. 111 & 434 (EA).

***Tennantiasennii* (Chiov.) Verdc. & Bridson** – Habit: Shrub. Habitat: Dry bushland and woodland on red sandy soils, 525–800 m. Vouchers: Mrs. Robertson SA 6529, Medley KE 432, 494, 777, 863 & 960, Faden RB, Evans A & Rathbun G 69/414, Gillett JB 18772, Napier ER 1065, Ossent JR 144 (EA).

***Vangueriaapiculata* K.Schum.** – Habit: Tree. Habitat: Evergreen forest edges and thicket, rocky slopes, ca. 1814 m. Vouchers: SAJIT–006402 (EA, HIB).

**VangueriainfaustaBurch.subsp.rotundata (Robyns) Verdc.** – Habit: Tree. Habitat: Dry evergreen forest, woodland, 1800–2000 m. Vouchers: SAJIT–004584 (EA, HIB).

***Vangueriamadagascariensis* J.F.Gmel.** – Habit: Tree. Habitat: Evergreen forest, woodland, 650–1960 m. Vouchers: SAJIT–004567 (EA, HIB), Medley KE 564 & 1013 (EA).

**Vangueriaschumanniana(Robyns)Lantzsubsp.mucronulata (Robyns) ined.** – Habit: Shrub/Tree. Habitat: *Acacia*-*Grewia* thicket and bushland. Voucher: Lawton RM 1757 (EA).

**Vangueriaschumanniana(Robyns)Lantzsubsp.schumanniana** – Habit: Shrub/Tree. Habitat: *Acacia*-*Commiphora*-*Combretum* woodland and bushland, rocky slopes, 650–1000 m. Vouchers: Medley KE 634, 727 & 807 (EA).

**VangueriavolkensiiK.Schum.var.volkensii** – Habit: Tree. Habitat: Evergreen forest edges, rocky slopes, 800–1875 m. Vouchers: Watuma BM W0112 (EA, HIB), Kabuye CHS et al. NMK Exped. 602, Luke WRQ & Bytebier B et al. 5378, Beentje HJ et al. NMK Exped. 878, Drummond RB & Hemsley JH 4376, Raler I 0065, Faden RB et al. NMK Exped. 577 (EA).

**F142. RUTACEAE**

6 Genera, 14 species

***Calodendrumcapense* (L.f) Thunb.** – Habit: Tree. Habitat: Upland evergreen and riverine forests, 1200–1700 m. Voucher: Gardener in FD 2921 (EA).

***Clausenaanisata* (Willd.) Hook.f. ex Benth.** – Habit: Shrub. Habitat: Forest margins and regenerations, bushland, wooded grassland, 1700–1875 m. Vouchers: SAJIT–004600, Watuma BM W0069 (EA, HIB), Mwachala G et al. EW012, Faden RB et al. NMK Exped. 263 (EA).

***Fagaropsisangolensis* (Engl.) H.M.Gardner** – Habit: Tree. Habitat: Rainforest edges, drier evergreen forest, 1650–1800 m. Voucher: Faden RB et al. 72/209 (EA).

***Harrisoniaabyssinica* Oliv.** – Habit: Shrub. Habitat: Dry evergreen forest; forest edges and clearings, 550–1371 m. Vouchers: Watuma BM W0144 (EA, HIB), Mwachala G et al. EW2800, Mungai GM et al. EW1391 & 1657, Medley KE 944, Faden RB, Evans A & Rathbun G 69/416, Joanna B 8885 (EA).

**^E^*Veprisfadenii* (Kokwaro) Mziray** – Habit: Tree. Habitat: Mist forest, mountain and hill forests, 1200–1875 m. Vouchers: Luke WRQ & Luke PA 9436, Faden RB et al. NMK Exped. 575, Faden RB et al. 71/57 & 145, Joanna B 9032, Raler I 0037 (EA). Vulnerable.

***Veprisglomerata* (F.Hoffm.) Engl.** – Habit: Shrub/Tree. Habitat: Wooded grassland, deciduous woodland, 550–810 m. Vouchers: Medley KE 755, Vesey-FitzGerald 42 (EA).

**Vepris sp. nr. glomerata (F.Hoffm.) Engl.** – Habit: Shrub/Tree. Habitat: Montane woodland, 650–1000 m. Vouchers: Medley KE 480 & 509 (EA).

***Veprisnobilis* (Delile) Mziray** – Habit: Tree. Habitat: Evergreen forest, woodland, rocky hills, 1000–1875 m. Vouchers: Faden RB et al. NMK Exped. 464, Medley KE 1012 (EA).

***Veprissimplicifolia* (Engl.) Mziray** – Habit: Tree. Habitat: Evergreen forest, wooded grassland and bushland, 650–1875 m. Vouchers: Mwachala G et al. EW180 & 3114A, Kimuzi D 3, Faden RB et al. NMK Exped. 526, Medley KE 501 (EA).

***Vepristrichocarpa* (Engl.) Mziray** – Habit: Tree. Habitat: Upland forest, riverine associations, ca. 950 m. Voucher: Mungai GM et al. EW1762 (EA).

***Veprisuguenensis* Engl.** – Habit: Shrub. Habitat: Wooded grassland, riverine, bushland in rocky places, 610–1050 m. Vouchers: Luke WRQ & Luke PA 5514 & 6433, Faden RB & Faden AJ 74/514, Faden RB et al. 71/0114, Gardner HM 2961, Ossent JR 121 (EA).

***Zanthoxylumasiaticum* (L.) Appelhans, Groppo & J.Wen** – Habit: Liana. Habitat: Forest edges, riverine forest, bushland, 1000–1875 m. Vouchers: SAJIT–004122, 004562 & 005310, Watuma BM W0064 (EA, HIB), Faden RB et al. NMK Exped. 228 & 492, Raler I 0021, Medley KE 627 (EA).

***Zanthoxylumchalybeum* Engl.** – Habit: Tree. Habitat: Dry bushland and wooded grassland, 550–1000 m. Voucher: Medley KE 495 (EA).

***Zanthoxylumusambarense* (Engl.) Kokwaro** – Habit: Tree. Habitat: Upland forest, ca. 1400–1600 m. Vouchers: Joanna B 8935, Kimuzi D 2 (EA).

**F143. SALICACEAE**

6 Genera, 7 species

***Biviniajalbertii* Tul.** – Habit: Tree. Habitat: Evergreen forest, 1200–1600 m. Voucher: Medley KE 910 (EA). Near Threatened.

***Dovyalisabyssinica* (A.Rich.) Warb.** – Habit: Shrub. Habitat: Upland rainforest, dry evergreen forest, open wooded grassland, 1700–1875 m. Vouchers: Faden RB et al. NMK Exped. 242 & 503, Drummond RB & Hemsley JH 4285, Raler I 0092 (EA).

***Flacourtiaindica* (Burm.f.) Merr.** – Habit: Shrub. Habitat: Forest, woodland, wooded grassland, bushland, riparian, 950–1150 m. Vouchers: Mwachala G et al. EW614 & 3263, Mungai GM et al. EW1752 (EA).

***Ludiamauritiana* J.F.Gmel.** – Habit: Shrub/Tree. Habitat: Dry evergreen forest, riverine woodland, 650–1350 m. Vouchers: Kabuye CHS 82/130, Kabuye CHS et al. NMK Exped. 616, Mwachala G et al. EW64, Luke WRQ et al. 5343, Faden RB et al. 71/135, Medley KE 590, 607, 630, 641, 794 & 800 (EA).

***Scolopiarhamniphylla* Gilg** – Habit: Tree. Habitat: Rainforest, dry evergreen forest, 800–1000 m. Vouchers: Medley KE 587 & 673 (EA).

***Scolopiatheifolia* Gilg** – Habit: Tree. Habitat: Upland dry evergreen forest, 800–1000 m. Voucher: Medley KE 901 (EA).

**Trimeriagrandifolia(Hochst.)Warb.subsp.tropica (Burkill) Sleumer** – Habit: Shrub/Tree. Habitat: Rainforest, dry evergreen and riverine forest, 1250–1875 m. Vouchers: Watuma BM W0076 (EA, HIB), Kabuye CHS 82/86 & 145, Luke WRQ & Robertson SA 507, Wakanene KM & Mwangangi OM 459, Faden RB et al. NMK Exped. 327, Raler I 0043, Christenhusz MJM, Kamau P, Chase MW, Mbale M & Kyaa J 6636 (EA).

**F144. SALVADORACEAE**

2 Genera, 2 species

***Doberaglabra* (Forssk.) Juss. ex Poir.** – Habit: Tree. Habitat: *Acacia*-*Commiphora* scrub on rocky hillsides, 500–670 m. Vouchers: Medley KE 977, Zùmer M 39 (EA).

***Salvadorapersica* L.** – Habit: Shrub. Habitat: *Acacia*-*Commiphora* thicket, 550–650 m. Voucher: Medley KE 974 (EA).

**F1145. SANTALACEAE**

3 Genera, 4 species

***Osyridicarposschimperianus* (Hochst. ex A.Rich.) A.DC.** – Habit: Shrub. Habitat: Upland dry evergreen forest, deciduous woodland, riverine, 900–1100 m. Vouchers: Mwachala G et al. EW816 & 1503 (EA).

***Osyrislanceolata* Hochst. & Steud.** – Habit: Shrub. Habitat: Upland dry evergreen forest, mist forest and associated bushland, deciduous woodland, 650–2050 m. Vouchers: SAJIT–004119 & 005306, Watuma BM W0146 (EA, HIB), Kabuye CHS 82/147, Mwachala G et al. EW214, 430, 571, 925, 1185 & 1500, Wakanene KM & Mwangangi OM 341 & 442, Beentje HJ et al. NMK Exped. 848 & 1080, Faden RB et al. NMK Exped. 238, Drummond RB & Hemsley JH 4293, Raler I 0072, Medley KE 580, 741 & 803, Gardner HM 2994 (EA).

***Thesiumradicans* Hochst. ex A.Rich.** – Habit: Herb. Habitat: Short grassland, grazed places, rock crevices, 1040–1100 m. Voucher: Mwachala G et al. EW326 (EA).

***Thesiumschweinfurthii* Engl.** – Habit: Herb. Habitat: Upland grassland, mixed bushland, disturbed places and burnt areas, ca. 1420 m. Voucher: Mungai GM et al. EW3097A (EA).

**F146. SAPINDACEAE**

10 Genera, 13 species

***Allophylusabyssinicus* (Hochst.) Radlk.** – Habit: Tree. Habitat: Moist and dry upland forest margins, 1584–1875 m. Vouchers: Watuma BM W0133 & W0278 (EA, HIB), Faden RB et al. NMK Exped. 501, Faden RB et al. 71/262a (EA).

***Allophylusferrugineus* Taub.** – Habit: Shrub. Habitat: Forest, often by stream sides, 950–1875 m. Vouchers: Faden RB et al. NMK Exped 573, Mwachala G et al. EW930 (EA).

***Allophylusrubifolius* (Hochst. ex A.Rich.) Engl.** – Habit: Liana/Shrub. Habitat: Upland forest, woodland, *Commiphora*-*Acacia* bushland, grassland with scattered, 600–1875 m. Vouchers: Faden RB et al. NMK Exped. 522, Medley KE 448 & 456, Hildebrandt JM 2562 (EA).

***Blighiaunijugata* Baker** – Habit: Tree. Habitat: Evergreen forest, ca. 1200 m. Voucher: Faden RB et al. 71/59 (EA).

***Cardiospermumcorindum* L.** – Habit: Climber. Habitat: *Acacia*-*Commiphora* bushland and woodland, riverine, scrub on rocky hills, 600–900 m. Voucher: Hildebrandt JM 2565 (EA).

**Deinbolliaborbonicaf.glabrata Radlk.** – Habit: Shrub. Habitat: Riverine *Acacia* thorn bush and evergreen thicket, *Acacia*-*Combretum* woodland, 630–874 m. Vouchers: Drummond RB & Hemsley JH 4277, Kimeu JM, Meso M & Meroka D KEFRI 519 (EA).

***Deinbolliakilimandscharica* Taub.** – Habit: Shrub/Tree. Habitat: Evergreen forest, montane woodland, 650–1100 m. Vouchers: Medley KE 880, Mwachala G et al. EW236, Mungai GM et al. EW1350 (EA).

**DodonaeaviscosaJacq.subsp.angustifolia (L.f.) J.G.West** – Habit: Shrub. Habitat: Disturbed upland evergreen forest, woodland and thicket, 1158–1850 m. Vouchers: SAJIT–005305, Watuma BM W0093 (EA, HIB), Mwachala G et al. EW436 & 575, Wakanene KM & Mwangangi OM 247, Beentje HJ et al. NMK Exped. 876, Faden RB et al. NMK Exped. 34, Murray High School 21, Napier ER 1103, Raler I 0115, Heller E s.n. (EA).

***Filiciumdecipiens* (Wight & Arn.) Thwaites** – Habit: Tree. Habitat: Mixed forest in river valleys, 970–1260 m. Vouchers: Kabuye CHS et al. NMK Exped. 802, Mwachala G et al. EW1574, Beentje HJ et al. NMK Exped. 1182, Raler I 0122 (EA).

**Haplocoelumfoliolosum(Hiern)Bullocksubsp.strongylocarpum (Bullock) Verdc.** – Habit: Tree. Habitat: Riverine forest, grassland with *Combretum*, dry thicket and bushland, 650–1875 m. Vouchers: Mwachala G et al. EW3322, Wakanene KM & Mwangangi OM 511, Faden RB et al. NMK Exped. 517, Medley KE 861, Faden RB et al. 72/202, Raler I 0074 (EA).

**LecaniodiscusfraxinifoliusBakersubsp.vaughaniae (Dunkley) Friis** – Habit: Tree. Habitat: Riverine, deciduous bushland and woodland, 650–1000 m. Vouchers: SAJIT–004637 (EA, HIB), Kabuye CHS et al. NMK Exped. 646, Luke WRQ et al. 5345, Medley KE 569 (EA).

***Pancoviagolungensis* (Hiern) Exell & Mendonça** – Habit: Tree. Habitat: Moist evergreen forest, riverine forest, 1000–1640 m. Vouchers: Luke WRQ & Luke PA 9432B, Luke WRQ et al. 5322, Medley KE 662, 667, 918 & 934, Faden RB & Evans A 71/138 (EA).

***Pappeacapensis* Eckl. & Zeyh.** – Habit: Tree. Habitat: Grassland with scattered trees, woodland, 650–1100 m. Vouchers: Mwachala G et al. EW101, 202 & 974, Mungai GM et al. EW1663, Medley KE & Muasya J 820 (EA).

**F147. SAPOTACEAE**

6 Genera, 9 species

***Donellaviridifolia* (J.M.Wood & Franks) Aubrév. & Pellegr.** – Habit: Tree. Habitat: Upland moist evergreen forest, 1000–1640 m. Voucher: Medley KE 693 (EA).

***Englerophytumnatalense* (Sond.) T.D.Penn.** – Habit: Tree. Habitat: Upland rainforest, riverine forest, 1425–1875 m. Vouchers: SAJIT–005331 (EA, HIB), Faden RB et al. NMK Exped. 307, Beentje HJ et al. NMK Exped. 1005, Faden RB et al. 70/570, Raler I 0046 (EA).

***Gambeyagorungosana* (Engl.) Liben** – Habit: Tree. Habitat: Upland rainforest, commonly associated with *Podocarpus* sp. and *Ocoteausambarensis*, 1700–1875 m. Vouchers: Faden RB NMK Exped. 287, Drummond RB & Hemsley JH 4364 & 4365, Raler I 0033 (EA).

***Manilkaradiscolor* (Sond.) J.H.Hemsl.** – Habit: Tree. Habitat: Upland dry evergreen forest, upland rainforest in well drained sites, 800–1615 m. Vouchers: Watuma BM W0269 (EA, HIB), Medley KE 588 & 920 (EA).

***Manilkaramochisia* (Baker) Dubard** – Habit: Tree. Habitat: Deciduous bushland, dry scrub with trees, ca. 700 m. Voucher: Medley KE 952 (EA).

***Manilkarasulcata* (Engl.) Dubard** – Habit: Shrub/Tree. Habitat: Evergreen bushlands and thickets, 700–1100 m. Vouchers: SAJIT-005415 (EA, HIB), Faden RB & Faden AJ 74/466, Gilbert MG & Gilbert CI 6116, Bally PRO 13586, Medley KE 942 (EA).

***Mimusopsobtusifolia* Lam.** – Habit: Tree. Habitat: Riverine forest, montane woodland, 650–1000 m. Voucher: Medley KE 884 (EA).

***Mimusopssomalensis* Chiov.** – Habit: Tree. Habitat: Evergreen bushland. ca. 650 m. Voucher: Bytebier B 1737 (EA).

**Pouteriaadolfi-friedericii(Engl.)A.Meeusesubsp.adolfi-friedericii** – Habit: Tree. Habitat: Rainforest, 1350–1875 m. Vouchers: Faden RB et al. NMK Exped. 225, Raler I 0078, Faden RB et al. 71/237 (EA).

**F148. SCROPHULARIACEAE**

1 Genus, 1 species

***Buddlejapulchella* N.E.Br.** – Habit: Shrub. Habitat: Upland forest margins and upland evergreen bushland, 1700–1875 m. Vouchers: Faden RB et al. NMK Exped. 465 & 482, Drummond RB & Hemsley JH 4327, Gardner HM 2940, Raler I 0116 (EA).

**F149. SIMAROUBACEAE**

1 Genus, 1 species

***Bruceaantidysenterica* J.F.Mill.** – Habit: Shrub/Tree. Habitat: Evergreen rainforest, forest margins, secondary margins, 1500–2170 m. Vouchers: SAJIT–004522, 004526 & 006387, Watuma BM W0047 (EA, HIB), Wakanene KM & Mwangangi OM 627, Christenhusz MJM et al. 6618, Faden RB et al. NMK Exped. 163, Faden RB & Faden AJ 72/270, Hemp A 5191, Joanna B 9013 (EA).

**F150. SOLANACEAE**

5 Genera, 19 species

*Nicandraphysalodes (L.) Gaertn. – Habit: Herb. Habitat: Weed of cultivation, forest clearings, secondary bushland, wasteland, 1000–1200 m. Voucher: Mwachala G et al. EW1281 (EA).

*Nicotianatabacum L. – Habit: Herb. Habitat: Dry evergreen forest, bushland, homesteads, roadsides, 1100–1300 m. Voucher: Mwachala G et al. EW1186 (EA).

*Physalisperuviana L. – Habit: Herb. Habitat: Forest margins and clearings, disturbed areas, weed of cultivated places, 1675–1750 m. Vouchers: Watuma BM W0121 (EA, HIB), Wakanene KM & Mwangangi OM 552 (EA).

***Solanumaculeastrum* Dunal** – Habit: Shrub/Tree. Habitat: Forest margins, scrub, disturbed open places, 1402–1728 m. Vouchers: Watuma BM W0060 (EA, HIB), Napier ER 1131 (EA).

*Solanumaculeatissimum Jacq. – Habit: Shrub. Habitat: Moist and evergreen dry forest, *Acacia*-*Commiphora* woodland, 1425–1850 m. Voucher: Faden RB et al. NMK Exped. 413 (EA).

***Solanumanguivi* Lam.** – Habit: Shrub. Habitat: Forest and forest edges, disturbed areas, along roadsides, 1400–1875 m. Vouchers: Faden RB et al. NMK Exped. 45, 414 & 518, Beentje HJ et al. NMK Exped. 1108 (EA).

***Solanumdasyphyllum* Schumach. & Thonn.** – Habit: Subshrub. Habitat: Forest, wooded grassland, wasteland, 900–1250 m. Vouchers: Mwachala G et al. EW1267, Napier ER 1127 (EA).

***Solanumgiganteum* Jacq.** – Habit: Shrub. Habitat: Moist forest, secondary bushland, 1425–1850 m. Vouchers: Faden RB et al. NMK Exped. 376, Raler I s.n. (EA).

***Solanumincanum* L.** – Habit: Shrub. Habitat: Thickets, bushland, wooded grassland, 550–875 m. Vouchers: SAJIT–004609 (EA, HIB), Medley KE p.r. (EA).

***Solanummauense* Bitter** – Habit: Shrub. Habitat: Forest edge, secondary bushland, along roadsides, ca. 2024 m. Voucher: SAJIT–006361 (EA, HIB).

*Solanummauritianum Scop. – Habit: Shrub/Tree. Habitat: Forest margins, escaped from cultivated land, ca. 1584 m. Voucher: Watuma BM W0129 (EA, HIB).

***Solanumnakurense* C.H.Wright** – Habit: Subshrub. Habitat: *Acacia* scrub, roadsides, bare ground. Vouchers: Joanna B & Opiko 8758 & 8996 (EA).

***Solanumnigrum* L.** – Habit: Herb. Habitat: Forest edges, streambanks, *Acacia*-*Commiphora* woodland on rocky slopes, old cultivations, ca. 1800 m. Vouchers: Wakanene KM & Mwangangi OM 734, Kluguess LM 74 (EA).

*Solanumseaforthianum Andrews – Habit: Climber. Habitat: Semideciduous forest, grassland, farmland, plantations, 850–1100 m. Vouchers: Mwachala G et al. EW31, 117 & 1203, Cheseny CMC 30/72 (EA).

***Solanumschumannianum* Dammer** – Habit: Shrub. Habitat: Semi-deciduous and evergreen forest, secondary forest, margins of cultivation, 1402–2170 m. Vouchers: SAJIT–004527, Watuma BM W0075 (EA, HIB), Wakanene KM & Mwangangi OM 9 & 845, Christenhusz MJM et al. 6642, Drummond RB & Hemsley JH 4309, Verdcourt B & Polhill RM 2734, Napier ER 1134, Bally PRO 8754, Faden RB et al. NMK Exped. 128 & 323, Gillett JB, Burtt BL & Osborn RM 17104 (EA).

***Solanumterminale* Forssk.** – Habit: Liana. Habitat: Forest, forest margins, secondary forest, bushland, 1250–1875 m. Vouchers: SAJIT–004127 & 006392, Watuma BM W0070 & W0119 (EA, HIB), Mwachala G et al. EW1251 & 3543, Wakanene KM & Mwangangi OM 185 & 743, Faden RB et al. NMK Exped. 266 & 483, Beentje HJ et al. NMK Exped. 857, Napier ER 1132, Kluguess LM 67, Kirika P et al. s.n. (EA).

***Solanumtettense* Klotzsch** – Habit: Shrub. Habitat: *Acacia*-*Commiphora*-*Euphorbia* bushland, riverine, cultivation, 550–1000 m. Vouchers: Mwachala G et al. EW3299, Engler A 1971, Medley KE 988 (EA).

***Solanumvillosum* Mill.** – Habit: Herb. Habitat: Forest, riverine, bushland, cultivated and abandoned lands, 821–1612 m. Vouchers: Watuma BM W0303 (EA, HIB), Polhill RM & Kibuwa SP 974 (EA).

***Withaniasomnifera* (L.) Dunal** – Habit: Subshrub. Habitat: *Acacia*-*Commiphora* bushland, weed in old cultivations and disturbed places, 457–925 m. Vouchers: Mungai GM et al. EW3584, Katende AB, Lye K & Lye A 4786, Mrs. Robertson SA 3672, Bally PRO 8743, Greenway PJ & Kanuri K 12891 (EA).

**F151. STILBACEAE**

2 Genera, 4 species

***Hallerialucida* L.** – Habit: Tree. Habitat: Montane forest and forest margins, ca. 1850 m. Voucher: SAJIT–006370 (EA, HIB).

***Nuxiacongesta* R.Br. ex Fresen.** – Habit: Tree. Habitat: Upland rainforest, 1250–2170 m. Voucher: Kabuye CHS 82/81, Wakanene KM & Mwangangi OM 454 & 646, Beentje HJ 2189, Faden RB et al. NMK Exped. 104 & 542, Raler I 0107, Medley KE 660 & 996 (EA).

***Nuxiafloribunda* Benth.** – Habit: Tree. Habitat: Margins and relic patches of upland rainforest, 1350–1960 m. Vouchers: SAJIT–004564, Watuma BM W0097 (EA, HIB), Mungai GM et al. EW3090A, Luke WRQ & Luke PA 4199, Beentje HJ et al. NMK Exped. 874, 909 & 1077, Faden RB et al. NMK Exped. 25, 383 & 502, Drummond RB & Hemsley JH 4392, Raler I 0095, Dale IR 3783 (EA).

***Nuxiaoppositifolia* (Hochst.) Benth.** – Habit: Tree. Habitat: Riverine forest, 609–850 m. Vouchers: Mwachala G et al. EW3337, Gardner HM 3001 (EA).

**F152. TALINACEAE**

1 Genus, 2 species

***Talinumcaffrum* (Thunb.) Eckl. & Zeyh.** – Habit: Herb. Habitat: Rocky places in open woodland or grassland, 750–859 m. Vouchers: SAJIT–006416 (EA, HIB), Hucks M 466 (EA).

***Talinumportulacifolium* (Forssk.) Asch. ex Schweinf.** – Habit: Herb. Habitat: Open dry woodland or grassland in the hotter and drier parts, 600–1050 m. Vouchers: SAJIT–004616, Watuma BM W0174 (EA, HIB), Kabuye CHS et al. NMK Exped. 628, Mwachala G et al. EW3288, Gillett JB 19580, Bally PRO 8734 & 14157 (EA).

**F153. THYMELAEACEAE**

4 Genera, 5 species

**^NE^*Dicranolepisusambarica* Gilg** – Habit: Shrub. Habitat: Evergreen forest, 1425–1875 m. Vouchers: SAJIT–005318 & 006423 (EA, HIB), Beentje HJ et al. NMK Exped. 945, Faden RB et al. NMK Exped. 338 & 762, Faden RB et al. 69/830, 70/560 & 708, Wakanene KM & Mwangangi OM 277, 390 & 464, Gillett JB et al. 17154, Joanna B 9000, Christenhusz MJM et al. 6670, Gardner HM 2938, Raler I 0038, Muhesi 21 (EA).

***Englerodaphnesubcordata* (Meisn.) Engl.** – Habit: Shrub. Habitat: Upland dry evergreen forest and associated bushland, 1170–1450 m. Vouchers: Wakanene KM & Mwangangi OM 318 & 526, Mungai GM et al. EW3580, Joanna B 8914 (EA).

***Gnidiainvolucrata* Steud. ex A.Rich.** – Habit: Shrub. Habitat: Open wooded grassland, deciduous woodland and bushland, 1180–1371 m. Vouchers: Mwachala G et al. EW256, 381 & 3146, Luke WRQ et al. 5558, Gardner HM 2954 (EA).

***Gnidiastenophylla* Gilg** – Habit: Herb. Habitat: Grassland subject to burning and wooded grassland, 1350–1707 m. Vouchers: Beentje HJ et al. NMK Exped. 1138, Luke WRQ et al. 5559, Gardner HM 2955 (EA).

***Lasiosiphonlatifolius* (Oliv.) Brenan** – Habit: Shrub. Habitat: Upland evergreen bushland, wooded grassland, 550–1200 m. Vouchers: SAJIT–005307, Watuma BM W0002 (EA, HIB), Kabuye CHS et al. NMK Exped. 689, Mwachala G et al. EW100 & 518, Wakanene KM & Mwangangi OM 476, Medley KE 462 & 540, Christenhusz MJM et al. 6661, Gillett JB 18748, Bally PRO 8718 (EA).

**F154. URTICACEAE**

7 Genera, 11 species

***Droguetiadebilis* Rendle** – Habit: Herb. Habitat: Undergrowth of upland rainforest, 2000–2100 m. Voucher: SAJIT–004589 (EA, HIB).

***Elatostemamonticola* Hook.f.** – Habit: Herb. Habitat: Upland rainforest, in moist vegetation on forest floor, near streams, 1425–1875 m. Vouchers: Faden RB et al. NMK Exped. 773 & 774, Faden RB et al. 70/544, Christenhusz MJM et al. 6643, Gillett JB, Burtt BL & Osborn RM 17156 (EA).

***Obetiaradula* (Baker) Baker ex B.D.Jacks.** – Habit: Tree. Habitat: Evergreen or semi-evergreen bushland on basement complex, 650–1000 m. Voucher: Medley KEM pr.

***Parietariadebilis* G.Forst.** – Habit: Herb. Habitat: Upland rainforest, evergreen bushland in shade, under rocks, ca. 1350 m. Voucher: Mwachala G et al. EW3113A (EA).

***Pileajohnstonii* Oliv.** – Habit: Herb. Habitat: Upland rainforest and montane forest, 1400–1750 m. Vouchers: Watuma BM W0212 (EA, HIB), Wakanene KM & Mwangangi OM 233, 335 & 466, Faden RB et al. NMK Exped. 41 (EA).

***Pilearivularis* Wedd.** – Habit: Herb. Habitat: Upland rainforest, along streams and moist places, 1700–1875 m. Voucher: Faden RB et al. NMK Exped. 348 (EA).

**Pileac.f.usambarensis Engl.** – Habit: Herb. Habitat: Upland rainforest, 1425–2000 m. Vouchers: Faden RB et al. 72/275, Dale IR 7119, Drummond RB & Hemsley JH s.n. (EA).

**PileausambarensisEngl.var.usambarensis** – Habit: Herb. Habitat: Evergreen forest floor, disturbed forest patches, 1900–2195 m. Vouchers: Verdcourt B & Polhill RM 2723, Drummond RB & Hemsley HJ 4316 (EA).

**PileausambarensisEngl.var.engleri (Rendle) Friis** – Habit: Herb. Habitat: Upland rainforest in leaf litter, 1896–1950 m. Vouchers: SAJIT–004532 & 006349 (EA, HIB).

**PileausambarensisEngl.var.veronicifolia (Engl.) Friis** – Habit: Herb. Habitat: Upland rainforest in disturbed places, 2000–2170 m. Voucher: Faden RB et al. NMK Exped. 98 (EA).

***Pouzolziaparasitica* (Forssk.) Schweinf.** – Habit: Herb. Habitat: Rainforest along paths and streams, 1400–1640 m. Vouchers: Faden RB et al. NMK Exped. 13 (EA).

***Urerahypselodendron* (Hochst. ex A.Rich.) Wedd.** – Habit: Liana. Habitat: Upland rainforest especially in clearings and near edges, 1500–2200 m. Vouchers: Faden RB et al. NMK Exped. 101 & 200, Raler I 0148 (EA).

***Ureratrinervis* (Hochst.) Friis & Immelman** – Habit: Liana. Habitat: Lowland rainforest, riverine forest, along forest margins and clearings, 900–1640 m. Vouchers: Faden RB et al. 71/116, Medley KE s.n. (EA).

**F155. VERBENACEAE**

3 Genera, 6 species

*Lantanacamara L. – Habit: Shrub. Habitat: Forest clearings, roadsides, old cultivations, 650–1000 m. Voucher: Medley KEM s.r. (EA).

***Lantanaukambensis* (Vatke) Verdc.** – Habit: Subshrub. Habitat: Forest edges, wooded grassland, open woodland, old cultivations, granite rocks, 1700–1875 m. Voucher: Faden RB et al. NMK Exped. 223 (EA).

**Lantanaviburnoides(Forssk.)Vahlsubsp.kisi (A.Rich.) Verdc.** – Habit: Shrub. Habitat: Secondary scrub, bushed grassland, ca. 1000–1300 m. Vouchers: Bally PRO 8720, Gardner HM 2993 (EA).

***Lippiakituiensis* Vatke** – Habit: Subshrub. Habitat: Bushland, woodland, rough grassland on volcanic rocks, 1200–1300 m. Voucher: Murray High School 19 (EA).

***Lippiajavanica* (Burm.f.) Spreng.** – Habit: Shrub. Habitat: *Combretum* woodland, forest clearings, scrub bushland, 970–1180 m. Voucher: Mwachala G et al. EW357 (EA).

*Stachytarphetaurticifolia Sims – Habit: Subshrub. Habitat: Flood plain, bushland, roadside weed, 800–950 m. Voucher: Kabuye CHS et al. NMK Exped. 665 (EA).

**F156. VIOLACEAE**

3 Genera, 4 species

***Afrohybanthusenneaspermus* (L.) Flicker** – Habit: Herb. Habitat: Wooded grassland, cultivated and waste places, ca. 1160 m. Voucher: Mwachala G et al. EW298 (EA).

**Rinoreaangustifolia(Thouars)Baill.subsp.ardisiiflora (Oliv.) Grey-Wilson** – Habit: Shrub. Habitat: Evergreen forest, 1700–1875 m. Vouchers: Faden RB et al. NMK Exped. 476 & 536, Beentje HJ 2188 (EA).

***Rinoreaelliptica* (Oliv.) Kuntze** – Habit: Tree. Habitat: Lowland evergreen forest, 650–1000 m. Voucher: Medley KEM s.n. (EA).

***Violaabyssinica* Steud. ex Oliv.** – Habit: Herb. Habitat: Montane forest margins, forest clearings, moist grassland, 1400–2000 m. Vouchers: Drummond RB & Hemsely JH 4314, Faden RB et al. NMK Exped. 106, Christenhusz MJM et al. 6652, Gillett JB et al. 17086 & 17255, Joanna B 8815, Faden RB & Evans A 70/491 (EA).

**F157. VITACEAE**

3 Genera, 21 species

***Cissusadeyana* Masinde & L.E.Newton** – Habit: Climber. Habitat: Scattered scrub on rocky ground, dry *Acacia*-*Commiphora* bushland on rocky limestone ridge, 1128–1200 m. Vouchers: Luke PA & Luke WRQ 5512, Bally PRO 8123 (EA).

***Cissusalbiporcata* Masinde & L.E.Newton** – Habit: Climber. Habitat: Bushland in rocky areas, 440–500 m. Vouchers: Masinde PS 336 & 360, Mbugua PK 387a (EA).

***Cissusaphyllantha* Gilg** – Habit: Shrub/Liana. Habitat: *Acacia* desert thorn bush, ca. 800 m. Voucher: Drummond RB & Hemsley JH 4417 (EA).

***Cissuscactiformis* Gilg** – Habit: Climber. Habitat: *Commiphora*, *Acacia*, *Combretum* woodland, 487–609 m. Vouchers: Hucks M 1046, Sheldrick DB 8796, Napier ER 1022 (EA).

***Cissusoliveri* (Engl.) Gilg ex Engl.** – Habit: Climber. Habitat: Forest in riverine areas, valleys and open areas, 1400–1875 m. Vouchers: SAJIT–005349, Watuma BM W0019, 0062 & 0084 (EA, HIB), Faden RB et al. NMK Exped. 353, 428 & 800, Beentje HJ et al. NMK Exped. 846, Drummond RB & Hemsley JH 4382 (EA).

***Cissuspetiolata* Hook.f.** – Habit: Climber. Habitat: Forest edges, riverine forest, *Acacia* mixed bushland, rocky grounds with scattered trees, 1100–1358 m. Vouchers: Watuma BM W0233 (EA, HIB), Joanna B & Opiko 8763 (EA).

***Cissusphymatocarpa* Masinde & L.E.Newton** – Habit: Climber. Habitat: Forest edge, bushland and thicket, ca. 914 m. Voucher: Napier ER 939 (EA).

***Cissusquadrangularis* L.** – Habit: Liana. Habitat: Montane woodland, bushland, riverine thicket, 720–1000 m. Vouchers: Mwachala G et al. EW844 & 952A, Medley KE 949, Faden RB & Faden AJ 74/251, SAJIT s.n. (EA).

***Cissusrotundifolia* Vahl** – Habit: Liana. Habitat: Dry forest, *Acacia*-*Commiphora* scrub, 600–1150 m. Vouchers: Watuma BM W0183 (EA, HIB), Mwachala G et al. EW75, 173, 477 & 1182, Medley KE 948 (EA).

***Cissussciaphila* Gilg** – Habit: Climber. Habitat: Forest, riverine forest fringes, 1750–1800 m. Voucher: Christenhusz M, Chase MW, Kamau P, Mbale & Kyaa J 6672 (EA).

***Cissussylvicola* Masinde & L.E.Newton** – Habit: Liana. Habitat: Evergreen forest, forest on rocky hill, 800–950 m. Voucher: Kabuye CHS et al. NMK Exped. 700 (EA).

**^NE^*Cyphostemmabraunii* (Gilg & M.Brandt) Desc.** – Habit: Climber. Habitat: Margins evergreen forest with *Podocarpus* sp. and *Ocotea* sp., 1350–1450 m. Voucher: Beentje HJ et al. NMK Exped. 1081 (EA).

***Cyphostemmabuchananii* (Planch.) Desc. ex Wild & R.B.Drumm.** – Habit: Climber. Habitat: Evergreen forest, bushland, woodland, 1000–1875 m. Vouchers: Faden RB et al. NMK Exped. 521, 556 & 396 (EA), Mungai G et al. EW 2829 (EA).

***Cyphostemmacyphopetalum* (Fresen.) Desc. ex Wild & R.B.Drumm. subsp. cyphopetalum** – Habit: Climber. Habitat: Evergreen forest margins, 1700–1875 m. Vouchers: Watuma BM W0083 (EA, HIB), Faden RB et al. NMK Exped. 396 & 521 (EA).

**Cyphostemmacyphopetalumvar.nodiglandulosum (T.C.E.Fr.) Verdc.** – Habit: Climber. Habitat: Forest margins, grassland with scattered trees, 800–2170 m. Vouchers: Kabuye CHS et al. NMK Exped. 635, Faden RB et al. NMK Exped. 160 (EA).

***Cyphostemmadigitatum* (Lam.) Desc.** – Habit: Climber. Habitat: *Acacia*-*Grewia* bushland and thicket on rocky hillsides, ca. 600 m. Voucher: Hucks M 726 (EA).

***Cyphostemmahildebrandtii* (Gilg) Desc. ex Wild & R.B.Drumm.** – Habit: Climber. Habitat: Evergreen forest, *Commiphora*-*Terminalia*-*Acacia* bushland, 1220–1371 m. Vouchers: Hildebrandt JM 2436, Napier ER 1107 (EA).

***Cyphostemmajiguu* Verdc.** – Habit: Climber. Habitat: Riverine bush with scattered trees, 1200–1350 m. Voucher: SAJIT–005356 (EA, HIB).

***Cyphostemmaknittelii* (Gilg) Desc.** – Habit: Climber. Habitat: *Acacia*-*Commiphora*-*Combretum* bushland and woodland, ca, 884 m. Voucher: Watuma BM W0031 (EA, HIB).

***Cyphostemmathomasii* (Gilg & M.Brandt) Desc.** – Habit: Climber. Habitat: Forest margins, deciduous woodland, ca. 1422 m. Voucher: Watuma BM W0238 (EA, HIB).

***Rhoicissusrevoilii* Planch.** – Habit: Liana. Habitat: Secondary forest edges, woodland, bushland, grassland, 550–1569 m. Vouchers: Watuma BM W0253 (EA, HIB), Wakanene KM & Mwangangi OM 493, Medley KE 493, Gillett JB 19569 (EA).

***Rhoicissustridentata* (L.f.) Wild & R.B.Drumm.** – Habit: Liana. Habitat: Forest edges, thicket and scrub often on rock crevices, ca. 1769 m. Vouchers: Kabuye CHS 82/84, Raler I 0132 (EA).

**F158. XIMENIACEAE**

1 Genus, 1 species

***Ximeniacaffra* Sond.** – Habit: Shrub/Tree. Habitat: Dry woodland and bushland, 500–650 m. Voucher: Medley KE 967 (EA).

**F159. ZYGOPHYLLACEAE**

1 Genus, 2 species

***Balanitesaegyptiaca* (L.) Delile** – Habit: Shrub/Tree. Habitat: Montane woodland, deciduous bushland, scattered tree grassland, 550–1000 m. Voucher: Medley KE 485 (EA).

**BalanitespedicellarisMildbr. & Schltr.subsp.pedicellaris** – Habit: Shrub/Tree. Habitat: Deciduous bushland, ca. 560 m. Vouchers: Medley KE 770, Mildbraed 19 (EA).
